# Prevalence of hepatitis E virus in China from 1997 to 2022: a systematic review and meta-analysis

**DOI:** 10.3389/fpubh.2023.1243408

**Published:** 2023-09-07

**Authors:** Kexin Cao, Xiaoyue Wu, Mengya Yang, Can Chen, Xiaobao Zhang, Daixi Jiang, Yuxia Du, Mengsha Chen, Yue You, Wenkai Zhou, Jiaxing Qi, Dingmo Chen, Rui Yan, Ziping Miao, Shigui Yang

**Affiliations:** ^1^Department of Emergency Medicine, Second Affiliated Hospital, Department of Public Health, State Key Laboratory for Diagnosis and Treatment of Infectious Diseases, The Key Laboratory of Intelligent Preventive Medicine of Zhejiang Province, Zhejiang University School of Medicine, Hangzhou, China; ^2^Zhejiang Provincial Center for Disease Control and Prevention, Hangzhou, China

**Keywords:** hepatitis E, seroprevalence, risk factors, meta-analysis, China

## Abstract

**Introduction:**

Several studies have reported on hepatitis E virus (HEV) prevalence in various regions of China, but the results vary widely. Herein, we conducted a systematic review and meta-analysis to assess the seroprevalence, RNA-positive rate, genotype distribution of HEV in China, and its risk factors.

**Methods:**

We included 208 related studies involving 1,785,569 participants published between 1997 and 2022. Random-effects models were used to pool prevalence, and subgroup analyses were conducted by population, gender, age, study period, regions, and rural–urban distribution. The meta regression models and pooled odds ratios (OR) were performed to identify risk factors for HEV infections.

**Results:**

The pooled anti-HEV IgG, IgM, and Ag seroprevalence, and RNA detection rates in China from 1997 to 2022 were 23.17% [95% confidence interval (CI): 20.23–26.25], 0.73% (95% CI: 0.55–0.93), 0.12% (95% CI: 0.01–0.32), and 6.55% (95% CI: 3.46–12.05), respectively. The anti-HEV IgG seropositivity was higher in the occupational population (48.41%; 95% CI: 40.02–56.85) and older adult aged 50–59 years (40.87%; 95% CI: 31.95–50.11). The dominant genotype (GT) of hepatitis E in China was GT4. Notably, drinking non-tap water (OR = 1.82; 95% CI: 1.50–2.20), consumption of raw or undercooked meat (OR = 1.47; 95% CI: 1.17–1.84), and ethnic minorities (OR = 1.50; 95% CI: 1.29–1.73) were risk factors of anti-HEV IgG seroprevalence.

**Discussions:**

Overall, the prevalence of hepatitis E was relatively high in China, especially among older adults, ethnic minorities, and humans with occupational exposure to pigs. Thus, there is a need for preventive measures, including HEV infection screening and surveillance, health education, and hepatitis E vaccine intervention in high-risk areas and populations.

**Systematic review registration:**

https://www.crd.york.ac.uk/prospero/, identifier CRD42023397036.

## 1. Introduction

The hepatitis E virus (HEV) is the RNA virus that causes hepatitis E. In the 1980s, Balayan and other scholars used immuno-electron microscopy to discover virus-like particles in the feces of infected volunteers (1). In a study published in 1990, the viral genome was successfully cloned and named HEV (2). HEV belongs to the hepeviridae family, classified into Orthohepevirus and Piscihepevirus. Orthohepatitis virus has four species: A, B, C, and D. The Piscihepevirus only includes the Cutthroat trout virus (3). Eight HEV genotypes (GTs) have been identified in the Orthohepevirus A: GT1 and GT2 infect only humans, and GT3, GT4, and GT7 are zoonotic viruses (4). GT3 and 4 have been detected in several animals, including pigs (5), rabbits (6), and cattle (7); GT5 and GT6 have only been detected in wild boars (8); studies have found GT7 and GT8 in camels (9, 10).

Previous studies have confirmed many risk factors for hepatitis E infection. In areas with poor sanitation, exposure to contaminated water (11) is a major risk factor. The two outbreaks in Shimla in 2015–2016 (12) and Yavatmal in 2019 (13) were caused by contaminated drinking water. In high-income countries, exposure to infected animals and consumption of HEV-contaminated food are proven risk factors. Additionally, in persons living with the human immunodeficiency virus, a low CD4 cell count (<200 cells/mm^3^) can be considered a risk factor for HEV infection (14). HEV infection is more common in males, possibly due to greater exposure to contaminated water (15).

An analysis published in 2020 systematically assessed the global prevalence of hepatitis E and showed that one in eight individuals (about 939 million) are infected with hepatitis E, and 15–110 million people have been recently or persistently infected with HEV (16). Globally, it is estimated that there are 20 million HEV infections and 3.3 million symptomatic cases each year (17). A study of hepatitis E GT1 and 2 involving 71% of the global population estimated that there were 3.4 million symptomatic cases, 70,000 deaths, and 3,000 stillbirths in 2005 (18). In addition, large hepatitis E outbreaks pose a considerable public health burden. For instance, 79,091 people were affected in Kanpur, India, in 1991 (19); 2,621 cases and 45 deaths were reported in Darfur, Sudan, in 2004 (20); and 1,293 cases in Chad in 2016–2017 (21). A study indicated that the seroprevalence of hepatitis E in people aged 15–30 years has stabilized at 30–40% in Asia, Africa, Latin America, Mexico, and West Africa, while in some high-income countries (including East Asia, Central Europe, etc.), the seroprevalence increases steadily with age, ranging from 7 to 21% (22). In Europe, the seroprevalence of hepatitis E ranges from 0.60 to 52.50% (23, 24) and is estimated at 6.00% in the United States (25). China is considered an endemic region for hepatitis E. In the 1980s, the hepatitis E GT1 outbreak in Xinjiang, China, caused 120,000 cases and 765 deaths (26). Additionally, a large cohort study involving 6,269 participants showed that the prevalence of anti-HEV IgG and IgM in China was 4.78 and 0.14%, respectively (27). In 2018, an analysis including 1,864 blood donors found that the anti-HEV IgG, IgM, and IgA in Dali, China, were 13.36, 1.13, and 1.82% (28). Moreover, a study of 19,762 pregnant women in Qujing, Yunnan Province, China, from 2019 to 2020 showed that the seroprevalence of hepatitis E in pregnant women was 11.60% (29). Over the past few decades, there has been a shift in the predominant genotype of hepatitis E in China, with GT1 infections caused by contaminated water (often outbreaks) gradually transitioning to zoonotic GT4 sporadic infections (30).

However, the prevalence of hepatitis E reported in these studies varied widely and mostly focused only on anti-HEV IgG positivity rates. Anti-HEV IgG is a marker of previous infection, anti-HEV IgM indicates acute infection, HEV RNA is the gold standard for detecting HEV infection (4), and anti-HEV Ag is highly specific and sensitive (31). Thus, a systematic study is urgently needed to reliably estimate the status of hepatitis E infection in China. The seroprevalence of hepatitis E in China varies greatly by gender, age, population, period, and region, which is not conducive to assessing the burden of hepatitis E infection in China. This study aimed to systematically evaluate the seroprevalence and risk factors of hepatitis E in China.

## 2. Materials and methods

This study is consistent with the Preferred Reporting Items for Systematic Reviews and Meta-Analyses statement (32) and has been registered on the PROSPERO website (CRD42023397036).

### 2.1. Definition of the study population, hepatitis E infection, and hepatitis E seroprevalence

Our meta-analysis divided populations into the general population, occupational population, pregnant women, volunteer blood donors, and hospital attendees. The general population referred to community-dwelling populations with unknown hepatitis E infection status. The occupational population was those who worked in occupations associated with pigs or with known risk factors for hepatitis E, such as pig farm workers, butchers, and food processing industry practitioners. Hospital attendees were those who came into medical facilities for consultation, examination, or treatment, including patients (acute hepatitis and asymptomatic patients) and healthy individuals who attended medical examinations.

Hepatitis E virus infection can be diagnosed either indirectly by detecting anti-HEV antibodies in the serum or directly by detecting HEV RNA or capsid antigen in the blood or other body fluids (4). Hepatitis E seroprevalence was defined as the proportion of hepatitis E infection in the population, calculated as the number of infected persons divided by the total population.

### 2.2. Data sources and searches

All publications about hepatitis E infection in China between 1997 and 2022 were searched in the Chinese databases (China National Knowledge Infrastructure, WanFang, and WeiPu) and English databases (PubMed, Web of Science, Embase, and Cochrane). We used the following subject terms for the search: [“Hepatitis E” OR “HEV” OR “Hepatitis E antibody” OR “ET-NANBH” OR “Hepatitis, Viral, Non-A, Non-B, Enterically-Transmitted” OR “Epidemic Non-A, Non-B Hepatitis” OR “Hepatitis E virus”] AND [“Prevalence” OR “Infections” OR “Serology”] AND [“Chinese” OR “China”] AND [(“1997” [Date - Publication]: “2022” [Date - Publication])].

### 2.3. Study selection and data extraction

The inclusion criteria for this meta-analysis were: (1) Studies with data on hepatitis E IgG and IgM antibodies, antigens and RNA; (2) Studies that did not conduct trials or interventions; (3) Studies conducted from 1997 to 2022 in China; (4) The study subjects were human beings; and (5) Studies with a sample size of more than 50 to prevent random error.

We excluded the following studies: (1) Systematic reviews, meta-analyses, case reports, opinions, and abstracts; (2) Studies with no data on humans; (3) Studies not conducted in China from 1997 to 2022; (4) Studies with incomplete or duplicate data; (5) Studies on HEV outbreaks; (6) Studies conducted on high-risk groups (men who have sex with men, female sex workers), transplant patients, drug addicts, hemodialysis patients, individuals living with the human immunodeficiency virus, and other immunodeficient people.

The following data were extracted from the included studies: first author, year of publication and implementation, population, age, gender, region, urban and rural distribution, risk factors, sample size, and the number of anti-HEV antibody-positive, antigen-positive and HEV RNA-positive individuals. Two reviewers (KC and MY) independently determined whether the studies met the inclusion and exclusion criteria and collected data. Any inconsistencies were resolved through mutual discussion or by a third reviewer (CC).

### 2.4. Quality assessment

We used the Joanna Briggs Institute (JBI) checklist to assess the quality of prevalence studies (Supplementary Table S1). The JBI checklist is a quality assessment tool proposed by the JBI Center for Evidence-Based Health Care in Australia. The JBI scale has nine items scored on several dimensions, including sampling, sample size, coverage, measurement method, data analysis method, and response rates. Each item is evaluated with yes, no, unclear, or not applicable. A “yes” result scores 1 point and 0 points otherwise. The scores of the nine items are summed. A total score of 0–3 is C grade, indicating a high risk of bias and need to be excluded; a total score of 4–6 is rated B, indicating a moderate risk of bias, and a total score of 7–9 is rated A, indicating a low risk of bias. A and B-grade studies were included to ensure the comprehensiveness of the included studies.

Literature scoring was done independently by two researchers (KC and XW), and studies with inconsistent scores were judged by a third researcher (CC).

### 2.5. Statistical analysis

Random-effects models were used to pool the rates as forest plots showed high heterogeneity (I^2^ > 50%). When the raw data did not follow a normal distribution, it was transformed using the arcsine transformation or Freeman-Tukey double arcsine transformation. Subgroup analyses and meta-regression were performed to investigate sources of heterogeneity. Individuals were further divided into subgroups according to population, gender, age, study period, region, and urban and rural distribution. In studies that lasted for several years, we divided the study period by median implementation time. Considering that there is a certain time interval between the study publication time and implementation time, the research period of studies without an implementation time was divided by the year of publication minus 2 years.

In addition, meta-regression was used to screen for factors influencing heterogeneity. The following variables were included in univariable meta-regression: population, gender, age, study period, region, kits, and rural and urban distribution. Variables with *p* values less than 0.05 were included in multivariable meta-regression.

Adjusted odd ratios (OR) of anti-HEV IgG seropositivity were pooled to report risk factors for hepatitis E infection. We pooled adjusted ORs with the following risk factors: nation, consumption of raw or undercooked meat, water sources, and working years for the occupational population. We excluded studies with JBI quality scores ≤5 and sample sizes <200, < 300, and < 500 to evaluate the robustness and reliability of the overall pooled rate. The meta-analyses were conducted by the R version 4.2.1 using the *“metaprop”* and *“metareg”* packages.

## 3. Results

### 3.1. Search results

**Figure 1:**
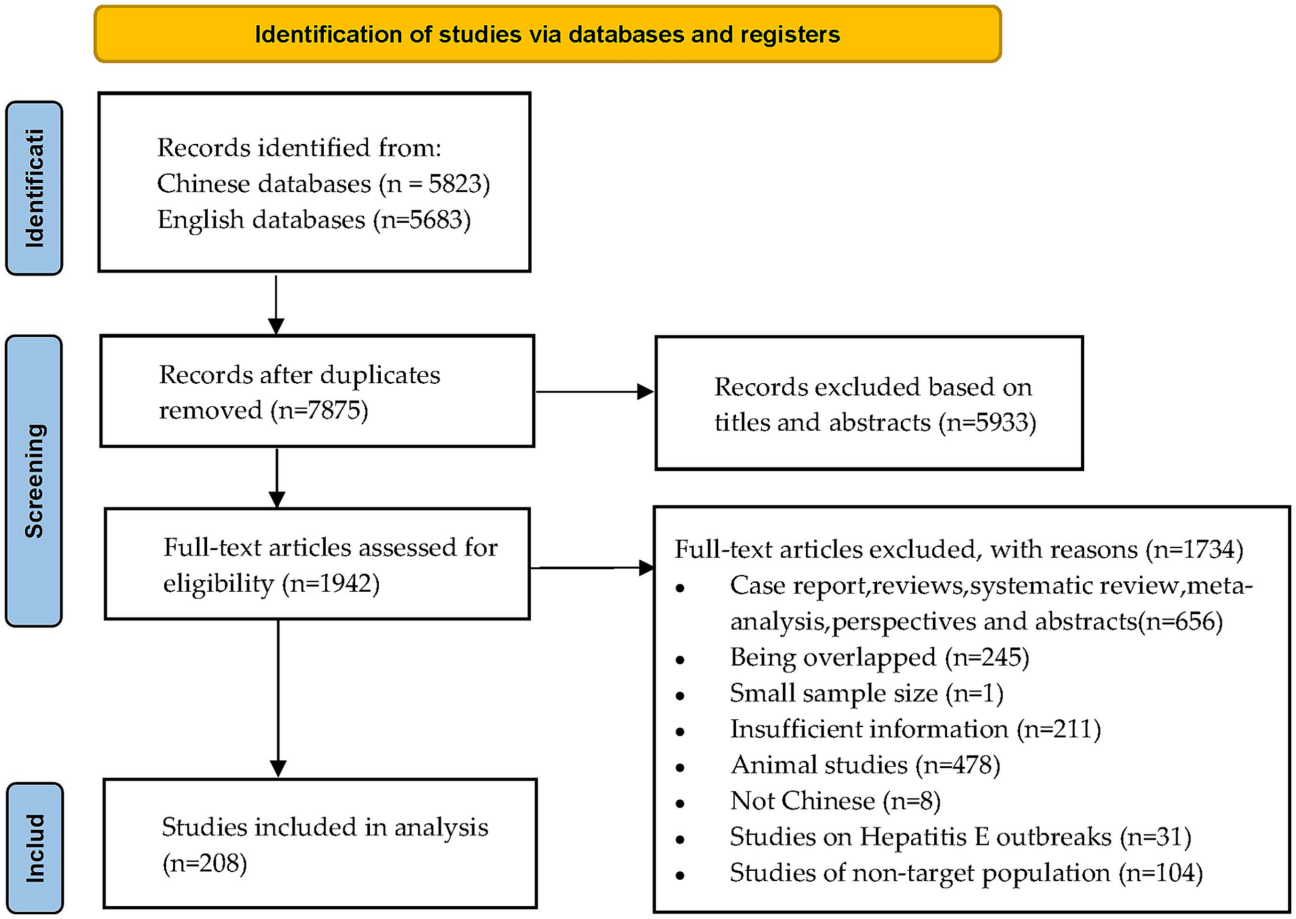
Flowchart of study selection.

We initially retrieved 11,506 publications. After removing duplicates, 5,933 records were excluded after reading the titles and abstracts. The remaining 1,942 full articles were assessed, and 1,734 publications were excluded according to the inclusion and exclusion criteria. Finally, we included 208 studies published between 1997 and 2022 involving 1,785,569 participants (Figure 1).

### 3.2. Prevalence of hepatitis E in China

We systematically assessed the infection of HEV in China from 1997 to 2022. The pooled seroprevalence of anti-HEV IgG in China from 152 studies was 23.17% [95% confidence interval (CI): 20.23–26.25; Supplementary Figure S1]. The overall estimated anti-HEV IgM seroprevalence of China based on 115 studies was 0.73% (95% CI: 0.55–0.93; Supplementary Figure S2). Sixteen studies were included, and the pooled estimated anti-HEV antigen positivity rate in China was 0.12% (95% CI: 0.01–0.32; Supplementary Figure S3). We pooled data from 19 studies to estimate an HEV RNA detection rate of 6.55% (95% CI: 3.46–12.05; Supplementary Figure S4).

**Figure 2:**
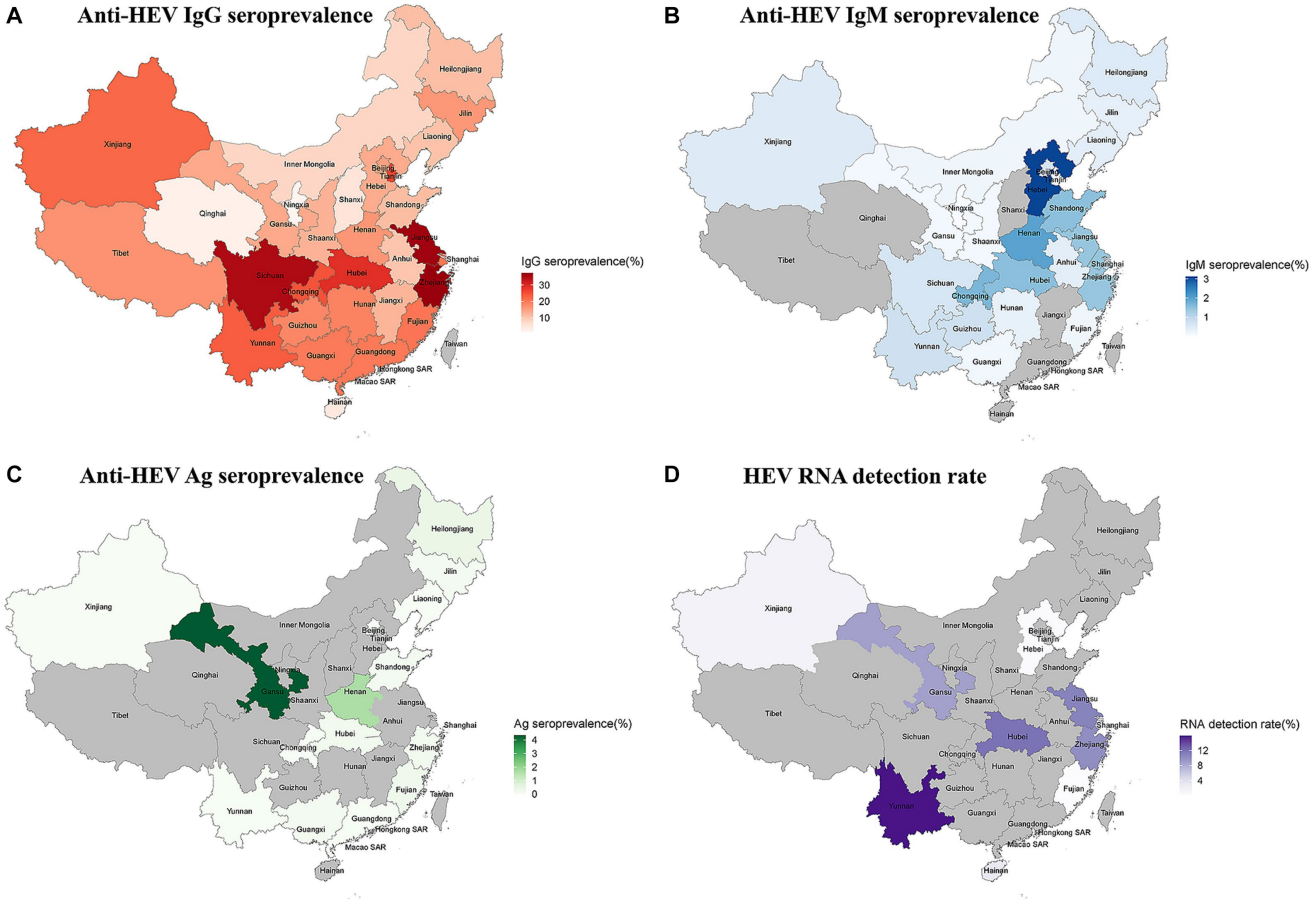
**(A)** The anti-HEV IgG seroprevalence in China; **(B)** The anti-HEV IgM seroprevalence in China; **(C)** The anti-HEV Ag seroprevalence in China; and **(D)** The anti-HEV RNA detection rate in China.

The provinces of Zhejiang (anti-HEV IgG seroprevalence: 37.24%; 95% CI: 26.15–49.06) and Hebei (anti-HEV IgM seroprevalence: 3.13%; 95% CI: 1.98–4.52) had the highest prevalence of HEV antibodies. Gansu province had the highest rate of antigen positivity (4.35, 95% CI: 3.52–5.32). Yunnan (15.91%; 95% CI: 0.00–48.69) had the highest RNA detection rate (Figure 2; Supplementary Figures S5–S8).

### 3.3. Geographical distribution of hepatitis E genotypes

**Figure 3:**
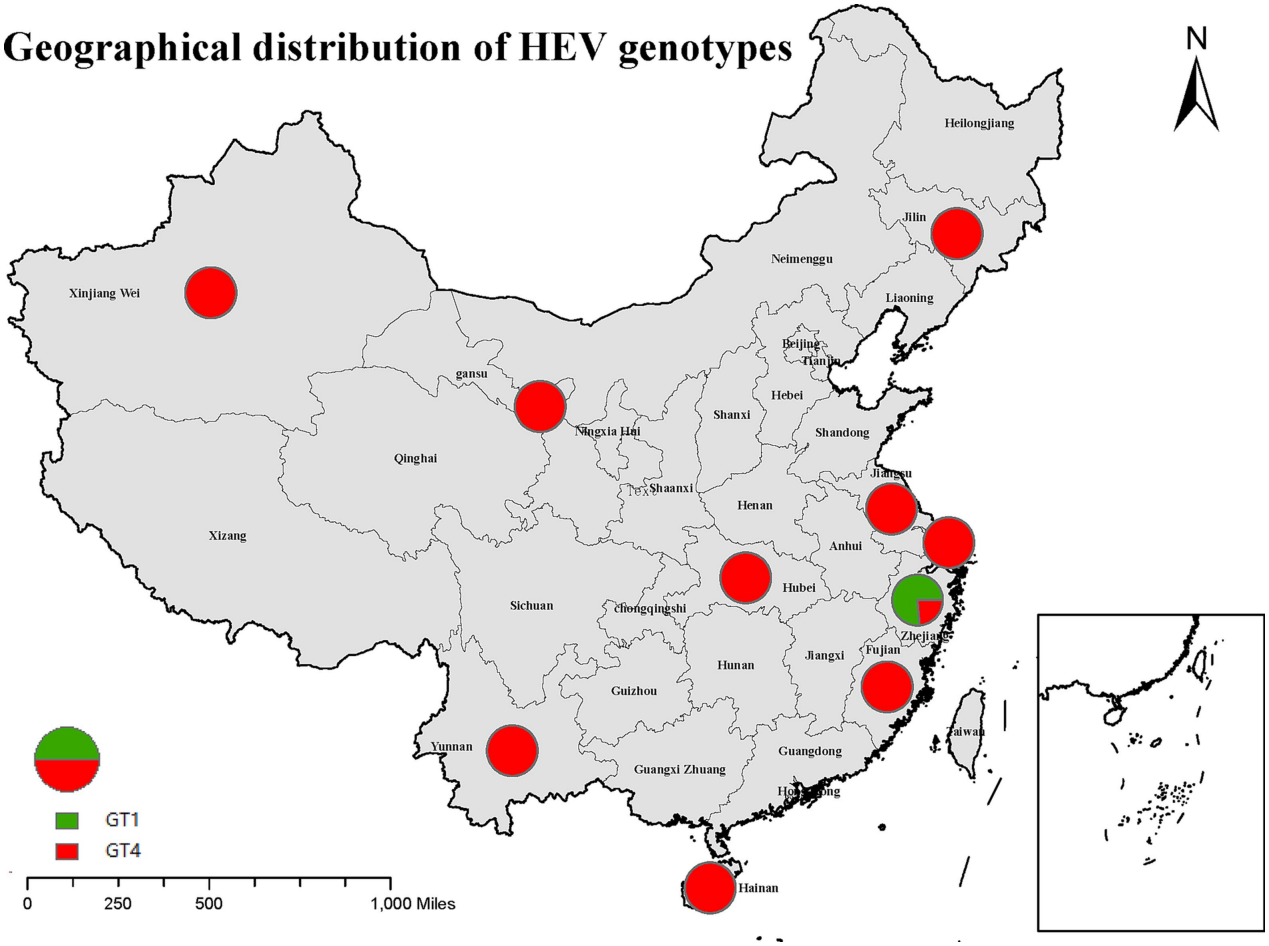
Geographical distribution of hepatitis E genotypes in China.

Twenty studies involving 10 provinces and cities showed that the dominant genotype for hepatitis E in China was GT4 (Figure 3). GT4 was the hepatitis E genotype in nine provinces and cities, including Jiangsu, Hainan, Jilin, Xinjiang, Shanghai, Yunnan, Gansu, Hubei, and Fujian. Six studies reported the subtypes of hepatitis E GT4, including 4a (three cases), 4d (14 cases), 4f (22 cases), and 4 h (one case). The 4a subtype was predominantly found in Xinjiang, Gansu, and Jiangsu; 4d was mainly found in Gansu; 4f was reported in Hubei, Shanghai, Jiangsu, and Gansu; and the only strain classified as subtype 4 h was reported in Fujian. A sequence isolated from one eligible donor was clustered between GT2 and GT4i. However, the included study did not provide any information on the subtypes of GT1. Furthermore, only 11 cases of GT1 and four cases of GT4 were found in Zhejiang Province. Before 2006, the percentage of GT1 was 83.33% (10/12), and GT4 was 16.67% (2/10) in Zhejiang Province, and the percentages after 2006 were 33.33% (1/3) and 66.67% (2/3), respectively. This indicates that the hepatitis E genotype in Zhejiang Province gradually changes from GT1 to GT4.

### 3.4. Estimated the prevalence of hepatitis E by population, gender, age, type of kits, region, and study period

The population was divided into five groups. The highest anti-HEV IgG positivity rate was found in the occupational population (48.41%; 95% CI: 40.02–56.85), while the population with the highest anti-HEV IgM seroprevalence was hospital attendees (1.93%; 95% CI: 0.66–3.83; Table 1; Supplementary Figures S7, S8).

**Table 1:** HEV antibody positivity rates in different populations.

Population	HEV IgG				HEV IgM			
	Number of studies	Events	Total	IgG (95% CI)	Number of studies	Events	Total	IgM (95% CI)
The general population	111	58,140	413,997	21.49 (18.04–25.16)	84	2,242	1,102,600	0.49 (0.33–0.67)
Occupational population	24	4,531	10,598	48.41 (40.02–56.85)	10	73	4,974	1.47 (0.57–2.74)
Volunteer blood donors	22	36,281	118,845	24.36 (19.58–29.48)	19	1,400	123,686	1.22 (1.03–1.42)
Hospital attendees	10	5,697	25,081	27.53 (21.05–34.53)	9	562	43,054	1.93 (0.66–3.83)
Pregnant women	16	3,924	32,678	13.17 (11.19–15.28)	10	210	28,041	1.87 (0.97–3.05)

**Figure 4:**
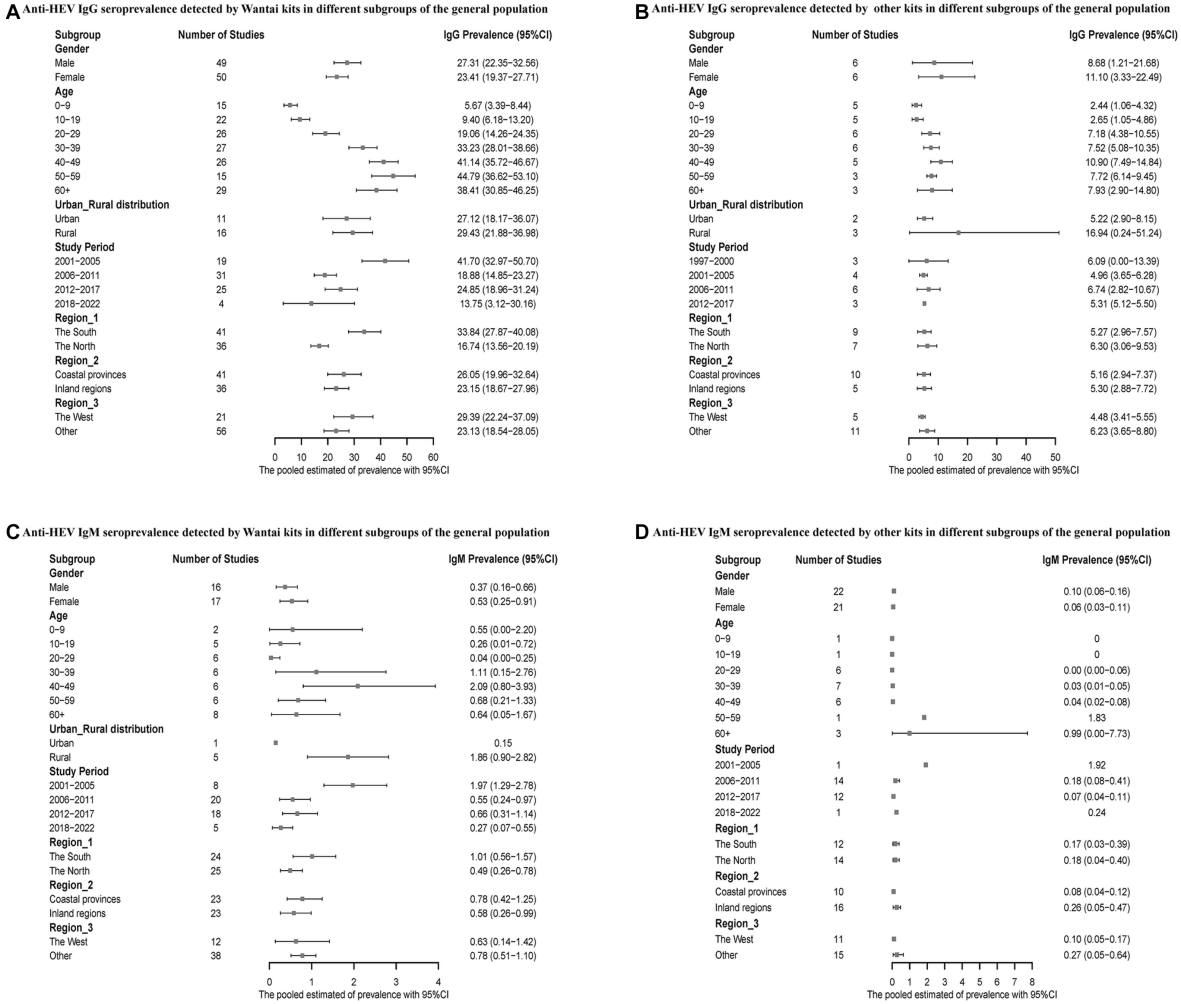
**(A)** Anti-HEV IgG seroprevalence detected by Wantai kits in different subgroups of the general population; **(B)** Anti-HEV IgG seroprevalence detected by other kits in different subgroups of the general population; **(C)** Anti-HEV IgM seroprevalence detected by Wantai kits in different subgroups of the general population; and **(D)** Anti-HEV IgM seroprevalence detected by other kits in different subgroups of the general population.

In addition, subgroup analyses were performed for the general population. When tested with the Wantai kits, men had higher anti-HEV IgG and lower IgM seroprevalence, while the results showed the opposite with the other kits. Overall, the prevalence of hepatitis E antibody positivity increased with age. Notably, hepatitis E antibody prevalence was higher in rural areas than in urban areas. The Wantai testing kit detected a higher prevalence of hepatitis E infection in southern and coastal China, while the results of other kits were reversed. In Western China, the combined results indicated a higher seroprevalence of anti-HEV IgG antibodies detected by the Wantai kits, while other kits showed a lower rate. Additionally, regardless of the kits used, the seroprevalence of anti-HEV IgM antibodies was lower in the western region. Overall, the seroprevalence of hepatitis E infection was higher from 2001 to 2005 (Figure 4).

**Figure 5:**
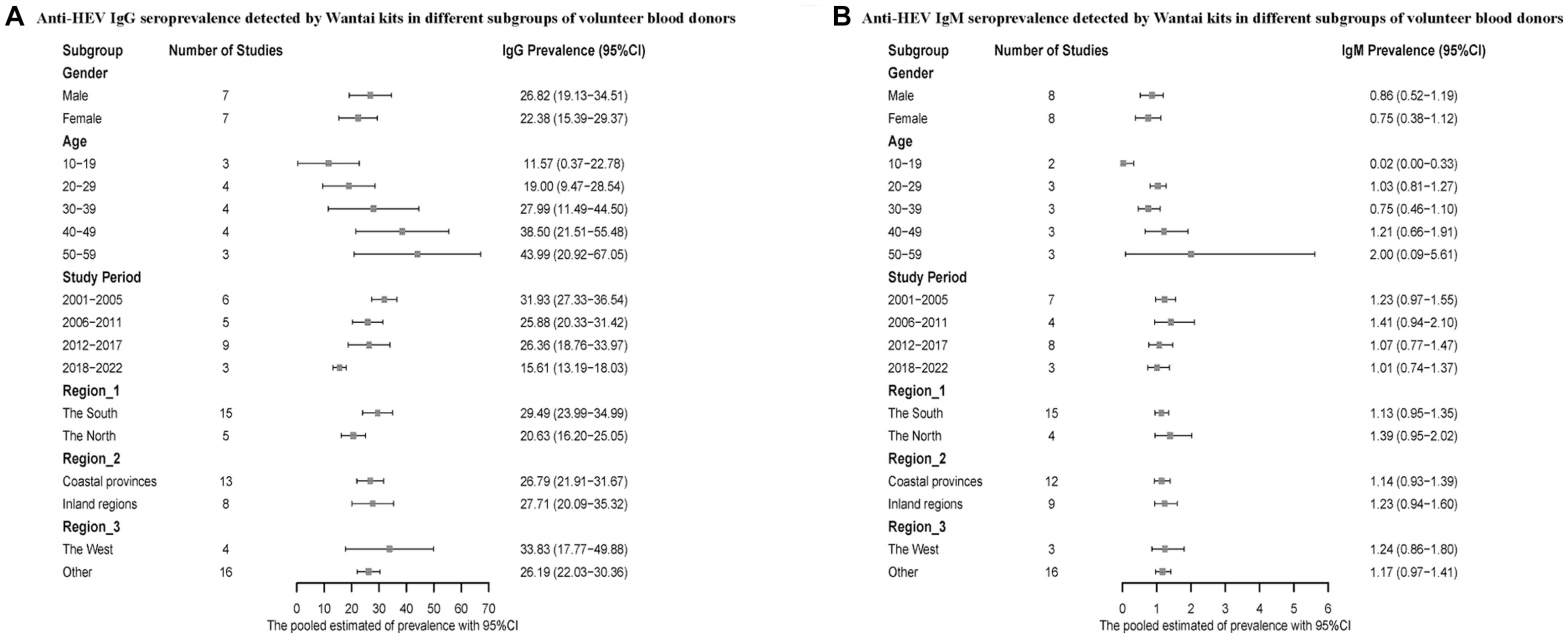
**(A)** Anti-HEV IgG seroprevalence detected by Wantai kits in different subgroups of volunteer blood donors; **(B)** Anti-HEV IgM seroprevalence detected by Wantai kits in different subgroups of volunteer blood donors.

We also conducted a subgroup analysis on voluntary blood donors. Men had a higher positive rate of hepatitis E antibodies compared to women. The infection rate of hepatitis E was higher in the 50–59 age group compared to the 10–19 age group. The prevalence of HEV antibodies was higher during the period of 2001–2005 compared to 2018–2022. Regarding the geographical distribution, Southern China reported higher anti-HEV IgG and lower IgM seroprevalence, and the infection rate was higher in both the inland regions and western China (Figure 5).

### 3.5. Risk factors of HEV infection

We performed univariable and multivariable meta-regression analyses to explore risk factors of HEV infection. The results of the univariable analysis showed that population, age, type of kits, North–South division in China, and study period were statistically significantly associated with the prevalence of HEV IgG. These variables were incorporated into a multivariable model, and the analysis indicated that the risk of hepatitis E infection in the occupational population was 1.19 times that of the general population (OR = 1.19; 95% CI: 1.06–1.33), and the risk in the 50–59 age group was 1.53 times higher than the 0–9 age group (OR = 1.53, 95% CI: 1.39–1.69). The detection rate of the Wantai kit for hepatitis E IgG antibodies was higher than that of other kits (OR = 1.26; 95% CI: 1.16–1.36), and Southern China reported a higher risk of detecting IgG antibodies than Northern China (OR = 1.10; 95% CI: 1.04–1.16; Table 2). Furthermore, a multivariable meta-regression model for HEV IgM seroprevalence found that the Wantai kits had a lower detection rate than other kits, and Southern China reported a higher prevalence than Northern China (Supplementary Table S2).

**Table 2:** Univariable and multivariable meta-regression models of anti-HEV IgG positive rates.

Variable	Univariable regression		Multivariable regression		
	β (95% CI)	*p* value	β (95% CI)	*p* value	OR (95% CI)
Population					
The general population (reference)					
Occupational population	0.2868 (0.1954, 0.3783)	<0.0001^*^	0.1704 (0.0571, 0.2837)	0.0032^*^	1.19 (1.06, 1.33)
Pregnant women	−0.1102 (−0.2184, −0.0020)	0.0459^*^	−0.1314 (−0.2931, 0.0304)	0.1114	
Hospital attendees	0.0709 (−0.0624, 0.2043)	0.2971	0.0648 (−0.0386, 0.1682)	0.2193	
Volunteer blood donors	0.0329 (−0.0612, 0.1270)	0.4926	−0.0277 (−0.1449, 0.0896)	0.6439	
Age (years)					
0–9 (reference)					
10–19	0.0536 (−0.0535, 0.1607)	0.3268	0.0611 (−0.0313, 0.1535)	0.1948	
20–29	0.1731 (0.0716, 0.2746)	<0.0008^*^	0.1841 (0.0943, 0.2739)	<0.0001^*^	1.20 (1.10, 1.32)
30–39	0.3205 (0.2192, 0.4219)	<0.0001^*^	0.3175 (0.2285, 0.4065)	<0.0001^*^	1.37 (1.26, 1.50)
40–49	0.3941 (0.2920, 0.4961)	<0.0001^*^	0.3935 (0.3035, 0.4836)	<0.0001^*^	1.48 (1.35, 1.62)
50–59	0.4362 (0.3258, 0.5465)	<0.0001^*^	0.4257 (0.3273, 0.5240)	<0.0001^*^	1.53 (1.39, 1.69)
60+	0.4165 (0.3107, 0.5222)	<0.0001^*^	0.4143 (0.3175, 0.5111)	<0.0001^*^	1.51 (1.37, 1.67)
Gender					
Female (reference)					
Male	0.0389 (−0.0211, 0.0989)	0.2040			
Type of kits					
Other (reference)					
WanTai	0.2974 (0.2046, 0.3902)	<0.0001^*^	0.2309 (0.1552, 0.3066)	<0.0001^*^	1.26 (1.16, 1.36)
Region_1					
The north (reference)					
The south	0.1309 (0.0592, 0.2026)	0.0003^*^	0.0930 (0.0396, 0.1465)	0.0006^*^	1.10 (1.04, 1.16)
Region_2					
Coastal provinces (reference)					
Inland regions	−0.0351 (−0.1083, 0.0381)	0.3471			
Region_3					
Other (reference)					
The west	−0.0127 (−0.0967, 0.0713)	0.7671			
Study period					
1997–2000 (reference)					
2001–2005	0.4424 (0.2541, 0.6308)	<0.0001^*^	0.0121 (−0.1234, 0.1476)	0.8610	
2006–2011	0.2479 (0.0627, 0.4331)	0.0087^*^	−0.0317 (−0.1722, 0.1088)	0.6579	
2012–2017	0.2812 (0.0947, 0.4677)	0.0031^*^	−0.0327 (−0.1698, 0.1044)	0.6401	
2018–2022	0.2090 (−0.0078, 0.4259)	0.0589	−0.0018 (−0.2749, 0.2713)	0.9897	
Urban_rural distribution					
Rural (reference)					
Urban	−0.0632 (−0.1665, 0.0400)	0.2300			

* Statistically significant at *p* < 0.05.

We also investigated the risk factors of anti-HEV IgG positive rates by pooling adjusted ORs. The pooled results showed that drinking non-tap water (OR = 1.82; 95% CI: 1.50–2.20), consumption of raw or undercooked meat (OR = 1.47; 95% CI: 1.17–1.84), and ethnic minorities (OR = 1.50; 95% CI: 1.29–1.73) were the risk factors of anti-HEV IgG positive rates. Moreover, working years for the occupational population (OR = 1.69; 95% CI: 0.53–5.35) were not statistically significant (Supplementary Figure S39).

### 3.6. Sensitivity analysis

After excluding studies with a JBI score ≤ 5 and sample sizes ≤200, ≤ 300, and ≤ 500, the results of the sensitivity analysis were consistent with the primary results, indicating that the current conclusions are robust (Supplementary Figures S35, S36).

## 4. Discussion

Hepatitis E infection is considered a pending issue in industrialized countries (33), and its burden is largely unknown (34). The systematic review and meta-analysis found that the seroprevalence of anti-HEV IgG, IgM, Ag, and RNA detection rates in China from 1997 to 2022 were 23.17% (95% CI: 20.23–26.25), 0.73% (95% CI: 0.55–0.93), 0.12% (95% CI: 0.01–0.32), and 6.55% (95% CI: 3.46–12.05), respectively. In addition, we estimated the status of hepatitis E infection across provinces of China. The anti-HEV IgG seroprevalence of 37.24% indicates that past hepatitis E infection was most severe in Zhejiang Province, similar to a survey in southern Zhejiang Province (33.28%) (35). Based on the positive rate of anti-HEV IgM antibodies (3.13%), Hubei Province had the highest recent/current infection rate. Besides, Yunnan Province was found to have the most severe situation of ongoing infections based on RNA detection rates (15.91%).

The sensitivity and specificity of hepatitis E assays vary widely (36). Compared to other kits, the prevalence of anti-HEV IgG antibodies detected by Wantai kits has been proven to be higher (23). Similarly, we found that the IgG antibody detection rate of Wantai kits was 1.26 times higher than other commercial kits. Furthermore, the heterogeneity caused by different commercial kits may explain the differences between studies.

China is classified as a highly endemic area, where males seem to be more susceptible to HEV infection (15, 37). In our study, when using the Wantai kits, the pooled results indicated that males had a higher prevalence, while meta-regression analysis revealed that gender did not have statistical significance. This indicates that gender is not a true risk factor, possibly because males are more exposed to susceptible environments. Hepatitis E infection is more common in older adults (38), which is consistent with our study, and we found that the anti-HEV IgG seroprevalence was 1.53 times higher in those aged 50–59 years than in those aged 0–9. In addition, our analysis found that the prevalence of anti-HEV IgG in the occupational population was 1.19 times higher than in the general population, similar to the results of previous studies (39). However, in the analysis of the risk factors, the number of working years of the occupational population was not statistically significant, contrary to the results of a study conducted in Portugal (40). This discrepancy might be due to the small number of available studies.

The prevalence of hepatitis E infection differs between rural and urban areas (41). Our study found a higher prevalence of anti-HEV IgG among the general population in rural China. Additionally, the seroprevalence HEV antibody varies by geographical location (42). Our analysis indicates that the prevalence of anti-HEV IgG in Southern China was 1.10 times higher than that in northern China, consistent with the findings of a recent meta-analysis (43). The pooled results of studies using the Wantai kits revealed that past HEV infection was more severe in coastal regions and western China, and dietary habits may be the trigger. We found that individuals who eat raw or undercooked meat had a 1.47-fold higher risk of hepatitis E than people who do not. HEV has been found in meat products in Japan (44), Thailand (45), and Vietnam (46). Reports have confirmed that consumption of meat products from pigs (47), rabbits (48), and some ruminants (49), such as cows, goats, sheep, and antelopes, is the infectious route of hepatitis E. Hence, it is necessary to thoroughly heat these meat products to ensure complete inactivation of HEV. In addition, we observed that people who used non-piped water had a 1.82-fold risk of developing hepatitis E than those who used tap water. Water-borne outbreaks of hepatitis E have been reported in Uganda (50) and Bangladesh (51). Milk consumption is a novel risk factor for HEV infection (52). HEV has been found in the milk of various mammalian hosts (53), including cows, goats, donkeys, water buffaloes, sheep, and camels, and this finding has been confirmed by a study in Yunnan Province, China (54).

Furthermore, we divided these studies into five groups according to the year of publication or implementation. We found that the highest anti-HEV IgG positivity rates occurred during 2001–2005, possibly because there was no hepatitis E vaccine for prevention and sanitary conditions were relatively poor during this period. Moreover, we found that ethnic minorities are more susceptible to hepatitis E infection, which aligns with the findings of a large multi-ethnic cohort study (27). This finding is probably because individuals from ethnic minorities tend to be distributed in less developed areas (55).

Risk factors are closely related to HEV genotypes, and exposure to polluted water commonly results in GT1 infection. The dominant genotype of hepatitis E in China has changed from GT1 to GT4, which is also confirmed by data from Zhejiang Province in our meta-analysis. To a certain extent, transitions in dominant genotypes shows that the water environment pollution situation in Zhejiang Province has improved. Besides, GT4 was identified in more regions of China; since GT4 is zoonotic, it is essential to be cautious about contact with animal hosts and the consumption of animal products. For GT7, camel meat and milk consumption is a possible trigger for hepatitis E infection (9). Notably, specific genotypes (including GT3, 4, and 7) have been reported to cause chronic HEV infection (usually in immunocompromised populations) and extrahepatic manifestations, such as neurological and kidney damage and contact with animals, consumption of contaminated meat products, and blood transfusions are proven risk factors (56). Thus, immunocompromised populations should pay attention to avoid exposure to these risk factors.

This research had several limitations. First, a comprehensive analysis could not be performed due to the number of studies on RNA detection, genotype, and influencing factors of hepatitis E. Second, there was a high heterogeneity in our analysis as a large number of studies were included, including studies from different economic, cultural, and healthcare contexts. Additionally, there was heterogeneity between commercial or in-house serological tests. Third, since most of the previous studies on hepatitis E antibodies were cross-sectional, there is a lack of prospective studies to strengthen the causal association between risk factors and hepatitis E infection. Finally, the lack of data in some provinces and cities, particularly Taiwan and Macau, limits the generalization of pooled estimates to represent hepatitis E seroprevalence.

## 5. Conclusion

Our study indicated that the prevalence of hepatitis E in China was relatively high (16), and the pooled seroprevalence of anti-HEV IgG in China was 23.17% (95% CI: 20.23–26.25). The anti-HEV IgG seropositivity was higher in the occupational population (48.41%; 95% CI: 40.02–56.85) and individuals aged 50–59 (40.87%; 95% CI: 31.95–50.11). The dominant genotype of hepatitis E in China was GT4, and its risk factors were drinking non-tap water, consumption of raw or undercooked meat, and being an ethnic minority. Thus, preventive measures should be taken in high-risk groups and areas, including increasing hepatitis E screening and vaccination and advocating scientific and reasonable cooking temperature and duration.

## Data availability statement

The original contributions presented in the study are included in the article/Supplementary material, further inquiries can be directed to the corresponding authors.

## Author contributions

SY and ZM designed and supervised this study. KC, MY, and XW conducted literature search, data extraction and analysis, and manuscript writing. CC provided solutions to disagreements and analytical guidance. WZ, DC, YY, YD, XZ, DJ, RY, JQ, and MC revised draft and provided opinions. All authors contributed to the article and approved the submitted version.

## Funding

This study was supported by grants from the National Natural Science Foundation of China (grant numbers: 82173577, 81672005, U1611264, and 81001271) and the Mega-Project of National Science and Technology for the 12 and 13th Five-Year Plan of China (grant numbers: 2018ZX10715-014-002 and 2014ZX10004008).

## Conflict of interest

The authors declare that the research was conducted in the absence of any commercial or financial relationships that could be construed as a potential conflict of interest.

## Publisher’s note

All claims expressed in this article are solely those of the authors and do not necessarily represent those of their affiliated organizations, or those of the publisher, the editors and the reviewers. Any product that may be evaluated in this article, or claim that may be made by its manufacturer, is not guaranteed or endorsed by the publisher.

## Abbreviations

HEV: Hepatitis E virus
OR: Odd ratio
95% CI: 95% Confidential interval
GT: Genotype
JBI: Joanna Briggs Institute
