# Supplementary material for: Prevalence of hepatitis E virus in China from 1997 to 2022: a systematic review and meta-analysis
Source: Front Public Health. 2023 Sep 7;11:1243408. doi: 10.3389/fpubh.2023.1243408 (PMC10512461; doi:10.3389/fpubh.2023.1243408)
Supplement: Supplementary file 1 [file Data_Sheet_1.pdf]

# *Supplementary Material*

# Prevalence of hepatitis E virus in China from 1997 to 2022: a systematic review and meta-analysis

**Kexin Cao1†, BS; Xiaoyue Wu1†, MD; Mengya Yang1†, BS; Can Chen1, PHD; Xiaobao Zhang1, MD; Daixi Jiang1, MD; Yuxia Du1, MD; Mengsha Chen1, BS; Yue You1, BS; Wenkai Zhou1, BS; Jiaxing Qi1, BS; Dingmo Chen1, BS; Rui Yan2, MD; Ziping Miao2*, PhD; Shigui Yang1*, PhD**

[1]Department of Emergency Medicine, Second Affiliated Hospital, Department of Public Health, State Key Laboratory for Diagnosis and Treatment of Infectious Diseases. The key Laboratory of Intelligent Preventive Medicine of Zhejiang Province, Zhejiang University School of Medicine, Hangzhou, China;

[2]Zhejiang Provincial Centre for Disease Control and Prevention, Hangzhou, China; [†]: These authors contributed equally to this work and share first authorship.

*** Correspondence author:**
Correspond to Prof Shigui Yang, PhD, Email: yangshigui@zju.edu.cn; Telephone: 13605705640, Address: 866 Yuhangtang Road, Hangzhou, China.
Correspond to Prof Ziping Miao, Email: zpmiao@cdc.zj.cn; Telephone: 13606505901, Address: 3399 Binsheng Road, Hangzhou, China.

*: These authors contributed equally.

## 1 Supplementary Figures and Tables

### 1.1 Supplementary Figures

| Study | Events | Total | Proportion | 95%-CI | Weight (common) | Weight (random) |
|---|---|---|---|---|---|---|
| Zhang ZX-2003 | 210 | 574 | 0.3659 | [0.3264; 0.4067] | 0.1% | 0.7% |
| AYiGuLi-2010 | 13 | 151 | 0.0861 | [0.0466; 0.1427] | 0.0% | 0.6% |
| Ai X-2009 | 5588 | 12555 | 0.4451 | [0.4364; 0.4538] | 2.1% | 0.7% |
| Bao ZY-2013 | 28 | 180 | 0.1556 | [0.1059; 0.2169] | 0.0% | 0.6% |
| Bi L-2008 | 156 | 1204 | 0.1296 | [0.1111; 0.1499] | 0.2% | 0.7% |
| Bo QN-2018 | 127 | 1019 | 0.1246 | [0.1050; 0.1465] | 0.2% | 0.7% |
| Cai YS-2013 | 230 | 510 | 0.4510 | [0.4072; 0.4953] | 0.1% | 0.7% |
| Cao HJ-2004 | 881 | 2290 | 0.3847 | [0.3647; 0.4050] | 0.4% | 0.7% |
| Cao HJ-2004 | 146 | 189 | 0.7725 | [0.7060; 0.8302] | 0.0% | 0.6% |
| Shen JY-2007 | 749 | 1570 | 0.4771 | [0.4521; 0.5021] | 0.3% | 0.7% |
| Chen XM-2014 | 281 | 868 | 0.3237 | [0.2927; 0.3560] | 0.1% | 0.7% |
| Ning LF-2008 | 1086 | 3561 | 0.3050 | [0.2899; 0.3204] | 0.6% | 0.7% |
| Chen YZ-2006 | 254 | 1084 | 0.2343 | [0.2094; 0.2607] | 0.2% | 0.7% |
| Cheng Y-2007 | 10 | 140 | 0.0714 | [0.0348; 0.1274] | 0.0% | 0.6% |
| Du JY-2014 | 295 | 952 | 0.3099 | [0.2806; 0.3403] | 0.2% | 0.7% |
| Du L-2013 | 7 | 235 | 0.0298 | [0.0121; 0.0604] | 0.0% | 0.6% |
| Fan LZ-2012 | 0 | 158 | 0.0000 | [0.0000; 0.0231] | 0.0% | 0.6% |
| You QZ-2019 | 1113 | 5552 | 0.2005 | [0.1900; 0.2112] | 0.9% | 0.7% |
| Gao DY-2004 | 1804 | 6988 | 0.2582 | [0.2479; 0.2686] | 1.2% | 0.7% |
| Gao XL-2002 | 1 | 182 | 0.0055 | [0.0001; 0.0302] | 0.0% | 0.6% |
| Gong YH-2005 | 134 | 144 | 0.9306 | [0.8760; 0.9662] | 0.0% | 0.6% |
| Gu HY-2013 | 505 | 6258 | 0.0807 | [0.0741; 0.0877] | 1.0% | 0.7% |
| Yao XF-2013 | 806 | 2012 | 0.4006 | [0.3791; 0.4224] | 0.3% | 0.7% |
| He YW-2018 | 15 | 35857 | 0.0004 | [0.0002; 0.0007] | 6.0% | 0.7% |
| Wu Y-2016 | 482 | 2206 | 0.2185 | [0.2014; 0.2363] | 0.4% | 0.7% |
| Wu JY-2016 | 440 | 1720 | 0.2558 | [0.2353; 0.2771] | 0.3% | 0.7% |
| Wu JY-2016 | 317 | 611 | 0.5188 | [0.4784; 0.5591] | 0.1% | 0.7% |
| Hu AQ-2010 | 377 | 2019 | 0.1867 | [0.1699; 0.2044] | 0.3% | 0.7% |
| Zhou X-2015 | 124 | 912 | 0.1360 | [0.1144; 0.1599] | 0.2% | 0.7% |
| Huang GY-2009 | 1013 | 3044 | 0.3328 | [0.3160; 0.3498] | 0.5% | 0.7% |
| Yao MF-2007 | 635 | 1316 | 0.4825 | [0.4552; 0.5099] | 0.2% | 0.7% |
| Huang SY-2020 | 120 | 648 | 0.1852 | [0.1560; 0.2173] | 0.1% | 0.7% |
| Huang SM-2017 | 273 | 5345 | 0.0511 | [0.0453; 0.0573] | 0.9% | 0.7% |
| Huang XY-2012 | 501 | 2250 | 0.2227 | [0.2056; 0.2404] | 0.4% | 0.7% |
| Meng ZH-2005 | 413 | 980 | 0.4214 | [0.3903; 0.4531] | 0.2% | 0.7% |
| Lu B-2008 | 208 | 1060 | 0.1962 | [0.1727; 0.2214] | 0.2% | 0.7% |
| Wang FD-2004 | 394 | 850 | 0.4635 | [0.4296; 0.4977] | 0.1% | 0.7% |
| Zheng YJ-2005 | 573 | 852 | 0.6725 | [0.6399; 0.7040] | 0.1% | 0.7% |
| Zhu JF-2007 | 29 | 176 | 0.1648 | [0.1132; 0.2280] | 0.0% | 0.6% |
| Kong DG-2017 | 825 | 1945 | 0.4242 | [0.4021; 0.4465] | 0.3% | 0.7% |
| Li B-2003 | 14 | 178 | 0.0787 | [0.0437; 0.1284] | 0.0% | 0.6% |
| Li JT-2014 | 197 | 456 | 0.4320 | [0.3860; 0.4789] | 0.1% | 0.7% |
| Bo QN-2019 | 259 | 1700 | 0.1524 | [0.1356; 0.1703] | 0.3% | 0.7% |
| Li L-2012 | 15 | 149 | 0.1007 | [0.0574; 0.1606] | 0.0% | 0.6% |
| Li MY-2008 | 169 | 768 | 0.2201 | [0.1912; 0.2510] | 0.1% | 0.7% |
| Li WJ-2007 | 337 | 1553 | 0.2170 | [0.1967; 0.2383] | 0.3% | 0.7% |
| Li W-2018 | 4923 | 10008 | 0.4919 | [0.4821; 0.5018] | 1.7% | 0.7% |
| Li YB-2004 | 572 | 3336 | 0.1715 | [0.1588; 0.1847] | 0.6% | 0.7% |
| Wang HR-2007 | 1089 | 3931 | 0.2770 | [0.2631; 0.2913] | 0.7% | 0.7% |
| Lin CY-2009 | 403 | 4959 | 0.0813 | [0.0738; 0.0892] | 0.8% | 0.7% |
| Liu JY-2016 | 57 | 2127 | 0.0268 | [0.0204; 0.0346] | 0.4% | 0.7% |
| Liu K-2009 | 223 | 1365 | 0.1634 | [0.1441; 0.1841] | 0.2% | 0.7% |
| Liu XG-2007 | 610 | 1585 | 0.3849 | [0.3608; 0.4093] | 0.3% | 0.7% |
| Liu XG-2008 | 571 | 1351 | 0.4226 | [0.3961; 0.4495] | 0.2% | 0.7% |
| Yu LM-2001 | 54 | 417 | 0.1295 | [0.0988; 0.1656] | 0.1% | 0.7% |
| Lu B-2008 | 373 | 828 | 0.4505 | [0.4162; 0.4851] | 0.1% | 0.7% |
| Lu J-2009 | 259 | 1977 | 0.1310 | [0.1164; 0.1467] | 0.3% | 0.7% |
| Lu YH-2006 | 421 | 663 | 0.6350 | [0.5971; 0.6717] | 0.1% | 0.7% |
| Luo YX-2005 | 215 | 3864 | 0.0556 | [0.0486; 0.0633] | 0.6% | 0.7% |
| Ma TW-2013 | 30 | 324 | 0.0926 | [0.0633; 0.1295] | 0.1% | 0.7% |
| Nong CS-2007 | 172 | 377 | 0.4562 | [0.4051; 0.5080] | 0.1% | 0.7% |
| Pan TJ-2002 | 0 | 1580 | 0.0000 | [0.0000; 0.0023] | 0.3% | 0.7% |
| Pan YL-2021 | 0 | 103794 | 0.0000 | [0.0000; 0.0000] | 17.3% | 0.7% |
| Pu MH-2008 | 408 | 1360 | 0.3000 | [0.2757; 0.3251] | 0.2% | 0.7% |
| Sang LY-2007 | 1107 | 3701 | 0.2991 | [0.2844; 0.3141] | 0.6% | 0.7% |
| Shao HW-2009 | 79 | 830 | 0.0952 | [0.0761; 0.1172] | 0.1% | 0.7% |
| Shao JS-2006 | 2565 | 14020 | 0.1830 | [0.1766; 0.1895] | 2.3% | 0.7% |
| Sun ZH-2017 | 2555 | 47852 | 0.0534 | [0.0514; 0.0554] | 8.0% | 0.7% |
| Sun Z-2014 | 471 | 1483 | 0.3176 | [0.2939; 0.3420] | 0.2% | 0.7% |
| Tang WF-2014 | 139 | 303 | 0.4587 | [0.4016; 0.5167] | 0.1% | 0.7% |
| Tian JS-2007 | 35 | 778 | 0.0450 | [0.0315; 0.0620] | 0.1% | 0.7% |
| Wang DM-2016 | 28 | 1000 | 0.0280 | [0.0187; 0.0402] | 0.2% | 0.7% |
| Wang FY-1999 | 33 | 575 | 0.0574 | [0.0398; 0.0797] | 0.1% | 0.7% |
| Wang L-2013 | 1788 | 4396 | 0.4067 | [0.3922; 0.4214] | 0.7% | 0.7% |
| Wang YC-2005 | 297 | 409 | 0.7262 | [0.6802; 0.7688] | 0.1% | 0.7% |
| Zhu JF-2006 | 72 | 94 | 0.7660 | [0.6674; 0.8471] | 0.0% | 0.6% |
| Wang Q-2014 | 422 | 2812 | 0.1501 | [0.1371; 0.1638] | 0.5% | 0.7% |
| Wang ZZ-2007 | 1105 | 1234 | 0.8955 | [0.8770; 0.9120] | 0.2% | 0.7% |
| Wang RL-2012 | 509 | 2028 | 0.2510 | [0.2322; 0.2705] | 0.3% | 0.7% |
| Wu CH-2003 | 4 | 148 | 0.0270 | [0.0074; 0.0678] | 0.0% | 0.6% |
| Wu XX-2021 | 169 | 836 | 0.2022 | [0.1754; 0.2310] | 0.1% | 0.7% |
| Wu JY-2017 | 310 | 1459 | 0.2125 | [0.1917; 0.2344] | 0.2% | 0.7% |
| Xia XW-2015 | 914 | 3513 | 0.2602 | [0.2457; 0.2750] | 0.6% | 0.7% |
| Xiao ZB-2022 | 1217 | 4661 | 0.2611 | [0.2485; 0.2740] | 0.8% | 0.7% |
| Xie SF-2014 | 1019 | 2614 | 0.3898 | [0.3711; 0.4088] | 0.4% | 0.7% |
| Xing XM-2011 | 185 | 812 | 0.2278 | [0.1994; 0.2583] | 0.1% | 0.7% |
| Xu PN-2014 | 91 | 415 | 0.2193 | [0.1804; 0.2622] | 0.1% | 0.7% |
| Yan GX-2004 | 1269 | 3047 | 0.4165 | [0.3989; 0.4342] | 0.5% | 0.7% |
| Yang F-2012 | 893 | 3771 | 0.2368 | [0.2233; 0.2507] | 0.6% | 0.7% |
| Yang B-2013 | 40 | 597 | 0.0670 | [0.0483; 0.0901] | 0.1% | 0.7% |
| Yang LL-2015 | 966 | 3654 | 0.2644 | [0.2501; 0.2790] | 0.6% | 0.7% |
| Yin YZ-2001 | 63 | 676 | 0.0932 | [0.0724; 0.1177] | 0.1% | 0.7% |

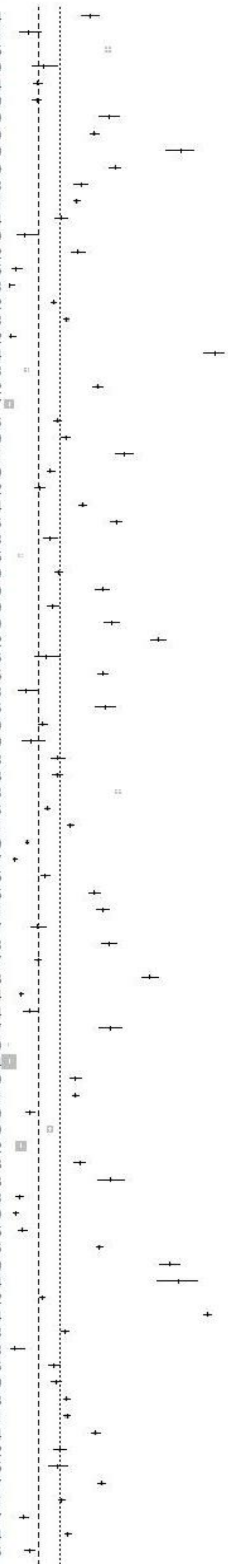

| | | | | | | |
|---|---|---|---|---|---|---|
| Yu DS-2011 | 338 | 2429 | 0.1392 | [0.1256; 0.1536] | 0.4% | 0.7% |
| Yu Q-2022 | 183 | 1076 | 0.1701 | [0.1481; 0.1939] | 0.2% | 0.7% |
| Yu WX-2012 | 138 | 5000 | 0.0276 | [0.0232; 0.0325] | 0.8% | 0.7% |
| Zhang D-2022 | 1000 | 6493 | 0.1540 | [0.1453; 0.1630] | 1.1% | 0.7% |
| Zhang LM-2017 | 95 | 739 | 0.1286 | [0.1053; 0.1548] | 0.1% | 0.7% |
| Zhang P-2015 | 47 | 1195 | 0.0393 | [0.0290; 0.0520] | 0.2% | 0.7% |
| Zhang XF-2007 | 2152 | 4139 | 0.5199 | [0.5046; 0.5353] | 0.7% | 0.7% |
| Zhao HL-2012 | 17 | 327 | 0.0520 | [0.0306; 0.0819] | 0.1% | 0.7% |
| Zheng RD-2013 | 207 | 850 | 0.2435 | [0.2150; 0.2738] | 0.1% | 0.7% |
| Zheng SJ-2015 | 55 | 205 | 0.2683 | [0.2090; 0.3345] | 0.0% | 0.6% |
| Zhong CF-2011 | 1074 | 2627 | 0.4088 | [0.3900; 0.4279] | 0.4% | 0.7% |
| Zhong SQ-2007 | 702 | 1239 | 0.5666 | [0.5385; 0.5944] | 0.2% | 0.7% |
| Zhu GZ-2015 | 690 | 3305 | 0.2088 | [0.1950; 0.2230] | 0.5% | 0.7% |
| Zhu GZ-2007 | 1127 | 4944 | 0.2280 | [0.2163; 0.2399] | 0.8% | 0.7% |
| Zhou HF-2006 | 47 | 175 | 0.2686 | [0.2045; 0.3407] | 0.0% | 0.6% |
| Cai Y-2017 | 366 | 1842 | 0.1987 | [0.1807; 0.2177] | 0.3% | 0.7% |
| Chan DP-2017 | 492 | 1539 | 0.3197 | [0.2964; 0.3436] | 0.3% | 0.7% |
| Chang Y-2009 | 627 | 2819 | 0.2224 | [0.2072; 0.2382] | 0.5% | 0.7% |
| Chen X-2019 | 799 | 4044 | 0.1976 | [0.1854; 0.2102] | 0.7% | 0.7% |
| Chiu DM-2013 | 129 | 450 | 0.2867 | [0.2453; 0.3309] | 0.1% | 0.7% |
| Cong W-2014 | 404 | 1955 | 0.2066 | [0.1889; 0.2253] | 0.3% | 0.7% |
| Cui W-2016 | 228 | 1028 | 0.2218 | [0.1967; 0.2485] | 0.2% | 0.7% |
| Dong C-2012 | 2793 | 14208 | 0.1966 | [0.1901; 0.2032] | 2.4% | 0.7% |
| Feng Y-2018 | 1337 | 1912 | 0.6993 | [0.6782; 0.7198] | 0.3% | 0.7% |
| Fu H-2010 | 70 | 296 | 0.2365 | [0.1892; 0.2891] | 0.0% | 0.7% |
| Fu P-2021 | 249 | 1864 | 0.1336 | [0.1185; 0.1499] | 0.3% | 0.7% |
| Shenyang G-2011 | 37 | 456 | 0.0811 | [0.0578; 0.1101] | 0.1% | 0.7% |
| Geng Y-2019 | 79 | 496 | 0.1593 | [0.1282; 0.1945] | 0.1% | 0.7% |
| Gu G-2015 | 112 | 1491 | 0.0751 | [0.0623; 0.0897] | 0.2% | 0.7% |
| Guo QS-2010 | 14608 | 44816 | 0.3260 | [0.3216; 0.3303] | 7.5% | 0.7% |
| Huang F-2013 | 30 | 293 | 0.1024 | [0.0702; 0.1429] | 0.0% | 0.7% |
| Huang F-2015 | 36 | 388 | 0.0928 | [0.0658; 0.1261] | 0.1% | 0.7% |
| Huang H-2016 | 42 | 391 | 0.1074 | [0.0785; 0.1424] | 0.1% | 0.7% |
| Jia Z-2014 | 3719 | 15852 | 0.2346 | [0.2280; 0.2413] | 2.6% | 0.7% |
| Taniguchi M-2009 | 143 | 300 | 0.4767 | [0.4190; 0.5348] | 0.0% | 0.7% |
| Li H-2021 | 289 | 6269 | 0.0461 | [0.0410; 0.0516] | 1.0% | 0.7% |
| Li MY-2020 | 157 | 946 | 0.1660 | [0.1428; 0.1912] | 0.2% | 0.7% |
| Li RC-2006 | 4839 | 10715 | 0.4516 | [0.4422; 0.4611] | 1.8% | 0.7% |
| Li W-2011 | 69 | 173 | 0.3988 | [0.3253; 0.4759] | 0.0% | 0.6% |
| Liang H-2014 | 129 | 307 | 0.4202 | [0.3644; 0.4776] | 0.1% | 0.7% |
| Liu KSH-2019 | 3408 | 10241 | 0.3328 | [0.3237; 0.3420] | 1.7% | 0.7% |
| Lu J-2009 | 1547 | 8762 | 0.1766 | [0.1686; 0.1847] | 1.5% | 0.7% |
| Ma L-2015 | 87 | 366 | 0.2377 | [0.1950; 0.2847] | 0.1% | 0.7% |
| Ma XX-2021 | 220 | 3568 | 0.0617 | [0.0540; 0.0701] | 0.6% | 0.7% |
| Ma Z-2010 | 407 | 2090 | 0.1947 | [0.1780; 0.2124] | 0.3% | 0.7% |
| Qian Z-2022 | 2275 | 19762 | 0.1151 | [0.1107; 0.1197] | 3.3% | 0.7% |
| Ren F-2013 | 2945 | 10741 | 0.2742 | [0.2658; 0.2827] | 1.8% | 0.7% |
| Rui Z-2018 | 65 | 433 | 0.1501 | [0.1178; 0.1873] | 0.1% | 0.7% |
| Shu Y-2019 | 455 | 1505 | 0.3023 | [0.2792; 0.3262] | 0.3% | 0.7% |
| Tsoi WC-2020 | 315 | 2000 | 0.1575 | [0.1418; 0.1742] | 0.3% | 0.7% |
| Wang M-2017 | 1008 | 4046 | 0.2491 | [0.2359; 0.2628] | 0.7% | 0.7% |
| Wang Y-2018 | 973 | 2592 | 0.3754 | [0.3567; 0.3944] | 0.4% | 0.7% |
| Wen GP-2018 | 1227 | 5345 | 0.2296 | [0.2183; 0.2411] | 0.9% | 0.7% |
| Wong KH-2004 | 176 | 934 | 0.1884 | [0.1638; 0.2150] | 0.2% | 0.7% |
| Xue Y-2013 | 164 | 529 | 0.3100 | [0.2708; 0.3514] | 0.1% | 0.7% |
| Yu Y-2009 | 1299 | 5493 | 0.2365 | [0.2253; 0.2479] | 0.9% | 0.7% |
| Zhang LM-2018 | 1002 | 4102 | 0.2443 | [0.2312; 0.2577] | 0.7% | 0.7% |
| Zhang L-2017 | 236 | 600 | 0.3933 | [0.3540; 0.4337] | 0.1% | 0.7% |
| Zhang W-2009 | 117 | 1476 | 0.0793 | [0.0660; 0.0942] | 0.2% | 0.7% |
| Zhuang W-2014 | 113 | 486 | 0.2325 | [0.1956; 0.2727] | 0.1% | 0.7% |
| **Common effect model** | | **601199** | **0.1336** | **[0.1327; 0.1344]** | **100.0%** | -- |
| **Random effects model** | | | **0.2317** | **[0.2023; 0.2625]** | -- | **100.0%** |
| Heterogeneity: $I^2$ = 100%, $\tau^2$ = 0.0498, $p$ = 0 | | | | | | |

0 0.2 0.4 0.6 0.8

Figure S1. Forest plot of pooled anti-HEV IgG seroprevalence in China

| Study | Events | Total | Proportion | 95%-CI | Weight (common) | Weight (random) |
|---|---|---|---|---|---|---|
| Ao YY-2016 | 26 | 5012 | 0.0052 | [0.0034; 0.0076] | 0.4% | 0.9% |
| Bi L-2008 | 6 | 1204 | 0.0050 | [0.0018; 0.0108] | 0.1% | 0.9% |
| Cao HJ-2004 | 49 | 2290 | 0.0214 | [0.0159; 0.0282] | 0.2% | 0.9% |
| Cao HJ-2004 | 5 | 189 | 0.0265 | [0.0086; 0.0607] | 0.0% | 0.7% |
| Zhang MM-2013 | 3 | 835 | 0.0036 | [0.0007; 0.0105] | 0.1% | 0.8% |
| Chen JP-2014 | 2 | 8697 | 0.0002 | [0.0000; 0.0008] | 0.6% | 0.9% |
| Chen JZ-2014 | 6 | 14275 | 0.0004 | [0.0002; 0.0009] | 1.0% | 0.9% |
| Chen JZ-2015 | 11 | 21612 | 0.0005 | [0.0003; 0.0009] | 1.6% | 0.9% |
| Chen K-2016 | 79 | 10156 | 0.0078 | [0.0062; 0.0097] | 0.7% | 0.9% |
| Jiang RJ-2006 | 17 | 1084 | 0.0157 | [0.0092; 0.0250] | 0.1% | 0.9% |
| Chen WG-2006 | 66 | 8213 | 0.0080 | [0.0062; 0.0102] | 0.6% | 0.9% |
| Xu LL-2016 | 1 | 3225 | 0.0003 | [0.0000; 0.0017] | 0.2% | 0.9% |
| Duan ZJ-2014 | 15 | 10803 | 0.0014 | [0.0008; 0.0023] | 0.8% | 0.9% |
| Fang Y-2003 | 12 | 503 | 0.0239 | [0.0124; 0.0413] | 0.0% | 0.8% |
| You QZ-2019 | 42 | 5552 | 0.0076 | [0.0055; 0.0102] | 0.4% | 0.9% |
| Gao QP-2013 | 20 | 26780 | 0.0007 | [0.0005; 0.0012] | 1.9% | 0.9% |
| Ge SX-2006 | 262 | 17731 | 0.0148 | [0.0131; 0.0167] | 1.3% | 0.9% |
| Gong Q-2012 | 45 | 10220 | 0.0044 | [0.0032; 0.0059] | 0.7% | 0.9% |
| Gu YF-2018 | 227 | 6772 | 0.0335 | [0.0294; 0.0381] | 0.5% | 0.9% |
| Guo MY-2007 | 19 | 26181 | 0.0007 | [0.0004; 0.0011] | 1.9% | 0.9% |
| Guo QL-2011 | 2 | 240 | 0.0083 | [0.0010; 0.0298] | 0.0% | 0.7% |
| Wang XJ-2018 | 5 | 31696 | 0.0002 | [0.0001; 0.0004] | 2.3% | 0.9% |
| Han XX-2010 | 6 | 11362 | 0.0005 | [0.0002; 0.0011] | 0.8% | 0.9% |
| Hu AQ-2010 | 8 | 2019 | 0.0040 | [0.0017; 0.0078] | 0.1% | 0.9% |
| Zhou X-2015 | 13 | 912 | 0.0143 | [0.0076; 0.0243] | 0.1% | 0.8% |
| Huang GY-2009 | 28 | 1516 | 0.0185 | [0.0123; 0.0266] | 0.1% | 0.9% |
| Yao MF-2007 | 35 | 1316 | 0.0266 | [0.0186; 0.0368] | 0.1% | 0.9% |
| Huang XY-2012 | 37 | 2250 | 0.0164 | [0.0116; 0.0226] | 0.2% | 0.9% |

| | | | | | | |
|---|---|---|---|---|---|---|
| Wang FD-2004 | 26 | 850 | 0.0306 | [0.0201; 0.0445] | 0.1% | 0.8% |
| Jiang T-2018 | 26 | 9797 | 0.0027 | [0.0017; 0.0039] | 0.7% | 0.9% |
| Jin LP-2012 | 6 | 11461 | 0.0005 | [0.0002; 0.0011] | 0.8% | 0.9% |
| Kong P-2013 | 22 | 33211 | 0.0007 | [0.0004; 0.0010] | 2.4% | 0.9% |
| Bo QN-2019 | 50 | 1700 | 0.0294 | [0.0219; 0.0386] | 0.1% | 0.9% |
| Li SB-2010 | 4 | 76654 | 0.0001 | [0.0000; 0.0001] | 5.5% | 0.9% |
| Li WJ-2007 | 5 | 1553 | 0.0032 | [0.0010; 0.0075] | 0.1% | 0.9% |
| Li W-2018 | 167 | 10008 | 0.0167 | [0.0143; 0.0194] | 0.7% | 0.9% |
| Li XJ-2019 | 55 | 16022 | 0.0034 | [0.0026; 0.0045] | 1.2% | 0.9% |
| Li YH-2021 | 36 | 25098 | 0.0014 | [0.0010; 0.0020] | 1.8% | 0.9% |
| Li ZR-2005 | 16 | 82670 | 0.0002 | [0.0001; 0.0003] | 6.0% | 0.9% |
| Li TQ-2016 | 37 | 8257 | 0.0045 | [0.0032; 0.0062] | 0.6% | 0.9% |
| Lin Q-2012 | 4 | 5681 | 0.0007 | [0.0002; 0.0018] | 0.4% | 0.9% |
| Liu DX-2019 | 33 | 40182 | 0.0008 | [0.0006; 0.0012] | 2.9% | 0.9% |
| Liu F-2018 | 6 | 9529 | 0.0006 | [0.0002; 0.0014] | 0.7% | 0.9% |
| Liu JY-2016 | 3 | 2127 | 0.0014 | [0.0003; 0.0041] | 0.2% | 0.9% |
| Liu K-2009 | 4 | 1365 | 0.0029 | [0.0008; 0.0075] | 0.1% | 0.9% |
| Liu XG-2007 | 51 | 1585 | 0.0322 | [0.0240; 0.0421] | 0.1% | 0.9% |
| Liu Y-2013 | 115 | 25391 | 0.0045 | [0.0037; 0.0054] | 1.8% | 0.9% |
| Luo Y-2011 | 7 | 31137 | 0.0002 | [0.0001; 0.0005] | 2.2% | 0.9% |
| Ma SB-2014 | 11 | 5639 | 0.0020 | [0.0010; 0.0035] | 0.4% | 0.9% |
| Ma XL-2015 | 12 | 3707 | 0.0032 | [0.0017; 0.0056] | 0.3% | 0.9% |
| Zhang LF-2003 | 19 | 2223 | 0.0085 | [0.0052; 0.0133] | 0.2% | 0.9% |
| Nong HY-2013 | 7 | 11938 | 0.0006 | [0.0002; 0.0012] | 0.9% | 0.9% |
| Qiu SH-2013 | 27 | 27977 | 0.0010 | [0.0006; 0.0014] | 2.0% | 0.9% |
| Sang LY-2007 | 50 | 3701 | 0.0135 | [0.0100; 0.0178] | 0.3% | 0.9% |
| Shao HW-2009 | 4 | 830 | 0.0048 | [0.0013; 0.0123] | 0.1% | 0.8% |
| Sun C-2016 | 214 | 128833 | 0.0017 | [0.0014; 0.0019] | 9.3% | 0.9% |
| Sun JW-2009 | 72 | 5494 | 0.0131 | [0.0103; 0.0165] | 0.4% | 0.9% |
| Sun LP-2004 | 20 | 1043 | 0.0192 | [0.0118; 0.0295] | 0.1% | 0.8% |
| Tan PY-1999 | 124 | 1531 | 0.0810 | [0.0678; 0.0958] | 0.1% | 0.9% |
| Tian GJ-2007 | 13 | 288 | 0.0451 | [0.0243; 0.0760] | 0.0% | 0.7% |
| Wang DM-2016 | 2 | 1000 | 0.0020 | [0.0002; 0.0072] | 0.1% | 0.8% |
| Wang JF-2021 | 29 | 11917 | 0.0024 | [0.0016; 0.0035] | 0.9% | 0.9% |
| Wang LP-2013 | 10 | 25180 | 0.0004 | [0.0002; 0.0007] | 1.8% | 0.9% |
| Wang XH-2018 | 41 | 36152 | 0.0011 | [0.0008; 0.0015] | 2.6% | 0.9% |
| Wu ZT-2013 | 14 | 4441 | 0.0032 | [0.0017; 0.0053] | 0.3% | 0.9% |
| Wu ZH-2017 | 5 | 32120 | 0.0002 | [0.0001; 0.0004] | 2.3% | 0.9% |
| Wu JY-2017 | 7 | 1459 | 0.0048 | [0.0019; 0.0099] | 0.1% | 0.9% |
| Chun X-2012 | 11 | 749 | 0.0147 | [0.0074; 0.0261] | 0.1% | 0.8% |
| Xiao ZY-2016 | 32 | 8952 | 0.0036 | [0.0024; 0.0050] | 0.6% | 0.9% |
| Xie SF-2014 | 144 | 2614 | 0.0551 | [0.0467; 0.0645] | 0.2% | 0.9% |
| Xing Y-2016 | 164 | 95217 | 0.0017 | [0.0015; 0.0020] | 6.9% | 0.9% |
| Xu WL-2018 | 43 | 15897 | 0.0027 | [0.0020; 0.0036] | 1.1% | 0.9% |
| Yan GX-2004 | 46 | 3047 | 0.0151 | [0.0111; 0.0201] | 0.2% | 0.9% |
| Yang LL-2015 | 73 | 3654 | 0.0200 | [0.0157; 0.0251] | 0.3% | 0.9% |
| Yang XY-2016 | 26 | 12271 | 0.0021 | [0.0014; 0.0031] | 0.9% | 0.9% |
| Yu Q-2022 | 10 | 1076 | 0.0093 | [0.0045; 0.0170] | 0.1% | 0.8% |
| Yuan ZZ-2022 | 10 | 1604 | 0.0062 | [0.0030; 0.0114] | 0.1% | 0.9% |
| Zhang LM-2017 | 6 | 739 | 0.0081 | [0.0030; 0.0176] | 0.1% | 0.8% |
| Zhang P-2015 | 3 | 1195 | 0.0025 | [0.0005; 0.0073] | 0.1% | 0.9% |
| Zhang WS-2012 | 3 | 6456 | 0.0005 | [0.0001; 0.0014] | 0.5% | 0.9% |
| Zhang XF-2007 | 103 | 4139 | 0.0249 | [0.0204; 0.0301] | 0.3% | 0.9% |
| Zhao HL-2012 | 8 | 327 | 0.0245 | [0.0106; 0.0476] | 0.0% | 0.7% |
| Zhao JN-2017 | 10 | 10000 | 0.0010 | [0.0005; 0.0018] | 0.7% | 0.9% |
| Zhen RD-2013 | 0 | 850 | 0.0000 | [0.0000; 0.0043] | 0.1% | 0.8% |
| Zhen Y-2015 | 21 | 26583 | 0.0008 | [0.0005; 0.0012] | 1.9% | 0.9% |
| Zhen B-2014 | 124 | 118253 | 0.0010 | [0.0009; 0.0013] | 8.5% | 0.9% |
| Cai Y-2017 | 21 | 1842 | 0.0114 | [0.0071; 0.0174] | 0.1% | 0.9% |
| Chen X-2019 | 43 | 4044 | 0.0106 | [0.0077; 0.0143] | 0.3% | 0.9% |
| Cong W-2014 | 61 | 1955 | 0.0312 | [0.0239; 0.0399] | 0.1% | 0.9% |
| Dong C-2012 | 4 | 14208 | 0.0003 | [0.0001; 0.0007] | 1.0% | 0.9% |
| Feng Y-2018 | 80 | 1912 | 0.0418 | [0.0333; 0.0518] | 0.1% | 0.9% |
| Fu P-2021 | 21 | 1864 | 0.0113 | [0.0070; 0.0172] | 0.1% | 0.9% |
| Geng Y-2019 | 10 | 496 | 0.0202 | [0.0097; 0.0368] | 0.0% | 0.8% |
| Gu G-2015 | 7 | 1491 | 0.0047 | [0.0019; 0.0096] | 0.1% | 0.9% |
| Guo QS-2010 | 420 | 44816 | 0.0094 | [0.0085; 0.0103] | 3.2% | 0.9% |
| Huang F-2013 | 4 | 293 | 0.0137 | [0.0037; 0.0346] | 0.0% | 0.7% |
| Huang F-2015 | 15 | 388 | 0.0387 | [0.0218; 0.0630] | 0.0% | 0.8% |
| Li H-2021 | 11 | 6269 | 0.0018 | [0.0009; 0.0031] | 0.5% | 0.9% |
| Li M-2020 | 42 | 946 | 0.0444 | [0.0322; 0.0595] | 0.1% | 0.8% |
| Li W-2011 | 11 | 173 | 0.0636 | [0.0322; 0.1109] | 0.0% | 0.6% |
| Ma XX-2021 | 21 | 3568 | 0.0059 | [0.0036; 0.0090] | 0.3% | 0.9% |
| Ma Z-2010 | 9 | 2090 | 0.0043 | [0.0020; 0.0082] | 0.2% | 0.9% |
| Qian Z-2022 | 50 | 19762 | 0.0025 | [0.0019; 0.0033] | 1.4% | 0.9% |
| Ren F-2013 | 109 | 10741 | 0.0101 | [0.0083; 0.0122] | 0.8% | 0.9% |
| Rui Z-2018 | 8 | 433 | 0.0185 | [0.0080; 0.0361] | 0.0% | 0.8% |
| Shu Y-2019 | 30 | 1505 | 0.0199 | [0.0135; 0.0283] | 0.1% | 0.9% |
| Tsoi WC-2020 | 16 | 2000 | 0.0080 | [0.0046; 0.0130] | 0.1% | 0.9% |
| Wang M-2017 | 60 | 4046 | 0.0148 | [0.0113; 0.0190] | 0.3% | 0.9% |
| Wang Y-2018 | 16 | 2592 | 0.0062 | [0.0035; 0.0100] | 0.2% | 0.9% |
| Wen GP-2018 | 38 | 5345 | 0.0071 | [0.0050; 0.0097] | 0.4% | 0.9% |
| Xue Y-2013 | 5 | 269 | 0.0186 | [0.0061; 0.0428] | 0.0% | 0.7% |
| Yu Y-2009 | 71 | 4979 | 0.0143 | [0.0112; 0.0180] | 0.4% | 0.9% |
| Zhang L-2018 | 17 | 2048 | 0.0083 | [0.0048; 0.0133] | 0.1% | 0.9% |
| Zhang W-2009 | 29 | 1476 | 0.0196 | [0.0132; 0.0281] | 0.1% | 0.9% |
| Liu XJ-2008 | 11 | 502 | 0.0219 | [0.0110; 0.0389] | 0.0% | 0.8% |
| **Common effect model** | | **1388072** | **0.0017** | **[0.0016; 0.0017]** | **100.0%** | -- |
| **Random effects model** | | | **0.0073** | **[0.0055; 0.0093]** | -- | **100.0%** |

Heterogeneity: $I^2 = 98\%$, $\tau^2 = 0.0033$, $p = 0$

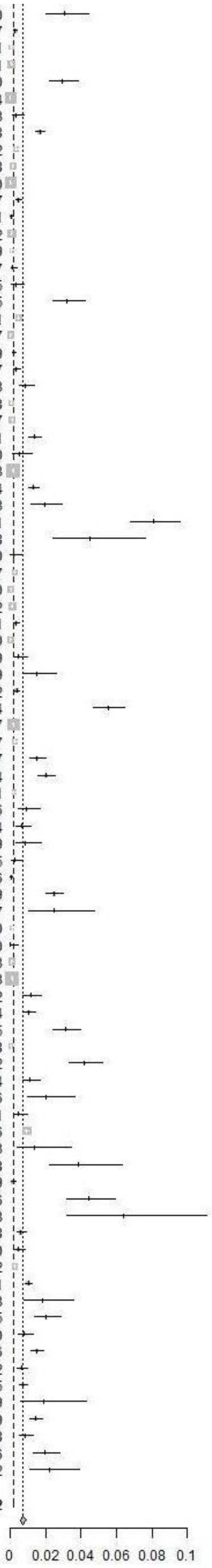

Figure S2. Forest plot of pooled anti-HEV IgM seroprevalence in China

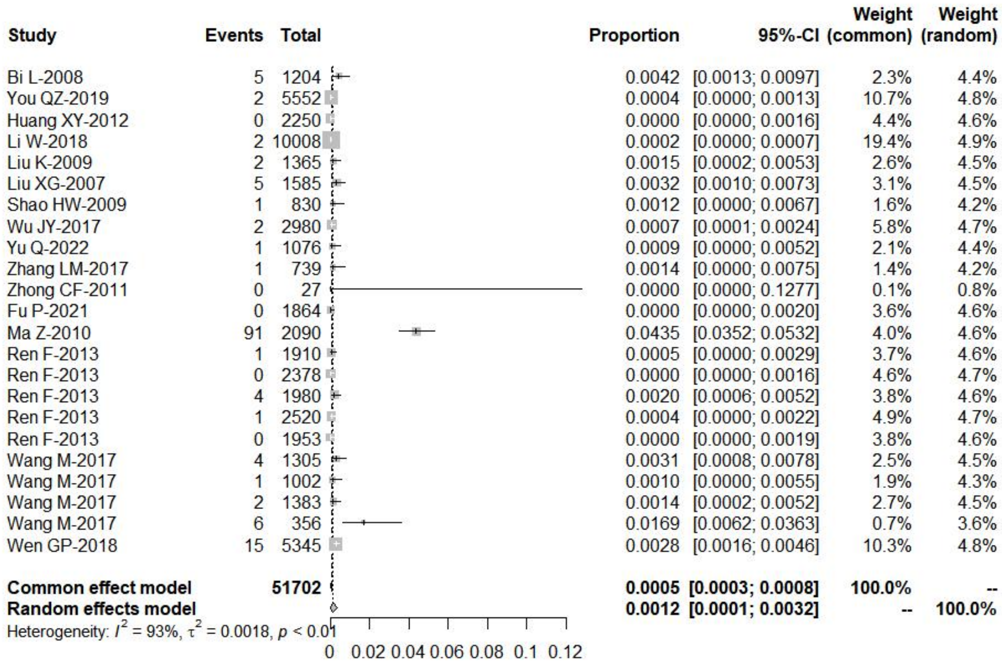


Figure S3. Forest plot of pooled HEV anti-HEV Ag seroprevalence in China

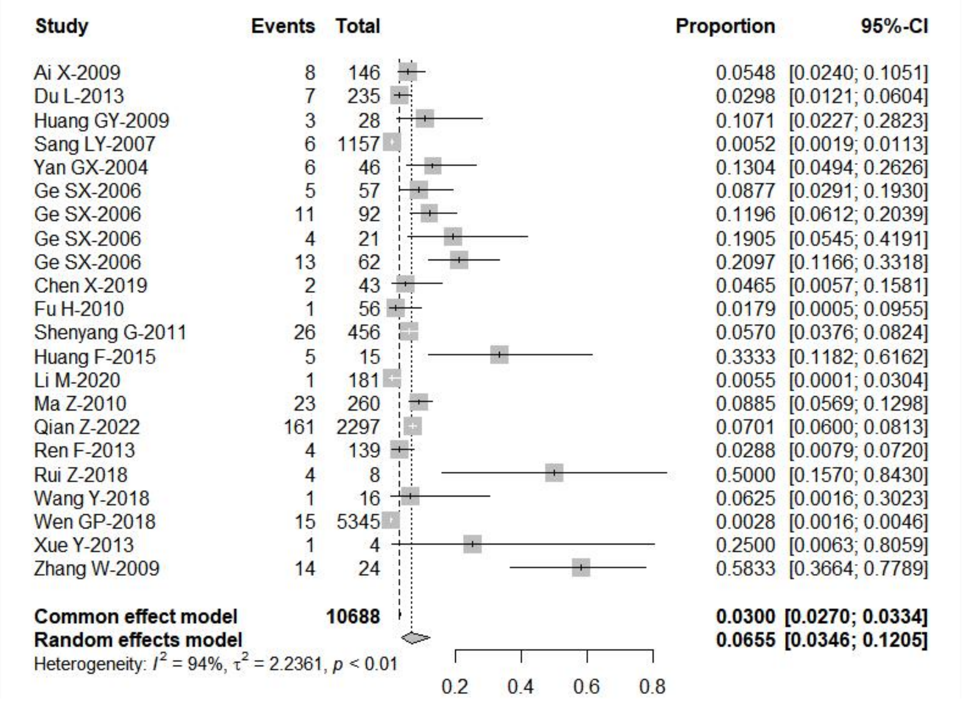


Figure S4. Forest plot of pooled HEV RNA detection rate in China

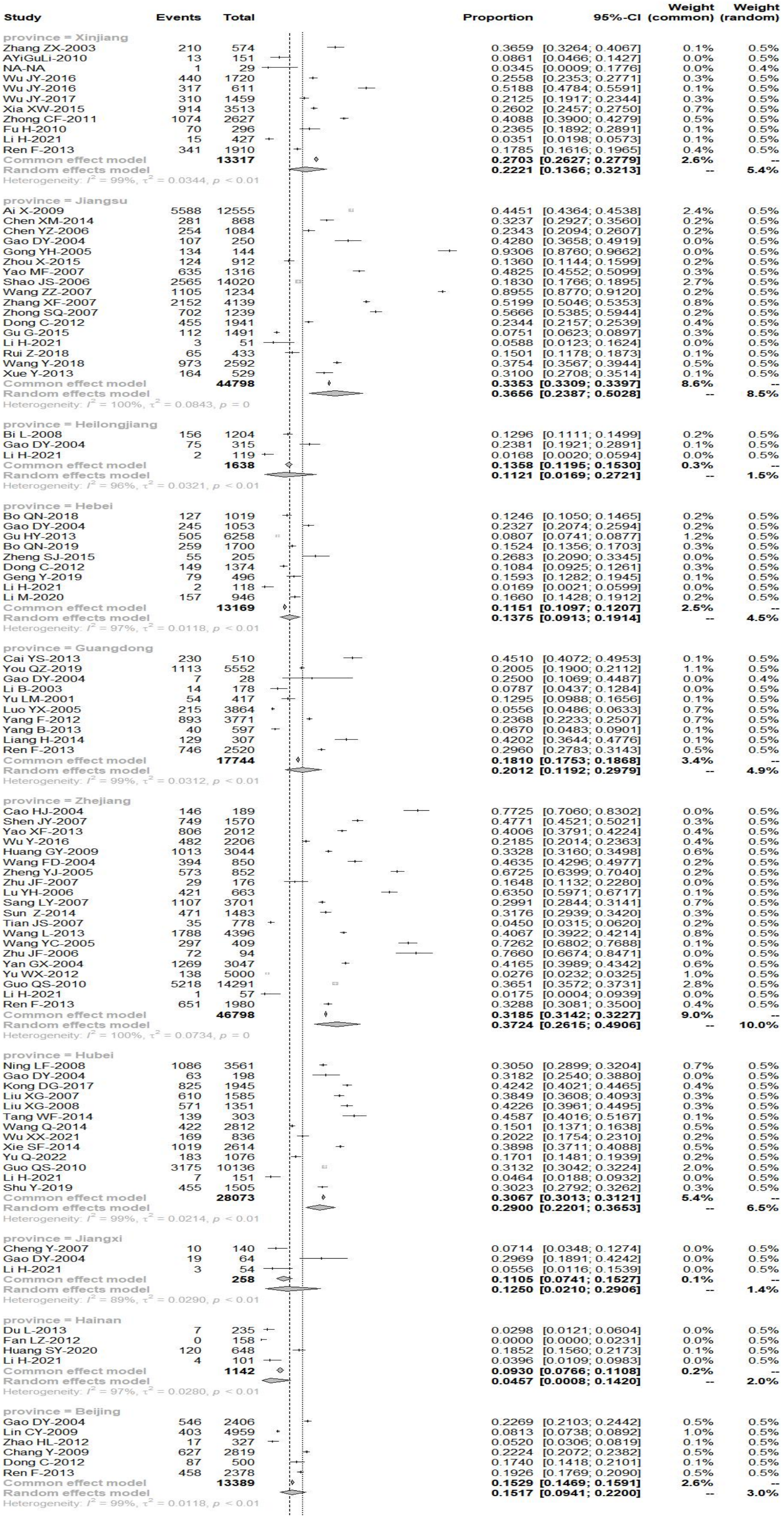

Study Events Total Proportion 95%-CI Weight (common) Weight (random)
province = Xinjiang
Zhang ZX-2003 210 574 0.3659 [0.3264; 0.4067] 0.1% 0.5%
AYiGuLi-2010 13 151 0.0861 [0.0466; 0.1427] 0.0% 0.5%
NA-NA 1 29 0.0345 [0.0009; 0.1776] 0.0% 0.4%
Wu JY-2016 440 1720 0.2558 [0.2353; 0.2771] 0.3% 0.5%
Wu JY-2016 317 611 0.5188 [0.4784; 0.5591] 0.1% 0.5%
Wu JY-2017 310 1459 0.2125 [0.1917; 0.2344] 0.3% 0.5%
Xia XW-2015 914 3513 0.2602 [0.2457; 0.2750] 0.7% 0.5%
Zhong CF-2011 1074 2627 0.4088 [0.3900; 0.4279] 0.5% 0.5%
Fu H-2010 70 296 0.2365 [0.1892; 0.2891] 0.1% 0.5%
Li H-2021 15 427 0.0351 [0.0198; 0.0573] 0.1% 0.5%
Ren F-2013 341 1910 0.1785 [0.1616; 0.1965] 0.4% 0.5%
Common effect model 13317 0.2703 [0.2627; 0.2779] 2.6% --
Random effects model 0.2221 [0.1366; 0.3213] -- 5.4%
Heterogeneity: $I^2$ = 99%, $\tau^2$ = 0.0344, $p$ < 0.01
province = Jiangsu
Ai X-2009 5588 12555 0.4451 [0.4364; 0.4538] 2.4% 0.5%
Chen XM-2014 281 868 0.3237 [0.2927; 0.3560] 0.2% 0.5%
Chen YZ-2006 254 1084 0.2343 [0.2094; 0.2607] 0.2% 0.5%
Gao DY-2004 107 250 0.4280 [0.3658; 0.4919] 0.0% 0.5%
Gong YH-2005 134 144 0.9306 [0.8760; 0.9662] 0.0% 0.5%
Zhou X-2015 124 912 0.1360 [0.1144; 0.1599] 0.2% 0.5%
Yao MF-2007 635 1316 0.4825 [0.4552; 0.5099] 0.3% 0.5%
Shao JS-2006 2565 14020 0.1830 [0.1766; 0.1895] 2.7% 0.5%
Wang ZZ-2007 1105 1234 0.8955 [0.8770; 0.9120] 0.2% 0.5%
Zhang XF-2007 2152 4139 0.5199 [0.5046; 0.5353] 0.8% 0.5%
Zhong SQ-2007 702 1239 0.5666 [0.5385; 0.5944] 0.2% 0.5%
Dong C-2012 455 1941 0.2344 [0.2157; 0.2539] 0.4% 0.5%
Gu G-2015 112 1491 0.0751 [0.0623; 0.0897] 0.3% 0.5%
Li H-2021 3 51 0.0588 [0.0123; 0.1624] 0.0% 0.5%
Rui Z-2018 65 433 0.1501 [0.1178; 0.1873] 0.1% 0.5%
Wang Y-2018 973 2592 0.3754 [0.3567; 0.3944] 0.5% 0.5%
Xue Y-2013 164 529 0.3100 [0.2708; 0.3514] 0.1% 0.5%
Common effect model 44798 0.3353 [0.3309; 0.3397] 8.6% --
Random effects model 0.3656 [0.2387; 0.5028] -- 8.5%
Heterogeneity: $I^2$ = 100%, $\tau^2$ = 0.0843, $p$ = 0
province = Heilongjiang
Bi L-2008 156 1204 0.1296 [0.1111; 0.1499] 0.2% 0.5%
Gao DY-2004 75 315 0.2381 [0.1921; 0.2891] 0.1% 0.5%
Li H-2021 2 119 0.0168 [0.0020; 0.0594] 0.0% 0.5%
Common effect model 1638 0.1358 [0.1195; 0.1530] 0.3% --
Random effects model 0.1121 [0.0169; 0.2721] -- 1.5%
Heterogeneity: $I^2$ = 96%, $\tau^2$ = 0.0321, $p$ < 0.01
province = Hebei
Bo QN-2018 127 1019 0.1246 [0.1050; 0.1465] 0.2% 0.5%
Gao DY-2004 245 1053 0.2327 [0.2074; 0.2594] 0.2% 0.5%
Gu HY-2013 505 6258 0.0807 [0.0741; 0.0877] 1.2% 0.5%
Bo QN-2019 259 1700 0.1524 [0.1356; 0.1703] 0.3% 0.5%
Zheng SJ-2015 55 205 0.2683 [0.2090; 0.3345] 0.0% 0.5%
Dong C-2012 149 1374 0.1084 [0.0925; 0.1261] 0.3% 0.5%
Geng Y-2019 79 496 0.1593 [0.1282; 0.1945] 0.1% 0.5%
Li H-2021 2 118 0.0169 [0.0021; 0.0599] 0.0% 0.5%
Li M-2020 157 946 0.1660 [0.1428; 0.1912] 0.2% 0.5%
Common effect model 13169 0.1151 [0.1097; 0.1207] 2.5% --
Random effects model 0.1375 [0.0913; 0.1914] -- 4.5%
Heterogeneity: $I^2$ = 97%, $\tau^2$ = 0.0118, $p$ < 0.01
province = Guangdong
Cai YS-2013 230 510 0.4510 [0.4072; 0.4953] 0.1% 0.5%
You QZ-2019 1113 5552 0.2005 [0.1900; 0.2112] 1.1% 0.5%
Gao DY-2004 7 28 0.2500 [0.1069; 0.4487] 0.0% 0.4%
Li B-2003 14 178 0.0787 [0.0437; 0.1284] 0.0% 0.5%
Yu LM-2001 54 417 0.1295 [0.0988; 0.1656] 0.1% 0.5%
Luo YX-2005 215 3864 0.0556 [0.0486; 0.0633] 0.7% 0.5%
Yang F-2012 893 3771 0.2368 [0.2233; 0.2507] 0.7% 0.5%
Yang B-2013 40 597 0.0670 [0.0483; 0.0901] 0.1% 0.5%
Liang H-2014 129 307 0.4202 [0.3644; 0.4776] 0.1% 0.5%
Ren F-2013 746 2520 0.2960 [0.2783; 0.3143] 0.5% 0.5%
Common effect model 17744 0.1810 [0.1753; 0.1868] 3.4% --
Random effects model 0.2012 [0.1192; 0.2979] -- 4.9%
Heterogeneity: $I^2$ = 99%, $\tau^2$ = 0.0312, $p$ < 0.01
province = Zhejiang
Cao HJ-2004 146 189 0.7725 [0.7060; 0.8302] 0.0% 0.5%
Shen JY-2007 749 1570 0.4771 [0.4521; 0.5021] 0.3% 0.5%
Yao XF-2013 806 2012 0.4006 [0.3791; 0.4224] 0.4% 0.5%
Wu Y-2016 482 2206 0.2185 [0.2014; 0.2363] 0.4% 0.5%
Huang GY-2009 1013 3044 0.3328 [0.3160; 0.3498] 0.6% 0.5%
Wang FD-2004 394 850 0.4635 [0.4296; 0.4977] 0.2% 0.5%
Zheng YJ-2005 573 852 0.6725 [0.6399; 0.7040] 0.2% 0.5%
Zhu JF-2007 29 176 0.1648 [0.1132; 0.2280] 0.0% 0.5%
Lu YH-2006 421 663 0.6350 [0.5971; 0.6717] 0.1% 0.5%
Sang LY-2007 1107 3701 0.2991 [0.2844; 0.3141] 0.7% 0.5%
Sun Z-2014 471 1483 0.3176 [0.2939; 0.3420] 0.3% 0.5%
Tian JS-2007 35 778 0.0450 [0.0315; 0.0620] 0.2% 0.5%
Wang L-2013 1788 4396 0.4067 [0.3922; 0.4214] 0.8% 0.5%
Wang YC-2005 297 409 0.7262 [0.6802; 0.7688] 0.1% 0.5%
Zhu JF-2006 72 94 0.7660 [0.6674; 0.8471] 0.0% 0.5%
Yan GX-2004 1269 3047 0.4165 [0.3989; 0.4342] 0.6% 0.5%
Yu WX-2012 138 5000 0.0276 [0.0232; 0.0325] 1.0% 0.5%
Guo QS-2010 5218 14291 0.3651 [0.3572; 0.3731] 2.8% 0.5%
Li H-2021 1 57 0.0175 [0.0004; 0.0939] 0.0% 0.5%
Ren F-2013 651 1980 0.3288 [0.3081; 0.3500] 0.4% 0.5%
Common effect model 46798 0.3185 [0.3142; 0.3227] 9.0% --
Random effects model 0.3724 [0.2615; 0.4906] -- 10.0%
Heterogeneity: $I^2$ = 100%, $\tau^2$ = 0.0734, $p$ = 0
province = Hubei
Ning LF-2008 1086 3561 0.3050 [0.2899; 0.3204] 0.7% 0.5%
Gao DY-2004 63 198 0.3182 [0.2540; 0.3880] 0.0% 0.5%
Kong DG-2017 825 1945 0.4242 [0.4021; 0.4465] 0.4% 0.5%
Liu XG-2007 610 1585 0.3849 [0.3608; 0.4093] 0.3% 0.5%
Liu XG-2008 571 1351 0.4226 [0.3961; 0.4495] 0.3% 0.5%
Tang WF-2014 139 303 0.4587 [0.4016; 0.5167] 0.1% 0.5%
Wang Q-2014 422 2812 0.1501 [0.1371; 0.1638] 0.5% 0.5%
Wu XX-2021 169 836 0.2022 [0.1754; 0.2310] 0.2% 0.5%
Xie SF-2014 1019 2614 0.3898 [0.3711; 0.4088] 0.5% 0.5%
Yu Q-2022 183 1076 0.1701 [0.1481; 0.1939] 0.2% 0.5%
Guo QS-2010 3175 10136 0.3132 [0.3042; 0.3224] 2.0% 0.5%
Li H-2021 7 151 0.0464 [0.0188; 0.0932] 0.0% 0.5%
Shu Y-2019 455 1505 0.3023 [0.2792; 0.3262] 0.3% 0.5%
Common effect model 28073 0.3067 [0.3013; 0.3121] 5.4% --
Random effects model 0.2900 [0.2201; 0.3653] -- 6.5%
Heterogeneity: $I^2$ = 99%, $\tau^2$ = 0.0214, $p$ < 0.01
province = Jiangxi
Cheng Y-2007 10 140 0.0714 [0.0348; 0.1274] 0.0% 0.5%
Gao DY-2004 19 64 0.2969 [0.1891; 0.4242] 0.0% 0.5%
Li H-2021 3 54 0.0556 [0.0116; 0.1539] 0.0% 0.5%
Common effect model 258 0.1105 [0.0741; 0.1527] 0.1% --
Random effects model 0.1250 [0.0210; 0.2906] -- 1.4%
Heterogeneity: $I^2$ = 89%, $\tau^2$ = 0.0290, $p$ < 0.01
province = Hainan
Du L-2013 7 235 0.0298 [0.0121; 0.0604] 0.0% 0.5%
Fan LZ-2012 0 158 0.0000 [0.0000; 0.0231] 0.0% 0.5%
Huang SY-2020 120 648 0.1852 [0.1560; 0.2173] 0.1% 0.5%
Li H-2021 4 101 0.0396 [0.0109; 0.0983] 0.0% 0.5%
Common effect model 1142 0.0930 [0.0766; 0.1108] 0.2% --
Random effects model 0.0457 [0.0008; 0.1420] -- 2.0%
Heterogeneity: $I^2$ = 97%, $\tau^2$ = 0.0280, $p$ < 0.01
province = Beijing
Gao DY-2004 546 2406 0.2269 [0.2103; 0.2442] 0.5% 0.5%
Lin CY-2009 403 4959 0.0813 [0.0738; 0.0892] 1.0% 0.5%
Zhao HL-2012 17 327 0.0520 [0.0306; 0.0819] 0.1% 0.5%
Chang Y-2009 627 2819 0.2224 [0.2072; 0.2382] 0.5% 0.5%
Dong C-2012 87 500 0.1740 [0.1418; 0.2101] 0.1% 0.5%
Ren F-2013 458 2378 0.1926 [0.1769; 0.2090] 0.5% 0.5%
Common effect model 13389 0.1529 [0.1469; 0.1591] 2.6% --
Random effects model 0.1517 [0.0941; 0.2200] -- 3.0%
Heterogeneity: $I^2$ = 99%, $\tau^2$ = 0.0118, $p$ < 0.01

| Study | Events | Total | Proportion | 95% CI | Weight (common) | Weight (random) |
|---|---|---|---|---|---|---|
| **province = Sichuan** | | | | | | |
| Gao DY-2004 | 228 | 348 | 0.6552 | [0.6026; 0.7050] | 0.1% | 0.5% |
| Li H-2021 | 26 | 257 | 0.1012 | [0.0672; 0.1447] | 0.0% | 0.5% |
| Common effect model | | **605** | **0.3958** | **[0.3571; 0.4351]** | **0.1%** | -- |
| Random effects model | | | **0.3510** | **[0.0000; 0.8951]** | -- | **1.0%** |
| Heterogeneity: $I^2 = 100\%$, $\tau^2 = 0.1892$, $p < 0.01$ | | | | | | |
| **province = Henan** | | | | | | |
| Gao DY-2004 | 152 | 523 | 0.2906 | [0.2520; 0.3316] | 0.1% | 0.5% |
| Xu PN-2014 | 91 | 415 | 0.2193 | [0.1804; 0.2622] | 0.1% | 0.5% |
| Yang LL-2015 | 966 | 3654 | 0.2644 | [0.2501; 0.2790] | 0.7% | 0.5% |
| Yin YZ-2001 | 63 | 676 | 0.0932 | [0.0724; 0.1177] | 0.1% | 0.5% |
| Dong C-2012 | 376 | 1866 | 0.2015 | [0.1835; 0.2204] | 0.4% | 0.5% |
| Li H-2021 | 2 | 176 | 0.0114 | [0.0014; 0.0404] | 0.0% | 0.5% |
| Common effect model | | **7310** | **0.2200** | **[0.2106; 0.2296]** | **1.4%** | -- |
| Random effects model | | | **0.1637** | **[0.0771; 0.2746]** | -- | **3.0%** |
| Heterogeneity: $I^2 = 98\%$, $\tau^2 = 0.0278$, $p < 0.01$ | | | | | | |
| **province = Shandong** | | | | | | |
| Gao DY-2004 | 84 | 467 | 0.1799 | [0.1461; 0.2178] | 0.1% | 0.5% |
| Liu JY-2016 | 57 | 2127 | 0.0268 | [0.0204; 0.0346] | 0.4% | 0.5% |
| Pan TJ-2002 | 0 | 1580 | 0.0000 | [0.0000; 0.0023] | 0.3% | 0.5% |
| Wang DM-2016 | 28 | 1000 | 0.0280 | [0.0187; 0.0402] | 0.2% | 0.5% |
| Wang FY-1999 | 33 | 575 | 0.0574 | [0.0398; 0.0797] | 0.1% | 0.5% |
| Wang RL-2012 | 509 | 2028 | 0.2510 | [0.2322; 0.2705] | 0.4% | 0.5% |
| Xing XM-2011 | 185 | 812 | 0.2278 | [0.1994; 0.2583] | 0.2% | 0.5% |
| Zhang LM-2017 | 95 | 739 | 0.1286 | [0.1053; 0.1548] | 0.1% | 0.5% |
| Cai Y-2017 | 267 | 1334 | 0.2001 | [0.1790; 0.2226] | 0.3% | 0.5% |
| Cong W-2014 | 404 | 1955 | 0.2066 | [0.1889; 0.2253] | 0.4% | 0.5% |
| Cui W-2016 | 228 | 1028 | 0.2218 | [0.1967; 0.2485] | 0.2% | 0.5% |
| Li H-2021 | 1 | 104 | 0.0096 | [0.0002; 0.0524] | 0.0% | 0.5% |
| Zhang L-2018 | 1002 | 4102 | 0.2443 | [0.2312; 0.2577] | 0.8% | 0.5% |
| Common effect model | | **17851** | **0.1378** | **[0.1328; 0.1430]** | **3.4%** | -- |
| Random effects model | | | **0.1143** | **[0.0601; 0.1830]** | -- | **6.5%** |
| Heterogeneity: $I^2 = 99\%$, $\tau^2 = 0.0313$, $p = 0$ | | | | | | |
| **province = Anhui** | | | | | | |
| Gao DY-2004 | 53 | 210 | 0.2524 | [0.1951; 0.3168] | 0.0% | 0.5% |
| Gao XL-2002 | 1 | 182 | 0.0055 | [0.0001; 0.0302] | 0.0% | 0.5% |
| Hu AQ-2010 | 377 | 2019 | 0.1867 | [0.1699; 0.2044] | 0.4% | 0.5% |
| Dong C-2012 | 205 | 1529 | 0.1341 | [0.1174; 0.1522] | 0.3% | 0.5% |
| Li H-2021 | 2 | 62 | 0.0323 | [0.0039; 0.1117] | 0.0% | 0.5% |
| Common effect model | | **4002** | **0.1524** | **[0.1413; 0.1638]** | **0.8%** | -- |
| Random effects model | | | **0.1036** | **[0.0275; 0.2186]** | -- | **2.5%** |
| Heterogeneity: $I^2 = 97\%$, $\tau^2 = 0.0308$, $p < 0.01$ | | | | | | |
| **province = Inner Mongoria** | | | | | | |
| Gao DY-2004 | 50 | 203 | 0.2463 | [0.1887; 0.3115] | 0.0% | 0.5% |
| Li H-2021 | 2 | 291 | 0.0069 | [0.0008; 0.0246] | 0.1% | 0.5% |
| Ma XX-2021 | 220 | 3568 | 0.0617 | [0.0540; 0.0701] | 0.7% | 0.5% |
| Common effect model | | **4062** | **0.0615** | **[0.0543; 0.0692]** | **0.8%** | -- |
| Random effects model | | | **0.0791** | **[0.0008; 0.2554]** | -- | **1.5%** |
| Heterogeneity: $I^2 = 98\%$, $\tau^2 = 0.0455$, $p < 0.01$ | | | | | | |
| **province = Shanxi** | | | | | | |
| Gao DY-2004 | 29 | 187 | 0.1551 | [0.1064; 0.2151] | 0.0% | 0.5% |
| Dong C-2012 | 43 | 786 | 0.0547 | [0.0399; 0.0730] | 0.2% | 0.5% |
| Li H-2021 | 0 | 51 | 0.0000 | [0.0000; 0.0698] | 0.0% | 0.5% |
| Common effect model | | **1024** | **0.0623** | **[0.0479; 0.0785]** | **0.2%** | -- |
| Random effects model | | | **0.0550** | **[0.0006; 0.1705]** | -- | **1.5%** |
| Heterogeneity: $I^2 = 92\%$, $\tau^2 = 0.0245$, $p < 0.01$ | | | | | | |
| **province = Liaoning** | | | | | | |
| Gao DY-2004 | 33 | 149 | 0.2215 | [0.1576; 0.2967] | 0.0% | 0.5% |
| Huang XY-2012 | 501 | 2250 | 0.2227 | [0.2056; 0.2404] | 0.4% | 0.5% |
| Sun ZH-2017 | 2555 | 47852 | 0.0534 | [0.0514; 0.0554] | 9.2% | 0.5% |
| Shenyang G-2011 | 37 | 456 | 0.0811 | [0.0578; 0.1101] | 0.1% | 0.5% |
| Li H-2021 | 4 | 152 | 0.0263 | [0.0072; 0.0660] | 0.0% | 0.5% |
| Common effect model | | **50859** | **0.0578** | **[0.0557; 0.0599]** | **9.8%** | -- |
| Random effects model | | | **0.1075** | **[0.0408; 0.2001]** | -- | **2.5%** |
| Heterogeneity: $I^2 = 99\%$, $\tau^2 = 0.0209$, $p < 0.01$ | | | | | | |
| **province = Jilin** | | | | | | |
| Gao DY-2004 | 22 | 130 | 0.1692 | [0.1092; 0.2449] | 0.0% | 0.5% |
| Li MY-2008 | 169 | 768 | 0.2201 | [0.1912; 0.2510] | 0.1% | 0.5% |
| Liu K-2009 | 223 | 1365 | 0.1634 | [0.1441; 0.1841] | 0.3% | 0.5% |
| Pu MH-2008 | 408 | 1360 | 0.3000 | [0.2757; 0.3251] | 0.3% | 0.5% |
| Shao HW-2009 | 79 | 830 | 0.0952 | [0.0761; 0.1172] | 0.2% | 0.5% |
| Zhu GZ-2015 | 690 | 3305 | 0.2088 | [0.1950; 0.2230] | 0.6% | 0.5% |
| Zhu GZ-2007 | 1127 | 4944 | 0.2280 | [0.2163; 0.2399] | 1.0% | 0.5% |
| Cai Y-2017 | 99 | 508 | 0.1949 | [0.1613; 0.2320] | 0.1% | 0.5% |
| Dong C-2012 | 127 | 1177 | 0.1079 | [0.0907; 0.1270] | 0.2% | 0.5% |
| Li H-2021 | 1 | 112 | 0.0089 | [0.0002; 0.0487] | 0.0% | 0.5% |
| Common effect model | | **14499** | **0.1991** | **[0.1926; 0.2056]** | **2.8%** | -- |
| Random effects model | | | **0.1611** | **[0.1086; 0.2216]** | -- | **5.0%** |
| Heterogeneity: $I^2 = 97\%$, $\tau^2 = 0.0148$, $p < 0.01$ | | | | | | |
| **province = Shannxi** | | | | | | |
| Gao DY-2004 | 26 | 118 | 0.2203 | [0.1493; 0.3059] | 0.0% | 0.5% |
| Zhang D-2022 | 1000 | 6493 | 0.1540 | [0.1453; 0.1630] | 1.3% | 0.5% |
| Zhang P-2015 | 47 | 1195 | 0.0393 | [0.0290; 0.0520] | 0.2% | 0.5% |
| Dong C-2012 | 237 | 870 | 0.2724 | [0.2431; 0.3033] | 0.2% | 0.5% |
| Li H-2021 | 4 | 118 | 0.0339 | [0.0093; 0.0845] | 0.0% | 0.5% |
| Common effect model | | **8794** | **0.1422** | **[0.1350; 0.1497]** | **1.7%** | -- |
| Random effects model | | | **0.1285** | **[0.0483; 0.2389]** | -- | **2.5%** |
| Heterogeneity: $I^2 = 99\%$, $\tau^2 = 0.0258$, $p < 0.01$ | | | | | | |
| **province = Hunan** | | | | | | |
| Gao DY-2004 | 35 | 106 | 0.3302 | [0.2419; 0.4282] | 0.0% | 0.5% |
| Li WJ-2007 | 337 | 1553 | 0.2170 | [0.1967; 0.2383] | 0.3% | 0.5% |
| Dong C-2012 | 488 | 2181 | 0.2238 | [0.2064; 0.2418] | 0.4% | 0.5% |
| Li H-2021 | 12 | 280 | 0.0429 | [0.0223; 0.0737] | 0.1% | 0.5% |
| Common effect model | | **4120** | **0.2072** | **[0.1949; 0.2198]** | **0.8%** | -- |
| Random effects model | | | **0.1877** | **[0.0780; 0.3305]** | -- | **2.0%** |
| Heterogeneity: $I^2 = 97\%$, $\tau^2 = 0.0270$, $p < 0.01$ | | | | | | |

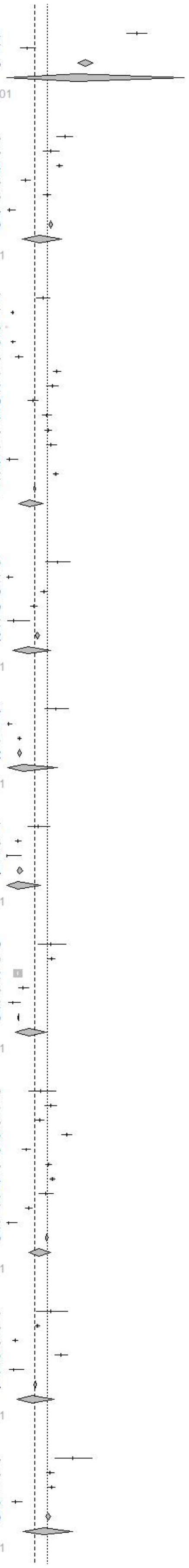

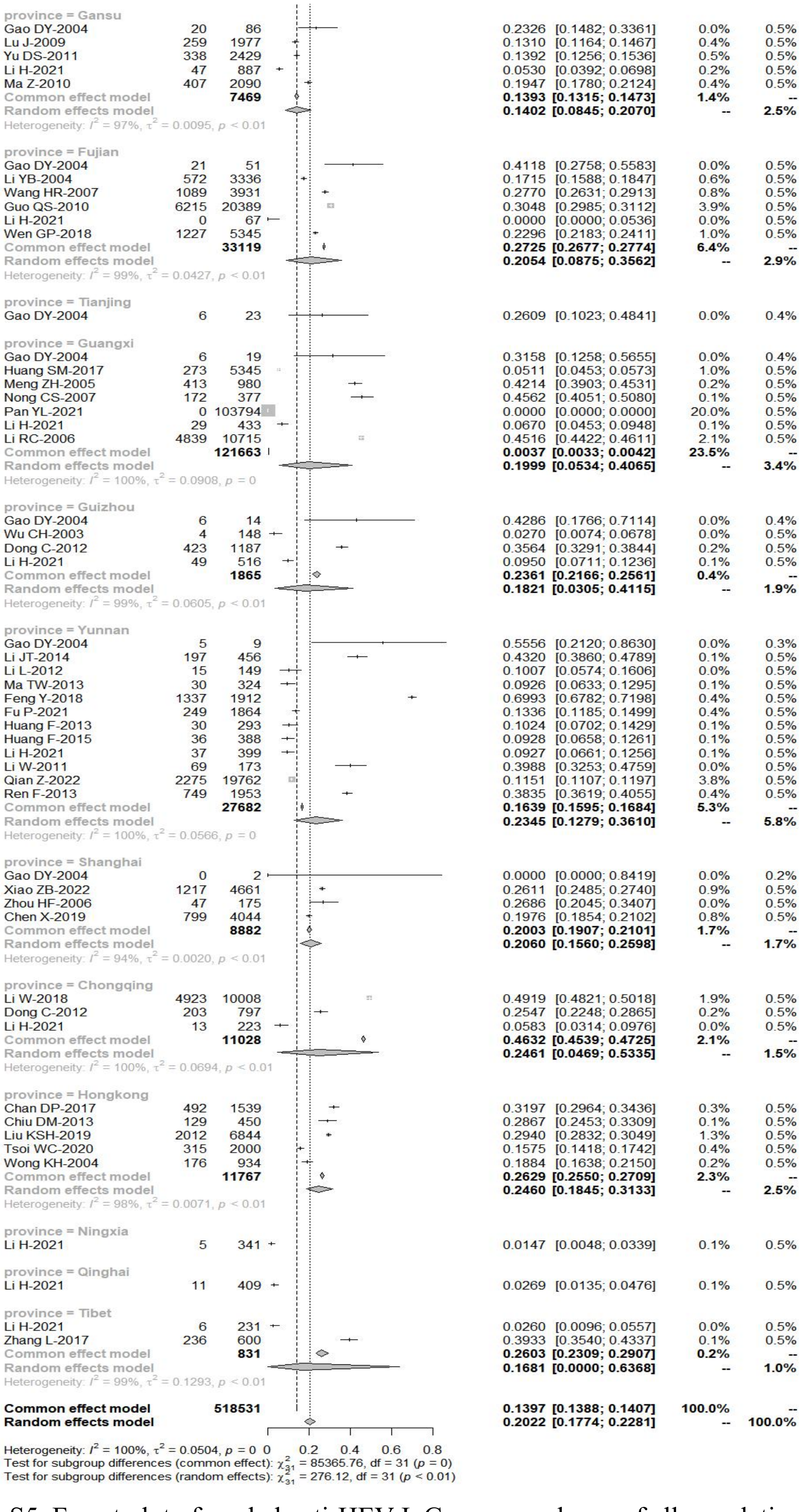


Figure S5. Forest plot of pooled anti-HEV IgG seroprevalence of all populations among different provinces

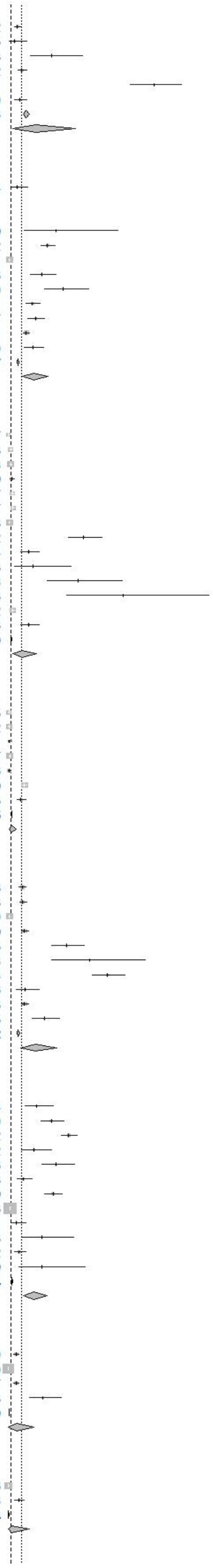

| Study | Events | Total | Proportion | 95%-CI | Weight (common) | Weight (random) |
|---|---|---|---|---|---|---|
| **province = Guangdong** | | | | | | |
| Ao YY-2016 | 26 | 5012 | 0.0052 | [0.0034; 0.0076] | 0.4% | 0.9% |
| Zhang MM-2013 | 3 | 835 | 0.0036 | [0.0007; 0.0105] | 0.1% | 0.9% |
| Fang Y-2003 | 12 | 503 | 0.0239 | [0.0124; 0.0413] | 0.0% | 0.8% |
| You QZ-2019 | 42 | 5552 | 0.0076 | [0.0055; 0.0102] | 0.4% | 0.9% |
| Tan PY-1999 | 124 | 1531 | 0.0810 | [0.0678; 0.0958] | 0.1% | 0.9% |
| Ren F-2013 | 16 | 2520 | 0.0063 | [0.0036; 0.0103] | 0.2% | 0.9% |
| Common effect model | | **15953** | **0.0100** | **[0.0085; 0.0117]** | **1.2%** | **--** |
| Random effects model | | | **0.0152** | **[0.0027; 0.0371]** | **--** | **5.3%** |
| Heterogeneity: $I^2 = 98\%$, $\tau^2 = 0.0074$, $p < 0.01$ | | | | | | |
| **province = Heilongjiang** | | | | | | |
| Bi L-2008 | 6 | 1204 | 0.0050 | [0.0018; 0.0108] | 0.1% | 0.9% |
| **province = Zhejiang** | | | | | | |
| Cao HJ-2004 | 5 | 189 | 0.0265 | [0.0086; 0.0607] | 0.0% | 0.7% |
| Ge SX-2006 | 113 | 5202 | 0.0217 | [0.0179; 0.0261] | 0.4% | 0.9% |
| Guo MY-2007 | 19 | 26181 | 0.0007 | [0.0004; 0.0011] | 1.9% | 0.9% |
| Huang GY-2009 | 28 | 1516 | 0.0185 | [0.0123; 0.0266] | 0.1% | 0.9% |
| Wang FD-2004 | 26 | 850 | 0.0306 | [0.0201; 0.0445] | 0.1% | 0.9% |
| Sang LY-2007 | 50 | 3701 | 0.0135 | [0.0100; 0.0178] | 0.3% | 0.9% |
| Yan GX-2004 | 46 | 3047 | 0.0151 | [0.0111; 0.0201] | 0.2% | 0.9% |
| Guo QS-2010 | 142 | 14291 | 0.0099 | [0.0084; 0.0117] | 1.1% | 0.9% |
| Ren F-2013 | 27 | 1980 | 0.0136 | [0.0090; 0.0198] | 0.1% | 0.9% |
| Common effect model | | **56957** | **0.0054** | **[0.0048; 0.0060]** | **4.2%** | **--** |
| Random effects model | | | **0.0142** | **[0.0080; 0.0220]** | **--** | **7.9%** |
| Heterogeneity: $I^2 = 98\%$, $\tau^2 = 0.0018$, $p < 0.01$ | | | | | | |
| **province = Yunnan** | | | | | | |
| Chen JP-2014 | 2 | 8697 | 0.0002 | [0.0000; 0.0008] | 0.6% | 0.9% |
| Duan ZJ-2014 | 15 | 10803 | 0.0014 | [0.0008; 0.0023] | 0.8% | 0.9% |
| Li YH-2021 | 36 | 25098 | 0.0014 | [0.0010; 0.0020] | 1.9% | 0.9% |
| Ma SB-2014 | 11 | 5639 | 0.0020 | [0.0010; 0.0035] | 0.4% | 0.9% |
| Wang JF-2021 | 29 | 11917 | 0.0024 | [0.0016; 0.0035] | 0.9% | 0.9% |
| Xu WL-2018 | 43 | 15897 | 0.0027 | [0.0020; 0.0036] | 1.2% | 0.9% |
| Zheng Y-2015 | 21 | 26583 | 0.0008 | [0.0005; 0.0012] | 2.0% | 0.9% |
| Feng Y-2018 | 80 | 1912 | 0.0418 | [0.0333; 0.0518] | 0.1% | 0.9% |
| Fu P-2021 | 21 | 1864 | 0.0113 | [0.0070; 0.0172] | 0.1% | 0.9% |
| Huang F-2013 | 4 | 293 | 0.0137 | [0.0037; 0.0346] | 0.0% | 0.7% |
| Huang F-2015 | 15 | 388 | 0.0387 | [0.0218; 0.0630] | 0.0% | 0.8% |
| Li W-2011 | 11 | 173 | 0.0636 | [0.0322; 0.1109] | 0.0% | 0.6% |
| Qian Z-2022 | 50 | 19762 | 0.0025 | [0.0019; 0.0033] | 1.5% | 0.9% |
| Ren F-2013 | 22 | 1953 | 0.0113 | [0.0071; 0.0170] | 0.1% | 0.9% |
| Common effect model | | **130979** | **0.0016** | **[0.0013; 0.0018]** | **9.8%** | **--** |
| Random effects model | | | **0.0077** | **[0.0023; 0.0159]** | **--** | **12.3%** |
| Heterogeneity: $I^2 = 97\%$, $\tau^2 = 0.0050$, $p < 0.01$ | | | | | | |
| **province = Fujian** | | | | | | |
| Chen JZ-2014 | 6 | 14275 | 0.0004 | [0.0002; 0.0009] | 1.1% | 0.9% |
| Chen JZ-2014 | 11 | 21612 | 0.0005 | [0.0003; 0.0009] | 1.6% | 0.9% |
| Lin Q-2012 | 4 | 5681 | 0.0007 | [0.0002; 0.0018] | 0.4% | 0.9% |
| Qiu SH-2013 | 27 | 27977 | 0.0010 | [0.0006; 0.0014] | 2.1% | 0.9% |
| Zhang WS-2012 | 3 | 6456 | 0.0005 | [0.0001; 0.0014] | 0.5% | 0.9% |
| Guo QS-2010 | 186 | 20389 | 0.0091 | [0.0079; 0.0105] | 1.5% | 0.9% |
| Wen GP-2018 | 38 | 5345 | 0.0071 | [0.0050; 0.0097] | 0.4% | 0.9% |
| Common effect model | | **101735** | **0.0018** | **[0.0016; 0.0021]** | **7.6%** | **--** |
| Random effects model | | | **0.0019** | **[0.0004; 0.0045]** | **--** | **6.5%** |
| Heterogeneity: $I^2 = 98\%$, $\tau^2 = 0.0010$, $p < 0.01$ | | | | | | |
| **province = Hubei** | | | | | | |
| Chen K-2106 | 79 | 10156 | 0.0078 | [0.0062; 0.0097] | 0.8% | 0.9% |
| Cheng WG-2006 | 66 | 8213 | 0.0080 | [0.0062; 0.0102] | 0.6% | 0.9% |
| Gao YP-2013 | 20 | 26780 | 0.0007 | [0.0005; 0.0012] | 2.0% | 0.9% |
| Ge SX-2006 | 92 | 10139 | 0.0091 | [0.0073; 0.0111] | 0.8% | 0.9% |
| Liu XG-2007 | 51 | 1585 | 0.0322 | [0.0240; 0.0421] | 0.1% | 0.9% |
| Tian GJ-2007 | 13 | 288 | 0.0451 | [0.0243; 0.0760] | 0.0% | 0.7% |
| Xie SF-2014 | 144 | 2614 | 0.0551 | [0.0467; 0.0645] | 0.2% | 0.9% |
| Yu Q-2022 | 10 | 1076 | 0.0093 | [0.0045; 0.0170] | 0.1% | 0.9% |
| Guo QS-2010 | 92 | 10136 | 0.0091 | [0.0073; 0.0111] | 0.8% | 0.9% |
| Shu Y-2019 | 30 | 1505 | 0.0199 | [0.0135; 0.0283] | 0.1% | 0.9% |
| Common effect model | | **72492** | **0.0057** | **[0.0051; 0.0062]** | **5.4%** | **--** |
| Random effects model | | | **0.0154** | **[0.0070; 0.0270]** | **--** | **8.9%** |
| Heterogeneity: $I^2 = 99\%$, $\tau^2 = 0.0041$, $p < 0.01$ | | | | | | |
| **province = Jiangsu** | | | | | | |
| Chen YZ-2006 | 17 | 1084 | 0.0157 | [0.0092; 0.0250] | 0.1% | 0.9% |
| Ge SX-2006 | 57 | 2390 | 0.0238 | [0.0181; 0.0308] | 0.2% | 0.9% |
| Gu YF-2018 | 227 | 6772 | 0.0335 | [0.0294; 0.0381] | 0.5% | 0.9% |
| Zhou X-2015 | 13 | 912 | 0.0143 | [0.0076; 0.0243] | 0.1% | 0.9% |
| Yao MF-2007 | 35 | 1316 | 0.0266 | [0.0186; 0.0368] | 0.1% | 0.9% |
| Zhang LF-2003 | 19 | 2223 | 0.0085 | [0.0052; 0.0133] | 0.2% | 0.9% |
| Zhang XF-2007 | 103 | 4139 | 0.0249 | [0.0204; 0.0301] | 0.3% | 0.9% |
| Zheng Y-2014 | 124 | 118253 | 0.0010 | [0.0009; 0.0013] | 8.8% | 0.9% |
| Gu G-2015 | 7 | 1491 | 0.0047 | [0.0019; 0.0096] | 0.1% | 0.9% |
| Rui Z-2018 | 8 | 433 | 0.0185 | [0.0080; 0.0361] | 0.0% | 0.8% |
| Wang Y-2018 | 16 | 2592 | 0.0062 | [0.0035; 0.0100] | 0.2% | 0.9% |
| Xue Y-2013 | 5 | 269 | 0.0186 | [0.0061; 0.0428] | 0.0% | 0.7% |
| Common effect model | | **141874** | **0.0021** | **[0.0019; 0.0024]** | **10.6%** | **--** |
| Random effects model | | | **0.0143** | **[0.0085; 0.0215]** | **--** | **10.5%** |
| Heterogeneity: $I^2 = 99\%$, $\tau^2 = 0.0021$, $p < 0.01$ | | | | | | |
| **province = Shanxi** | | | | | | |
| Gong Q-2012 | 45 | 10220 | 0.0044 | [0.0032; 0.0059] | 0.8% | 0.9% |
| Li ZR-2005 | 16 | 82670 | 0.0002 | [0.0001; 0.0003] | 6.2% | 0.9% |
| Li TQ-2016 | 37 | 8257 | 0.0045 | [0.0032; 0.0062] | 0.6% | 0.9% |
| Sun LP-2004 | 20 | 1043 | 0.0192 | [0.0118; 0.0295] | 0.1% | 0.9% |
| Common effect model | | **102190** | **0.0005** | **[0.0003; 0.0006]** | **7.6%** | **--** |
| Random effects model | | | **0.0048** | **[0.0003; 0.0143]** | **--** | **3.6%** |
| Heterogeneity: $I^2 = 99\%$, $\tau^2 = 0.0025$, $p < 0.01$ | | | | | | |
| **province = Inner Mongolia** | | | | | | |
| Wang XJ-2018 | 5 | 31696 | 0.0002 | [0.0001; 0.0004] | 2.4% | 0.9% |
| Ma XX-2021 | 21 | 3568 | 0.0059 | [0.0036; 0.0090] | 0.3% | 0.9% |
| Common effect model | | **35264** | **0.0003** | **[0.0001; 0.0005]** | **2.6%** | **--** |
| Random effects model | | | **0.0019** | **[0.0000; 0.0116]** | **--** | **1.8%** |
| Heterogeneity: $I^2 = 98\%$, $\tau^2 = 0.0020$, $p < 0.01$ | | | | | | |

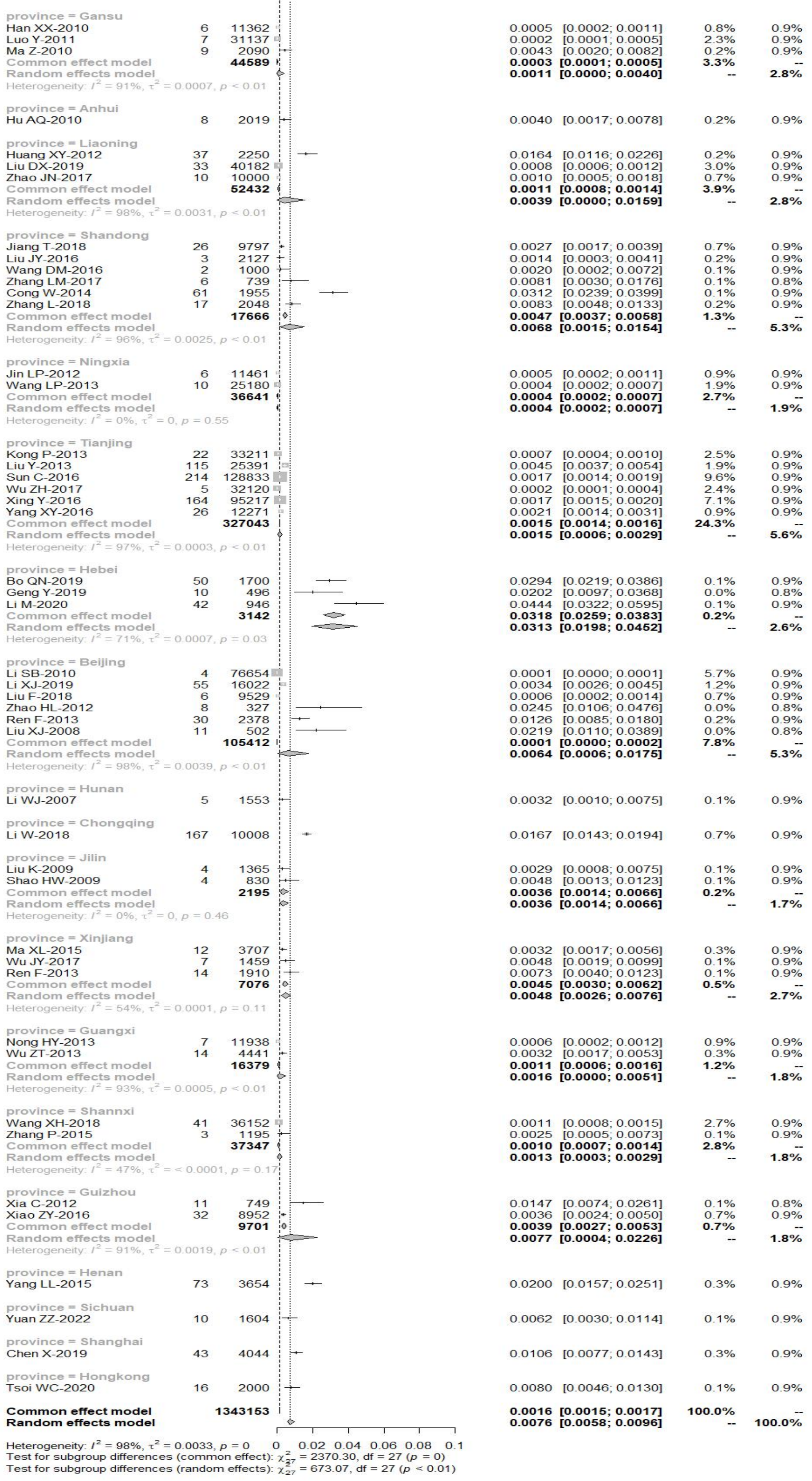


Figure S6. Forest plot of pooled anti-HEV IgM seroprevalence of all populations among different provinces

| Study | Events | Total | Proportion | 95%-CI | Weight (common) | Weight (random) |
|---|---|---|---|---|---|---|
| province = Heilongjiang | | | | | | |
| Bi L-2008 | 5 | 1204 | 0.0042 | [0.0013; 0.0097] | 2.3% | 4.4% |
| province = Guangdong | | | | | | |
| You QZ-2019 | 2 | 5552 | 0.0004 | [0.0000; 0.0013] | 10.7% | 4.8% |
| Ren F-2013 | 1 | 2520 | 0.0004 | [0.0000; 0.0022] | 4.9% | 4.7% |
| Common effect model | | **8072** | **0.0004** | **[0.0000; 0.0009]** | **15.6%** | -- |
| Random effects model | | | **0.0004** | **[0.0000; 0.0009]** | -- | **9.5%** |
| Heterogeneity: $I^2 = 0\%$, $\tau^2 = 0$, $p = 0.81$ | | | | | | |
| province = Liaoning | | | | | | |
| Huang XY-2012 | 0 | 2250 | 0.0000 | [0.0000; 0.0016] | 4.4% | 4.6% |
| province = Chongqing | | | | | | |
| Li W-2018 | 2 | 10008 | 0.0002 | [0.0000; 0.0007] | 19.4% | 4.9% |
| province = Jilin | | | | | | |
| Liu K-2009 | 2 | 1365 | 0.0015 | [0.0002; 0.0053] | 2.6% | 4.5% |
| Shao HW-2009 | 1 | 830 | 0.0012 | [0.0000; 0.0067] | 1.6% | 4.2% |
| Common effect model | | **2195** | **0.0013** | **[0.0001; 0.0035]** | **4.2%** | -- |
| Random effects model | | | **0.0013** | **[0.0001; 0.0035]** | -- | **8.7%** |
| Heterogeneity: $I^2 = 0\%$, $\tau^2 = 0$, $p = 0.97$ | | | | | | |
| province = Hubei | | | | | | |
| Liu XG-2007 | 5 | 1585 | 0.0032 | [0.0010; 0.0073] | 3.1% | 4.5% |
| Yu Q-2022 | 1 | 1076 | 0.0009 | [0.0000; 0.0052] | 2.1% | 4.4% |
| Common effect model | | **2661** | **0.0021** | **[0.0006; 0.0044]** | **5.1%** | -- |
| Random effects model | | | **0.0021** | **[0.0005; 0.0046]** | -- | **8.9%** |
| Heterogeneity: $I^2 = 20\%$, $\tau^2 = < 0.0001$, $p = 0.26$ | | | | | | |
| province = Xinjiang | | | | | | |
| Wu JY-2017 | 2 | 2980 | 0.0007 | [0.0001; 0.0024] | 5.8% | 4.7% |
| Zhong CF-2011 | 0 | 27 | 0.0000 | [0.0000; 0.1277] | 0.1% | 0.8% |
| Ren F-2013 | 1 | 1910 | 0.0005 | [0.0000; 0.0029] | 3.7% | 4.6% |
| Wang M-2017 | 1 | 1002 | 0.0010 | [0.0000; 0.0055] | 1.9% | 4.3% |
| Common effect model | | **5919** | **0.0000** | **[0.0000; 0.0000]** | **11.4%** | -- |
| Random effects model | | | **0.0000** | **[0.0000; 0.0000]** | -- | **14.5%** |
| Heterogeneity: $I^2 = 0\%$, $\tau^2 = 0$, $p = 0.85$ | | | | | | |
| province = Shandong | | | | | | |
| Zhang LM-2017 | 1 | 739 | 0.0014 | [0.0000; 0.0075] | 1.4% | 4.2% |
| province = Yunnan | | | | | | |
| Fu P-2021 | 0 | 1864 | 0.0000 | [0.0000; 0.0020] | 3.6% | 4.6% |
| Ren F-2013 | 0 | 1953 | 0.0000 | [0.0000; 0.0019] | 3.8% | 4.6% |
| Wang M-2017 | 4 | 1305 | 0.0031 | [0.0008; 0.0078] | 2.5% | 4.5% |
| Common effect model | | **5122** | **0.0003** | **[0.0000; 0.0011]** | **9.9%** | -- |
| Random effects model | | | **0.0004** | **[0.0000; 0.0029]** | -- | **13.6%** |
| Heterogeneity: $I^2 = 77\%$, $\tau^2 = 0.0006$, $p = 0.01$ | | | | | | |
| province = Gansu | | | | | | |
| Ma Z-2010 | 91 | 2090 | 0.0435 | [0.0352; 0.0532] | 4.0% | 4.6% |
| province = Beijing | | | | | | |
| Ren F-2013 | 0 | 2378 | 0.0000 | [0.0000; 0.0016] | 4.6% | 4.7% |
| province = Zhejiang | | | | | | |
| Ren F-2013 | 4 | 1980 | 0.0020 | [0.0006; 0.0052] | 3.8% | 4.6% |
| province = Guangxi | | | | | | |
| Wang M-2017 | 2 | 1383 | 0.0014 | [0.0002; 0.0052] | 2.7% | 4.5% |
| province = Henan | | | | | | |
| Wang M-2017 | 6 | 356 | 0.0169 | [0.0062; 0.0363] | 0.7% | 3.6% |
| province = Fujian | | | | | | |
| Wen GP-2018 | 15 | 5345 | 0.0028 | [0.0016; 0.0046] | 10.3% | 4.8% |
| **Common effect model** | | **51702** | **0.0005** | **[0.0003; 0.0008]** | **100.0%** | -- |
| **Random effects model** | | | **0.0012** | **[0.0001; 0.0032]** | -- | **100.0%** |

Axis: 0 0.02 0.04 0.06 0.08 0.1 0.12

Heterogeneity: $I^2 = 93\%$, $\tau^2 = 0.0018$, $p < 0.01$
Test for subgroup differences (common effect): $\chi^2_{14} = 323.16$, df = 14 ($p < 0.01$)
Test for subgroup differences (random effects): $\chi^2_{14} = 318.88$, df = 14 ($p < 0.01$)

Figure S7. Forest plot of pooled anti-HEV Ag seroprevalence of all populations among different provinces

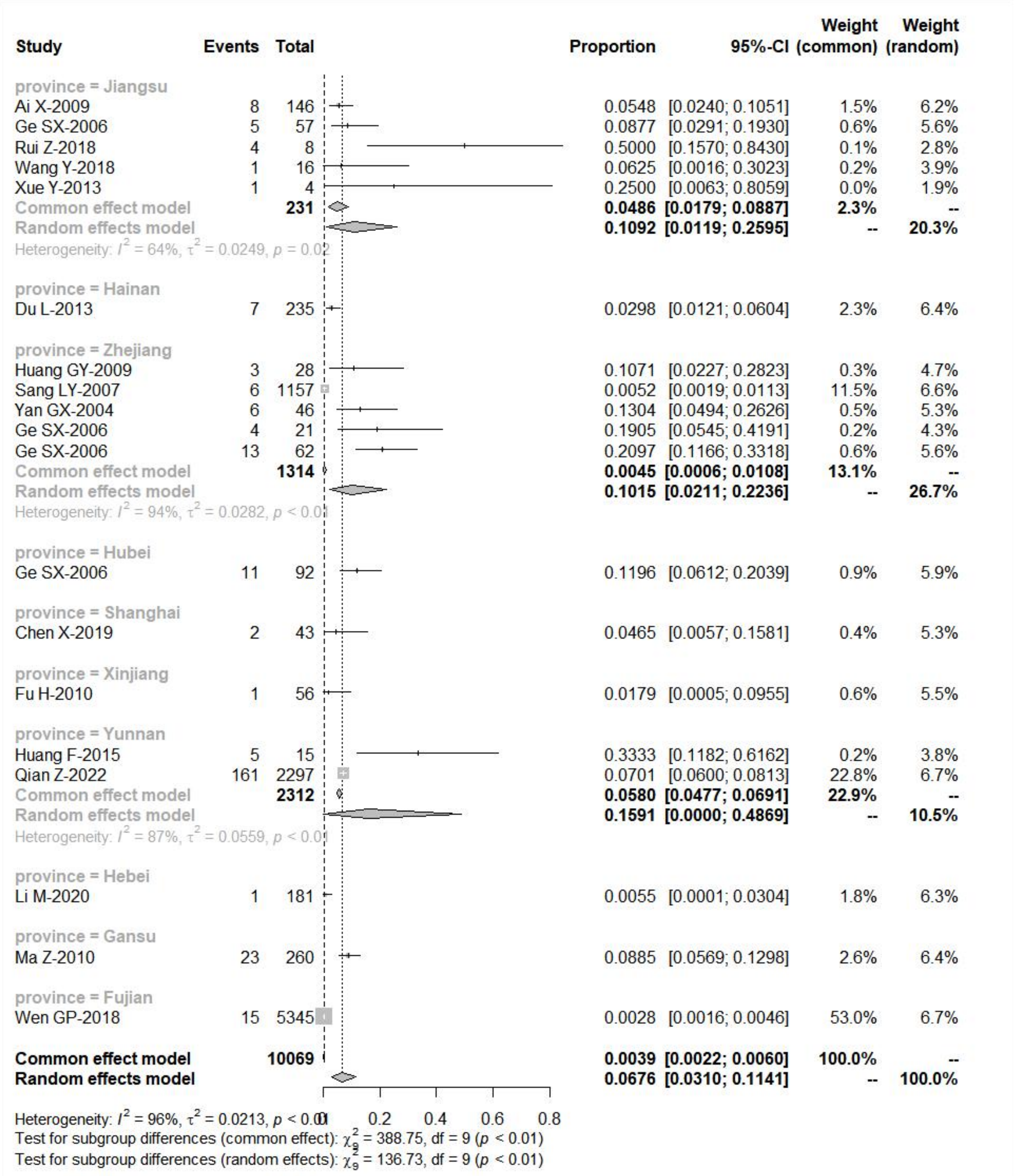


Figure S8. Forest plot of pooled HEV RNA detection rate of all populations in different provinces

| Study | Events | Total | Proportion | 95%-CI | Weight (common) | Weight (random) |
|---|---|---|---|---|---|---|
| **Population = The General Population** | | | | | | |
| Zhang ZX-2003 | 210 | 574 | 0.3659 | [0.3264; 0.4067] | 0.1% | 0.5% |
| AYiGuLi-2010 | 13 | 151 | 0.0861 | [0.0466; 0.1427] | 0.0% | 0.5% |
| Ai X-2009 | 5588 | 12555 | 0.4451 | [0.4364; 0.4538] | 2.1% | 0.6% |
| Bao ZY-2013 | 28 | 180 | 0.1556 | [0.1059; 0.2169] | 0.0% | 0.5% |
| Bi L-2008 | 107 | 1001 | 0.1069 | [0.0884; 0.1277] | 0.2% | 0.5% |
| Cao HJ-2004 | 648 | 1934 | 0.3351 | [0.3140; 0.3566] | 0.3% | 0.6% |
| Shen JY-2007 | 749 | 1570 | 0.4771 | [0.4521; 0.5021] | 0.3% | 0.5% |
| Chen XM-2014 | 281 | 868 | 0.3237 | [0.2927; 0.3560] | 0.1% | 0.5% |
| Ning LF-2008 | 1086 | 3561 | 0.3050 | [0.2899; 0.3204] | 0.6% | 0.6% |
| Chen YZ-2006 | 254 | 1084 | 0.2343 | [0.2094; 0.2607] | 0.2% | 0.5% |
| Cheng Y-2007 | 10 | 140 | 0.0714 | [0.0348; 0.1274] | 0.0% | 0.5% |
| Du JY-2014 | 295 | 952 | 0.3099 | [0.2806; 0.3403] | 0.2% | 0.5% |
| Du L-2013 | 7 | 235 | 0.0298 | [0.0121; 0.0604] | 0.0% | 0.5% |
| Fan LZ-2012 | 0 | 158 | 0.0000 | [0.0000; 0.0231] | 0.0% | 0.5% |
| Gong YH-2005 | 134 | 144 | 0.9306 | [0.8760; 0.9662] | 0.0% | 0.5% |
| Gu HY-2013 | 505 | 6258 | 0.0807 | [0.0741; 0.0877] | 1.0% | 0.6% |
| Yao XF-2013 | 806 | 2012 | 0.4006 | [0.3791; 0.4224] | 0.3% | 0.6% |
| He YW-2018 | 15 | 35857 | 0.0004 | [0.0002; 0.0007] | 6.0% | 0.6% |
| Wu Y-2016 | 482 | 2206 | 0.2185 | [0.2014; 0.2363] | 0.4% | 0.6% |
| Wu JY-2016 | 287 | 1529 | 0.1877 | [0.1684; 0.2082] | 0.3% | 0.5% |
| Wu JY-2016 | 112 | 312 | 0.3590 | [0.3057; 0.4149] | 0.1% | 0.5% |
| Yao MF-2007 | 635 | 1316 | 0.4825 | [0.4552; 0.5099] | 0.2% | 0.5% |
| Huang SY-2020 | 120 | 648 | 0.1852 | [0.1560; 0.2173] | 0.1% | 0.5% |
| Huang SM-2017 | 273 | 5345 | 0.0511 | [0.0453; 0.0573] | 0.9% | 0.6% |
| Meng ZH-2005 | 413 | 980 | 0.4214 | [0.3903; 0.4531] | 0.2% | 0.5% |
| Lu B-2008 | 208 | 1060 | 0.1962 | [0.1727; 0.2214] | 0.2% | 0.5% |
| Wang FD-2004 | 394 | 850 | 0.4635 | [0.4296; 0.4977] | 0.1% | 0.5% |
| Zheng YJ-2005 | 319 | 512 | 0.6230 | [0.5795; 0.6652] | 0.1% | 0.5% |
| Kong DG-2017 | 825 | 1945 | 0.4242 | [0.4021; 0.4465] | 0.3% | 0.6% |
| Li B-2003 | 14 | 178 | 0.0787 | [0.0437; 0.1284] | 0.0% | 0.5% |
| Li JT-2014 | 197 | 456 | 0.4320 | [0.3860; 0.4789] | 0.1% | 0.5% |
| Bo QN-2019 | 124 | 836 | 0.1483 | [0.1249; 0.1743] | 0.1% | 0.5% |
| Li MY-2008 | 169 | 768 | 0.2201 | [0.1912; 0.2510] | 0.1% | 0.5% |
| Li WJ-2007 | 337 | 1553 | 0.2170 | [0.1967; 0.2383] | 0.3% | 0.5% |
| Li YB-2004 | 572 | 3336 | 0.1715 | [0.1588; 0.1847] | 0.6% | 0.6% |
| Wang HR-2007 | 515 | 2209 | 0.2331 | [0.2156; 0.2513] | 0.4% | 0.6% |
| Lin CY-2009 | 403 | 4959 | 0.0813 | [0.0738; 0.0892] | 0.8% | 0.6% |
| Liu JY-2016 | 57 | 2127 | 0.0268 | [0.0204; 0.0346] | 0.4% | 0.6% |
| Liu K-2009 | 111 | 939 | 0.1182 | [0.0983; 0.1406] | 0.2% | 0.5% |
| Liu XG-2007 | 81 | 300 | 0.2700 | [0.2206; 0.3240] | 0.0% | 0.5% |
| Liu XG-2008 | 209 | 566 | 0.3693 | [0.3294; 0.4105] | 0.1% | 0.5% |
| Yu LM-2001 | 54 | 417 | 0.1295 | [0.0988; 0.1656] | 0.1% | 0.5% |
| Lu B-2008 | 373 | 828 | 0.4505 | [0.4162; 0.4851] | 0.1% | 0.5% |
| Lu J-2009 | 259 | 1977 | 0.1310 | [0.1164; 0.1467] | 0.3% | 0.6% |
| Lu YH-2006 | 421 | 663 | 0.6350 | [0.5971; 0.6717] | 0.1% | 0.5% |
| Luo YX-2005 | 215 | 3864 | 0.0556 | [0.0486; 0.0633] | 0.6% | 0.6% |
| Ma TW-2013 | 3 | 90 | 0.0333 | [0.0069; 0.0943] | 0.0% | 0.5% |
| Nong CS-2007 | 172 | 377 | 0.4562 | [0.4051; 0.5080] | 0.1% | 0.5% |
| Pan TJ-2002 | 0 | 1580 | 0.0000 | [0.0000; 0.0023] | 0.3% | 0.6% |
| Pan YL-2021 | 0 | 103794 | 0.0000 | [0.0000; 0.0000] | 17.3% | 0.6% |
| Shao HW-2009 | 41 | 648 | 0.0633 | [0.0458; 0.0849] | 0.1% | 0.5% |
| Sun ZH-2017 | 2555 | 47852 | 0.0534 | [0.0514; 0.0554] | 8.0% | 0.6% |
| Sun Z-2014 | 471 | 1483 | 0.3176 | [0.2939; 0.3420] | 0.2% | 0.5% |
| Tang WF-2014 | 61 | 158 | 0.3861 | [0.3098; 0.4667] | 0.0% | 0.5% |
| Tian JS-2007 | 35 | 778 | 0.0450 | [0.0315; 0.0620] | 0.1% | 0.5% |
| Wang DM-2016 | 28 | 1000 | 0.0280 | [0.0187; 0.0402] | 0.2% | 0.5% |
| Wang FY-1999 | 33 | 575 | 0.0574 | [0.0398; 0.0797] | 0.1% | 0.5% |
| Wang ZZ-2007 | 1105 | 1234 | 0.8955 | [0.8770; 0.9120] | 0.2% | 0.5% |
| Wang RL-2012 | 509 | 2028 | 0.2510 | [0.2322; 0.2705] | 0.3% | 0.6% |
| Wu CH-2003 | 4 | 148 | 0.0270 | [0.0074; 0.0678] | 0.0% | 0.5% |
| Wu JY-2017 | 310 | 1459 | 0.2125 | [0.1917; 0.2344] | 0.2% | 0.5% |
| Xia XW-2015 | 914 | 3513 | 0.2602 | [0.2457; 0.2750] | 0.6% | 0.6% |
| Xiao ZB-2022 | 1217 | 4661 | 0.2611 | [0.2485; 0.2740] | 0.8% | 0.6% |
| Xing XM-2011 | 185 | 812 | 0.2278 | [0.1994; 0.2583] | 0.1% | 0.5% |
| Yang F-2012 | 893 | 3771 | 0.2368 | [0.2233; 0.2507] | 0.6% | 0.6% |
| Yang B-2013 | 40 | 597 | 0.0670 | [0.0483; 0.0901] | 0.1% | 0.5% |
| Yin YZ-2001 | 63 | 676 | 0.0932 | [0.0724; 0.1177] | 0.1% | 0.5% |
| Yu DS-2011 | 338 | 2429 | 0.1392 | [0.1256; 0.1536] | 0.4% | 0.6% |
| Yu WX-2012 | 138 | 5000 | 0.0276 | [0.0232; 0.0325] | 0.8% | 0.6% |
| Zhang D-2022 | 1000 | 6493 | 0.1540 | [0.1453; 0.1630] | 1.1% | 0.6% |
| Zhang P-2015 | 47 | 1195 | 0.0393 | [0.0290; 0.0520] | 0.2% | 0.5% |
| Zhang XF-2007 | 2152 | 4139 | 0.5199 | [0.5046; 0.5353] | 0.7% | 0.6% |
| Zhao HL-2012 | 17 | 327 | 0.0520 | [0.0306; 0.0819] | 0.1% | 0.5% |
| Zheng RD-2013 | 207 | 850 | 0.2435 | [0.2150; 0.2738] | 0.1% | 0.5% |
| Zheng SJ-2015 | 55 | 205 | 0.2683 | [0.2090; 0.3345] | 0.0% | 0.5% |
| Zhong CF-2011 | 1074 | 2627 | 0.4088 | [0.3900; 0.4279] | 0.4% | 0.6% |
| Zhong SQ-2007 | 702 | 1239 | 0.5666 | [0.5385; 0.5944] | 0.2% | 0.5% |
| Zhu GZ-2015 | 458 | 2957 | 0.1549 | [0.1420; 0.1684] | 0.5% | 0.6% |
| Zhu GZ-2007 | 1127 | 4944 | 0.2280 | [0.2163; 0.2399] | 0.8% | 0.6% |
| Zhou HF-2006 | 47 | 175 | 0.2686 | [0.2045; 0.3407] | 0.0% | 0.5% |
| Cai Y-2017 | 366 | 1842 | 0.1987 | [0.1807; 0.2177] | 0.3% | 0.6% |
| Chan DP-2017 | 131 | 408 | 0.3211 | [0.2760; 0.3688] | 0.1% | 0.5% |
| Chang Y-2009 | 522 | 2572 | 0.2030 | [0.1876; 0.2190] | 0.4% | 0.6% |
| Chiu DM-2013 | 129 | 450 | 0.2867 | [0.2453; 0.3309] | 0.1% | 0.5% |
| Cong W-2014 | 244 | 965 | 0.2528 | [0.2257; 0.2815] | 0.2% | 0.5% |
| Dong C-2012 | 2793 | 14208 | 0.1966 | [0.1901; 0.2032] | 2.4% | 0.6% |
| Feng Y-2018 | 1337 | 1912 | 0.6993 | [0.6782; 0.7198] | 0.3% | 0.6% |
| Fu H-2010 | 70 | 296 | 0.2365 | [0.1892; 0.2891] | 0.0% | 0.5% |
| Shenyang G-2011 | 37 | 456 | 0.0811 | [0.0578; 0.1101] | 0.1% | 0.5% |
| Geng Y-2019 | 45 | 421 | 0.1069 | [0.0790; 0.1404] | 0.1% | 0.5% |
| Gu G-2015 | 57 | 994 | 0.0573 | [0.0437; 0.0737] | 0.2% | 0.5% |
| Huang F-2015 | 12 | 114 | 0.1053 | [0.0556; 0.1767] | 0.0% | 0.5% |
| Jia Z-2014 | 3719 | 15852 | 0.2346 | [0.2280; 0.2413] | 2.6% | 0.6% |
| Taniguchi M-2009 | 143 | 300 | 0.4767 | [0.4190; 0.5348] | 0.0% | 0.5% |
| Li H-2021 | 289 | 6269 | 0.0461 | [0.0410; 0.0516] | 1.0% | 0.6% |
| Li RC-2006 | 4839 | 10715 | 0.4516 | [0.4422; 0.4611] | 1.8% | 0.6% |
| Li W-2011 | 69 | 173 | 0.3988 | [0.3253; 0.4759] | 0.0% | 0.5% |
| Liang H-2014 | 74 | 193 | 0.3834 | [0.3145; 0.4560] | 0.0% | 0.5% |
| Liu KSH-2019 | 3408 | 10241 | 0.3328 | [0.3237; 0.3420] | 1.7% | 0.6% |
| Lu J-2009 | 1547 | 8762 | 0.1766 | [0.1686; 0.1847] | 1.5% | 0.6% |
| Ma XX-2021 | 13 | 290 | 0.0448 | [0.0241; 0.0754] | 0.0% | 0.5% |
| Ma Z-2010 | 407 | 2090 | 0.1947 | [0.1780; 0.2124] | 0.3% | 0.6% |
| Rui Z-2018 | 16 | 208 | 0.0769 | [0.0446; 0.1219] | 0.0% | 0.5% |
| Shu Y-2019 | 323 | 1232 | 0.2622 | [0.2378; 0.2877] | 0.2% | 0.5% |
| Wang Y-2018 | 528 | 1475 | 0.3580 | [0.3335; 0.3830] | 0.2% | 0.5% |
| Wong KH-2004 | 176 | 934 | 0.1884 | [0.1638; 0.2150] | 0.2% | 0.5% |
| Xue Y-2013 | 70 | 260 | 0.2692 | [0.2163; 0.3275] | 0.0% | 0.5% |
| Yu Y-2009 | 988 | 4508 | 0.2192 | [0.2072; 0.2315] | 0.7% | 0.6% |
| Zhang L-2018 | 519 | 2054 | 0.2527 | [0.2340; 0.2721] | 0.3% | 0.6% |
| Zhang L-2017 | 236 | 600 | 0.3933 | [0.3540; 0.4337] | 0.1% | 0.5% |
| Zhang W-2009 | 117 | 1476 | 0.0793 | [0.0660; 0.0942] | 0.2% | 0.5% |
| **Common effect model** | | **413997** | **0.0874** | **[0.0865; 0.0883]** | **68.9%** | **--** |
| **Random effects model** | | | **0.2149** | **[0.1804; 0.2516]** | **--** | **60.7%** |
| Heterogeneity: $I^2 = 100\%$, $\tau^2 = 0.0538$, $p = 0$ | | | | | | |

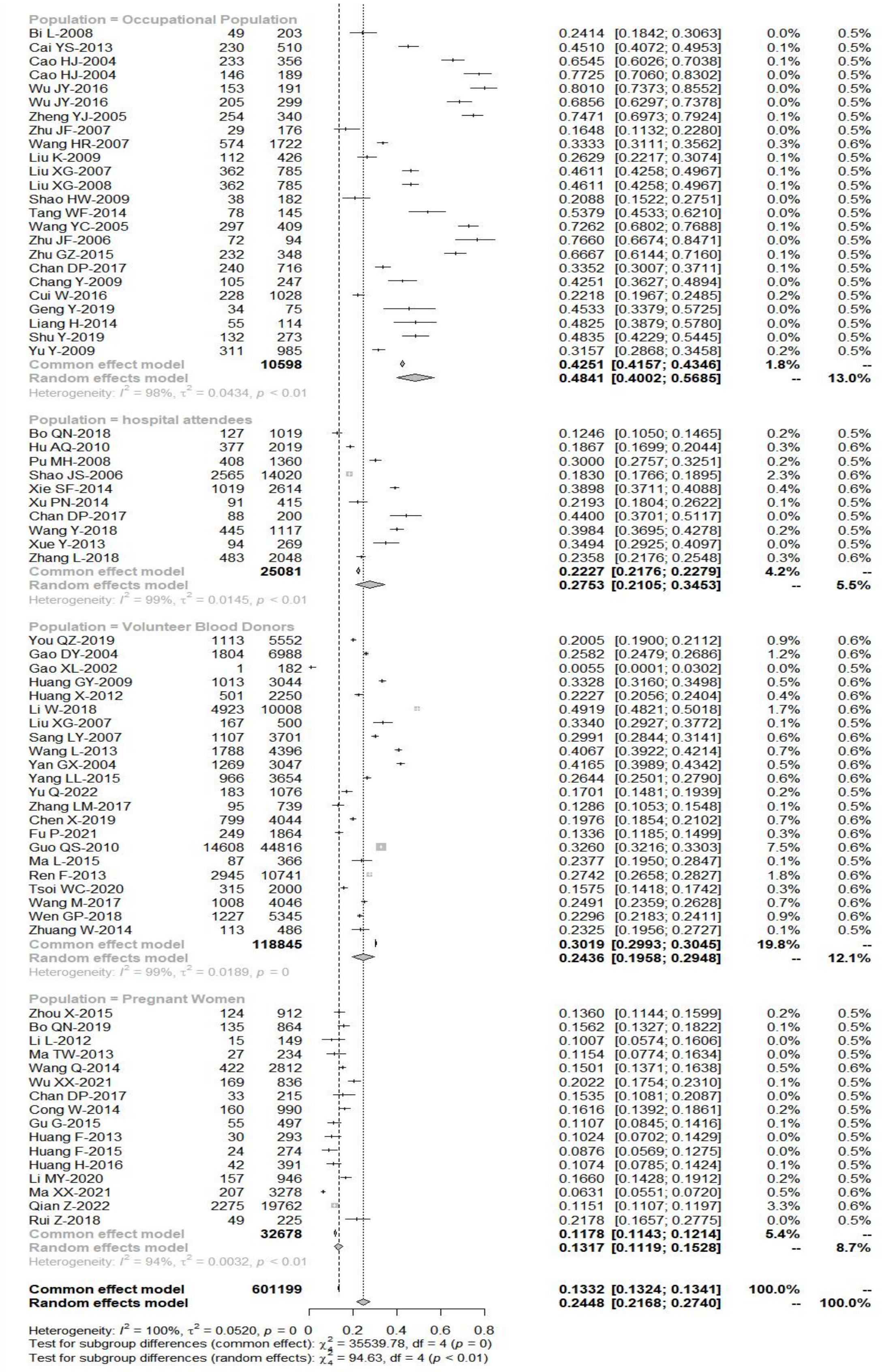


Figure S9. Forest plot of pooled anti-HEV IgG seroprevalence among different population

| Study | Events | Total | Proportion | 95%-CI | Weight (common) | Weight (random) |
|---|---|---|---|---|---|---|
| population = The General Population | | | | | | |
| Ao YY-2016 | 26 | 5012 | 0.0052 | [0.0034; 0.0076] | 0.4% | 0.8% |
| Bi L-2008 | 5 | 1001 | 0.0050 | [0.0016; 0.0116] | 0.1% | 0.8% |
| Cao HJ-2004 | 43 | 1934 | 0.0222 | [0.0161; 0.0298] | 0.1% | 0.8% |
| Zhang MM-2013 | 3 | 835 | 0.0036 | [0.0007; 0.0105] | 0.1% | 0.7% |
| Chen JP-2014 | 2 | 8697 | 0.0002 | [0.0000; 0.0008] | 0.7% | 0.8% |
| Chen JZ-2014 | 6 | 14275 | 0.0004 | [0.0002; 0.0009] | 1.1% | 0.8% |
| Chen JZ-2014 | 11 | 21612 | 0.0005 | [0.0003; 0.0009] | 1.7% | 0.8% |
| Chen K-2106 | 79 | 10156 | 0.0078 | [0.0062; 0.0097] | 0.8% | 0.8% |
| Jiang RJ-2006 | 17 | 1084 | 0.0157 | [0.0092; 0.0250] | 0.1% | 0.8% |
| Xu LL-2016 | 1 | 3225 | 0.0003 | [0.0000; 0.0017] | 0.2% | 0.8% |
| Duan ZJ-2014 | 15 | 10803 | 0.0014 | [0.0008; 0.0023] | 0.8% | 0.8% |
| Gao YP-2013 | 20 | 26780 | 0.0007 | [0.0005; 0.0012] | 2.1% | 0.8% |
| Ge SX-2006 | 108 | 4110 | 0.0263 | [0.0216; 0.0316] | 0.3% | 0.8% |
| Gong Q-2012 | 45 | 10220 | 0.0044 | [0.0032; 0.0059] | 0.8% | 0.8% |
| Guo QL-2011 | 2 | 240 | 0.0083 | [0.0010; 0.0298] | 0.0% | 0.6% |
| Wang XJ-2018 | 5 | 31696 | 0.0002 | [0.0001; 0.0004] | 2.4% | 0.8% |
| Han XX-2010 | 6 | 11362 | 0.0005 | [0.0002; 0.0011] | 0.9% | 0.8% |
| Yao MF-2007 | 35 | 1316 | 0.0266 | [0.0186; 0.0368] | 0.1% | 0.8% |
| Wang FD-2004 | 26 | 850 | 0.0306 | [0.0201; 0.0445] | 0.1% | 0.7% |
| Jiang T-2018 | 26 | 9797 | 0.0027 | [0.0017; 0.0039] | 0.8% | 0.8% |
| Jin LP-2012 | 6 | 11461 | 0.0005 | [0.0002; 0.0011] | 0.9% | 0.8% |
| Kong P-2013 | 22 | 33211 | 0.0007 | [0.0004; 0.0010] | 2.5% | 0.8% |
| Bo QN-2019 | 17 | 836 | 0.0203 | [0.0119; 0.0324] | 0.1% | 0.7% |
| Li SB-2010 | 4 | 76654 | 0.0001 | [0.0000; 0.0001] | 5.9% | 0.8% |
| Li WJ-2007 | 5 | 1553 | 0.0032 | [0.0010; 0.0075] | 0.1% | 0.8% |
| Li XJ-2019 | 55 | 16022 | 0.0034 | [0.0026; 0.0045] | 1.2% | 0.8% |
| Li YH-2021 | 36 | 25098 | 0.0014 | [0.0010; 0.0020] | 1.9% | 0.8% |
| Li TQ-2016 | 37 | 8257 | 0.0045 | [0.0032; 0.0062] | 0.6% | 0.8% |
| Lin Q-2012 | 4 | 5681 | 0.0007 | [0.0002; 0.0018] | 0.4% | 0.8% |
| Liu DX-2019 | 33 | 40182 | 0.0008 | [0.0006; 0.0012] | 3.1% | 0.8% |
| Liu F-2018 | 6 | 9529 | 0.0006 | [0.0002; 0.0014] | 0.7% | 0.8% |
| Liu JY-2016 | 3 | 2127 | 0.0014 | [0.0003; 0.0041] | 0.2% | 0.8% |
| Liu K-2009 | 1 | 939 | 0.0011 | [0.0000; 0.0059] | 0.1% | 0.8% |
| Liu XG-2007 | 5 | 300 | 0.0167 | [0.0054; 0.0385] | 0.0% | 0.7% |
| Liu Y-2013 | 115 | 25391 | 0.0045 | [0.0037; 0.0054] | 1.9% | 0.8% |
| Luo Y-2011 | 7 | 31137 | 0.0002 | [0.0001; 0.0005] | 2.4% | 0.8% |
| Ma SB-2014 | 11 | 5639 | 0.0020 | [0.0010; 0.0035] | 0.4% | 0.8% |
| Ma XL-2015 | 12 | 3707 | 0.0032 | [0.0017; 0.0056] | 0.3% | 0.8% |
| Zhang LF-2003 | 19 | 2223 | 0.0085 | [0.0052; 0.0133] | 0.2% | 0.8% |
| Nong HY-2013 | 7 | 11938 | 0.0006 | [0.0002; 0.0012] | 0.9% | 0.8% |
| Qiu SH-2013 | 27 | 27977 | 0.0010 | [0.0006; 0.0014] | 2.1% | 0.8% |
| Shao HW-2009 | 1 | 648 | 0.0015 | [0.0000; 0.0086] | 0.0% | 0.7% |
| Sun C-2016 | 214 | 128833 | 0.0017 | [0.0014; 0.0019] | 9.9% | 0.8% |
| Sun JW-2009 | 65 | 3994 | 0.0163 | [0.0126; 0.0207] | 0.3% | 0.8% |
| Sun LP-2004 | 20 | 1043 | 0.0192 | [0.0118; 0.0295] | 0.1% | 0.8% |
| Tian GJ-2007 | 13 | 288 | 0.0451 | [0.0243; 0.0760] | 0.0% | 0.6% |
| Wang DM-2016 | 2 | 1000 | 0.0020 | [0.0002; 0.0072] | 0.1% | 0.8% |
| Wang JF-2021 | 29 | 11917 | 0.0024 | [0.0016; 0.0035] | 0.9% | 0.8% |
| Wang LP-2013 | 10 | 25180 | 0.0004 | [0.0002; 0.0007] | 1.9% | 0.8% |
| Wang XH-2018 | 41 | 36152 | 0.0011 | [0.0008; 0.0015] | 2.8% | 0.8% |
| Wu ZT-2013 | 14 | 4441 | 0.0032 | [0.0017; 0.0053] | 0.3% | 0.8% |
| Wu ZH-2017 | 55 | 32120 | 0.0017 | [0.0013; 0.0022] | 2.5% | 0.8% |
| Wu JY-2017 | 7 | 1459 | 0.0048 | [0.0019; 0.0099] | 0.1% | 0.8% |
| Xia C-2012 | 11 | 749 | 0.0147 | [0.0074; 0.0261] | 0.1% | 0.7% |
| Xiao ZY-2016 | 32 | 8952 | 0.0036 | [0.0024; 0.0050] | 0.7% | 0.8% |
| Xing Y-2016 | 164 | 95217 | 0.0017 | [0.0015; 0.0020] | 7.3% | 0.8% |
| Xu WL-2018 | 21 | 15897 | 0.0013 | [0.0008; 0.0020] | 1.2% | 0.8% |
| Yang XY-2016 | 26 | 12271 | 0.0021 | [0.0014; 0.0031] | 0.9% | 0.8% |
| Yuan ZZ-2022 | 10 | 1604 | 0.0062 | [0.0030; 0.0114] | 0.1% | 0.8% |
| Zhang P-2015 | 3 | 1195 | 0.0025 | [0.0005; 0.0073] | 0.1% | 0.8% |
| Zhang WS-2012 | 3 | 6456 | 0.0005 | [0.0001; 0.0014] | 0.5% | 0.8% |
| Zhang XF-2007 | 103 | 4139 | 0.0249 | [0.0204; 0.0301] | 0.3% | 0.8% |
| Zhao HL-2012 | 8 | 327 | 0.0245 | [0.0106; 0.0476] | 0.0% | 0.7% |
| Zhao JN-2017 | 10 | 10000 | 0.0010 | [0.0005; 0.0018] | 0.8% | 0.8% |
| Zheng RD-2013 | 0 | 850 | 0.0000 | [0.0000; 0.0043] | 0.1% | 0.7% |
| Zheng Y-2015 | 21 | 26583 | 0.0008 | [0.0005; 0.0012] | 2.0% | 0.8% |
| Zheng Y-2014 | 124 | 118253 | 0.0010 | [0.0009; 0.0013] | 9.1% | 0.8% |
| Cai Y-2017 | 21 | 1842 | 0.0114 | [0.0071; 0.0174] | 0.1% | 0.8% |
| Cong W-2014 | 35 | 965 | 0.0363 | [0.0254; 0.0501] | 0.1% | 0.8% |
| Dong C-2012 | 4 | 14208 | 0.0003 | [0.0001; 0.0007] | 1.1% | 0.8% |
| Feng Y-2018 | 80 | 1912 | 0.0418 | [0.0333; 0.0518] | 0.1% | 0.8% |
| Geng Y-2019 | 5 | 421 | 0.0119 | [0.0039; 0.0275] | 0.0% | 0.7% |
| Gu G-2015 | 4 | 994 | 0.0040 | [0.0011; 0.0103] | 0.1% | 0.8% |
| Huang F-2015 | 5 | 114 | 0.0439 | [0.0144; 0.0994] | 0.0% | 0.5% |
| Li H-2021 | 11 | 6269 | 0.0018 | [0.0009; 0.0031] | 0.5% | 0.8% |
| Li W-2011 | 11 | 173 | 0.0636 | [0.0322; 0.1109] | 0.0% | 0.6% |
| Ma XX-2021 | 0 | 290 | 0.0000 | [0.0000; 0.0126] | 0.0% | 0.6% |
| Ma Z-2010 | 9 | 2090 | 0.0043 | [0.0020; 0.0082] | 0.2% | 0.8% |
| Rui Z-2018 | 0 | 208 | 0.0000 | [0.0000; 0.0176] | 0.0% | 0.6% |
| Shu Y-2019 | 22 | 1232 | 0.0179 | [0.0112; 0.0269] | 0.1% | 0.8% |
| Wang Y-2018 | 10 | 1475 | 0.0068 | [0.0033; 0.0124] | 0.1% | 0.8% |
| Yu Y-2009 | 65 | 3994 | 0.0163 | [0.0126; 0.0207] | 0.3% | 0.8% |
| Zhang W-2009 | 29 | 1476 | 0.0196 | [0.0132; 0.0281] | 0.1% | 0.8% |
| Liu XJ-2008 | 11 | 502 | 0.0219 | [0.0110; 0.0389] | 0.0% | 0.7% |
| **Common effect model** | | **1102600** | **0.0010** | **[0.0010; 0.0011]** | **84.7%** | -- |
| **Random effects model** | | | **0.0049** | **[0.0033; 0.0067]** | -- | **64.6%** |
| Heterogeneity: $I^2 = 97\%$, $\tau^2 = 0.0027$, $p = 0$ | | | | | | |

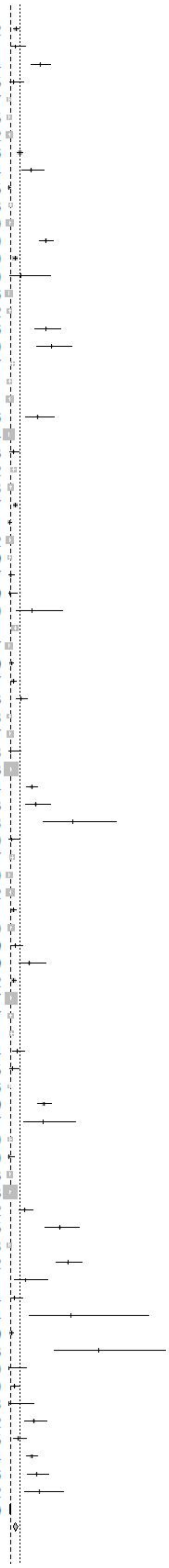

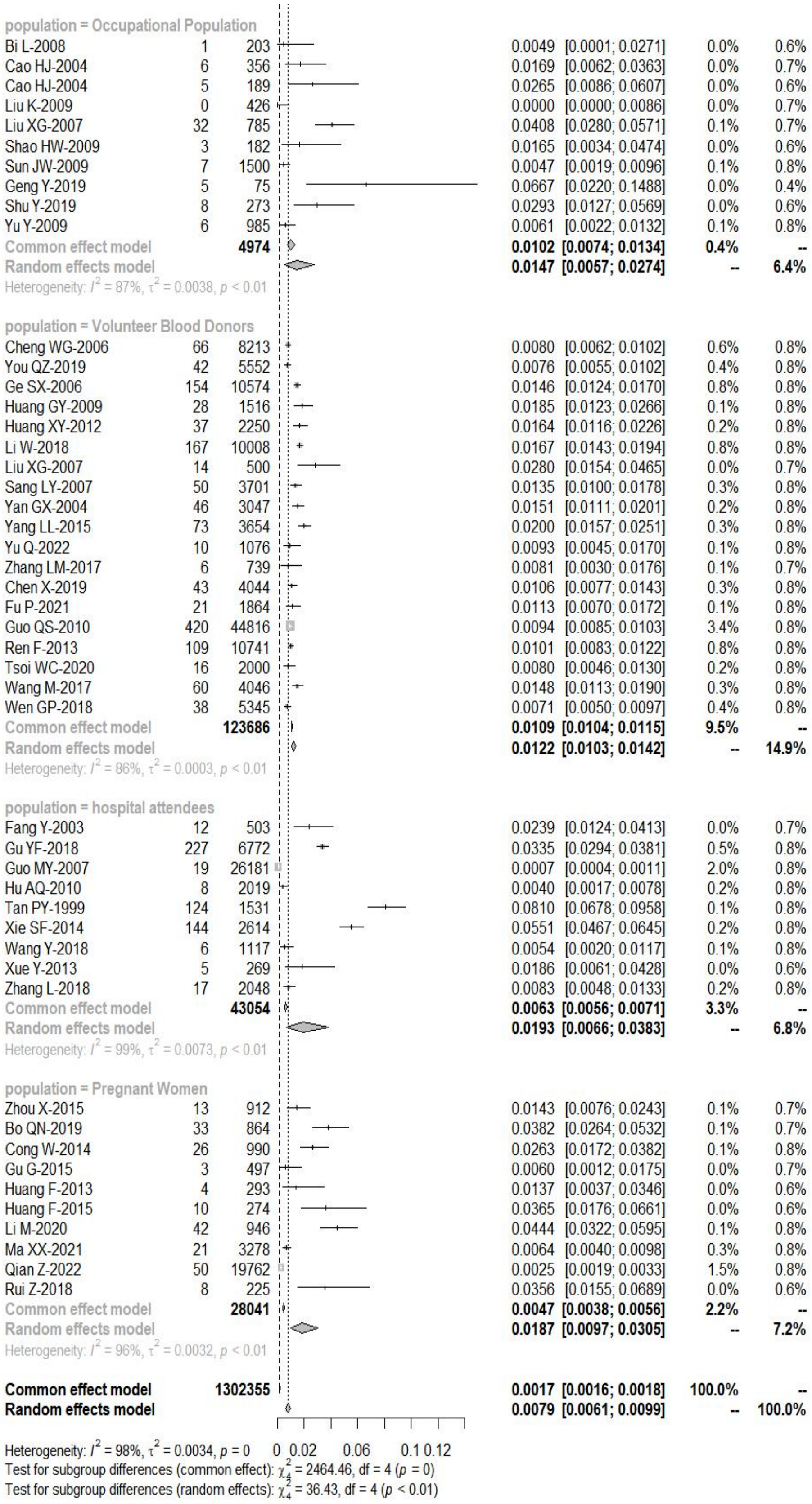


Figure S10. Forest plot of pooled anti-HEV IgM seroprevalence among different population

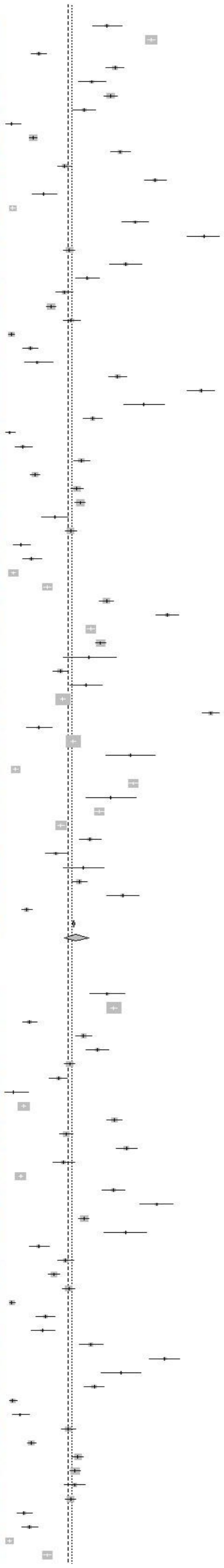

| Study | Events | Total | Proportion | 95%-CI | Weight (common) | Weight (random) |
|---|---|---|---|---|---|---|
| **Gender = Male** | | | | | | |
| Zhang ZX-2003 | 122 | 334 | 0.3653 | [0.3144; 0.4177] | 0.2% | 0.8% |
| Ai X-2009 | 2739 | 5243 | 0.5224 | [0.5089; 0.5359] | 2.9% | 0.8% |
| Bi L-2008 | 64 | 522 | 0.1226 | [0.0958; 0.1522] | 0.3% | 0.8% |
| Cao HJ-2004 | 356 | 902 | 0.3947 | [0.3630; 0.4268] | 0.5% | 0.8% |
| Chen XM-2014 | 108 | 346 | 0.3121 | [0.2643; 0.3620] | 0.2% | 0.8% |
| Ning LF-2008 | 664 | 1751 | 0.3792 | [0.3566; 0.4021] | 1.0% | 0.8% |
| Chen YZ-2006 | 139 | 488 | 0.2848 | [0.2456; 0.3258] | 0.3% | 0.8% |
| Du L-2013 | 4 | 148 | 0.0270 | [0.0058; 0.0606] | 0.1% | 0.8% |
| Gu HY-2013 | 205 | 1984 | 0.1033 | [0.0903; 0.1171] | 1.1% | 0.8% |
| Yao XF-2013 | 299 | 724 | 0.4130 | [0.3773; 0.4491] | 0.4% | 0.8% |
| Wu Y-2016 | 207 | 960 | 0.2156 | [0.1902; 0.2422] | 0.5% | 0.8% |
| Yao MF-2007 | 326 | 606 | 0.5380 | [0.4981; 0.5775] | 0.3% | 0.8% |
| Huang SY-2020 | 34 | 241 | 0.1411 | [0.0998; 0.1881] | 0.1% | 0.8% |
| Huang SM-2017 | 50 | 1590 | 0.0314 | [0.0234; 0.0406] | 0.9% | 0.8% |
| Meng ZH-2005 | 193 | 414 | 0.4662 | [0.4183; 0.5144] | 0.2% | 0.8% |
| Zheng YJ-2005 | 176 | 248 | 0.7097 | [0.6515; 0.7646] | 0.1% | 0.8% |
| Kong DG-2017 | 379 | 1636 | 0.2317 | [0.2115; 0.2524] | 0.9% | 0.8% |
| Li JT-2014 | 125 | 289 | 0.4325 | [0.3758; 0.4901] | 0.2% | 0.8% |
| Li MY-2008 | 126 | 426 | 0.2958 | [0.2533; 0.3401] | 0.2% | 0.8% |
| Li WJ-2007 | 156 | 727 | 0.2146 | [0.1855; 0.2452] | 0.4% | 0.8% |
| Li YB-2004 | 337 | 2014 | 0.1673 | [0.1513; 0.1840] | 1.1% | 0.8% |
| Wang HR-2007 | 185 | 772 | 0.2396 | [0.2102; 0.2704] | 0.4% | 0.8% |
| Liu JY-2016 | 28 | 1091 | 0.0257 | [0.0170; 0.0360] | 0.6% | 0.8% |
| Liu K-2009 | 46 | 497 | 0.0926 | [0.0685; 0.1197] | 0.3% | 0.8% |
| Yu LM-2001 | 19 | 160 | 0.1187 | [0.0727; 0.1739] | 0.1% | 0.8% |
| Lu B-2008 | 400 | 991 | 0.4036 | [0.3733; 0.4344] | 0.5% | 0.8% |
| Lu YH-2006 | 229 | 327 | 0.7003 | [0.6494; 0.7488] | 0.2% | 0.8% |
| Nong CS-2007 | 93 | 187 | 0.4973 | [0.4257; 0.5691] | 0.1% | 0.8% |
| Sun Z-2014 | 242 | 768 | 0.3151 | [0.2827; 0.3484] | 0.4% | 0.8% |
| Wang DM-2016 | 5 | 265 | 0.0189 | [0.0053; 0.0394] | 0.1% | 0.8% |
| Wang FY-1999 | 19 | 283 | 0.0671 | [0.0406; 0.0995] | 0.2% | 0.8% |
| Wang RL-2012 | 268 | 974 | 0.2752 | [0.2475; 0.3037] | 0.5% | 0.8% |
| Wu JY-2017 | 162 | 1459 | 0.1110 | [0.0954; 0.1277] | 0.8% | 0.8% |
| Xia XW-2015 | 435 | 1678 | 0.2592 | [0.2385; 0.2805] | 0.9% | 0.8% |
| Xiao ZB-2022 | 570 | 2108 | 0.2704 | [0.2516; 0.2896] | 1.2% | 0.8% |
| Xing XM-2011 | 47 | 261 | 0.1801 | [0.1357; 0.2292] | 0.1% | 0.8% |
| Yang F-2012 | 435 | 1834 | 0.2372 | [0.2180; 0.2569] | 1.0% | 0.8% |
| Yang B-2013 | 15 | 245 | 0.0612 | [0.0342; 0.0951] | 0.1% | 0.8% |
| Yin YZ-2001 | 30 | 310 | 0.0968 | [0.0662; 0.1324] | 0.2% | 0.8% |
| Yu WX-2012 | 96 | 2852 | 0.0337 | [0.0273; 0.0406] | 1.6% | 0.8% |
| Zhang D-2022 | 466 | 3024 | 0.1541 | [0.1414; 0.1672] | 1.7% | 0.8% |
| Zhong CF-2011 | 567 | 1558 | 0.3639 | [0.3402; 0.3880] | 0.9% | 0.8% |
| Zhong SQ-2007 | 310 | 534 | 0.5805 | [0.5384; 0.6221] | 0.3% | 0.8% |
| Zhu GZ-2015 | 1176 | 3810 | 0.3087 | [0.2941; 0.3234] | 2.1% | 0.8% |
| Zhu GZ-2007 | 863 | 2518 | 0.3427 | [0.3243; 0.3614] | 1.4% | 0.8% |
| Zhou HF-2006 | 28 | 93 | 0.3011 | [0.2116; 0.3987] | 0.1% | 0.7% |
| Cai Y-2017 | 169 | 846 | 0.1998 | [0.1735; 0.2274] | 0.5% | 0.8% |
| Chiu DM-2013 | 74 | 255 | 0.2902 | [0.2360; 0.3475] | 0.1% | 0.8% |
| Dong C-2012 | 1596 | 7620 | 0.2094 | [0.2004; 0.2187] | 4.2% | 0.8% |
| Feng Y-2018 | 588 | 800 | 0.7350 | [0.7038; 0.7650] | 0.4% | 0.8% |
| Geng Y-2019 | 24 | 196 | 0.1224 | [0.0799; 0.1724] | 0.1% | 0.8% |
| Jia Z-2014 | 1970 | 7996 | 0.2464 | [0.2370; 0.2559] | 4.4% | 0.8% |
| Taniguchi M-2009 | 56 | 125 | 0.4480 | [0.3615; 0.5361] | 0.1% | 0.8% |
| Li H-2021 | 111 | 2680 | 0.0414 | [0.0342; 0.0493] | 1.5% | 0.8% |
| Li RC-2006 | 1576 | 3440 | 0.4581 | [0.4415; 0.4748] | 1.9% | 0.8% |
| Liang H-2014 | 44 | 116 | 0.3793 | [0.2929; 0.4697] | 0.1% | 0.8% |
| Liu KSH-2019 | 1137 | 3353 | 0.3391 | [0.3232; 0.3552] | 1.8% | 0.8% |
| Lu J-2009 | 941 | 4647 | 0.2025 | [0.1911; 0.2142] | 2.5% | 0.8% |
| Shu Y-2019 | 178 | 582 | 0.3058 | [0.2690; 0.3439] | 0.3% | 0.8% |
| Wong KH-2004 | 69 | 374 | 0.1845 | [0.1467; 0.2255] | 0.2% | 0.8% |
| Xue Y-2013 | 42 | 149 | 0.2819 | [0.2123; 0.3571] | 0.1% | 0.8% |
| Zhang L-2018 | 281 | 1054 | 0.2666 | [0.2403; 0.2937] | 0.6% | 0.8% |
| Zhang L-2017 | 121 | 287 | 0.4216 | [0.3650; 0.4793] | 0.2% | 0.8% |
| Zhang W-2009 | 63 | 779 | 0.0809 | [0.0627; 0.1011] | 0.4% | 0.8% |
| **Common effect model** | | **85491** | **0.2485** | **[0.2456; 0.2514]** | **46.7%** | -- |
| **Random effects model** | | | **0.2564** | **[0.2133; 0.3020]** | -- | **50.0%** |
| Heterogeneity: $I^2 = 99\%$, $\tau^2 = 0.0423$, $p = 0$ | | | | | | |
| **Gender = Female** | | | | | | |
| Zhang ZX-2003 | 88 | 240 | 0.3667 | [0.3067; 0.4287] | 0.1% | 0.8% |
| Ai X-2009 | 2849 | 7312 | 0.3896 | [0.3785; 0.4008] | 4.0% | 0.8% |
| Bi L-2008 | 43 | 479 | 0.0898 | [0.0657; 0.1171] | 0.3% | 0.8% |
| Cao HJ-2004 | 292 | 1032 | 0.2829 | [0.2559; 0.3108] | 0.6% | 0.8% |
| Chen XM-2014 | 173 | 522 | 0.3314 | [0.2916; 0.3724] | 0.3% | 0.8% |
| Ning LF-2008 | 422 | 1810 | 0.2331 | [0.2139; 0.2529] | 1.0% | 0.8% |
| Chen YZ-2006 | 115 | 596 | 0.1930 | [0.1622; 0.2257] | 0.3% | 0.8% |
| Du L-2013 | 3 | 87 | 0.0345 | [0.0043; 0.0856] | 0.0% | 0.7% |
| Gu HY-2013 | 300 | 4274 | 0.0702 | [0.0627; 0.0780] | 2.3% | 0.8% |
| Yao XF-2013 | 507 | 1288 | 0.3936 | [0.3671; 0.4205] | 0.7% | 0.8% |
| Wu Y-2016 | 274 | 1246 | 0.2199 | [0.1973; 0.2433] | 0.7% | 0.8% |
| Yao MF-2007 | 309 | 710 | 0.4352 | [0.3989; 0.4719] | 0.4% | 0.8% |
| Huang SY-2020 | 86 | 407 | 0.2113 | [0.1729; 0.2524] | 0.2% | 0.8% |
| Huang SM-2017 | 223 | 3755 | 0.0594 | [0.0520; 0.0672] | 2.1% | 0.8% |
| Meng ZH-2005 | 220 | 566 | 0.3887 | [0.3489; 0.4292] | 0.3% | 0.8% |
| Zheng YJ-2005 | 143 | 264 | 0.5417 | [0.4812; 0.6015] | 0.1% | 0.8% |
| Kong DG-2017 | 639 | 2254 | 0.2835 | [0.2651; 0.3023] | 1.2% | 0.8% |
| Li JT-2014 | 72 | 167 | 0.4311 | [0.3568; 0.5071] | 0.1% | 0.8% |
| Li MY-2008 | 42 | 342 | 0.1228 | [0.0900; 0.1598] | 0.2% | 0.8% |
| Li WJ-2007 | 181 | 826 | 0.2191 | [0.1916; 0.2480] | 0.5% | 0.8% |
| Li YB-2004 | 235 | 1322 | 0.1778 | [0.1576; 0.1989] | 0.7% | 0.8% |
| Wang HR-2007 | 330 | 1437 | 0.2296 | [0.2083; 0.2518] | 0.8% | 0.8% |
| Liu JY-2016 | 29 | 1036 | 0.0280 | [0.0187; 0.0390] | 0.6% | 0.8% |
| Liu K-2009 | 65 | 442 | 0.1471 | [0.1155; 0.1817] | 0.2% | 0.8% |
| Yu LM-2001 | 35 | 257 | 0.1362 | [0.0968; 0.1810] | 0.1% | 0.8% |
| Lu B-2008 | 146 | 473 | 0.3087 | [0.2678; 0.3511] | 0.3% | 0.8% |
| Lu YH-2006 | 192 | 336 | 0.5714 | [0.5181; 0.6240] | 0.2% | 0.8% |
| Nong CS-2007 | 79 | 190 | 0.4158 | [0.3465; 0.4868] | 0.1% | 0.8% |
| Sun Z-2014 | 229 | 715 | 0.3203 | [0.2865; 0.3550] | 0.4% | 0.8% |
| Wang DM-2016 | 23 | 735 | 0.0313 | [0.0198; 0.0452] | 0.4% | 0.8% |
| Wang FY-1999 | 14 | 247 | 0.0567 | [0.0309; 0.0894] | 0.1% | 0.8% |
| Wang RL-2012 | 241 | 1054 | 0.2287 | [0.2038; 0.2545] | 0.6% | 0.8% |
| Wu JY-2017 | 148 | 1521 | 0.0973 | [0.0829; 0.1127] | 0.8% | 0.8% |
| Xia XW-2015 | 481 | 1835 | 0.2621 | [0.2422; 0.2825] | 1.0% | 0.8% |
| Xiao ZB-2022 | 645 | 2550 | 0.2529 | [0.2363; 0.2700] | 1.4% | 0.8% |
| Xing XM-2011 | 138 | 551 | 0.2505 | [0.2151; 0.2875] | 0.3% | 0.8% |
| Yang F-2012 | 458 | 1937 | 0.2364 | [0.2178; 0.2556] | 1.1% | 0.8% |
| Yang B-2013 | 25 | 352 | 0.0710 | [0.0463; 0.1004] | 0.2% | 0.8% |
| Yin YZ-2001 | 33 | 366 | 0.0902 | [0.0628; 0.1218] | 0.2% | 0.8% |
| Yu WX-2012 | 42 | 2148 | 0.0196 | [0.0141; 0.0259] | 1.2% | 0.8% |
| Zhang D-2022 | 534 | 3469 | 0.1539 | [0.1421; 0.1661] | 1.9% | 0.8% |

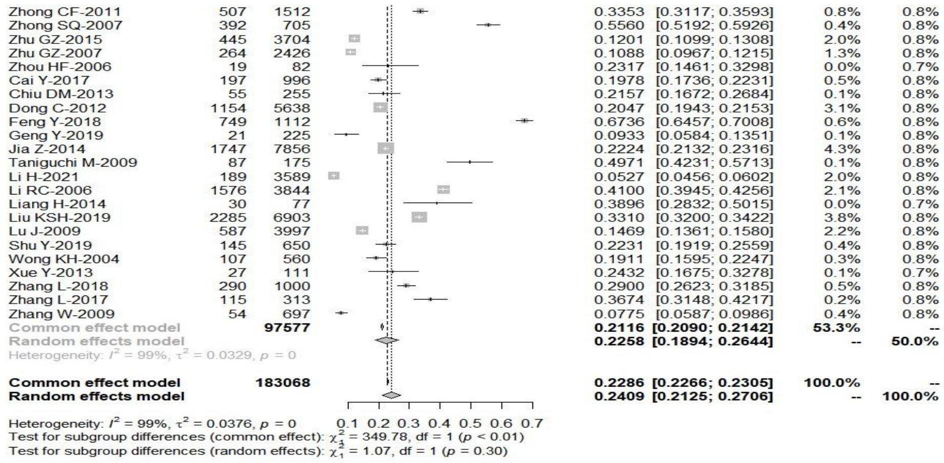


Figure S11. Forest plot of anti-HEV IgG prevalence by gender in the general population

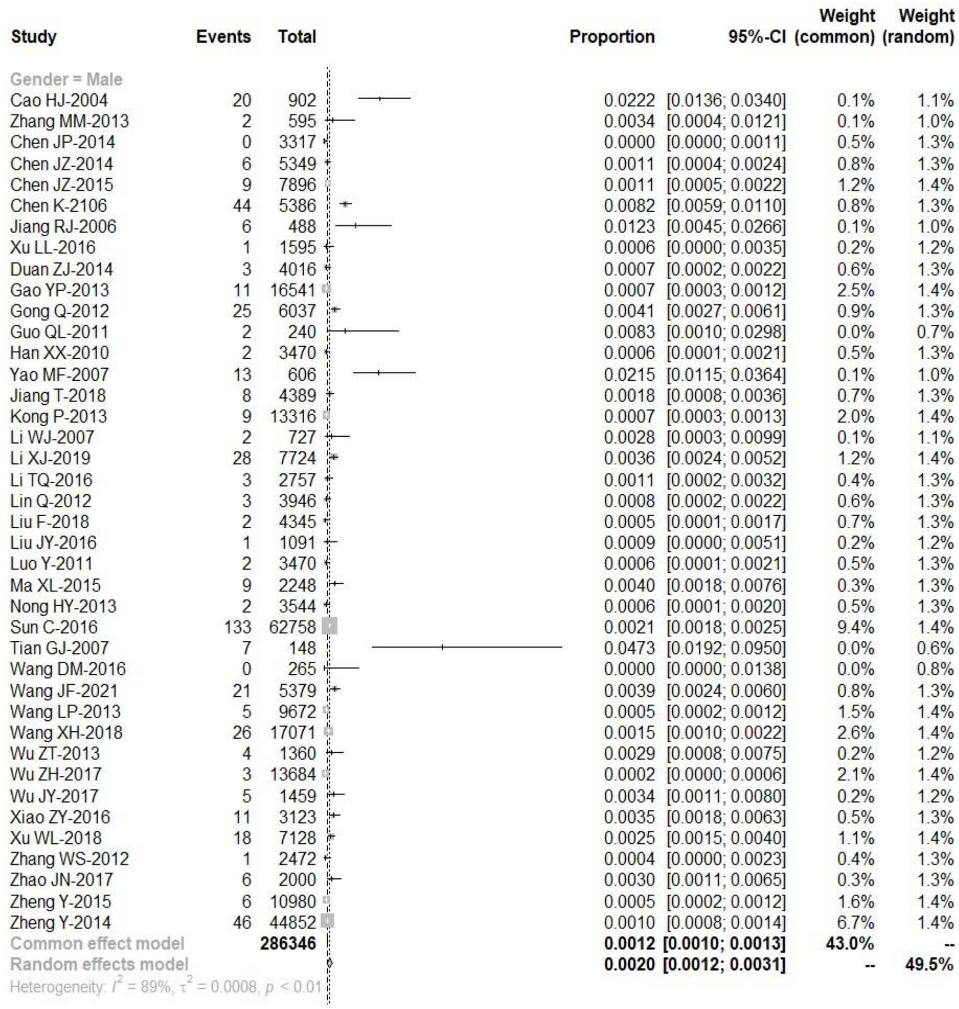

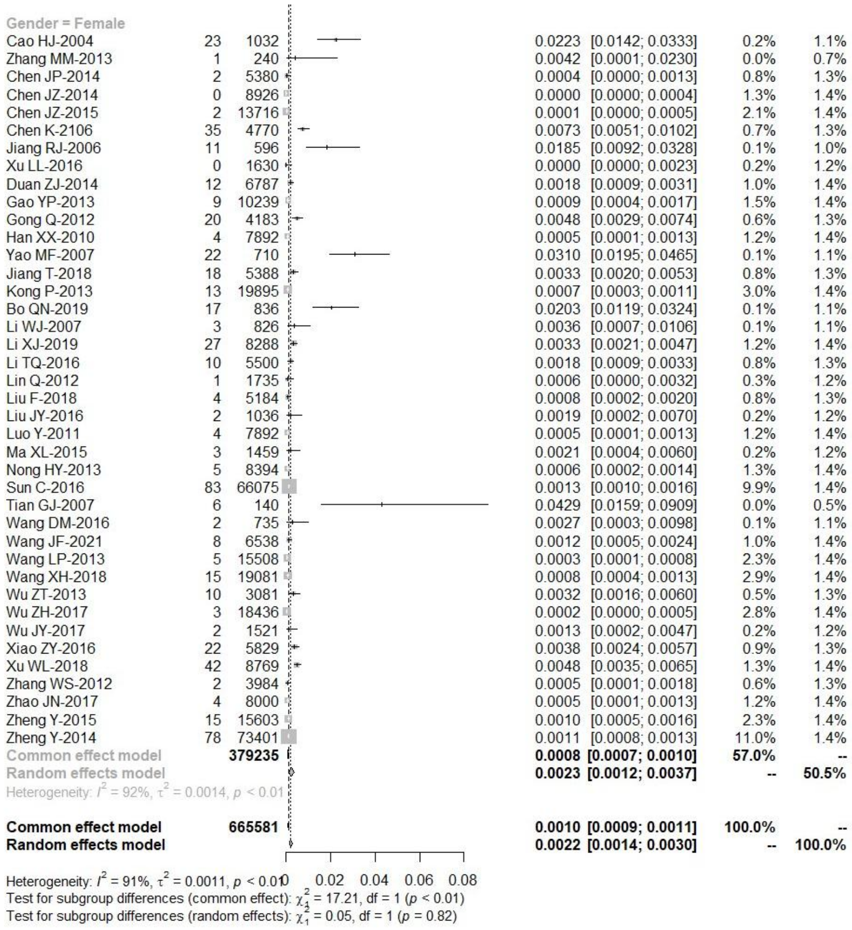


Figure S12. Forest plot of anti-HEV IgM prevalence by gender in the general population

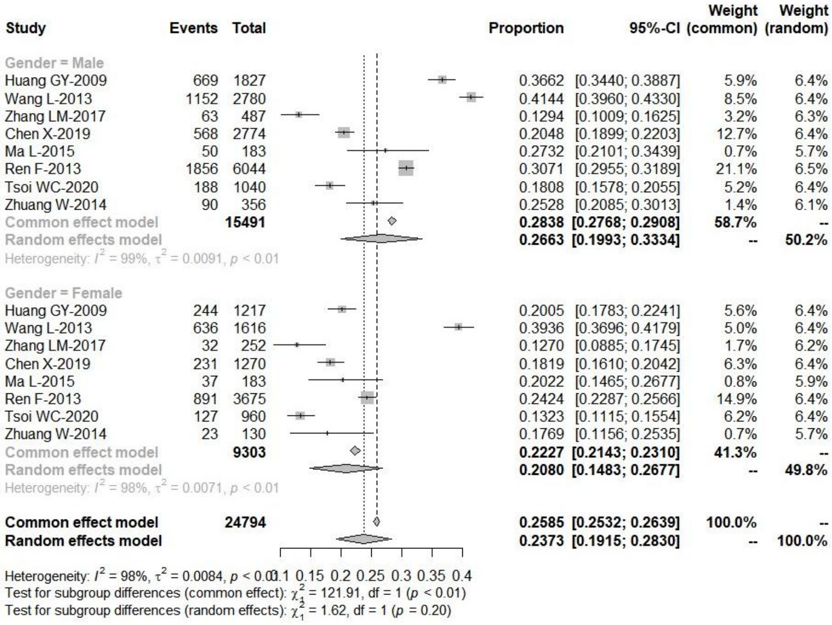


Figure S13. Forest plot of anti-HEV IgG prevalence by gender in volunteer blood donors

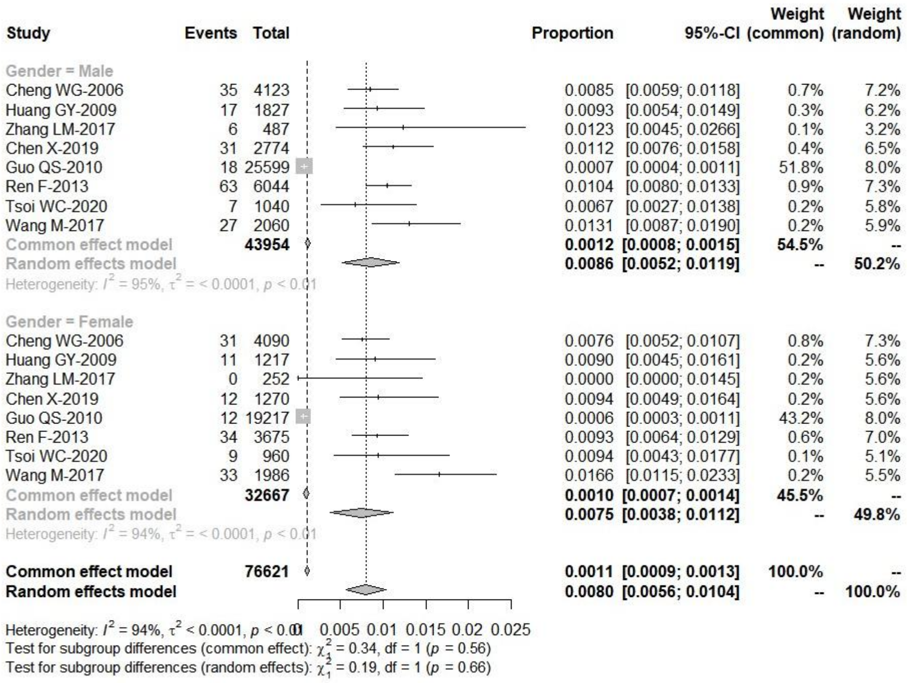


Figure S14. Forest plot of anti-HEV IgM prevalence by gender in volunteer blood donors

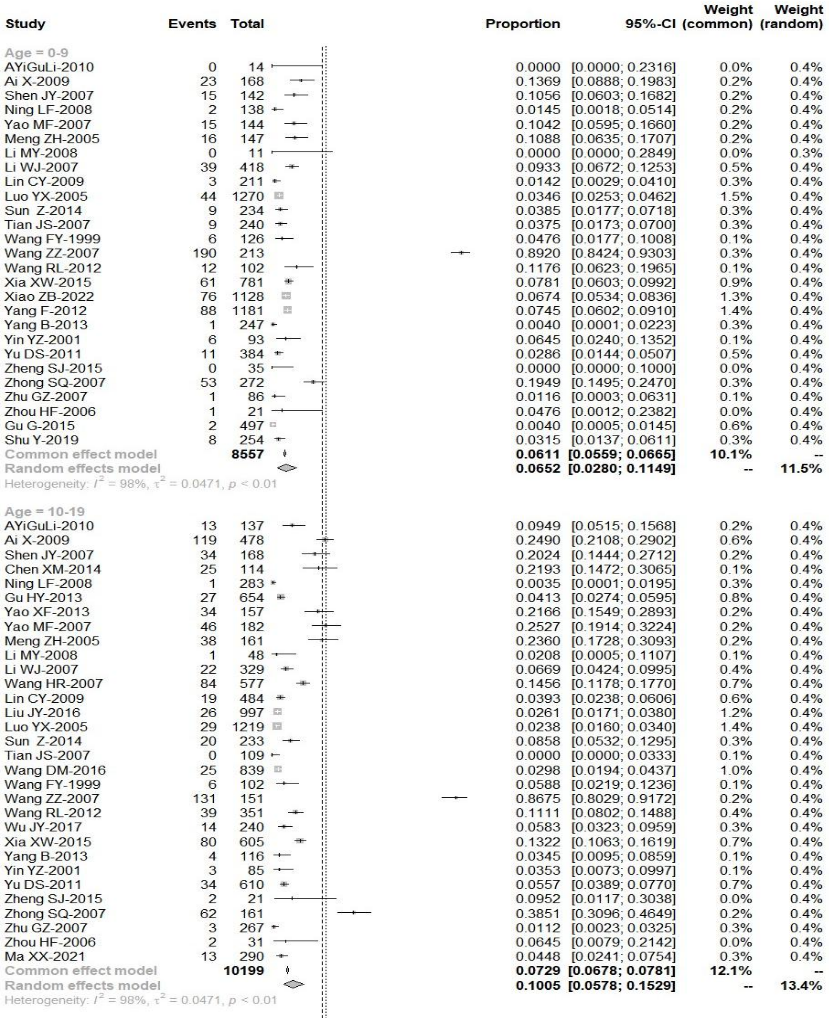

| | | | | | | |
|---|---|---|---|---|---|---|
| **Age = 20-29** | | | | | | |
| Ai X-2009 | 281 | 897 | 0.3133 | [0.2830; 0.3448] | 1.1% | 0.4% |
| Shen JY-2007 | 59 | 175 | 0.3371 | [0.2676; 0.4124] | 0.2% | 0.4% |
| Chen XM-2014 | 62 | 284 | 0.2183 | [0.1717; 0.2709] | 0.3% | 0.4% |
| Ning LF-2008 | 25 | 346 | 0.0723 | [0.0473; 0.1048] | 0.4% | 0.4% |
| Gu HY-2013 | 193 | 3522 | 0.0548 | [0.0475; 0.0628] | 4.2% | 0.4% |
| Yao XF-2013 | 192 | 739 | 0.2598 | [0.2285; 0.2930] | 0.9% | 0.4% |
| Yao MF-2007 | 60 | 152 | 0.3947 | [0.3165; 0.4772] | 0.2% | 0.4% |
| Meng ZH-2005 | 43 | 106 | 0.4057 | [0.3113; 0.5054] | 0.1% | 0.4% |
| Li MY-2008 | 8 | 79 | 0.1013 | [0.0447; 0.1898] | 0.1% | 0.4% |
| Li WJ-2007 | 11 | 105 | 0.1048 | [0.0535; 0.1797] | 0.1% | 0.4% |
| Wang HR-2007 | 170 | 701 | 0.2425 | [0.2112; 0.2760] | 0.8% | 0.4% |
| Lin CY-2009 | 24 | 583 | 0.0412 | [0.0266; 0.0606] | 0.7% | 0.4% |
| Liu JY-2016 | 31 | 1130 | 0.0274 | [0.0187; 0.0387] | 1.3% | 0.4% |
| Liu XG-2008 | 35 | 145 | 0.2414 | [0.1743; 0.3194] | 0.2% | 0.4% |
| Yu LM-2001 | 29 | 230 | 0.1261 | [0.0861; 0.1760] | 0.3% | 0.4% |
| Lu B-2008 | 42 | 98 | 0.4286 | [0.3290; 0.5325] | 0.1% | 0.4% |
| Lu YH-2006 | 11 | 20 | 0.5500 | [0.3153; 0.7694] | 0.0% | 0.4% |
| Luo YX-2005 | 24 | 297 | 0.0808 | [0.0525; 0.1179] | 0.4% | 0.4% |
| Sun Z-2014 | 69 | 256 | 0.2695 | [0.2162; 0.3283] | 0.3% | 0.4% |
| Tang WF-2014 | 5 | 34 | 0.1471 | [0.0495; 0.3106] | 0.0% | 0.4% |
| Tian JS-2007 | 5 | 120 | 0.0417 | [0.0137; 0.0946] | 0.1% | 0.4% |
| Wang DM-2016 | 3 | 161 | 0.0186 | [0.0039; 0.0535] | 0.2% | 0.4% |
| Wang FY-1999 | 7 | 83 | 0.0843 | [0.0346; 0.1661] | 0.1% | 0.4% |
| Wang ZZ-2007 | 95 | 108 | 0.8796 | [0.8030; 0.9343] | 0.1% | 0.4% |
| Wang RL-2012 | 64 | 408 | 0.1569 | [0.1229; 0.1958] | 0.5% | 0.4% |
| Wu JY-2017 | 36 | 230 | 0.1565 | [0.1121; 0.2100] | 0.3% | 0.4% |
| Xia XW-2015 | 105 | 483 | 0.2174 | [0.1814; 0.2569] | 0.6% | 0.4% |
| Xing XM-2011 | 53 | 323 | 0.1641 | [0.1254; 0.2091] | 0.4% | 0.4% |
| Yang F-2012 | 77 | 585 | 0.1316 | [0.1053; 0.1617] | 0.7% | 0.4% |
| Yang B-2013 | 3 | 29 | 0.1034 | [0.0219; 0.2735] | 0.0% | 0.4% |
| Yin YZ-2001 | 11 | 150 | 0.0733 | [0.0372; 0.1274] | 0.2% | 0.4% |
| Yu DS-2011 | 29 | 277 | 0.1047 | [0.0712; 0.1469] | 0.3% | 0.4% |
| Zheng SJ-2015 | 8 | 23 | 0.3478 | [0.1638; 0.5727] | 0.0% | 0.4% |
| Zhong SQ-2007 | 67 | 131 | 0.5115 | [0.4226; 0.5997] | 0.2% | 0.4% |
| Zhu GZ-2007 | 37 | 396 | 0.0934 | [0.0666; 0.1265] | 0.5% | 0.4% |
| Zhou HF-2006 | 7 | 32 | 0.2188 | [0.0928; 0.3997] | 0.0% | 0.4% |
| **Common effect model** | | **13438** | **0.1265** | **[0.1208; 0.1323]** | **15.9%** | -- |
| **Random effects model** | | | **0.1906** | **[0.1375; 0.2499]** | -- | **15.5%** |
| Heterogeneity: $I^2$ = 98%, $\tau^2$ = 0.0447, $p$ < 0.01 | | | | | | |
| **Age = 30-39** | | | | | | |
| Ai X-2009 | 944 | 2224 | 0.4245 | [0.4038; 0.4453] | 2.6% | 0.4% |
| Cao HJ-2004 | 147 | 449 | 0.3274 | [0.2841; 0.3729] | 0.5% | 0.4% |
| Shen JY-2007 | 166 | 346 | 0.4798 | [0.4261; 0.5338] | 0.4% | 0.4% |
| Chen XM-2014 | 66 | 190 | 0.3474 | [0.2799; 0.4197] | 0.2% | 0.4% |
| Ning LF-2008 | 37 | 297 | 0.1246 | [0.0893; 0.1676] | 0.4% | 0.4% |
| Chen YZ-2006 | 58 | 261 | 0.2222 | [0.1733; 0.2776] | 0.3% | 0.4% |
| Gu HY-2013 | 138 | 1280 | 0.1078 | [0.0913; 0.1261] | 1.5% | 0.4% |
| Yao XF-2013 | 215 | 480 | 0.4479 | [0.4028; 0.4936] | 0.6% | 0.4% |
| Yao MF-2007 | 103 | 193 | 0.5337 | [0.4607; 0.6056] | 0.2% | 0.4% |
| Meng ZH-2005 | 90 | 186 | 0.4839 | [0.4101; 0.5581] | 0.2% | 0.4% |
| Kong DG-2017 | 237 | 547 | 0.4333 | [0.3913; 0.4760] | 0.6% | 0.4% |
| Li MY-2008 | 14 | 99 | 0.1414 | [0.0795; 0.2259] | 0.1% | 0.4% |
| Li WJ-2007 | 86 | 263 | 0.3270 | [0.2706; 0.3873] | 0.3% | 0.4% |
| Wang HR-2007 | 130 | 463 | 0.2808 | [0.2403; 0.3241] | 0.5% | 0.4% |
| Lin CY-2009 | 55 | 772 | 0.0712 | [0.0541; 0.0917] | 0.9% | 0.4% |
| Liu XG-2008 | 39 | 129 | 0.3023 | [0.2246; 0.3894] | 0.2% | 0.4% |
| Yu LM-2001 | 15 | 111 | 0.1351 | [0.0777; 0.2131] | 0.1% | 0.4% |
| Lu B-2008 | 80 | 157 | 0.5096 | [0.4286; 0.5901] | 0.2% | 0.4% |
| Lu YH-2006 | 74 | 115 | 0.6435 | [0.5488; 0.7306] | 0.1% | 0.4% |
| Luo YX-2005 | 57 | 600 | 0.0950 | [0.0727; 0.1213] | 0.7% | 0.4% |
| Sun Z-2014 | 105 | 223 | 0.4709 | [0.4039; 0.5386] | 0.3% | 0.4% |
| Tang WF-2014 | 11 | 31 | 0.3548 | [0.1923; 0.5463] | 0.0% | 0.4% |
| Tian JS-2007 | 8 | 93 | 0.0860 | [0.0379; 0.1625] | 0.1% | 0.4% |
| Wang FY-1999 | 4 | 131 | 0.0305 | [0.0084; 0.0763] | 0.2% | 0.4% |
| Wang ZZ-2007 | 169 | 181 | 0.9337 | [0.8871; 0.9653] | 0.2% | 0.4% |
| Wang RL-2012 | 101 | 324 | 0.3117 | [0.2617; 0.3653] | 0.4% | 0.4% |
| Wu JY-2017 | 63 | 198 | 0.3182 | [0.2540; 0.3880] | 0.2% | 0.4% |
| Xia XW-2015 | 236 | 681 | 0.3465 | [0.3108; 0.3836] | 0.8% | 0.4% |
| Xing XM-2011 | 58 | 199 | 0.2915 | [0.2293; 0.3599] | 0.2% | 0.4% |
| Yang F-2012 | 245 | 721 | 0.3398 | [0.3053; 0.3757] | 0.9% | 0.4% |
| Yang B-2013 | 3 | 67 | 0.0448 | [0.0093; 0.1253] | 0.1% | 0.4% |
| Yin YZ-2001 | 23 | 166 | 0.1386 | [0.0899; 0.2006] | 0.2% | 0.4% |
| Yu DS-2011 | 63 | 357 | 0.1765 | [0.1384; 0.2201] | 0.4% | 0.4% |
| Zheng SJ-2015 | 14 | 34 | 0.4118 | [0.2465; 0.5930] | 0.0% | 0.4% |
| Zhong SQ-2007 | 128 | 173 | 0.7399 | [0.6678; 0.8035] | 0.2% | 0.4% |
| Zhu GZ-2007 | 116 | 753 | 0.1541 | [0.1290; 0.1818] | 0.9% | 0.4% |
| Zhou HF-2006 | 9 | 32 | 0.2812 | [0.1375; 0.4675] | 0.0% | 0.4% |
| **Common effect model** | | **13526** | **0.2886** | **[0.2810; 0.2963]** | **16.0%** | -- |
| **Random effects model** | | | **0.3060** | **[0.2388; 0.3777]** | -- | **16.1%** |
| Heterogeneity: $I^2$ = 98%, $\tau^2$ = 0.0526, $p$ = 0 | | | | | | |
| **Age = 40-49** | | | | | | |
| Ai X-2009 | 1685 | 3754 | 0.4489 | [0.4329; 0.4649] | 4.4% | 0.4% |
| Cao HJ-2004 | 178 | 477 | 0.3732 | [0.3296; 0.4183] | 0.6% | 0.4% |
| Shen JY-2007 | 239 | 341 | 0.7009 | [0.6492; 0.7490] | 0.4% | 0.4% |
| Chen XM-2014 | 82 | 196 | 0.4184 | [0.3485; 0.4908] | 0.2% | 0.4% |
| Ning LF-2008 | 95 | 352 | 0.2699 | [0.2242; 0.3195] | 0.4% | 0.4% |
| Chen YZ-2006 | 76 | 281 | 0.2705 | [0.2194; 0.3264] | 0.3% | 0.4% |
| Gu HY-2013 | 110 | 631 | 0.1743 | [0.1455; 0.2062] | 0.7% | 0.4% |
| Yao XF-2013 | 258 | 460 | 0.5609 | [0.5142; 0.6068] | 0.5% | 0.4% |
| Yao MF-2007 | 115 | 199 | 0.5779 | [0.5060; 0.6474] | 0.2% | 0.4% |
| Meng ZH-2005 | 86 | 155 | 0.5548 | [0.4730; 0.6346] | 0.2% | 0.4% |
| Kong DG-2017 | 240 | 490 | 0.4898 | [0.4447; 0.5350] | 0.6% | 0.4% |
| Li MY-2008 | 30 | 140 | 0.2143 | [0.1495; 0.2916] | 0.2% | 0.4% |
| Li WJ-2007 | 93 | 236 | 0.3941 | [0.3313; 0.4595] | 0.3% | 0.4% |
| Wang HR-2007 | 81 | 252 | 0.3214 | [0.2642; 0.3829] | 0.3% | 0.4% |
| Lin CY-2009 | 111 | 1038 | 0.1069 | [0.0888; 0.1273] | 1.2% | 0.4% |
| Liu XG-2008 | 44 | 107 | 0.4112 | [0.3170; 0.5105] | 0.1% | 0.4% |
| Lu B-2008 | 104 | 189 | 0.5503 | [0.4764; 0.6225] | 0.2% | 0.4% |
| Lu YH-2006 | 125 | 183 | 0.6831 | [0.6103; 0.7497] | 0.2% | 0.4% |
| Luo YX-2005 | 43 | 329 | 0.1307 | [0.0962; 0.1720] | 0.4% | 0.4% |
| Sun Z-2014 | 107 | 229 | 0.4672 | [0.4012; 0.5341] | 0.3% | 0.4% |
| Tang WF-2014 | 22 | 52 | 0.4231 | [0.2873; 0.5680] | 0.1% | 0.4% |
| Tian JS-2007 | 8 | 102 | 0.0784 | [0.0345; 0.1487] | 0.1% | 0.4% |
| Wang FY-1999 | 3 | 65 | 0.0462 | [0.0096; 0.1290] | 0.1% | 0.4% |
| Wang ZZ-2007 | 194 | 204 | 0.9510 | [0.9117; 0.9762] | 0.2% | 0.4% |
| Wang RL-2012 | 105 | 337 | 0.3116 | [0.2625; 0.3640] | 0.4% | 0.4% |
| Wu JY-2017 | 76 | 220 | 0.3455 | [0.2828; 0.4123] | 0.3% | 0.4% |
| Xia XW-2015 | 247 | 578 | 0.4273 | [0.3866; 0.4688] | 0.7% | 0.4% |
| Xing XM-2011 | 58 | 191 | 0.3037 | [0.2394; 0.3742] | 0.2% | 0.4% |
| Yang F-2012 | 218 | 518 | 0.4208 | [0.3779; 0.4647] | 0.6% | 0.4% |
| Yang B-2013 | 15 | 76 | 0.1974 | [0.1149; 0.3046] | 0.1% | 0.4% |
| Yin YZ-2001 | 8 | 84 | 0.0952 | [0.0420; 0.1791] | 0.1% | 0.4% |
| Yu DS-2011 | 72 | 316 | 0.2278 | [0.1828; 0.2781] | 0.4% | 0.4% |
| Zheng SJ-2015 | 21 | 57 | 0.3684 | [0.2445; 0.5066] | 0.1% | 0.4% |
| Zhong SQ-2007 | 161 | 204 | 0.7892 | [0.7268; 0.8431] | 0.2% | 0.4% |
| Zhu GZ-2007 | 220 | 956 | 0.2301 | [0.2038; 0.2581] | 1.1% | 0.4% |
| Zhou HF-2006 | 13 | 28 | 0.4643 | [0.2751; 0.6613] | 0.0% | 0.4% |
| **Common effect model** | | **14027** | **0.3732** | **[0.3651; 0.3812]** | **16.6%** | -- |
| **Random effects model** | | | **0.3755** | **[0.3033; 0.4504]** | -- | **15.6%** |
| Heterogeneity: $I^2$ = 98%, $\tau^2$ = 0.0522, $p$ = 0 | | | | | | |

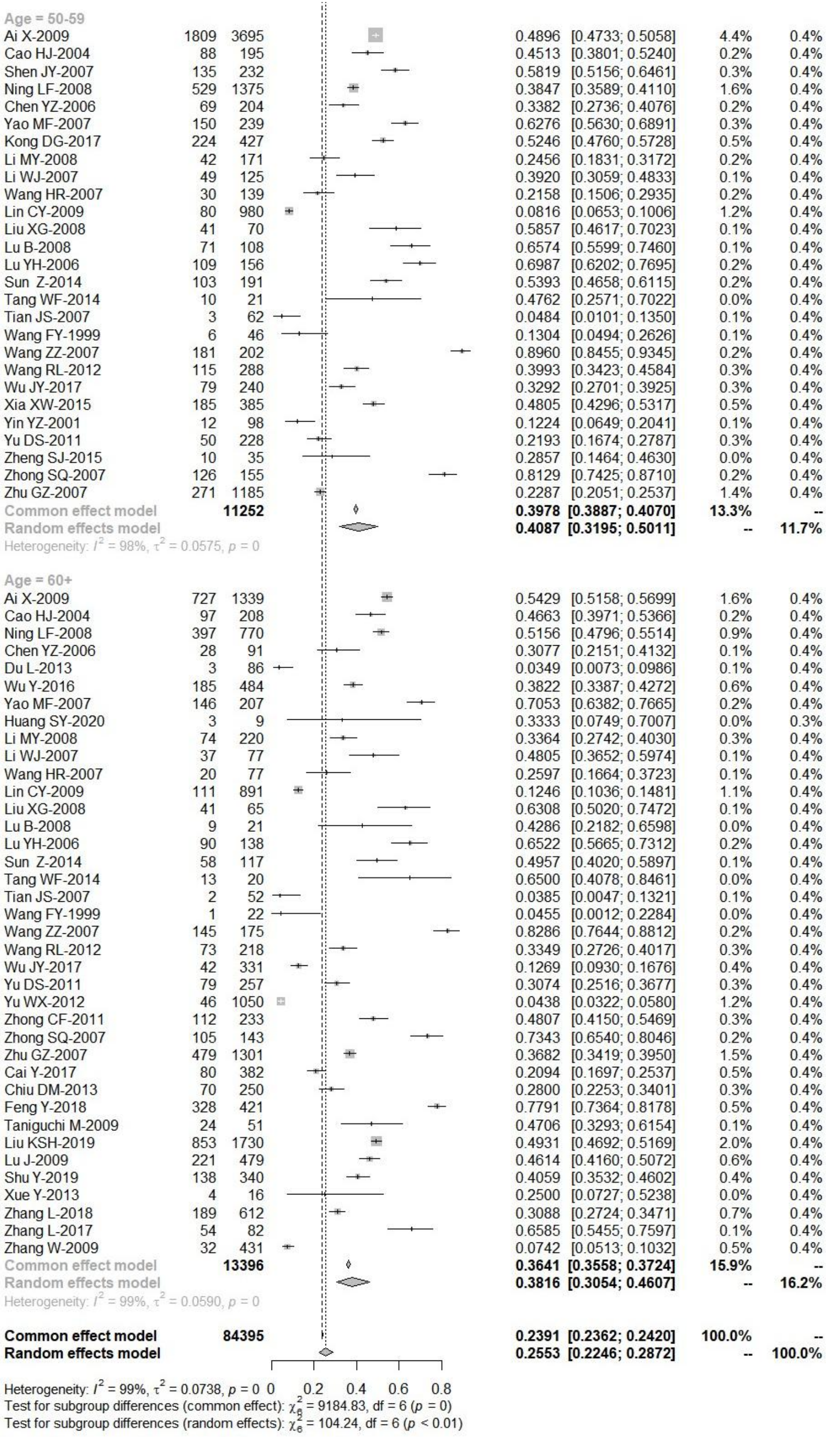


Figure S15. Forest plot of anti-HEV IgG prevalence by age in the general population

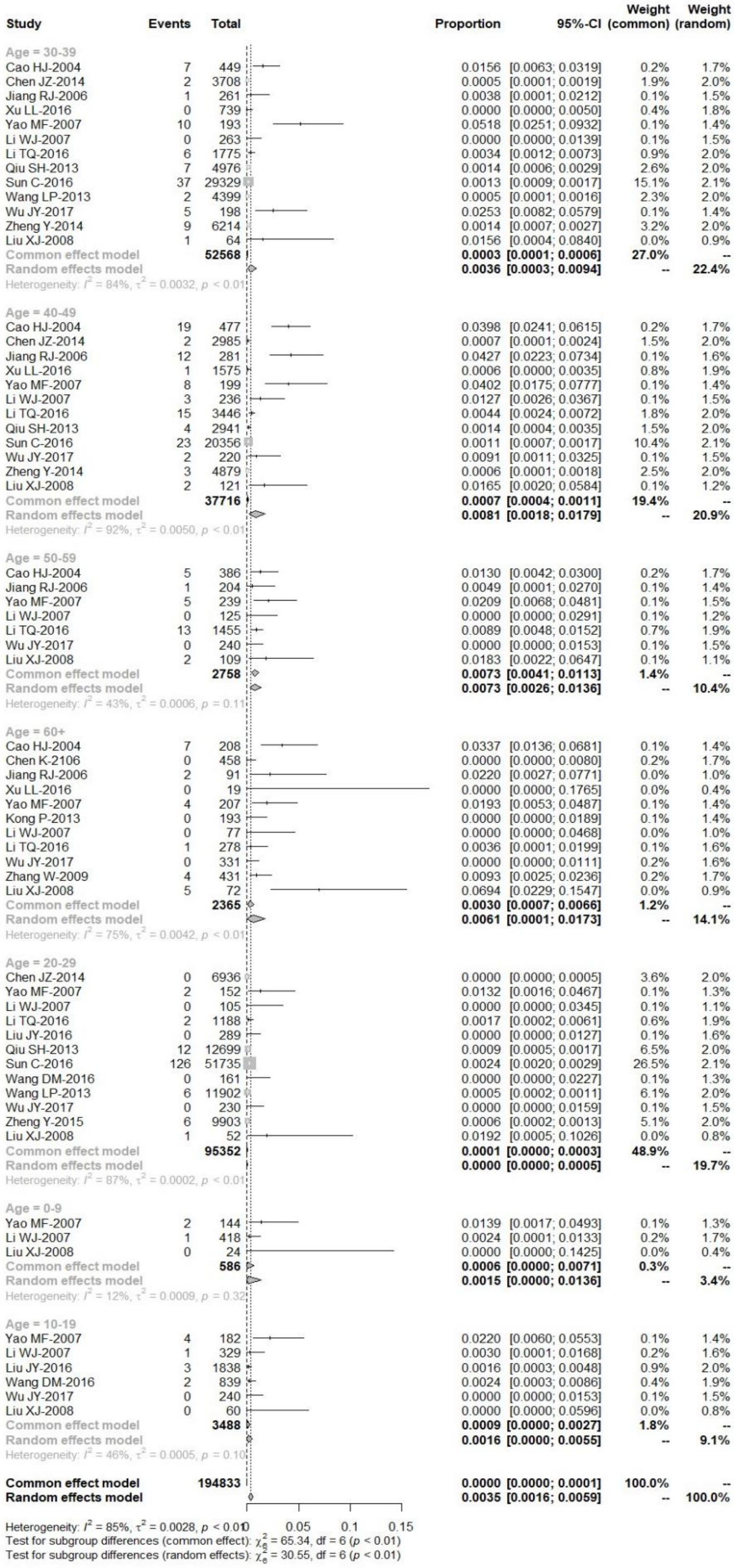


Figure S16. Forest plot of anti-HEV IgM prevalence by age in the general population

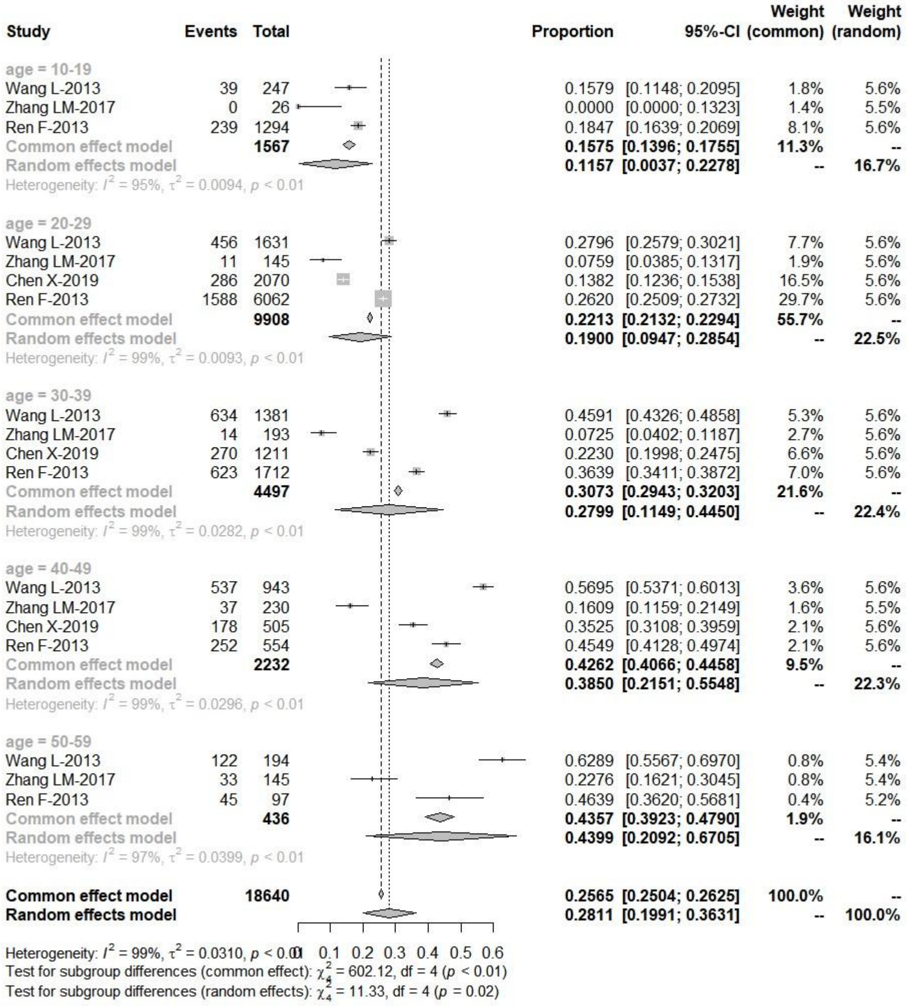


Figure S17. Forest plot of anti-HEV IgG prevalence by age in volunteer blood donors

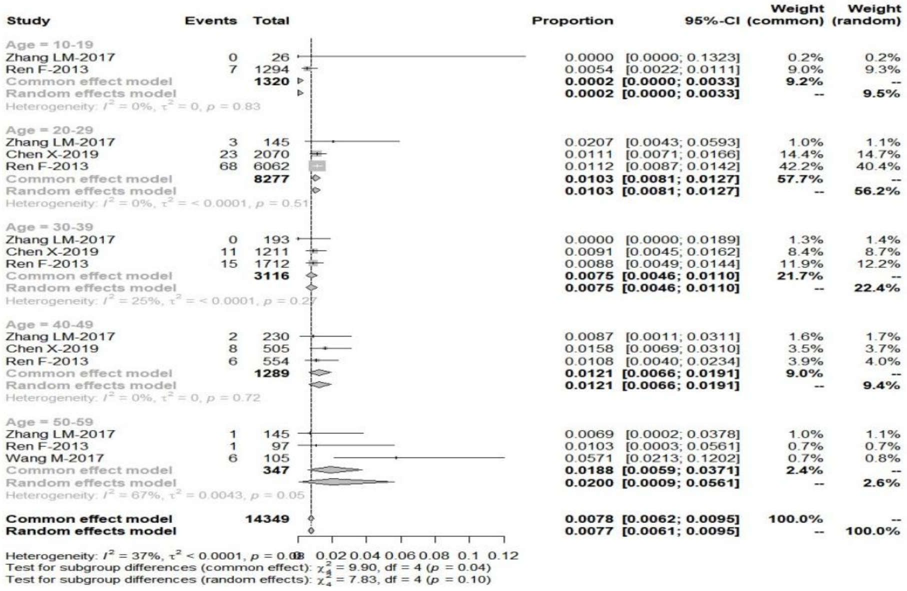


Figure S18. Forest plot of anti-HEV IgM prevalence by age in volunteer blood donors

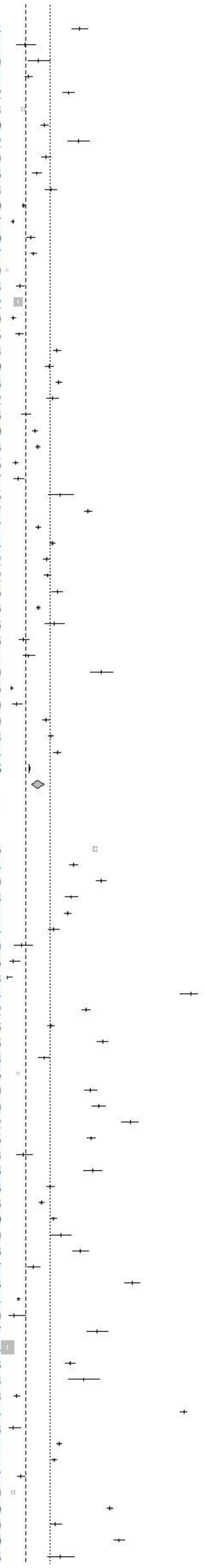

| Study | Events | Total | Proportion | 95%-CI | Weight (common) | Weight (random) |
|---|---|---|---|---|---|---|
| **region_1 = The North** | | | | | | |
| Zhang ZX-2003 | 210 | 574 | 0.3659 | [0.3264; 0.4067] | 0.2% | 0.9% |
| AYiGuLi-2010 | 13 | 151 | 0.0861 | [0.0466; 0.1427] | 0.0% | 0.9% |
| Bao ZY-2013 | 28 | 180 | 0.1556 | [0.1059; 0.2169] | 0.1% | 0.9% |
| Bi L-2008 | 107 | 1001 | 0.1069 | [0.0884; 0.1277] | 0.3% | 0.9% |
| Du JY-2014 | 295 | 952 | 0.3099 | [0.2806; 0.3403] | 0.3% | 0.9% |
| Gu HY-2013 | 505 | 6258 | 0.0807 | [0.0741; 0.0877] | 1.8% | 0.9% |
| Wu JY-2016 | 287 | 1529 | 0.1877 | [0.1684; 0.2082] | 0.4% | 0.9% |
| Wu JY-2016 | 112 | 312 | 0.3590 | [0.3057; 0.4149] | 0.1% | 0.9% |
| Lu B-2008 | 208 | 1060 | 0.1962 | [0.1727; 0.2214] | 0.3% | 0.9% |
| Bo QN-2019 | 124 | 836 | 0.1483 | [0.1249; 0.1743] | 0.2% | 0.9% |
| Li MY-2008 | 169 | 768 | 0.2201 | [0.1912; 0.2510] | 0.2% | 0.9% |
| Lin CY-2009 | 403 | 4959 | 0.0813 | [0.0738; 0.0892] | 1.4% | 0.9% |
| Liu JY-2016 | 57 | 2127 | 0.0268 | [0.0204; 0.0346] | 0.6% | 0.9% |
| Liu K-2009 | 111 | 939 | 0.1182 | [0.0983; 0.1406] | 0.3% | 0.9% |
| Lu J-2009 | 259 | 1977 | 0.1310 | [0.1164; 0.1467] | 0.6% | 0.9% |
| Pan TJ-2002 | 0 | 1580 | 0.0000 | [0.0000; 0.0023] | 0.5% | 0.9% |
| Shao HW-2009 | 41 | 648 | 0.0633 | [0.0458; 0.0849] | 0.2% | 0.9% |
| Sun ZH-2017 | 2555 | 47852 | 0.0534 | [0.0514; 0.0554] | 13.9% | 0.9% |
| Wang DM-2016 | 28 | 1000 | 0.0280 | [0.0187; 0.0402] | 0.3% | 0.9% |
| Wang FY-1999 | 33 | 575 | 0.0574 | [0.0398; 0.0797] | 0.2% | 0.9% |
| Wang RL-2012 | 509 | 2028 | 0.2510 | [0.2322; 0.2705] | 0.6% | 0.9% |
| Wu JY-2017 | 310 | 1459 | 0.2125 | [0.1917; 0.2344] | 0.4% | 0.9% |
| Xia XW-2015 | 914 | 3513 | 0.2602 | [0.2457; 0.2750] | 1.0% | 0.9% |
| Xing XM-2011 | 185 | 812 | 0.2278 | [0.1994; 0.2583] | 0.2% | 0.9% |
| Yin YZ-2001 | 63 | 676 | 0.0932 | [0.0724; 0.1177] | 0.2% | 0.9% |
| Yu DS-2011 | 338 | 2429 | 0.1392 | [0.1256; 0.1536] | 0.7% | 0.9% |
| Zhang D-2022 | 1000 | 6493 | 0.1540 | [0.1453; 0.1630] | 1.9% | 0.9% |
| Zhang P-2015 | 47 | 1195 | 0.0393 | [0.0290; 0.0520] | 0.3% | 0.9% |
| Zhao HL-2012 | 17 | 327 | 0.0520 | [0.0306; 0.0819] | 0.1% | 0.9% |
| Zheng SJ-2015 | 55 | 205 | 0.2683 | [0.2090; 0.3345] | 0.1% | 0.9% |
| Zhong CF-2011 | 1074 | 2627 | 0.4088 | [0.3900; 0.4279] | 0.8% | 0.9% |
| Zhu GZ-2015 | 458 | 2957 | 0.1549 | [0.1420; 0.1684] | 0.9% | 0.9% |
| Zhu GZ-2007 | 1127 | 4944 | 0.2280 | [0.2163; 0.2399] | 1.4% | 0.9% |
| Cai Y-2017 | 366 | 1842 | 0.1987 | [0.1807; 0.2177] | 0.5% | 0.9% |
| Chang Y-2009 | 522 | 2572 | 0.2030 | [0.1876; 0.2190] | 0.7% | 0.9% |
| Cong W-2014 | 244 | 965 | 0.2528 | [0.2257; 0.2815] | 0.3% | 0.9% |
| Dong C-2012 | 1019 | 6573 | 0.1550 | [0.1464; 0.1640] | 1.9% | 0.9% |
| Fu H-2010 | 70 | 296 | 0.2365 | [0.1892; 0.2891] | 0.1% | 0.9% |
| Shenyang G-2011 | 37 | 456 | 0.0811 | [0.0578; 0.1101] | 0.1% | 0.9% |
| Geng Y-2019 | 45 | 421 | 0.1069 | [0.0790; 0.1404] | 0.1% | 0.9% |
| Taniguchi M-2009 | 143 | 300 | 0.4767 | [0.4190; 0.5348] | 0.1% | 0.9% |
| Li H-2021 | 43 | 2005 | 0.0214 | [0.0156; 0.0288] | 0.6% | 0.9% |
| Ma XX-2021 | 13 | 290 | 0.0448 | [0.0241; 0.0754] | 0.1% | 0.9% |
| Ma Z-2010 | 407 | 2090 | 0.1947 | [0.1780; 0.2124] | 0.6% | 0.9% |
| Yu Y-2009 | 988 | 4508 | 0.2192 | [0.2072; 0.2315] | 1.3% | 0.9% |
| Zhang L-2018 | 519 | 2054 | 0.2527 | [0.2340; 0.2721] | 0.6% | 0.9% |
| **Common effect model** | | **129315** | **0.1106** | **[0.1089; 0.1124]** | **37.5%** | **--** |
| **Random effects model** | | | **0.1511** | **[0.1202; 0.1848]** | **--** | **42.7%** |
| Heterogeneity: $I^2 = 100\%$, $\tau^2 = 0.0239$, $p = 0$ | | | | | | |
| **region_1 = The South** | | | | | | |
| Ai X-2009 | 5588 | 12555 | 0.4451 | [0.4364; 0.4538] | 3.6% | 0.9% |
| Cao HJ-2004 | 648 | 1934 | 0.3351 | [0.3140; 0.3566] | 0.6% | 0.9% |
| Shen JY-2007 | 749 | 1570 | 0.4771 | [0.4521; 0.5021] | 0.5% | 0.9% |
| Chen XM-2014 | 281 | 868 | 0.3237 | [0.2927; 0.3560] | 0.3% | 0.9% |
| Ning LF-2008 | 1086 | 3561 | 0.3050 | [0.2899; 0.3204] | 1.0% | 0.9% |
| Chen YZ-2006 | 254 | 1084 | 0.2343 | [0.2094; 0.2607] | 0.3% | 0.9% |
| Cheng Y-2007 | 10 | 140 | 0.0714 | [0.0348; 0.1274] | 0.0% | 0.9% |
| Du L-2013 | 7 | 235 | 0.0298 | [0.0121; 0.0604] | 0.1% | 0.9% |
| Fan LZ-2012 | 0 | 158 | 0.0000 | [0.0000; 0.0231] | 0.0% | 0.9% |
| Gong YH-2005 | 134 | 144 | 0.9306 | [0.8760; 0.9662] | 0.0% | 0.9% |
| Yao XF-2013 | 806 | 2012 | 0.4006 | [0.3791; 0.4224] | 0.6% | 0.9% |
| Wu Y-2016 | 482 | 2206 | 0.2185 | [0.2014; 0.2363] | 0.6% | 0.9% |
| Yao MF-2007 | 635 | 1316 | 0.4825 | [0.4552; 0.5099] | 0.4% | 0.9% |
| Huang SY-2020 | 120 | 648 | 0.1852 | [0.1560; 0.2173] | 0.2% | 0.9% |
| Huang SM-2017 | 273 | 5345 | 0.0511 | [0.0453; 0.0573] | 1.6% | 0.9% |
| Meng ZH-2005 | 413 | 980 | 0.4214 | [0.3903; 0.4531] | 0.3% | 0.9% |
| Wang FD-2004 | 394 | 850 | 0.4635 | [0.4296; 0.4977] | 0.2% | 0.9% |
| Zheng YJ-2005 | 319 | 512 | 0.6230 | [0.5795; 0.6652] | 0.1% | 0.9% |
| Kong DG-2017 | 825 | 1945 | 0.4242 | [0.4021; 0.4465] | 0.6% | 0.9% |
| Li B-2003 | 14 | 178 | 0.0787 | [0.0437; 0.1284] | 0.1% | 0.9% |
| Li JT-2014 | 197 | 456 | 0.4320 | [0.3860; 0.4789] | 0.1% | 0.9% |
| Li WJ-2007 | 337 | 1553 | 0.2170 | [0.1967; 0.2383] | 0.5% | 0.9% |
| Li YB-2004 | 572 | 3336 | 0.1715 | [0.1588; 0.1847] | 1.0% | 0.9% |
| Wang HR-2007 | 515 | 2209 | 0.2331 | [0.2156; 0.2513] | 0.6% | 0.9% |
| Liu XG-2007 | 81 | 300 | 0.2700 | [0.2206; 0.3240] | 0.1% | 0.9% |
| Liu XG-2008 | 209 | 566 | 0.3693 | [0.3294; 0.4105] | 0.2% | 0.9% |
| Yu LM-2001 | 54 | 417 | 0.1295 | [0.0988; 0.1656] | 0.1% | 0.9% |
| Lu YH-2006 | 421 | 663 | 0.6350 | [0.5971; 0.6717] | 0.2% | 0.9% |
| Luo YX-2005 | 215 | 3864 | 0.0556 | [0.0486; 0.0633] | 1.1% | 0.9% |
| Ma TW-2013 | 3 | 90 | 0.0333 | [0.0069; 0.0943] | 0.0% | 0.9% |
| Nong CS-2007 | 172 | 377 | 0.4562 | [0.4051; 0.5080] | 0.1% | 0.9% |
| Pan YL-2021 | 0 | 103794 | 0.0000 | [0.0000; 0.0000] | 30.1% | 0.9% |
| Sun Z-2014 | 471 | 1483 | 0.3176 | [0.2939; 0.3420] | 0.4% | 0.9% |
| Tang WF-2014 | 61 | 158 | 0.3861 | [0.3098; 0.4667] | 0.0% | 0.9% |
| Tian JS-2007 | 35 | 778 | 0.0450 | [0.0315; 0.0620] | 0.2% | 0.9% |
| Wang ZZ-2007 | 1105 | 1234 | 0.8955 | [0.8770; 0.9120] | 0.4% | 0.9% |
| Wu CH-2003 | 4 | 148 | 0.0270 | [0.0074; 0.0678] | 0.0% | 0.9% |
| Xiao ZB-2022 | 1217 | 4661 | 0.2611 | [0.2485; 0.2740] | 1.4% | 0.9% |
| Yang F-2012 | 893 | 3771 | 0.2368 | [0.2233; 0.2507] | 1.1% | 0.9% |
| Yang B-2013 | 40 | 597 | 0.0670 | [0.0483; 0.0901] | 0.2% | 0.9% |
| Yu WX-2012 | 138 | 5000 | 0.0276 | [0.0232; 0.0325] | 1.5% | 0.9% |
| Zhang XF-2007 | 2152 | 4139 | 0.5199 | [0.5046; 0.5353] | 1.2% | 0.9% |
| Zheng RD-2013 | 207 | 850 | 0.2435 | [0.2150; 0.2738] | 0.2% | 0.9% |
| Zhong SQ-2007 | 702 | 1239 | 0.5666 | [0.5385; 0.5944] | 0.4% | 0.9% |
| Zhou HF-2006 | 47 | 175 | 0.2686 | [0.2045; 0.3407] | 0.1% | 0.9% |

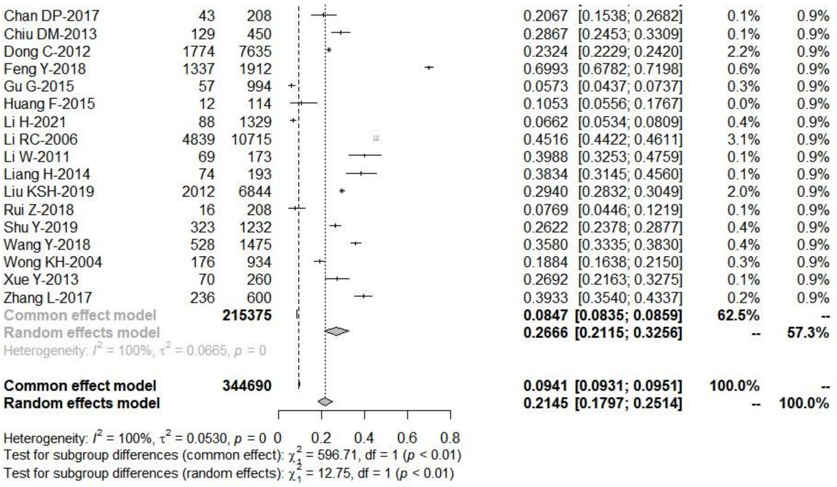


Figure S19. Forest plot of anti-HEV IgG prevalence in the general population in the North and South regions

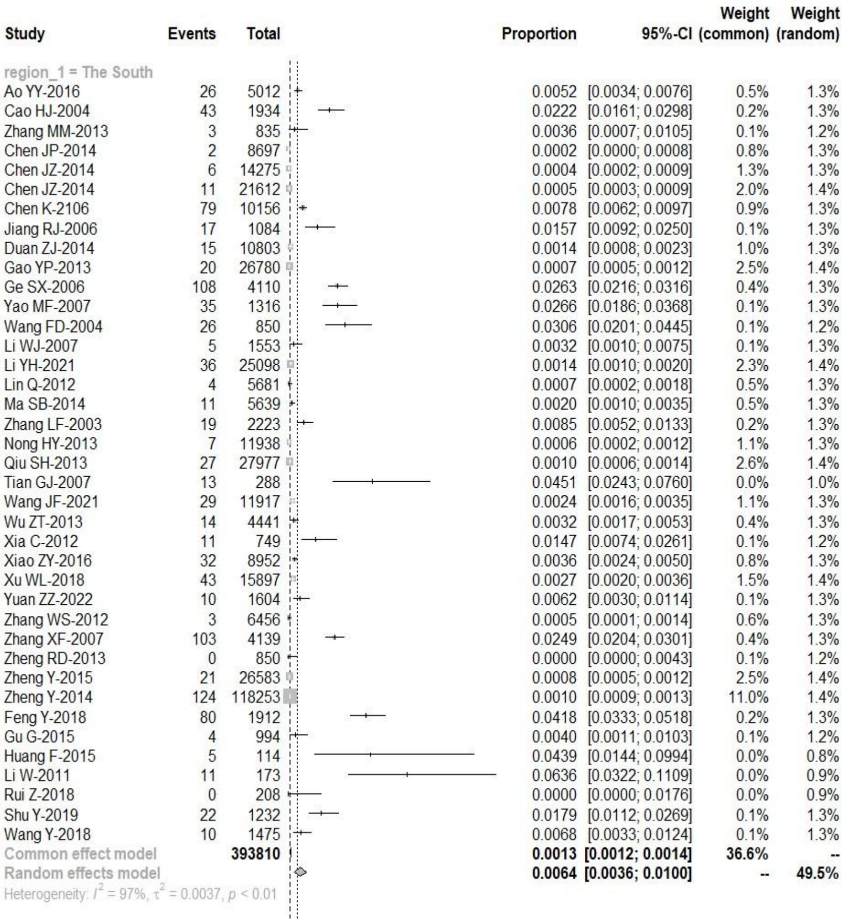

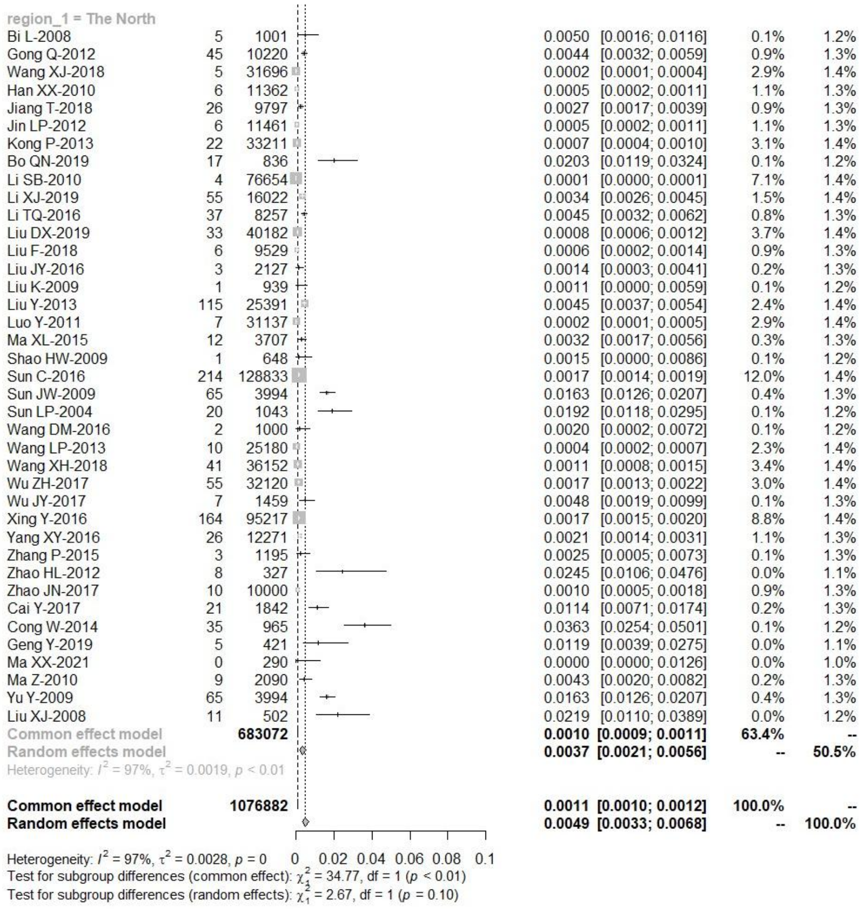


Figure S20. Forest plot of anti-HEV IgM prevalence in the general population in the North and South regions

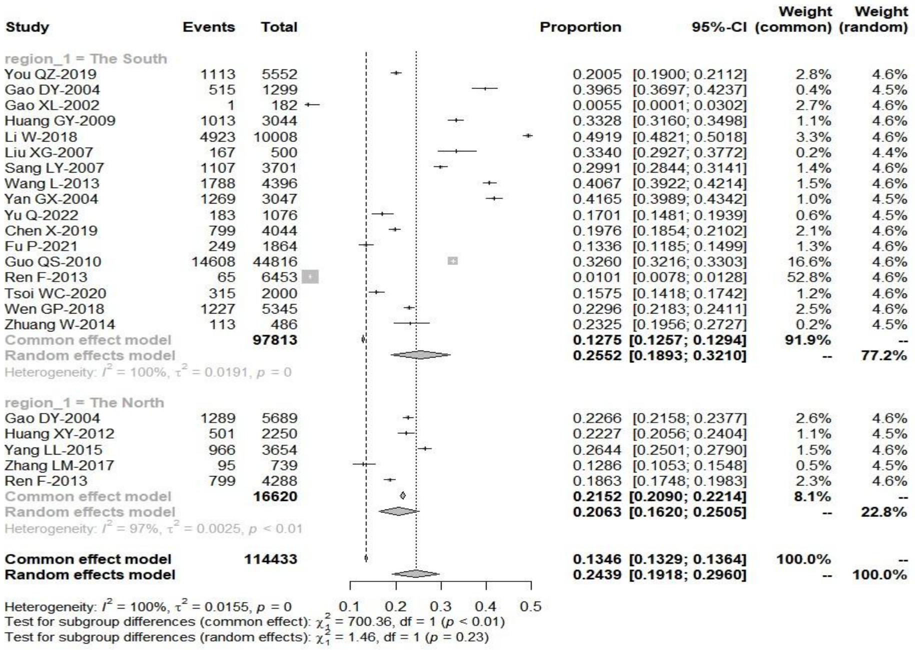


Figure S21. Forest plot of anti-HEV IgG prevalence in volunteer blood donors in the North and South regions

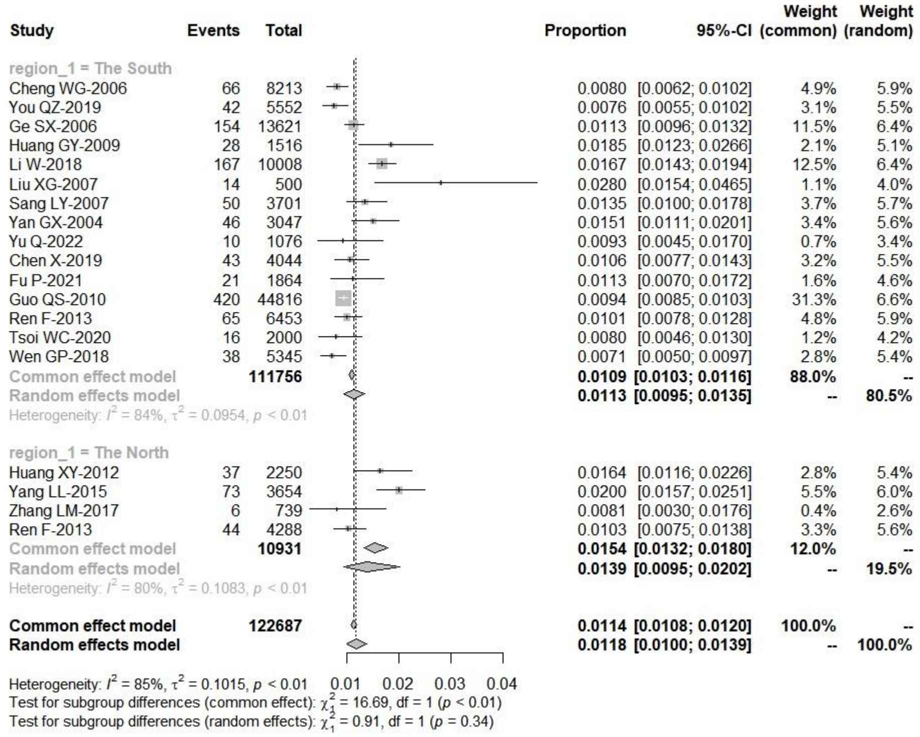


Figure S22. Forest plot of anti-HEV IgM prevalence in volunteer blood donors in the North and South regions

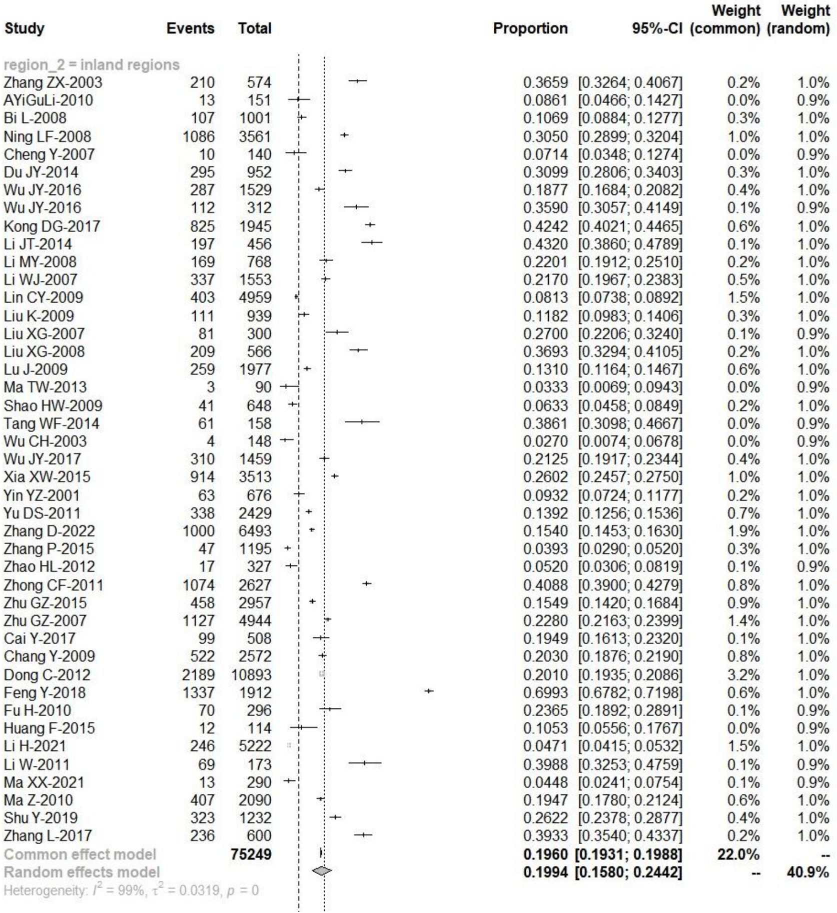

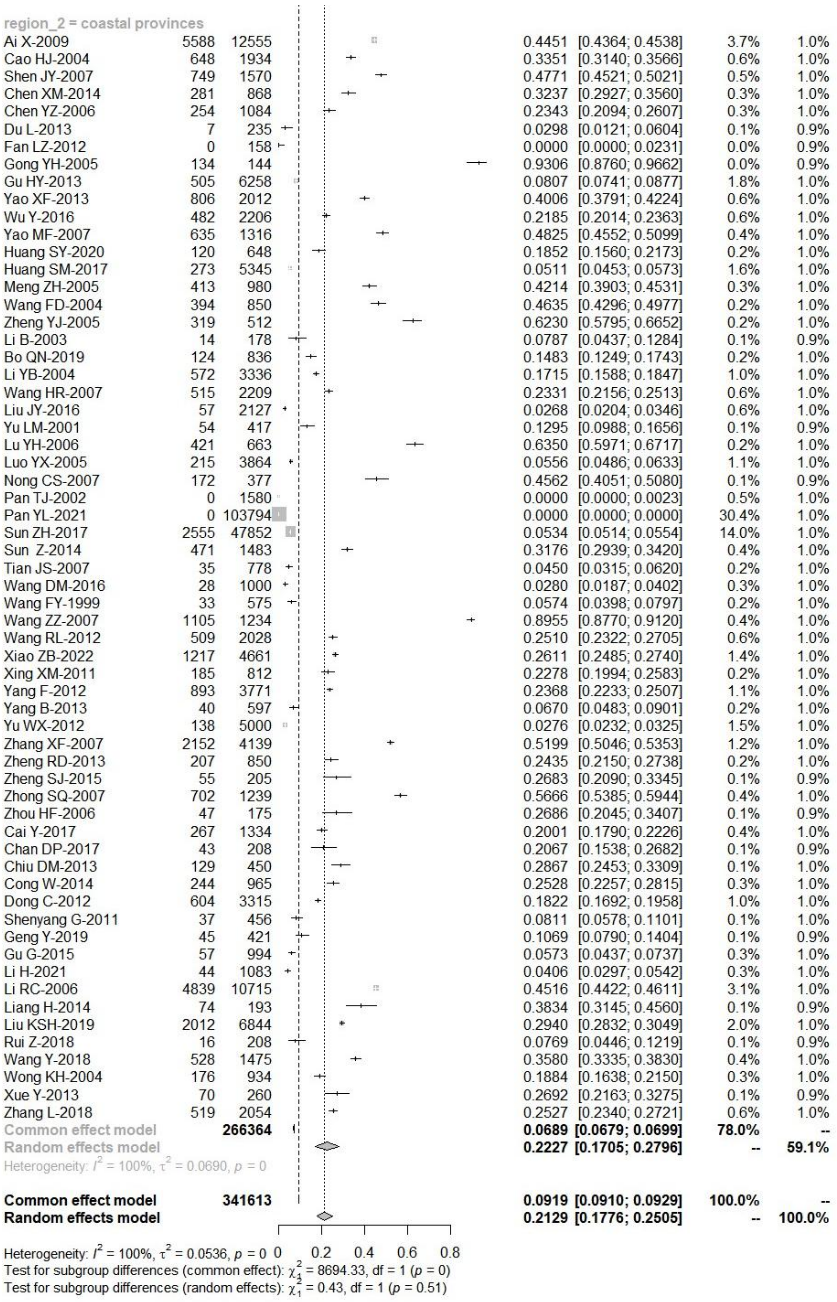


Figure S23. Forest plot of anti-HEV IgG prevalence in the general population in the coastal and inland areas

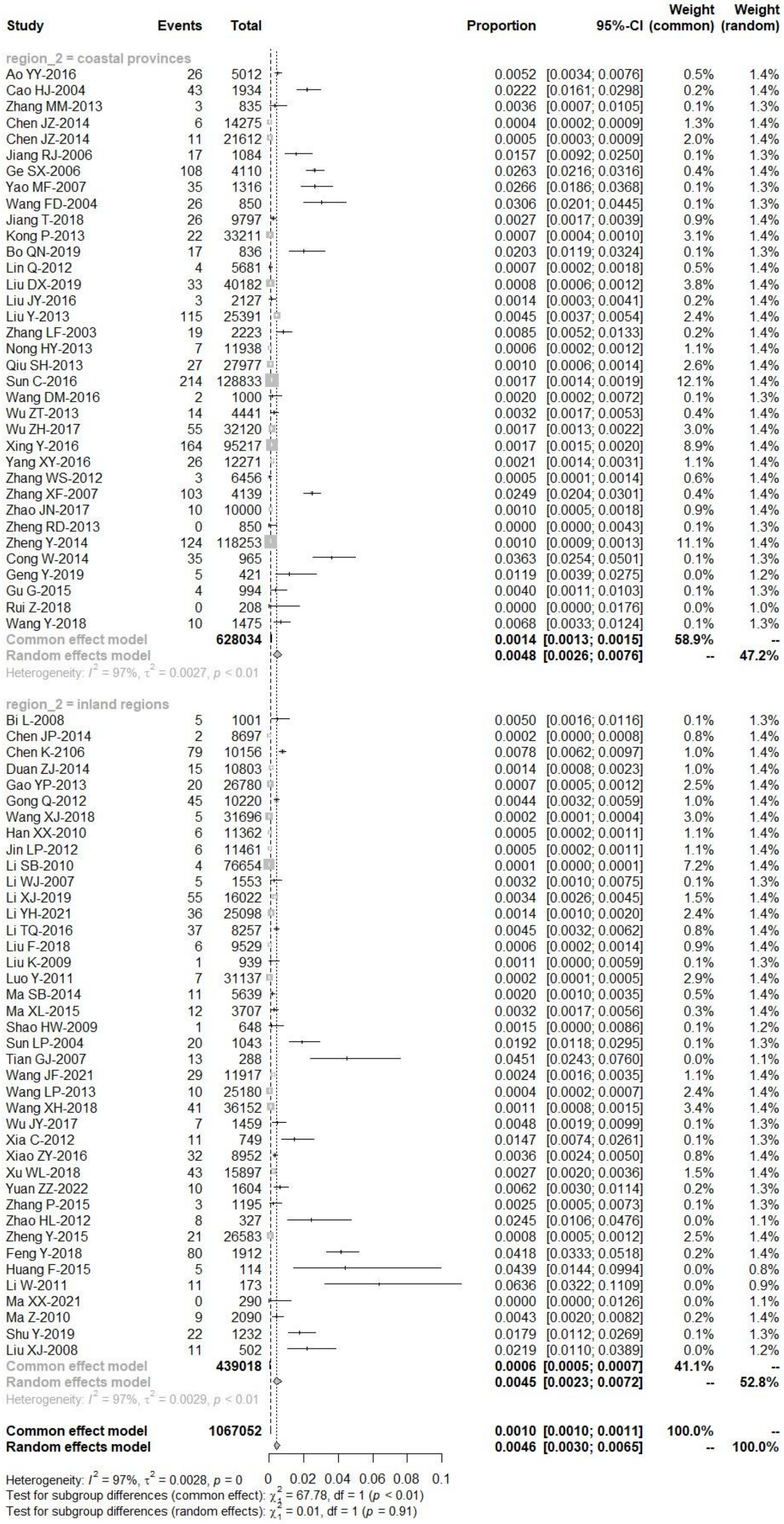


Figure S24. Forest plot of anti-HEV IgM prevalence in the general population in the coastal and inland areas

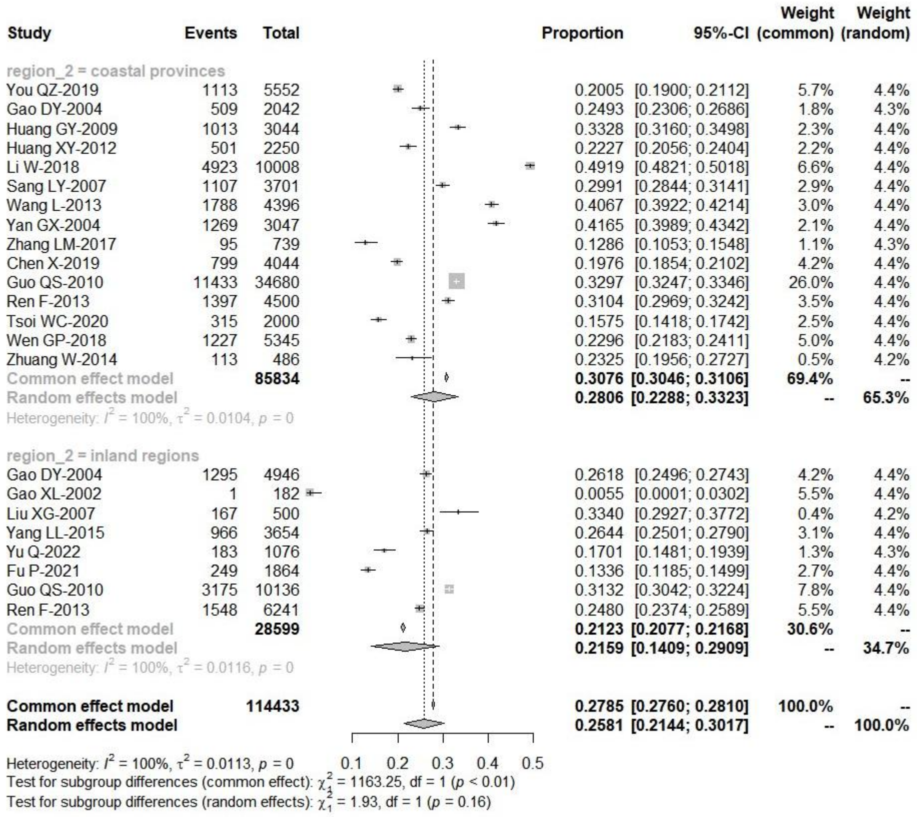


Figure S25. Forest plot of anti-HEV IgG prevalence in volunteer blood donors in the coastal and inland areas

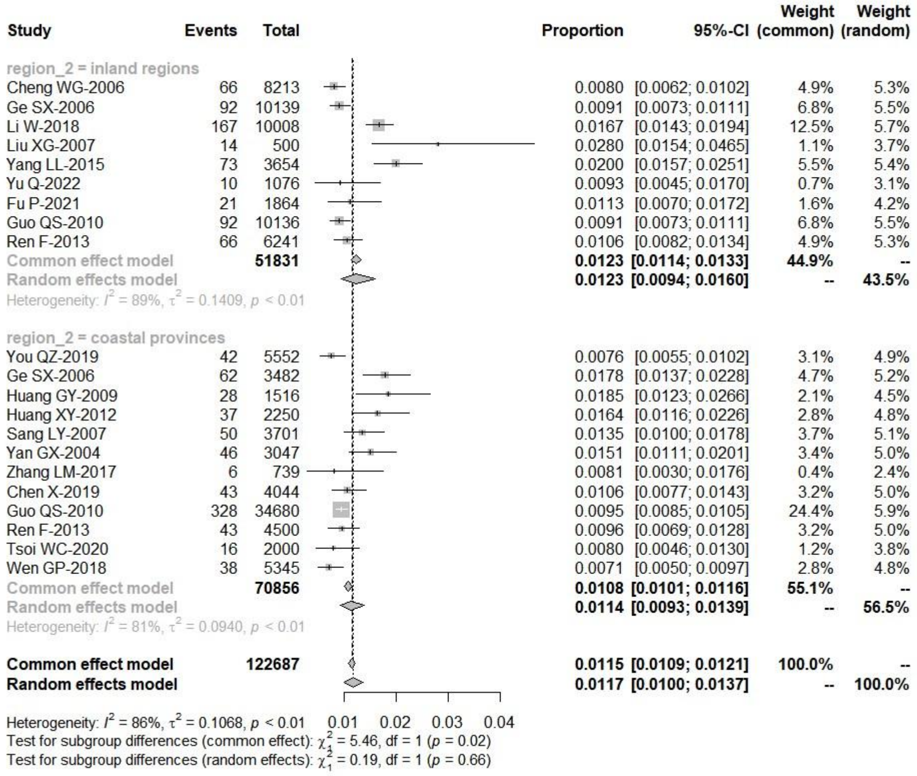


Figure S26. Forest plot of anti-HEV IgM prevalence in volunteer blood donors in the coastal and inland areas

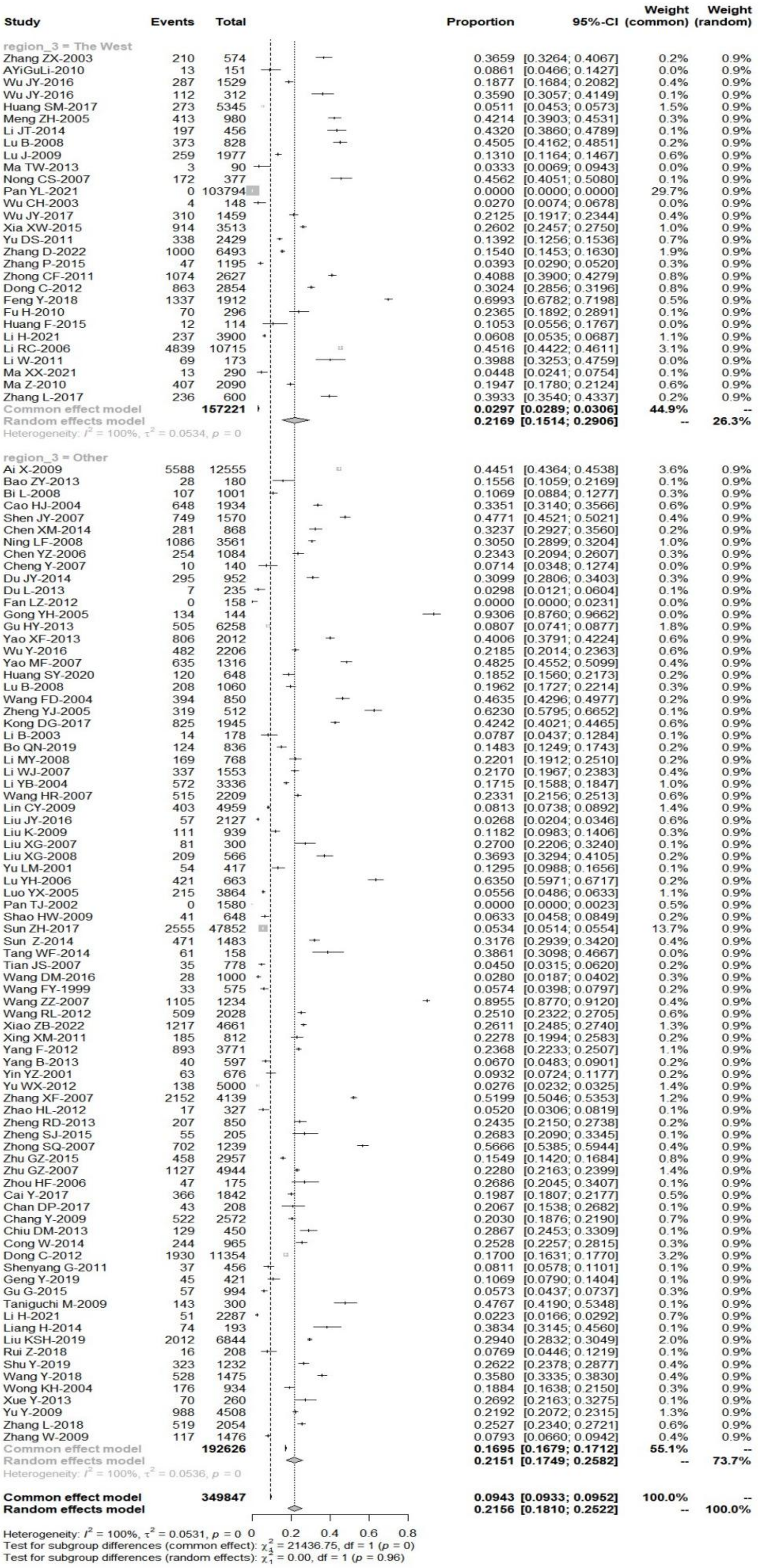


Figure S27. Forest plot of anti-HEV IgG prevalence in the general population in the western and non-western regions

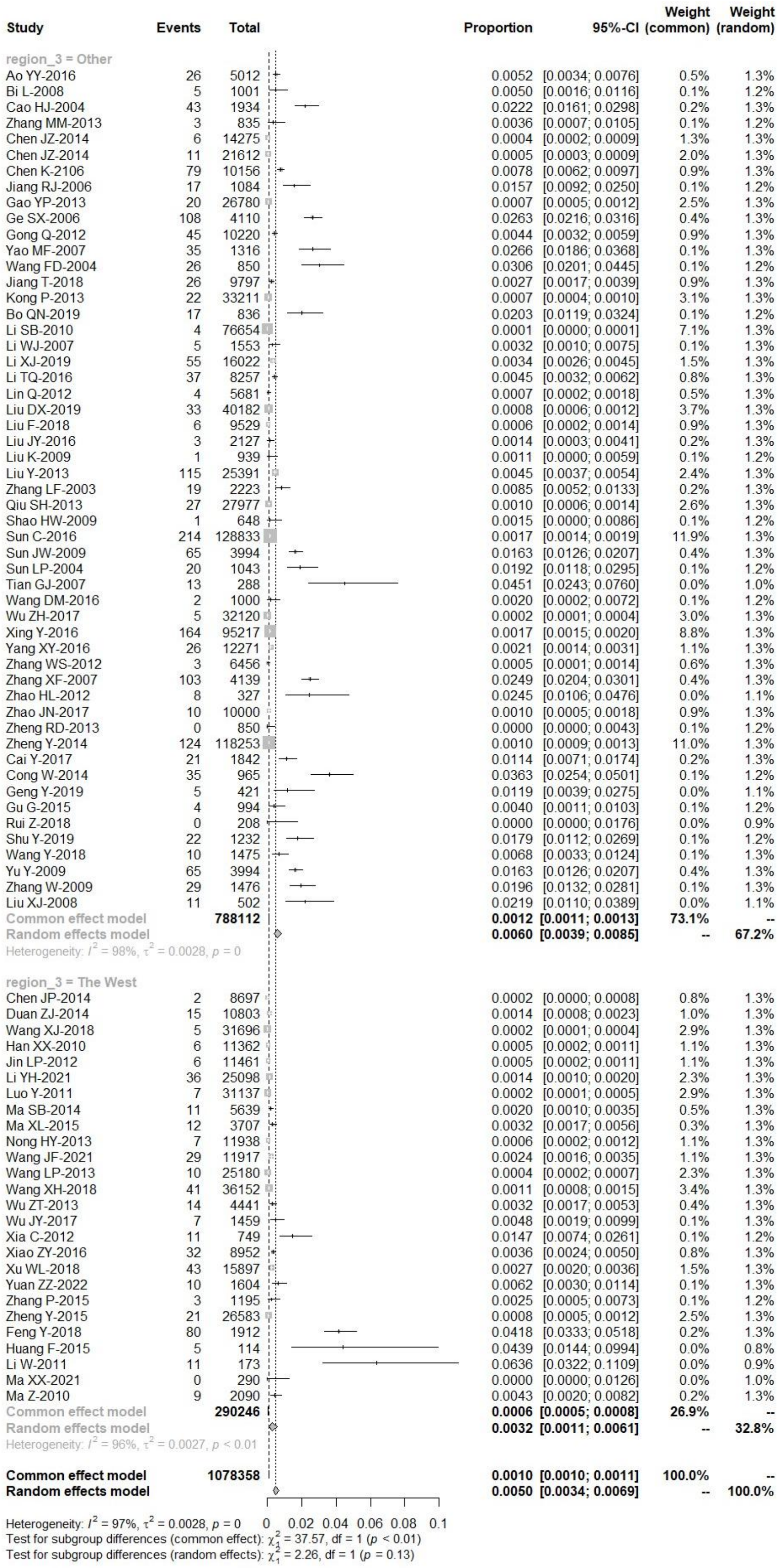


Figure S28. Forest plot of anti-HEV IgM prevalence in the general population in the western and non-western regions

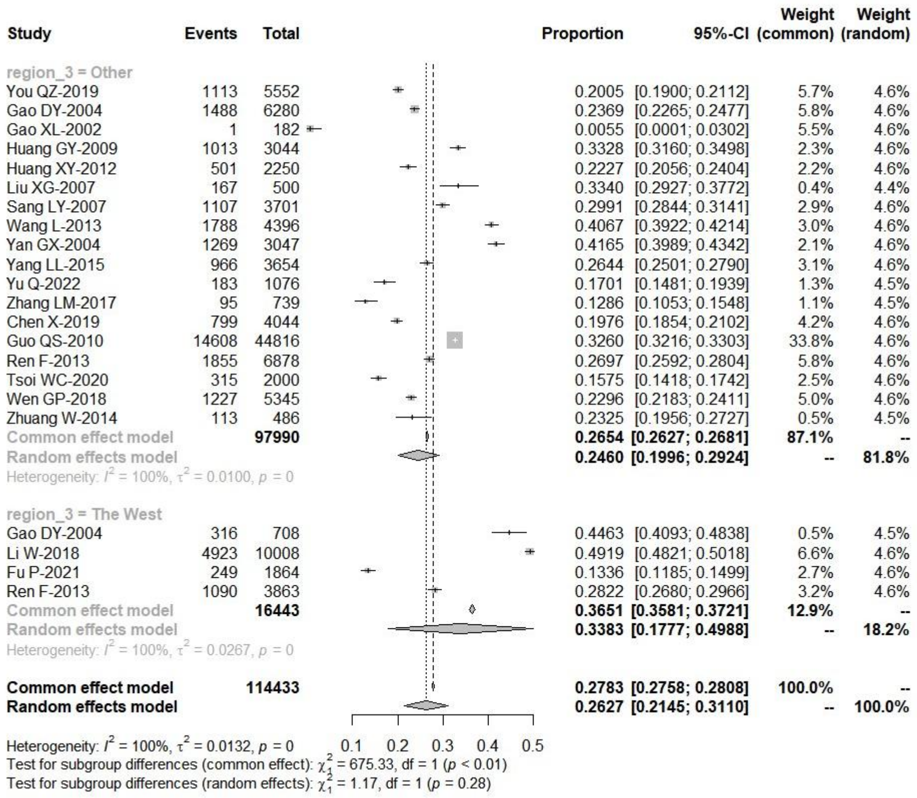


Figure S29. Forest plot of anti-HEV IgG prevalence in volunteer blood donors in the western and non-western regions

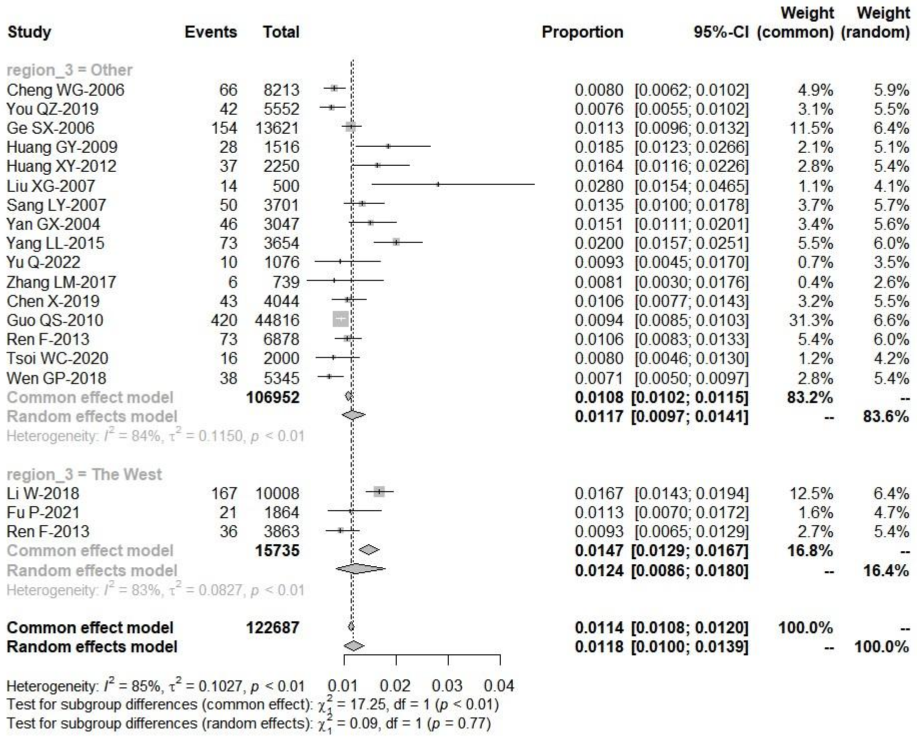


Figure S30. Forest plot of anti-HEV IgM prevalence in volunteer blood donors in the western and non-western regions

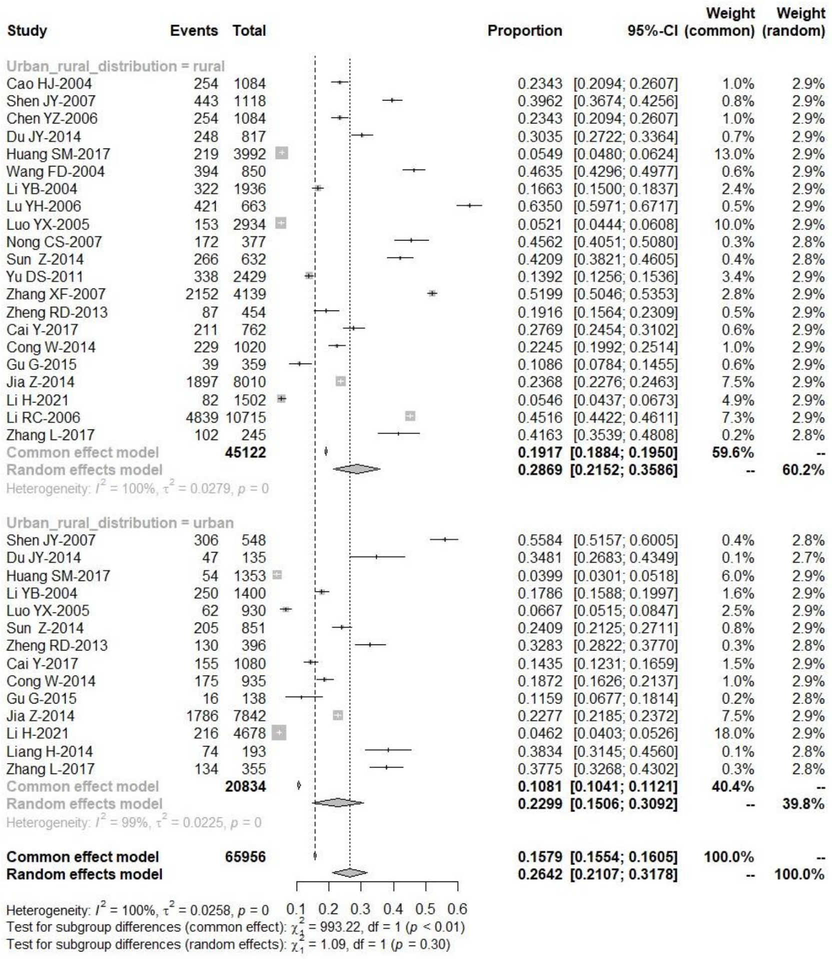


Figure S31. Forest plot of anti-HEV IgG prevalence in the general popualtion in the urban and rural areas

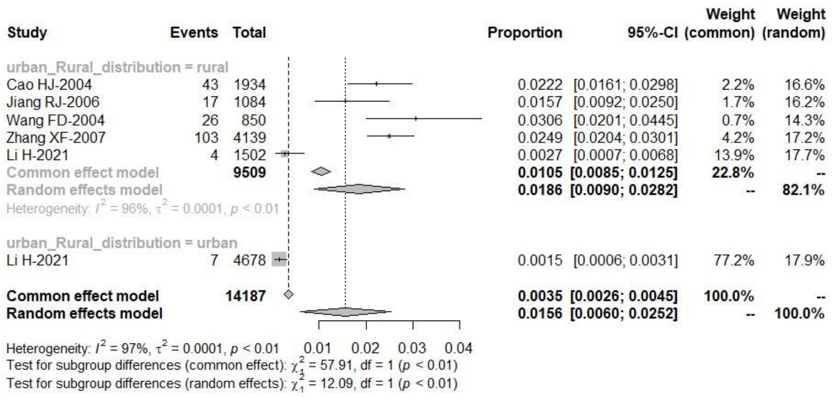


Figure S32. Forest plot of anti-HEV IgM prevalence in the general popualtion in the urban and rural areas

| Study | Events | Total | Proportion | 95%-CI | Weight (common) | Weight (random) |
|---|---|---|---|---|---|---|
| **Studyperiod = 2001-2005** | | | | | | |
| Zhang ZX-2003 | 210 | 574 | 0.3659 | [0.3264; 0.4067] | 0.1% | 0.9% |
| Ai X-2009 | 5588 | 12555 | 0.4451 | [0.4364; 0.4538] | 3.0% | 0.9% |
| Cao HJ-2004 | 648 | 1934 | 0.3351 | [0.3140; 0.3566] | 0.5% | 0.9% |
| Shen JY-2007 | 749 | 1570 | 0.4771 | [0.4521; 0.5021] | 0.4% | 0.9% |
| Chen YZ-2006 | 254 | 1084 | 0.2343 | [0.2094; 0.2607] | 0.3% | 0.9% |
| Gong YH-2005 | 134 | 144 | 0.9306 | [0.8760; 0.9662] | 0.0% | 0.9% |
| Yao MF-2007 | 635 | 1316 | 0.4825 | [0.4552; 0.5099] | 0.3% | 0.9% |
| Meng ZH-2005 | 413 | 980 | 0.4214 | [0.3903; 0.4531] | 0.2% | 0.9% |
| Wang FD-2004 | 394 | 850 | 0.4635 | [0.4296; 0.4977] | 0.2% | 0.9% |
| Zheng YJ-2005 | 319 | 512 | 0.6230 | [0.5795; 0.6652] | 0.1% | 0.9% |
| Li B-2003 | 14 | 178 | 0.0787 | [0.0437; 0.1284] | 0.0% | 0.9% |
| Li WJ-2007 | 337 | 1553 | 0.2170 | [0.1967; 0.2383] | 0.4% | 0.9% |
| Li YB-2004 | 572 | 3336 | 0.1715 | [0.1588; 0.1847] | 0.8% | 0.9% |
| Wang HR-2007 | 515 | 2209 | 0.2331 | [0.2156; 0.2513] | 0.5% | 0.9% |
| Liu XG-2007 | 81 | 300 | 0.2700 | [0.2206; 0.3240] | 0.1% | 0.9% |
| Lu YH-2006 | 421 | 663 | 0.6350 | [0.5971; 0.6717] | 0.2% | 0.9% |
| Luo YX-2005 | 215 | 3864 | 0.0556 | [0.0486; 0.0633] | 0.9% | 0.9% |
| Nong CS-2007 | 172 | 377 | 0.4562 | [0.4051; 0.5080] | 0.1% | 0.9% |
| Tian JS-2007 | 35 | 778 | 0.0450 | [0.0315; 0.0620] | 0.2% | 0.9% |
| Wang ZZ-2007 | 1105 | 1234 | 0.8955 | [0.8770; 0.9120] | 0.3% | 0.9% |
| Wu CH-2003 | 4 | 148 | 0.0270 | [0.0074; 0.0678] | 0.0% | 0.9% |
| Zhang XF-2007 | 2152 | 4139 | 0.5199 | [0.5046; 0.5353] | 1.0% | 0.9% |
| Zhong SQ-2007 | 702 | 1239 | 0.5666 | [0.5385; 0.5944] | 0.3% | 0.9% |
| Zhu GZ-2007 | 1127 | 4944 | 0.2280 | [0.2163; 0.2399] | 1.2% | 0.9% |
| Jia Z-2014 | 3719 | 15852 | 0.2346 | [0.2280; 0.2413] | 3.8% | 0.9% |
| Li RC-2006 | 4839 | 10715 | 0.4516 | [0.4422; 0.4611] | 2.6% | 0.9% |
| Wong KH-2004 | 176 | 934 | 0.1884 | [0.1638; 0.2150] | 0.2% | 0.9% |
| Common effect model | | **73982** | **0.3352** | **[0.3318; 0.3386]** | **17.9%** | -- |
| Random effects model | | | **0.3581** | **[0.2627; 0.4596]** | -- | **24.4%** |
| Heterogeneity: $I^2 = 100\%$, $\tau^2 = 0.0745$, $p = 0$ | | | | | | |
| **Studyperiod = 2006-2011** | | | | | | |
| AYiGuLi-2010 | 13 | 151 | 0.0861 | [0.0466; 0.1427] | 0.0% | 0.9% |
| Bao ZY-2013 | 28 | 180 | 0.1556 | [0.1059; 0.2169] | 0.0% | 0.9% |
| Bi L-2008 | 107 | 1001 | 0.1069 | [0.0884; 0.1277] | 0.2% | 0.9% |
| Ning LF-2008 | 1086 | 3561 | 0.3050 | [0.2899; 0.3204] | 0.9% | 0.9% |
| Cheng Y-2007 | 10 | 140 | 0.0714 | [0.0348; 0.1274] | 0.0% | 0.9% |
| Du L-2013 | 7 | 235 | 0.0298 | [0.0121; 0.0604] | 0.1% | 0.9% |
| Fan LZ-2012 | 0 | 158 | 0.0000 | [0.0000; 0.0231] | 0.0% | 0.9% |
| Gu HY-2013 | 505 | 6258 | 0.0807 | [0.0741; 0.0877] | 1.5% | 0.9% |
| Yao XF-2013 | 806 | 2012 | 0.4006 | [0.3791; 0.4224] | 0.5% | 0.9% |
| Lu B-2008 | 208 | 1060 | 0.1962 | [0.1727; 0.2214] | 0.3% | 0.9% |
| Li MY-2008 | 169 | 768 | 0.2201 | [0.1912; 0.2510] | 0.2% | 0.9% |
| Lin CY-2009 | 403 | 4959 | 0.0813 | [0.0738; 0.0892] | 1.2% | 0.9% |
| Liu K-2009 | 111 | 939 | 0.1182 | [0.0983; 0.1406] | 0.2% | 0.9% |
| Liu XG-2008 | 209 | 566 | 0.3693 | [0.3294; 0.4105] | 0.1% | 0.9% |
| Lu B-2008 | 373 | 828 | 0.4505 | [0.4162; 0.4851] | 0.2% | 0.9% |
| Lu J-2009 | 259 | 1977 | 0.1310 | [0.1164; 0.1467] | 0.5% | 0.9% |
| Shao HW-2009 | 41 | 648 | 0.0633 | [0.0458; 0.0849] | 0.2% | 0.9% |
| Wang RL-2012 | 509 | 2028 | 0.2510 | [0.2322; 0.2705] | 0.5% | 0.9% |
| Xing XM-2011 | 185 | 812 | 0.2278 | [0.1994; 0.2583] | 0.2% | 0.9% |
| Yang F-2012 | 893 | 3771 | 0.2368 | [0.2233; 0.2507] | 0.9% | 0.9% |
| Yang B-2013 | 40 | 597 | 0.0670 | [0.0483; 0.0901] | 0.1% | 0.9% |
| Yu DS-2011 | 338 | 2429 | 0.1392 | [0.1256; 0.1536] | 0.6% | 0.9% |
| Yu WX-2012 | 138 | 5000 | 0.0276 | [0.0232; 0.0325] | 1.2% | 0.9% |
| Zhang P-2015 | 47 | 1195 | 0.0393 | [0.0290; 0.0520] | 0.3% | 0.9% |
| Zhao HL-2012 | 17 | 327 | 0.0520 | [0.0306; 0.0819] | 0.1% | 0.9% |
| Zheng RD-2013 | 207 | 850 | 0.2435 | [0.2150; 0.2738] | 0.2% | 0.9% |
| Zheng SJ-2015 | 55 | 205 | 0.2683 | [0.2090; 0.3345] | 0.0% | 0.9% |
| Zhong CF-2011 | 1074 | 2627 | 0.4088 | [0.3900; 0.4279] | 0.6% | 0.9% |
| Zhou HF-2006 | 47 | 175 | 0.2686 | [0.2045; 0.3407] | 0.0% | 0.9% |
| Chang Y-2009 | 522 | 2572 | 0.2030 | [0.1876; 0.2190] | 0.6% | 0.9% |
| Chiu DM-2013 | 129 | 450 | 0.2867 | [0.2453; 0.3309] | 0.1% | 0.9% |
| Dong C-2012 | 2793 | 14208 | 0.1966 | [0.1901; 0.2032] | 3.4% | 0.9% |
| Fu H-2010 | 70 | 296 | 0.2365 | [0.1892; 0.2891] | 0.1% | 0.9% |
| Shenyang G-2011 | 37 | 456 | 0.0811 | [0.0578; 0.1101] | 0.1% | 0.9% |
| Gu G-2015 | 57 | 994 | 0.0573 | [0.0437; 0.0737] | 0.2% | 0.9% |
| Taniguchi M-2009 | 143 | 300 | 0.4767 | [0.4190; 0.5348] | 0.1% | 0.9% |
| Li W-2011 | 69 | 173 | 0.3988 | [0.3253; 0.4759] | 0.0% | 0.9% |
| Lu J-2009 | 1547 | 8762 | 0.1766 | [0.1686; 0.1847] | 2.1% | 0.9% |
| Ma Z-2010 | 407 | 2090 | 0.1947 | [0.1780; 0.2124] | 0.5% | 0.9% |
| Xue Y-2013 | 70 | 260 | 0.2692 | [0.2163; 0.3275] | 0.1% | 0.9% |
| Yu Y-2009 | 988 | 4508 | 0.2192 | [0.2072; 0.2315] | 1.1% | 0.9% |
| Zhang W-2009 | 117 | 1476 | 0.0793 | [0.0660; 0.0942] | 0.4% | 0.9% |
| Common effect model | | **82002** | **0.1686** | **[0.1661; 0.1712]** | **19.8%** | -- |
| Random effects model | | | **0.1718** | **[0.1348; 0.2122]** | -- | **37.8%** |
| Heterogeneity: $I^2 = 99\%$, $\tau^2 = 0.0282$, $p = 0$ | | | | | | |

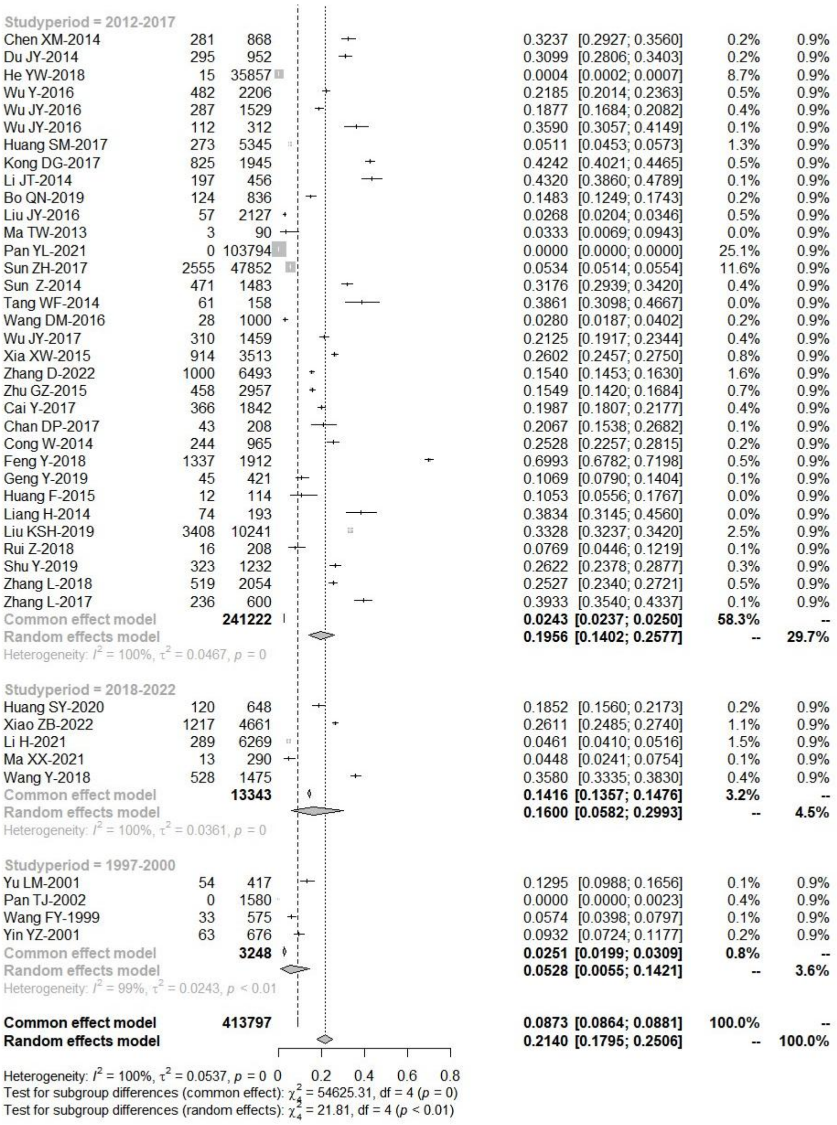


Figure S33. Forest plot of anti-HEV IgG prevalence in the general popualtion in different study periods

| Study | Events | Total | Proportion | 95%-CI | Weight (common) | Weight (random) |
|---|---|---|---|---|---|---|
| **studyperiod = 2001-2005** | | | | | | |
| Ao YY-2016 | 26 | 5012 | 0.0052 | [0.0034; 0.0076] | 0.5% | 1.3% |
| Cao HJ-2004 | 43 | 1934 | 0.0222 | [0.0161; 0.0298] | 0.2% | 1.2% |
| Jiang RJ-2006 | 17 | 1084 | 0.0157 | [0.0092; 0.0250] | 0.1% | 1.2% |
| Ge SX-2006 | 108 | 4110 | 0.0263 | [0.0216; 0.0316] | 0.4% | 1.3% |
| Yao MF-2007 | 35 | 1316 | 0.0266 | [0.0186; 0.0368] | 0.1% | 1.2% |
| Wang FD-2004 | 26 | 850 | 0.0306 | [0.0201; 0.0445] | 0.1% | 1.2% |
| Li WJ-2007 | 5 | 1553 | 0.0032 | [0.0010; 0.0075] | 0.1% | 1.2% |
| Zhang LF-2003 | 19 | 2223 | 0.0085 | [0.0052; 0.0133] | 0.2% | 1.2% |
| Sun LP-2004 | 20 | 1043 | 0.0192 | [0.0118; 0.0295] | 0.1% | 1.2% |
| Zhang XF-2007 | 103 | 4139 | 0.0249 | [0.0204; 0.0301] | 0.4% | 1.3% |
| Common effect model | | **23264** | **0.0156** | **[0.0140; 0.0172]** | **2.1%** | -- |
| Random effects model | | | **0.0166** | **[0.0106; 0.0239]** | -- | **12.1%** |
| Heterogeneity: $I^2 = 94\%$, $\tau^2 = 0.0016$, $p < 0.01$ | | | | | | |
| **studyperiod = 2006-2011** | | | | | | |
| Bi L-2008 | 5 | 1001 | 0.0050 | [0.0016; 0.0116] | 0.1% | 1.2% |
| Chen K-2106 | 79 | 10156 | 0.0078 | [0.0062; 0.0097] | 0.9% | 1.3% |
| Duan ZJ-2014 | 15 | 10803 | 0.0014 | [0.0008; 0.0023] | 1.0% | 1.3% |
| Gong Q-2012 | 45 | 10220 | 0.0044 | [0.0032; 0.0059] | 0.9% | 1.3% |
| Guo QL-2011 | 2 | 240 | 0.0083 | [0.0010; 0.0298] | 0.0% | 0.9% |
| Han XX-2010 | 6 | 11362 | 0.0005 | [0.0002; 0.0011] | 1.0% | 1.3% |
| Jin LP-2012 | 6 | 11461 | 0.0005 | [0.0002; 0.0011] | 1.0% | 1.3% |
| Kong P-2013 | 22 | 33211 | 0.0007 | [0.0004; 0.0010] | 3.0% | 1.3% |
| Li SB-2010 | 4 | 76654 | 0.0001 | [0.0000; 0.0001] | 7.0% | 1.3% |
| Lin Q-2012 | 4 | 5681 | 0.0007 | [0.0002; 0.0018] | 0.5% | 1.3% |
| Liu K-2009 | 1 | 939 | 0.0011 | [0.0000; 0.0059] | 0.1% | 1.2% |
| Liu Y-2013 | 115 | 25391 | 0.0045 | [0.0037; 0.0054] | 2.3% | 1.3% |
| Luo Y-2011 | 7 | 31137 | 0.0002 | [0.0001; 0.0005] | 2.8% | 1.3% |
| Ma XL-2015 | 12 | 3707 | 0.0032 | [0.0017; 0.0056] | 0.3% | 1.2% |
| Nong HY-2013 | 7 | 11938 | 0.0006 | [0.0002; 0.0012] | 1.1% | 1.3% |
| Shao HW-2009 | 1 | 648 | 0.0015 | [0.0000; 0.0086] | 0.1% | 1.1% |
| Sun JW-2009 | 65 | 3994 | 0.0163 | [0.0126; 0.0207] | 0.4% | 1.3% |
| Tian GJ-2007 | 13 | 288 | 0.0451 | [0.0243; 0.0760] | 0.0% | 1.0% |
| Wang LP-2013 | 10 | 25180 | 0.0004 | [0.0002; 0.0007] | 2.3% | 1.3% |
| Wu ZT-2013 | 14 | 4441 | 0.0032 | [0.0017; 0.0053] | 0.4% | 1.3% |
| Xia C-2012 | 11 | 749 | 0.0147 | [0.0074; 0.0261] | 0.1% | 1.1% |
| Zhang P-2015 | 3 | 1195 | 0.0025 | [0.0005; 0.0073] | 0.1% | 1.2% |
| Zhang WS-2012 | 3 | 6456 | 0.0005 | [0.0001; 0.0014] | 0.6% | 1.3% |
| Zhao HL-2012 | 8 | 327 | 0.0245 | [0.0106; 0.0476] | 0.0% | 1.0% |
| Zheng RD-2013 | 0 | 850 | 0.0000 | [0.0000; 0.0043] | 0.1% | 1.2% |
| Zheng Y-2015 | 21 | 26583 | 0.0008 | [0.0005; 0.0012] | 2.4% | 1.3% |
| Zheng Y-2014 | 124 | 118253 | 0.0010 | [0.0009; 0.0013] | 10.7% | 1.3% |
| Dong C-2012 | 4 | 14208 | 0.0003 | [0.0001; 0.0007] | 1.3% | 1.3% |
| Gu G-2015 | 4 | 994 | 0.0040 | [0.0011; 0.0103] | 0.1% | 1.2% |
| Li W-2011 | 11 | 173 | 0.0636 | [0.0322; 0.1109] | 0.0% | 0.8% |
| Ma Z-2010 | 9 | 2090 | 0.0043 | [0.0020; 0.0082] | 0.2% | 1.2% |
| Yu Y-2009 | 65 | 3994 | 0.0163 | [0.0126; 0.0207] | 0.4% | 1.3% |
| Zhang W-2009 | 29 | 1476 | 0.0196 | [0.0132; 0.0281] | 0.1% | 1.2% |
| Liu XJ-2008 | 11 | 502 | 0.0219 | [0.0110; 0.0389] | 0.0% | 1.1% |
| Common effect model | | **456302** | **0.0006** | **[0.0006; 0.0007]** | **41.4%** | -- |
| Random effects model | | | **0.0041** | **[0.0020; 0.0068]** | -- | **40.7%** |
| Heterogeneity: $I^2 = 97\%$, $\tau^2 = 0.0027$, $p < 0.01$ | | | | | | |
| **studyperiod = 2012-2017** | | | | | | |
| Zhang MM-2013 | 3 | 835 | 0.0036 | [0.0007; 0.0105] | 0.1% | 1.2% |
| Chen JP-2014 | 2 | 8697 | 0.0002 | [0.0000; 0.0008] | 0.8% | 1.3% |
| Chen JZ-2014 | 6 | 14275 | 0.0004 | [0.0002; 0.0009] | 1.3% | 1.3% |
| Chen JZ-2014 | 11 | 21612 | 0.0005 | [0.0003; 0.0009] | 2.0% | 1.3% |
| Xu LL-2016 | 1 | 3225 | 0.0003 | [0.0000; 0.0017] | 0.3% | 1.2% |
| Gao YP-2013 | 20 | 26780 | 0.0007 | [0.0005; 0.0012] | 2.4% | 1.3% |
| Wang XJ-2018 | 5 | 31696 | 0.0002 | [0.0001; 0.0004] | 2.9% | 1.3% |
| Jiang T-2018 | 26 | 9797 | 0.0027 | [0.0017; 0.0039] | 0.9% | 1.3% |
| Bo QN-2019 | 17 | 836 | 0.0203 | [0.0119; 0.0324] | 0.1% | 1.2% |
| Li XJ-2019 | 55 | 16022 | 0.0034 | [0.0026; 0.0045] | 1.5% | 1.3% |
| Li TQ-2016 | 37 | 8257 | 0.0045 | [0.0032; 0.0062] | 0.7% | 1.3% |
| Liu DX-2019 | 33 | 40182 | 0.0008 | [0.0006; 0.0012] | 3.6% | 1.3% |
| Liu F-2018 | 6 | 9529 | 0.0006 | [0.0002; 0.0014] | 0.9% | 1.3% |
| Liu JY-2016 | 3 | 2127 | 0.0014 | [0.0003; 0.0041] | 0.2% | 1.2% |
| Ma SB-2014 | 11 | 5639 | 0.0020 | [0.0010; 0.0035] | 0.5% | 1.3% |
| Qiu SH-2013 | 27 | 27977 | 0.0010 | [0.0006; 0.0014] | 2.5% | 1.3% |
| Sun C-2016 | 214 | 128833 | 0.0017 | [0.0014; 0.0019] | 11.7% | 1.3% |
| Wang DM-2016 | 2 | 1000 | 0.0020 | [0.0002; 0.0072] | 0.1% | 1.2% |
| Wang XH-2018 | 41 | 36152 | 0.0011 | [0.0008; 0.0015] | 3.3% | 1.3% |
| Wu ZH-2017 | 55 | 32120 | 0.0017 | [0.0013; 0.0022] | 2.9% | 1.3% |
| Wu JY-2017 | 7 | 1459 | 0.0048 | [0.0019; 0.0099] | 0.1% | 1.2% |
| Xiao ZY-2016 | 32 | 8952 | 0.0036 | [0.0024; 0.0050] | 0.8% | 1.3% |
| Xing Y-2016 | 164 | 95217 | 0.0017 | [0.0015; 0.0020] | 8.6% | 1.3% |
| Xu WL-2018 | 43 | 15897 | 0.0027 | [0.0020; 0.0036] | 1.4% | 1.3% |
| Yang XY-2016 | 26 | 12271 | 0.0021 | [0.0014; 0.0031] | 1.1% | 1.3% |
| Zhao JN-2017 | 10 | 10000 | 0.0010 | [0.0005; 0.0018] | 0.9% | 1.3% |
| Cai Y-2017 | 21 | 1842 | 0.0114 | [0.0071; 0.0174] | 0.2% | 1.2% |
| Cong W-2014 | 35 | 965 | 0.0363 | [0.0254; 0.0501] | 0.1% | 1.2% |
| Feng Y-2018 | 80 | 1912 | 0.0418 | [0.0333; 0.0518] | 0.2% | 1.2% |
| Geng Y-2019 | 5 | 421 | 0.0119 | [0.0039; 0.0275] | 0.0% | 1.1% |
| Huang F-2015 | 5 | 114 | 0.0439 | [0.0144; 0.0994] | 0.0% | 0.7% |
| Rui Z-2018 | 0 | 208 | 0.0000 | [0.0000; 0.0176] | 0.0% | 0.9% |
| Shu Y-2019 | 22 | 1232 | 0.0179 | [0.0112; 0.0269] | 0.1% | 1.2% |
| Common effect model | | **576081** | **0.0011** | **[0.0010; 0.0012]** | **52.3%** | -- |
| Random effects model | | | **0.0035** | **[0.0017; 0.0059]** | -- | **40.0%** |
| Heterogeneity: $I^2 = 96\%$, $\tau^2 = 0.0024$, $p < 0.01$ | | | | | | |
| **studyperiod = 2018-2022** | | | | | | |
| Li YH-2021 | 36 | 25098 | 0.0014 | [0.0010; 0.0020] | 2.3% | 1.3% |
| Wang JF-2021 | 29 | 11917 | 0.0024 | [0.0016; 0.0035] | 1.1% | 1.3% |
| Yuan ZZ-2022 | 10 | 1604 | 0.0062 | [0.0030; 0.0114] | 0.1% | 1.2% |
| Li H-2021 | 11 | 6269 | 0.0018 | [0.0009; 0.0031] | 0.6% | 1.3% |
| Ma XX-2021 | 0 | 290 | 0.0000 | [0.0000; 0.0126] | 0.0% | 1.0% |
| Wang Y-2018 | 10 | 1475 | 0.0068 | [0.0033; 0.0124] | 0.1% | 1.2% |
| Common effect model | | **46653** | **0.0016** | **[0.0012; 0.0020]** | **4.2%** | -- |
| Random effects model | | | **0.0026** | **[0.0010; 0.0046]** | -- | **7.2%** |
| Heterogeneity: $I^2 = 79\%$, $\tau^2 = 0.0003$, $p < 0.01$ | | | | | | |
| **Common effect model** | | **1102300** | **0.0011** | **[0.0010; 0.0011]** | **100.0%** | -- |
| **Random effects model** | | | **0.0048** | **[0.0033; 0.0066]** | -- | **100.0%** |

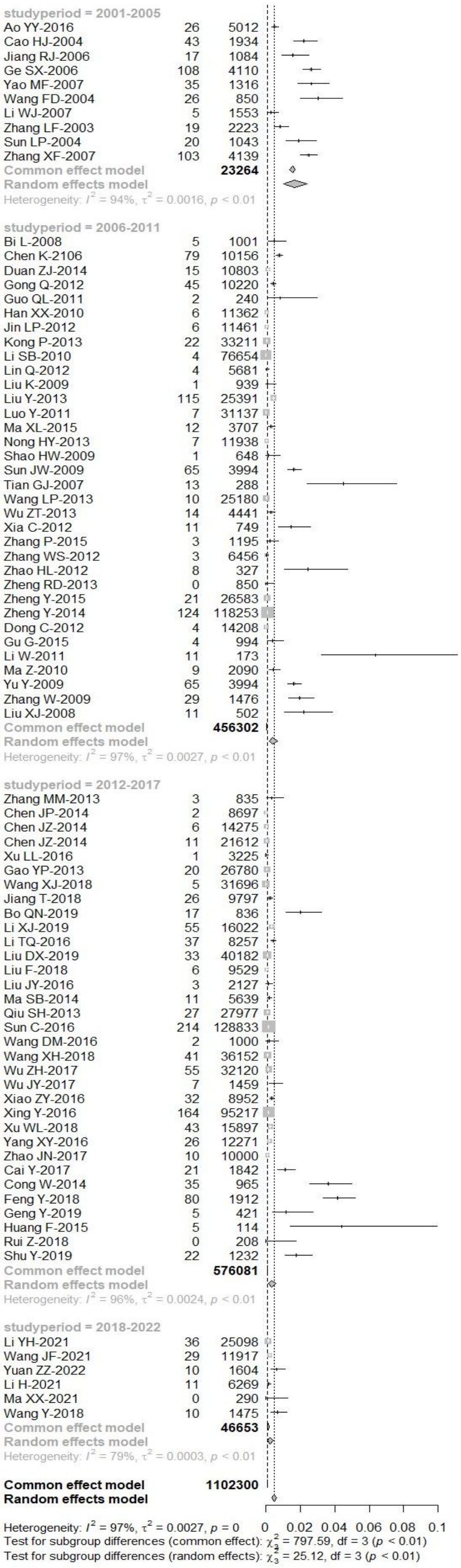


Heterogeneity: $I^2 = 97\%$, $\tau^2 = 0.0027$, $p = 0$
Test for subgroup differences (common effect): $\chi^2_3 = 797.59$, df = 3 ($p < 0.01$)
Test for subgroup differences (random effects): $\chi^2_3 = 25.12$, df = 3 ($p < 0.01$)

Figure S34. Forest plot of anti-HEV IgM prevalence in the general popualtion in different study periods

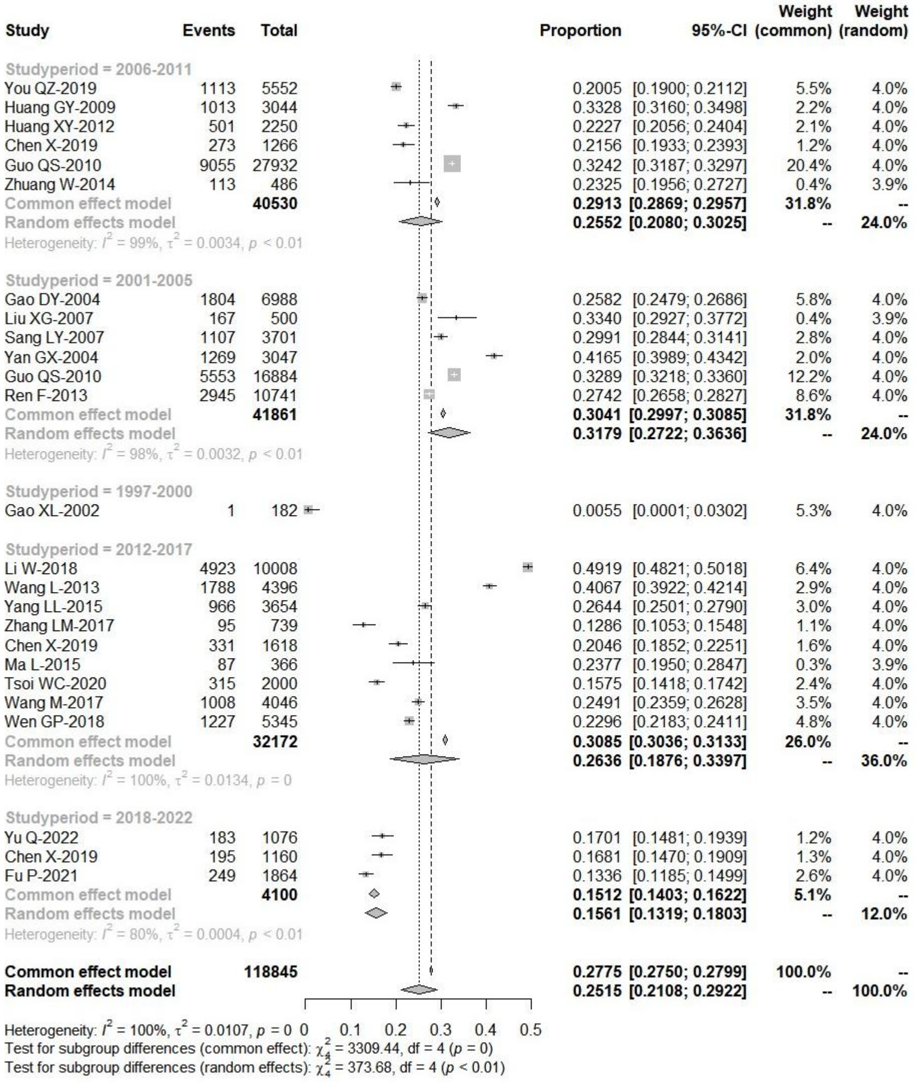


Figure S35. Forest plot of anti-HEV IgG prevalence in volunteer blood donors in different study periods

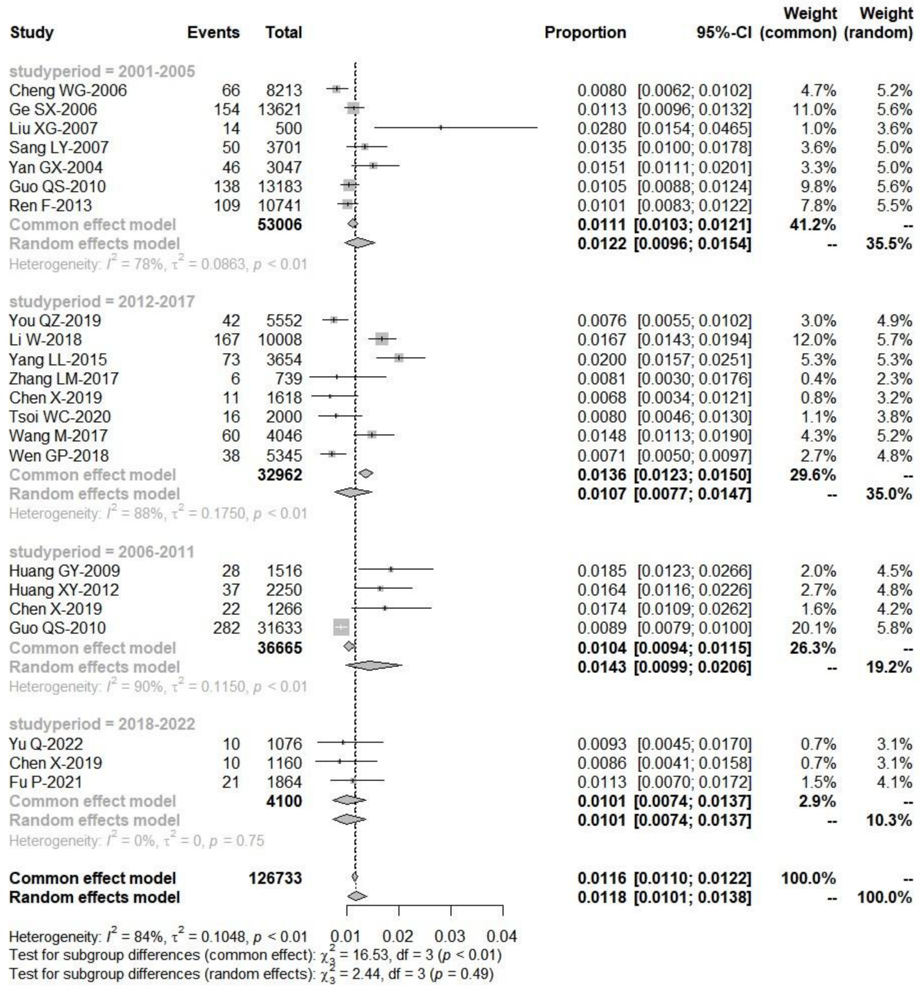


Figure S36. Forest plot of anti-HEV IgM prevalence in volunteer blood donors in different study periods

| Study | Events | Total | Proportion | 95%-CI | Weight (common) | Weight (random) |
|---|---|---|---|---|---|---|
| AYiGuLi-2010 | 13 | 151 | 0.0861 | [0.0466; 0.1427] | 0.1% | 1.2% |
| Ai X-2009 | 5588 | 12555 | 0.4451 | [0.4364; 0.4538] | 5.4% | 1.2% |
| Bao ZY-2013 | 28 | 180 | 0.1556 | [0.1059; 0.2169] | 0.1% | 1.2% |
| Bi L-2008 | 156 | 1204 | 0.1296 | [0.1111; 0.1499] | 0.5% | 1.2% |
| Bo QN-2018 | 127 | 1019 | 0.1246 | [0.1050; 0.1465] | 0.4% | 1.2% |
| Cai YS-2013 | 230 | 510 | 0.4510 | [0.4072; 0.4953] | 0.2% | 1.2% |
| Cao HJ-2004 | 881 | 2290 | 0.3847 | [0.3647; 0.4050] | 1.0% | 1.2% |
| Cao HJ-2004 | 146 | 189 | 0.7725 | [0.7060; 0.8302] | 0.1% | 1.2% |
| Shen JY-2007 | 749 | 1570 | 0.4771 | [0.4521; 0.5021] | 0.7% | 1.2% |
| Chen XM-2014 | 281 | 868 | 0.3237 | [0.2927; 0.3560] | 0.4% | 1.2% |
| Ning LF-2008 | 1086 | 3561 | 0.3050 | [0.2899; 0.3204] | 1.5% | 1.2% |
| Chen YZ-2006 | 254 | 1084 | 0.2343 | [0.2094; 0.2607] | 0.5% | 1.2% |
| Cheng Y-2007 | 10 | 140 | 0.0714 | [0.0348; 0.1274] | 0.1% | 1.2% |
| Du JY-2014 | 295 | 952 | 0.3099 | [0.2806; 0.3403] | 0.4% | 1.2% |
| Du L-2013 | 7 | 235 | 0.0298 | [0.0121; 0.0604] | 0.1% | 1.2% |
| Fan LZ-2012 | 0 | 158 | 0.0000 | [0.0000; 0.0231] | 0.1% | 1.2% |
| You QZ-2019 | 1113 | 5552 | 0.2005 | [0.1900; 0.2112] | 2.4% | 1.2% |
| Gao DY-2004 | 1804 | 6988 | 0.2582 | [0.2479; 0.2686] | 3.0% | 1.2% |
| Gao XL-2002 | 1 | 182 | 0.0055 | [0.0001; 0.0302] | 0.1% | 1.2% |
| Gu HY-2013 | 505 | 6258 | 0.0807 | [0.0741; 0.0877] | 2.7% | 1.2% |
| Yao XF-2013 | 806 | 2012 | 0.4006 | [0.3791; 0.4224] | 0.9% | 1.2% |
| He YW-2018 | 15 | 35857 | 0.0004 | [0.0002; 0.0007] | 15.4% | 1.2% |
| Wu Y-2016 | 482 | 2206 | 0.2185 | [0.2014; 0.2363] | 0.9% | 1.2% |
| Wu JY-2016 | 440 | 1720 | 0.2558 | [0.2353; 0.2771] | 0.7% | 1.2% |
| Wu JY-2016 | 317 | 611 | 0.5188 | [0.4784; 0.5591] | 0.3% | 1.2% |
| Zhou X-2015 | 124 | 912 | 0.1360 | [0.1144; 0.1599] | 0.4% | 1.2% |
| Huang GY-2009 | 1013 | 3044 | 0.3328 | [0.3160; 0.3498] | 1.3% | 1.2% |
| Yao MF-2007 | 635 | 1316 | 0.4825 | [0.4552; 0.5099] | 0.6% | 1.2% |
| Huang SY-2020 | 120 | 648 | 0.1852 | [0.1560; 0.2173] | 0.3% | 1.2% |
| Wang FD-2004 | 394 | 850 | 0.4635 | [0.4296; 0.4977] | 0.4% | 1.2% |
| Zhu JF-2007 | 29 | 176 | 0.1648 | [0.1132; 0.2280] | 0.1% | 1.2% |
| Kong DG-2017 | 825 | 1945 | 0.4242 | [0.4021; 0.4465] | 0.8% | 1.2% |
| Li YB-2004 | 572 | 3336 | 0.1715 | [0.1588; 0.1847] | 1.4% | 1.2% |
| Lin CY-2009 | 403 | 4959 | 0.0813 | [0.0738; 0.0892] | 2.1% | 1.2% |
| Liu JY-2016 | 57 | 2127 | 0.0268 | [0.0204; 0.0346] | 0.9% | 1.2% |
| Liu XG-2008 | 571 | 1351 | 0.4226 | [0.3961; 0.4495] | 0.6% | 1.2% |
| Lu J-2009 | 259 | 1977 | 0.1310 | [0.1164; 0.1467] | 0.8% | 1.2% |
| Lu YH-2006 | 421 | 663 | 0.6350 | [0.5971; 0.6717] | 0.3% | 1.2% |
| Luo YX-2005 | 215 | 3864 | 0.0556 | [0.0486; 0.0633] | 1.7% | 1.2% |
| Nong CS-2007 | 172 | 377 | 0.4562 | [0.4051; 0.5080] | 0.2% | 1.2% |
| Sun Z-2014 | 471 | 1483 | 0.3176 | [0.2939; 0.3420] | 0.6% | 1.2% |
| Wang DM-2016 | 28 | 1000 | 0.0280 | [0.0187; 0.0402] | 0.4% | 1.2% |
| Wang FY-1999 | 33 | 575 | 0.0574 | [0.0398; 0.0797] | 0.2% | 1.2% |
| Wang L-2013 | 1788 | 4396 | 0.4067 | [0.3922; 0.4214] | 1.9% | 1.2% |
| Wang YC-2005 | 297 | 409 | 0.7262 | [0.6802; 0.7688] | 0.2% | 1.2% |
| Zhu JF-2006 | 72 | 94 | 0.7660 | [0.6674; 0.8471] | 0.0% | 1.1% |
| Wang Q-2014 | 422 | 2812 | 0.1501 | [0.1371; 0.1638] | 1.2% | 1.2% |
| Wang RL-2012 | 509 | 2028 | 0.2510 | [0.2322; 0.2705] | 0.9% | 1.2% |
| Wu XX-2021 | 169 | 836 | 0.2022 | [0.1754; 0.2310] | 0.4% | 1.2% |
| Xia XW-2015 | 914 | 3513 | 0.2602 | [0.2457; 0.2750] | 1.5% | 1.2% |
| Xiao ZB-2022 | 1217 | 4661 | 0.2611 | [0.2485; 0.2740] | 2.0% | 1.2% |
| Xing XM-2011 | 185 | 812 | 0.2278 | [0.1994; 0.2583] | 0.3% | 1.2% |
| Yang F-2012 | 893 | 3771 | 0.2368 | [0.2233; 0.2507] | 1.6% | 1.2% |
| Yang B-2013 | 40 | 597 | 0.0670 | [0.0483; 0.0901] | 0.3% | 1.2% |
| Yang LL-2015 | 966 | 3654 | 0.2644 | [0.2501; 0.2790] | 1.6% | 1.2% |
| Yin YZ-2001 | 63 | 676 | 0.0932 | [0.0724; 0.1177] | 0.3% | 1.2% |
| Yu Q-2022 | 183 | 1076 | 0.1701 | [0.1481; 0.1939] | 0.5% | 1.2% |
| Yu WX-2012 | 138 | 5000 | 0.0276 | [0.0232; 0.0325] | 2.1% | 1.2% |
| Zhang XF-2007 | 2152 | 4139 | 0.5199 | [0.5046; 0.5353] | 1.8% | 1.2% |
| Zheng RD-2013 | 207 | 850 | 0.2435 | [0.2150; 0.2738] | 0.4% | 1.2% |
| Zheng SJ-2015 | 55 | 205 | 0.2683 | [0.2090; 0.3345] | 0.1% | 1.2% |
| Zhong CF-2011 | 1074 | 2627 | 0.4088 | [0.3900; 0.4279] | 1.1% | 1.2% |
| Zhong SQ-2007 | 702 | 1239 | 0.5666 | [0.5385; 0.5944] | 0.5% | 1.2% |
| Zhou HF-2006 | 47 | 175 | 0.2686 | [0.2045; 0.3407] | 0.1% | 1.2% |
| Cai Y-2017 | 366 | 1842 | 0.1987 | [0.1807; 0.2177] | 0.8% | 1.2% |
| Chan DP-2017 | 492 | 1539 | 0.3197 | [0.2964; 0.3436] | 0.7% | 1.2% |
| Chen X-2019 | 799 | 4044 | 0.1976 | [0.1854; 0.2102] | 1.7% | 1.2% |
| Cong W-2014 | 404 | 1955 | 0.2066 | [0.1889; 0.2253] | 0.8% | 1.2% |
| Cui W-2016 | 228 | 1028 | 0.2218 | [0.1967; 0.2485] | 0.4% | 1.2% |
| Feng Y-2018 | 1337 | 1912 | 0.6993 | [0.6782; 0.7198] | 0.8% | 1.2% |
| Fu P-2021 | 249 | 1864 | 0.1336 | [0.1185; 0.1499] | 0.8% | 1.2% |
| Geng Y-2019 | 79 | 496 | 0.1593 | [0.1282; 0.1945] | 0.2% | 1.2% |
| Gu G-2015 | 112 | 1491 | 0.0751 | [0.0623; 0.0897] | 0.6% | 1.2% |
| Jia Z-2014 | 3719 | 15852 | 0.2346 | [0.2280; 0.2413] | 6.8% | 1.2% |
| Taniguchi M-2009 | 143 | 300 | 0.4767 | [0.4190; 0.5348] | 0.1% | 1.2% |
| Li H-2021 | 289 | 6269 | 0.0461 | [0.0410; 0.0516] | 2.7% | 1.2% |
| Liu KSH-2019 | 3408 | 10241 | 0.3328 | [0.3237; 0.3420] | 4.4% | 1.2% |
| Lu J-2009 | 1547 | 8762 | 0.1766 | [0.1686; 0.1847] | 3.8% | 1.2% |
| Shu Y-2019 | 455 | 1505 | 0.3023 | [0.2792; 0.3262] | 0.6% | 1.2% |
| Tsoi WC-2020 | 315 | 2000 | 0.1575 | [0.1418; 0.1742] | 0.9% | 1.2% |
| Wang M-2017 | 1008 | 4046 | 0.2491 | [0.2359; 0.2628] | 1.7% | 1.2% |
| Wong KH-2004 | 176 | 934 | 0.1884 | [0.1638; 0.2150] | 0.4% | 1.2% |
| Xue Y-2013 | 164 | 529 | 0.3100 | [0.2708; 0.3514] | 0.2% | 1.2% |
| Zhang L-2018 | 1002 | 4102 | 0.2443 | [0.2312; 0.2577] | 1.8% | 1.2% |
| **Common effect model** | | **232936** | **0.1774** | **[0.1759; 0.1790]** | **100.0%** | **--** |
| **Random effects model** | | | **0.2406** | **[0.2012; 0.2823]** | **--** | **100.0%** |

Heterogeneity: $I^2 = 100\%$, $\tau^2 = 0.0487$, $p = 0$

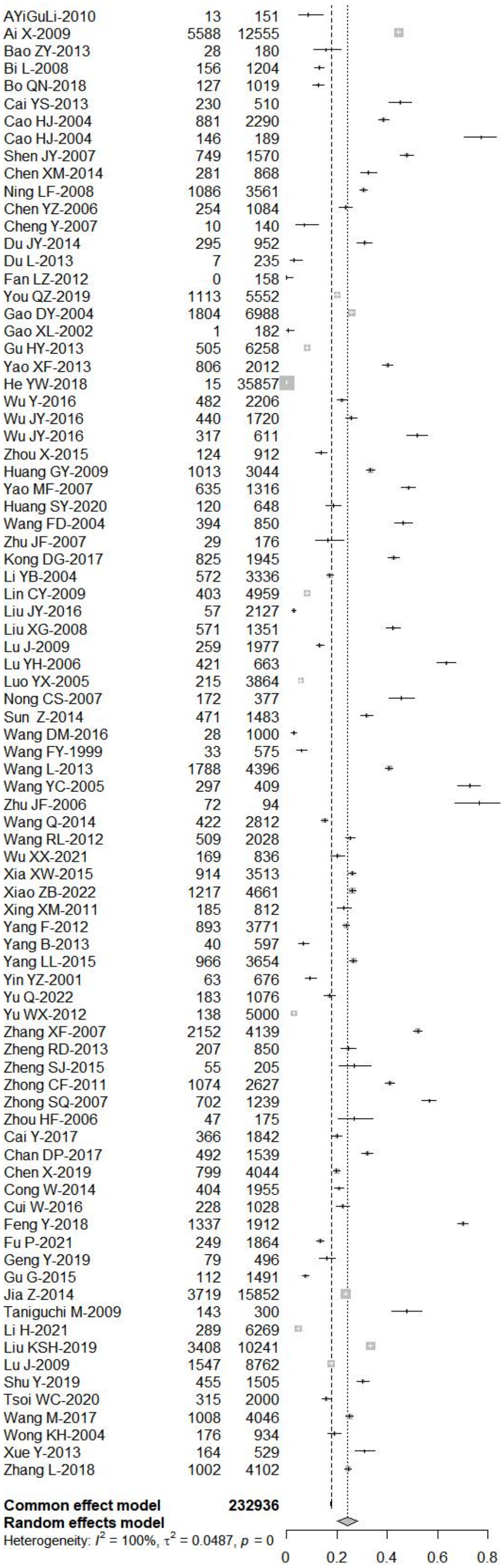


Figure S37. Sensitivity analysis of anti-HEV IgG positivity (1)Studies with a JBI score ≤5 were excluded

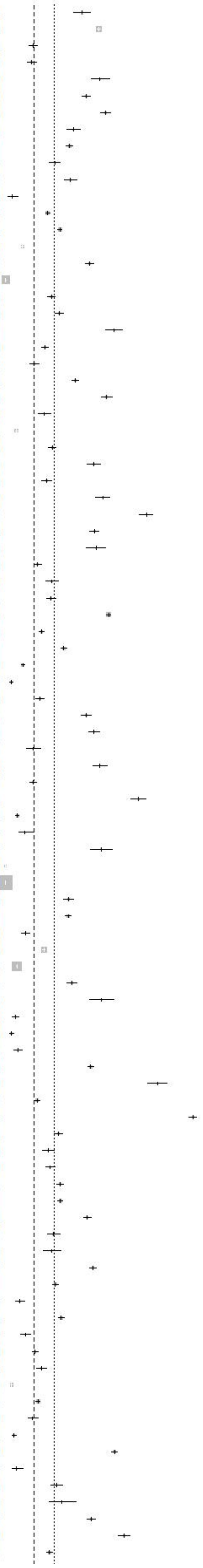

| Study | Events | Total | Proportion | 95%-CI | Weight (common) | Weight (random) |
|---|---|---|---|---|---|---|
| Zhang ZX-2003 | 210 | 574 | 0.3659 | [0.3264; 0.4067] | 0.1% | 0.7% |
| Ai X-2009 | 5588 | 12555 | 0.4451 | [0.4364; 0.4538] | 2.1% | 0.7% |
| Bi L-2008 | 156 | 1204 | 0.1296 | [0.1111; 0.1499] | 0.2% | 0.7% |
| Bo QN-2018 | 127 | 1019 | 0.1246 | [0.1050; 0.1465] | 0.2% | 0.7% |
| Cai YS-2013 | 230 | 510 | 0.4510 | [0.4072; 0.4953] | 0.1% | 0.7% |
| Cao HJ-2004 | 881 | 2290 | 0.3847 | [0.3647; 0.4050] | 0.4% | 0.7% |
| Shen JY-2007 | 749 | 1570 | 0.4771 | [0.4521; 0.5021] | 0.3% | 0.7% |
| Chen XM-2014 | 281 | 868 | 0.3237 | [0.2927; 0.3560] | 0.1% | 0.7% |
| Ning LF-2008 | 1086 | 3561 | 0.3050 | [0.2899; 0.3204] | 0.6% | 0.7% |
| Chen YZ-2006 | 254 | 1084 | 0.2343 | [0.2094; 0.2607] | 0.2% | 0.7% |
| Du JY-2014 | 295 | 952 | 0.3099 | [0.2806; 0.3403] | 0.2% | 0.7% |
| Du L-2013 | 7 | 235 | 0.0298 | [0.0121; 0.0604] | 0.0% | 0.7% |
| You QZ-2019 | 1113 | 5552 | 0.2005 | [0.1900; 0.2112] | 0.9% | 0.7% |
| Gao DY-2004 | 1804 | 6988 | 0.2582 | [0.2479; 0.2686] | 1.2% | 0.7% |
| Gu HY-2013 | 505 | 6258 | 0.0807 | [0.0741; 0.0877] | 1.0% | 0.7% |
| Yao XF-2013 | 806 | 2012 | 0.4006 | [0.3791; 0.4224] | 0.3% | 0.7% |
| He YW-2018 | 15 | 35857 | 0.0004 | [0.0002; 0.0007] | 6.0% | 0.7% |
| Wu Y-2016 | 482 | 2206 | 0.2185 | [0.2014; 0.2363] | 0.4% | 0.7% |
| Wu JY-2016 | 440 | 1720 | 0.2558 | [0.2353; 0.2771] | 0.3% | 0.7% |
| Wu JY-2016 | 317 | 611 | 0.5188 | [0.4784; 0.5591] | 0.1% | 0.7% |
| Hu AQ-2010 | 377 | 2019 | 0.1867 | [0.1699; 0.2044] | 0.3% | 0.7% |
| Zhou X-2015 | 124 | 912 | 0.1360 | [0.1144; 0.1599] | 0.2% | 0.7% |
| Huang GY-2009 | 1013 | 3044 | 0.3328 | [0.3160; 0.3498] | 0.5% | 0.7% |
| Yao MF-2007 | 635 | 1316 | 0.4825 | [0.4552; 0.5099] | 0.2% | 0.7% |
| Huang SY-2020 | 120 | 648 | 0.1852 | [0.1560; 0.2173] | 0.1% | 0.7% |
| Huang SM-2017 | 273 | 5345 | 0.0511 | [0.0453; 0.0573] | 0.9% | 0.7% |
| Huang XY-2012 | 501 | 2250 | 0.2227 | [0.2056; 0.2404] | 0.4% | 0.7% |
| Meng ZH-2005 | 413 | 980 | 0.4214 | [0.3903; 0.4531] | 0.2% | 0.7% |
| Lu B-2008 | 208 | 1060 | 0.1962 | [0.1727; 0.2214] | 0.2% | 0.7% |
| Wang FD-2004 | 394 | 850 | 0.4635 | [0.4296; 0.4977] | 0.1% | 0.7% |
| Zheng YJ-2005 | 573 | 852 | 0.6725 | [0.6399; 0.7040] | 0.1% | 0.7% |
| Kong DG-2017 | 825 | 1945 | 0.4242 | [0.4021; 0.4465] | 0.3% | 0.7% |
| Li JT-2014 | 197 | 456 | 0.4320 | [0.3860; 0.4789] | 0.1% | 0.7% |
| Bo QN-2019 | 259 | 1700 | 0.1524 | [0.1356; 0.1703] | 0.3% | 0.7% |
| Li MY-2008 | 169 | 768 | 0.2201 | [0.1912; 0.2510] | 0.1% | 0.7% |
| Li WJ-2007 | 337 | 1553 | 0.2170 | [0.1967; 0.2383] | 0.3% | 0.7% |
| Li W-2018 | 4923 | 10008 | 0.4919 | [0.4821; 0.5018] | 1.7% | 0.7% |
| Li YB-2004 | 572 | 3336 | 0.1715 | [0.1588; 0.1847] | 0.6% | 0.7% |
| Wang HR-2007 | 1089 | 3931 | 0.2770 | [0.2631; 0.2913] | 0.7% | 0.7% |
| Lin CY-2009 | 403 | 4959 | 0.0813 | [0.0738; 0.0892] | 0.8% | 0.7% |
| Liu JY-2016 | 57 | 2127 | 0.0268 | [0.0204; 0.0346] | 0.4% | 0.7% |
| Liu K-2009 | 223 | 1365 | 0.1634 | [0.1441; 0.1841] | 0.2% | 0.7% |
| Liu XG-2007 | 610 | 1585 | 0.3849 | [0.3608; 0.4093] | 0.3% | 0.7% |
| Liu XG-2008 | 571 | 1351 | 0.4226 | [0.3961; 0.4495] | 0.2% | 0.7% |
| Yu LM-2001 | 54 | 417 | 0.1295 | [0.0988; 0.1656] | 0.1% | 0.7% |
| Lu B-2008 | 373 | 828 | 0.4505 | [0.4162; 0.4851] | 0.1% | 0.7% |
| Lu J-2009 | 259 | 1977 | 0.1310 | [0.1164; 0.1467] | 0.3% | 0.7% |
| Lu YH-2006 | 421 | 663 | 0.6350 | [0.5971; 0.6717] | 0.1% | 0.7% |
| Luo YX-2005 | 215 | 3864 | 0.0556 | [0.0486; 0.0633] | 0.6% | 0.7% |
| Ma TW-2013 | 30 | 324 | 0.0926 | [0.0633; 0.1295] | 0.1% | 0.7% |
| Nong CS-2007 | 172 | 377 | 0.4562 | [0.4051; 0.5080] | 0.1% | 0.7% |
| Pan TJ-2002 | 0 | 1580 | 0.0000 | [0.0000; 0.0023] | 0.3% | 0.7% |
| Pan YL-2021 | 0 | 103794 | 0.0000 | [0.0000; 0.0000] | 17.3% | 0.7% |
| Pu MH-2008 | 408 | 1360 | 0.3000 | [0.2757; 0.3251] | 0.2% | 0.7% |
| Sang LY-2007 | 1107 | 3701 | 0.2991 | [0.2844; 0.3141] | 0.6% | 0.7% |
| Shao HW-2009 | 79 | 830 | 0.0952 | [0.0761; 0.1172] | 0.1% | 0.7% |
| Shao JS-2006 | 2565 | 14020 | 0.1830 | [0.1766; 0.1895] | 2.3% | 0.7% |
| Sun ZH-2017 | 2555 | 47852 | 0.0534 | [0.0514; 0.0554] | 8.0% | 0.7% |
| Sun Z-2014 | 471 | 1483 | 0.3176 | [0.2939; 0.3420] | 0.2% | 0.7% |
| Tang WF-2014 | 139 | 303 | 0.4587 | [0.4016; 0.5167] | 0.1% | 0.7% |
| Tian JS-2007 | 35 | 778 | 0.0450 | [0.0315; 0.0620] | 0.1% | 0.7% |
| Wang DM-2016 | 28 | 1000 | 0.0280 | [0.0187; 0.0402] | 0.2% | 0.7% |
| Wang FY-1999 | 33 | 575 | 0.0574 | [0.0398; 0.0797] | 0.1% | 0.7% |
| Wang L-2013 | 1788 | 4396 | 0.4067 | [0.3922; 0.4214] | 0.7% | 0.7% |
| Wang YC-2005 | 297 | 409 | 0.7262 | [0.6802; 0.7688] | 0.1% | 0.7% |
| Wang Q-2014 | 422 | 2812 | 0.1501 | [0.1371; 0.1638] | 0.5% | 0.7% |
| Wang ZZ-2007 | 1105 | 1234 | 0.8955 | [0.8770; 0.9120] | 0.2% | 0.7% |
| Wang RL-2012 | 509 | 2028 | 0.2510 | [0.2322; 0.2705] | 0.3% | 0.7% |
| Wu XX-2021 | 169 | 836 | 0.2022 | [0.1754; 0.2310] | 0.1% | 0.7% |
| Wu JY-2017 | 310 | 1459 | 0.2125 | [0.1917; 0.2344] | 0.2% | 0.7% |
| Xia XW-2015 | 914 | 3513 | 0.2602 | [0.2457; 0.2750] | 0.6% | 0.7% |
| Xiao ZB-2022 | 1217 | 4661 | 0.2611 | [0.2485; 0.2740] | 0.8% | 0.7% |
| Xie SF-2014 | 1019 | 2614 | 0.3898 | [0.3711; 0.4088] | 0.4% | 0.7% |
| Xing XM-2011 | 185 | 812 | 0.2278 | [0.1994; 0.2583] | 0.1% | 0.7% |
| Xu PN-2014 | 91 | 415 | 0.2193 | [0.1804; 0.2622] | 0.1% | 0.7% |
| Yan GX-2004 | 1269 | 3047 | 0.4165 | [0.3989; 0.4342] | 0.5% | 0.7% |
| Yang F-2012 | 893 | 3771 | 0.2368 | [0.2233; 0.2507] | 0.6% | 0.7% |
| Yang B-2013 | 40 | 597 | 0.0670 | [0.0483; 0.0901] | 0.1% | 0.7% |
| Yang LL-2015 | 966 | 3654 | 0.2644 | [0.2501; 0.2790] | 0.6% | 0.7% |
| Yin YZ-2001 | 63 | 676 | 0.0932 | [0.0724; 0.1177] | 0.1% | 0.7% |
| Yu DS-2011 | 338 | 2429 | 0.1392 | [0.1256; 0.1536] | 0.4% | 0.7% |
| Yu Q-2022 | 183 | 1076 | 0.1701 | [0.1481; 0.1939] | 0.2% | 0.7% |
| Yu WX-2012 | 138 | 5000 | 0.0276 | [0.0232; 0.0325] | 0.8% | 0.7% |
| Zhang D-2022 | 1000 | 6493 | 0.1540 | [0.1453; 0.1630] | 1.1% | 0.7% |
| Zhang LM-2017 | 95 | 739 | 0.1286 | [0.1053; 0.1548] | 0.1% | 0.7% |
| Zhang P-2015 | 47 | 1195 | 0.0393 | [0.0290; 0.0520] | 0.2% | 0.7% |
| Zhang XF-2007 | 2152 | 4139 | 0.5199 | [0.5046; 0.5353] | 0.7% | 0.7% |
| Zhao HL-2012 | 17 | 327 | 0.0520 | [0.0306; 0.0819] | 0.1% | 0.7% |
| Zheng RD-2013 | 207 | 850 | 0.2435 | [0.2150; 0.2738] | 0.1% | 0.7% |
| Zheng SJ-2015 | 55 | 205 | 0.2683 | [0.2090; 0.3345] | 0.0% | 0.7% |
| Zhong CF-2011 | 1074 | 2627 | 0.4088 | [0.3900; 0.4279] | 0.4% | 0.7% |
| Zhong SQ-2007 | 702 | 1239 | 0.5666 | [0.5385; 0.5944] | 0.2% | 0.7% |
| Zhu GZ-2015 | 690 | 3305 | 0.2088 | [0.1950; 0.2230] | 0.6% | 0.7% |

| | | | | | | |
|---|---|---|---|---|---|---|
| Zhu GZ-2007 | 1127 | 4944 | 0.2280 | [0.2163; 0.2399] | 0.8% | 0.7% |
| Cai Y-2017 | 366 | 1842 | 0.1987 | [0.1807; 0.2177] | 0.3% | 0.7% |
| Chan DP-2017 | 492 | 1539 | 0.3197 | [0.2964; 0.3436] | 0.3% | 0.7% |
| Chang Y-2009 | 627 | 2819 | 0.2224 | [0.2072; 0.2382] | 0.5% | 0.7% |
| Chen X-2019 | 799 | 4044 | 0.1976 | [0.1854; 0.2102] | 0.7% | 0.7% |
| Chiu DM-2013 | 129 | 450 | 0.2867 | [0.2453; 0.3309] | 0.1% | 0.7% |
| Cong W-2014 | 404 | 1955 | 0.2066 | [0.1889; 0.2253] | 0.3% | 0.7% |
| Cui W-2016 | 228 | 1028 | 0.2218 | [0.1967; 0.2485] | 0.2% | 0.7% |
| Dong C-2012 | 2793 | 14208 | 0.1966 | [0.1901; 0.2032] | 2.4% | 0.7% |
| Feng Y-2018 | 1337 | 1912 | 0.6993 | [0.6782; 0.7198] | 0.3% | 0.7% |
| Fu H-2010 | 70 | 296 | 0.2365 | [0.1892; 0.2891] | 0.0% | 0.7% |
| Fu P-2021 | 249 | 1864 | 0.1336 | [0.1185; 0.1499] | 0.3% | 0.7% |
| Shenyang G-2011 | 37 | 456 | 0.0811 | [0.0578; 0.1101] | 0.1% | 0.7% |
| Geng Y-2019 | 79 | 496 | 0.1593 | [0.1282; 0.1945] | 0.1% | 0.7% |
| Gu G-2015 | 112 | 1491 | 0.0751 | [0.0623; 0.0897] | 0.2% | 0.7% |
| Guo QS-2010 | 14608 | 44816 | 0.3260 | [0.3216; 0.3303] | 7.5% | 0.7% |
| Huang F-2013 | 30 | 293 | 0.1024 | [0.0702; 0.1429] | 0.0% | 0.7% |
| Huang F-2015 | 36 | 388 | 0.0928 | [0.0658; 0.1261] | 0.1% | 0.7% |
| Huang H-2016 | 42 | 391 | 0.1074 | [0.0785; 0.1424] | 0.1% | 0.7% |
| Jia Z-2014 | 3719 | 15852 | 0.2346 | [0.2280; 0.2413] | 2.6% | 0.7% |
| Taniguchi M-2009 | 143 | 300 | 0.4767 | [0.4190; 0.5348] | 0.1% | 0.7% |
| Li H-2021 | 289 | 6269 | 0.0461 | [0.0410; 0.0516] | 1.0% | 0.7% |
| Li M-2020 | 157 | 946 | 0.1660 | [0.1428; 0.1912] | 0.2% | 0.7% |
| Li RC-2006 | 4839 | 10715 | 0.4516 | [0.4422; 0.4611] | 1.8% | 0.7% |
| Liang H-2014 | 129 | 307 | 0.4202 | [0.3644; 0.4776] | 0.1% | 0.7% |
| Liu KSH-2019 | 3408 | 10241 | 0.3328 | [0.3237; 0.3420] | 1.7% | 0.7% |
| Lu J-2009 | 1547 | 8762 | 0.1766 | [0.1686; 0.1847] | 1.5% | 0.7% |
| Ma L-2015 | 87 | 366 | 0.2377 | [0.1950; 0.2847] | 0.1% | 0.7% |
| Ma XX-2021 | 220 | 3568 | 0.0617 | [0.0540; 0.0701] | 0.6% | 0.7% |
| Ma Z-2010 | 407 | 2090 | 0.1947 | [0.1780; 0.2124] | 0.3% | 0.7% |
| Qian Z-2022 | 2275 | 19762 | 0.1151 | [0.1107; 0.1197] | 3.3% | 0.7% |
| Ren F-2013 | 2945 | 10741 | 0.2742 | [0.2658; 0.2827] | 1.8% | 0.7% |
| Rui Z-2018 | 65 | 433 | 0.1501 | [0.1178; 0.1873] | 0.1% | 0.7% |
| Shu Y-2019 | 455 | 1505 | 0.3023 | [0.2792; 0.3262] | 0.3% | 0.7% |
| Tsoi WC-2020 | 315 | 2000 | 0.1575 | [0.1418; 0.1742] | 0.3% | 0.7% |
| Wang M-2017 | 1008 | 4046 | 0.2491 | [0.2359; 0.2628] | 0.7% | 0.7% |
| Wang Y-2018 | 973 | 2592 | 0.3754 | [0.3567; 0.3944] | 0.4% | 0.7% |
| Wen GP-2018 | 1227 | 5345 | 0.2296 | [0.2183; 0.2411] | 0.9% | 0.7% |
| Wong KH-2004 | 176 | 934 | 0.1884 | [0.1638; 0.2150] | 0.2% | 0.7% |
| Xue Y-2013 | 164 | 529 | 0.3100 | [0.2708; 0.3514] | 0.1% | 0.7% |
| Yu Y-2009 | 1299 | 5493 | 0.2365 | [0.2253; 0.2479] | 0.9% | 0.7% |
| Zhang L-2018 | 1002 | 4102 | 0.2443 | [0.2312; 0.2577] | 0.7% | 0.7% |
| Zhang L-2017 | 236 | 600 | 0.3933 | [0.3540; 0.4337] | 0.1% | 0.7% |
| Zhang W-2009 | 117 | 1476 | 0.0793 | [0.0660; 0.0942] | 0.2% | 0.7% |
| Zhuang W-2014 | 113 | 486 | 0.2325 | [0.1956; 0.2727] | 0.1% | 0.7% |
| **Common effect model** | | **598962** | **0.1335** | **[0.1326; 0.1344]** | **100.0%** | **--** |
| **Random effects model** | | | **0.2322** | **[0.2041; 0.2615]** | **--** | **100.0%** |

Heterogeneity: $I^2 = 100\%$, $\tau^2 = 0.0412$, $p = 0$

0 0.2 0.4 0.6 0.8

(2)Studies with sample sizes ≤ 200 were excluded

| Study | Events | Total | Proportion | 95%-CI | Weight (common) | Weight (random) |
|---|---|---|---|---|---|---|
| Zhang ZX-2003 | 210 | 574 | 0.3659 | [0.3264; 0.4067] | 0.1% | 0.7% |
| Ai X-2009 | 5588 | 12555 | 0.4451 | [0.4364; 0.4538] | 2.1% | 0.8% |
| Bi L-2008 | 156 | 1204 | 0.1296 | [0.1111; 0.1499] | 0.2% | 0.8% |
| Bo QN-2018 | 127 | 1019 | 0.1246 | [0.1050; 0.1465] | 0.2% | 0.8% |
| Cai YS-2013 | 230 | 510 | 0.4510 | [0.4072; 0.4953] | 0.1% | 0.7% |
| Cao HJ-2004 | 881 | 2290 | 0.3847 | [0.3647; 0.4050] | 0.4% | 0.8% |
| Shen JY-2007 | 749 | 1570 | 0.4771 | [0.4521; 0.5021] | 0.3% | 0.8% |
| Chen XM-2014 | 281 | 868 | 0.3237 | [0.2927; 0.3560] | 0.1% | 0.8% |
| Ning LF-2008 | 1086 | 3561 | 0.3050 | [0.2899; 0.3204] | 0.6% | 0.8% |
| Chen YZ-2006 | 254 | 1084 | 0.2343 | [0.2094; 0.2607] | 0.2% | 0.8% |
| Du JY-2014 | 295 | 952 | 0.3099 | [0.2806; 0.3403] | 0.2% | 0.8% |
| You QZ-2019 | 1113 | 5552 | 0.2005 | [0.1900; 0.2112] | 0.9% | 0.8% |
| Gao DY-2004 | 1804 | 6988 | 0.2582 | [0.2479; 0.2686] | 1.2% | 0.8% |
| Gu HY-2013 | 505 | 6258 | 0.0807 | [0.0741; 0.0877] | 1.0% | 0.8% |
| Yao XF-2013 | 806 | 2012 | 0.4006 | [0.3791; 0.4224] | 0.3% | 0.8% |
| He YW-2018 | 15 | 35857 | 0.0004 | [0.0002; 0.0007] | 6.0% | 0.8% |
| Wu Y-2016 | 482 | 2206 | 0.2185 | [0.2014; 0.2363] | 0.4% | 0.8% |
| Wu JY-2016 | 440 | 1720 | 0.2558 | [0.2353; 0.2771] | 0.3% | 0.8% |
| Wu JY-2016 | 317 | 611 | 0.5188 | [0.4784; 0.5591] | 0.1% | 0.7% |
| Hu AQ-2010 | 377 | 2019 | 0.1867 | [0.1699; 0.2044] | 0.3% | 0.8% |
| Zhou X-2015 | 124 | 912 | 0.1360 | [0.1144; 0.1599] | 0.2% | 0.8% |
| Huang GY-2009 | 1013 | 3044 | 0.3328 | [0.3160; 0.3498] | 0.5% | 0.8% |
| Yao MF-2007 | 635 | 1316 | 0.4825 | [0.4552; 0.5099] | 0.2% | 0.8% |
| Huang SY-2020 | 120 | 648 | 0.1852 | [0.1560; 0.2173] | 0.1% | 0.7% |
| Huang SM-2017 | 273 | 5345 | 0.0511 | [0.0453; 0.0573] | 0.9% | 0.8% |
| Huang XY-2012 | 501 | 2250 | 0.2227 | [0.2056; 0.2404] | 0.4% | 0.8% |
| Meng ZH-2005 | 413 | 980 | 0.4214 | [0.3903; 0.4531] | 0.2% | 0.8% |
| Lu B-2008 | 208 | 1060 | 0.1962 | [0.1727; 0.2214] | 0.2% | 0.8% |
| Wang FD-2004 | 394 | 850 | 0.4635 | [0.4296; 0.4977] | 0.1% | 0.8% |
| Zheng YJ-2005 | 573 | 852 | 0.6725 | [0.6399; 0.7040] | 0.1% | 0.8% |
| Kong DG-2017 | 825 | 1945 | 0.4242 | [0.4021; 0.4465] | 0.3% | 0.8% |
| Li JT-2014 | 197 | 456 | 0.4320 | [0.3860; 0.4789] | 0.1% | 0.7% |
| Bo QN-2019 | 259 | 1700 | 0.1524 | [0.1356; 0.1703] | 0.3% | 0.8% |
| Li MY-2008 | 169 | 768 | 0.2201 | [0.1912; 0.2510] | 0.1% | 0.7% |
| Li WJ-2007 | 337 | 1553 | 0.2170 | [0.1967; 0.2383] | 0.3% | 0.8% |
| Li W-2018 | 4923 | 10008 | 0.4919 | [0.4821; 0.5018] | 1.7% | 0.8% |
| Li YB-2004 | 572 | 3336 | 0.1715 | [0.1588; 0.1847] | 0.6% | 0.8% |
| Wang HR-2007 | 1089 | 3931 | 0.2770 | [0.2631; 0.2913] | 0.7% | 0.8% |
| Lin CY-2009 | 403 | 4959 | 0.0813 | [0.0738; 0.0892] | 0.8% | 0.8% |
| Liu JY-2016 | 57 | 2127 | 0.0268 | [0.0204; 0.0346] | 0.4% | 0.8% |
| Liu K-2009 | 223 | 1365 | 0.1634 | [0.1441; 0.1841] | 0.2% | 0.8% |
| Liu XG-2007 | 610 | 1585 | 0.3849 | [0.3608; 0.4093] | 0.3% | 0.8% |
| Liu XG-2008 | 571 | 1351 | 0.4226 | [0.3961; 0.4495] | 0.2% | 0.8% |
| Yu LM-2001 | 54 | 417 | 0.1295 | [0.0988; 0.1656] | 0.1% | 0.7% |
| Lu B-2008 | 373 | 828 | 0.4505 | [0.4162; 0.4851] | 0.1% | 0.8% |
| Lu J-2009 | 259 | 1977 | 0.1310 | [0.1164; 0.1467] | 0.3% | 0.8% |
| Lu YH-2006 | 421 | 663 | 0.6350 | [0.5971; 0.6717] | 0.1% | 0.7% |
| Luo YX-2005 | 215 | 3864 | 0.0556 | [0.0486; 0.0633] | 0.6% | 0.8% |
| Ma TW-2013 | 30 | 324 | 0.0926 | [0.0633; 0.1295] | 0.1% | 0.7% |
| Nong CS-2007 | 172 | 377 | 0.4562 | [0.4051; 0.5080] | 0.1% | 0.7% |
| Pan TJ-2002 | 0 | 1580 | 0.0000 | [0.0000; 0.0023] | 0.3% | 0.8% |
| Pan YL-2021 | 0 | 103794 | 0.0000 | [0.0000; 0.0000] | 17.4% | 0.8% |
| Pu MH-2008 | 408 | 1360 | 0.3000 | [0.2757; 0.3251] | 0.2% | 0.8% |
| Sang LY-2007 | 1107 | 3701 | 0.2991 | [0.2844; 0.3141] | 0.6% | 0.8% |
| Shao HW-2009 | 79 | 830 | 0.0952 | [0.0761; 0.1172] | 0.1% | 0.8% |
| Shao JS-2006 | 2565 | 14020 | 0.1830 | [0.1766; 0.1895] | 2.3% | 0.8% |
| Sun ZH-2017 | 2555 | 47852 | 0.0534 | [0.0514; 0.0554] | 8.0% | 0.8% |
| Sun Z-2014 | 471 | 1483 | 0.3176 | [0.2939; 0.3420] | 0.2% | 0.8% |
| Tang WF-2014 | 139 | 303 | 0.4587 | [0.4016; 0.5167] | 0.1% | 0.7% |
| Tian JS-2007 | 35 | 778 | 0.0450 | [0.0315; 0.0620] | 0.1% | 0.7% |
| Wang DM-2016 | 28 | 1000 | 0.0280 | [0.0187; 0.0402] | 0.2% | 0.8% |
| Wang FY-1999 | 33 | 575 | 0.0574 | [0.0398; 0.0797] | 0.1% | 0.7% |

| | | | | | | |
|---|---|---|---|---|---|---|
| Wang L-2013 | 1788 | 4396 | 0.4067 | [0.3922; 0.4214] | 0.7% | 0.8% |
| Wang YC-2005 | 297 | 409 | 0.7262 | [0.6802; 0.7688] | 0.1% | 0.7% |
| Wang Q-2014 | 422 | 2812 | 0.1501 | [0.1371; 0.1638] | 0.5% | 0.8% |
| Wang ZZ-2007 | 1105 | 1234 | 0.8955 | [0.8770; 0.9120] | 0.2% | 0.8% |
| Wang RL-2012 | 509 | 2028 | 0.2510 | [0.2322; 0.2705] | 0.3% | 0.8% |
| Wu XX-2021 | 169 | 836 | 0.2022 | [0.1754; 0.2310] | 0.1% | 0.8% |
| Wu JY-2017 | 310 | 1459 | 0.2125 | [0.1917; 0.2344] | 0.2% | 0.8% |
| Xia XW-2015 | 914 | 3513 | 0.2602 | [0.2457; 0.2750] | 0.6% | 0.8% |
| Xiao ZB-2022 | 1217 | 4661 | 0.2611 | [0.2485; 0.2740] | 0.8% | 0.8% |
| Xie SF-2014 | 1019 | 2614 | 0.3898 | [0.3711; 0.4088] | 0.4% | 0.8% |
| Xing XM-2011 | 185 | 812 | 0.2278 | [0.1994; 0.2583] | 0.1% | 0.8% |
| Xu PN-2014 | 91 | 415 | 0.2193 | [0.1804; 0.2622] | 0.1% | 0.7% |
| Yan GX-2004 | 1269 | 3047 | 0.4165 | [0.3989; 0.4342] | 0.5% | 0.8% |
| Yang F-2012 | 893 | 3771 | 0.2368 | [0.2233; 0.2507] | 0.6% | 0.8% |
| Yang B-2013 | 40 | 597 | 0.0670 | [0.0483; 0.0901] | 0.1% | 0.7% |
| Yang LL-2015 | 966 | 3654 | 0.2644 | [0.2501; 0.2790] | 0.6% | 0.8% |
| Yin YZ-2001 | 63 | 676 | 0.0932 | [0.0724; 0.1177] | 0.1% | 0.7% |
| Yu DS-2011 | 338 | 2429 | 0.1392 | [0.1256; 0.1536] | 0.4% | 0.8% |
| Yu Q-2022 | 183 | 1076 | 0.1701 | [0.1481; 0.1939] | 0.2% | 0.8% |
| Yu WX-2012 | 138 | 5000 | 0.0276 | [0.0232; 0.0325] | 0.8% | 0.8% |
| Zhang D-2022 | 1000 | 6493 | 0.1540 | [0.1453; 0.1630] | 1.1% | 0.8% |
| Zhang LM-2017 | 95 | 739 | 0.1286 | [0.1053; 0.1548] | 0.1% | 0.7% |
| Zhang P-2015 | 47 | 1195 | 0.0393 | [0.0290; 0.0520] | 0.2% | 0.8% |
| Zhang XF-2007 | 2152 | 4139 | 0.5199 | [0.5046; 0.5353] | 0.7% | 0.8% |
| Zhao HL-2012 | 17 | 327 | 0.0520 | [0.0306; 0.0819] | 0.1% | 0.7% |
| Zheng RD-2013 | 207 | 850 | 0.2435 | [0.2150; 0.2738] | 0.1% | 0.8% |
| Zhong CF-2011 | 1074 | 2627 | 0.4088 | [0.3900; 0.4279] | 0.4% | 0.8% |
| Zhong SQ-2007 | 702 | 1239 | 0.5666 | [0.5385; 0.5944] | 0.2% | 0.8% |
| Zhu GZ-2015 | 690 | 3305 | 0.2088 | [0.1950; 0.2230] | 0.6% | 0.8% |
| Zhu GZ-2007 | 1127 | 4944 | 0.2280 | [0.2163; 0.2399] | 0.8% | 0.8% |
| Cai Y-2017 | 366 | 1842 | 0.1987 | [0.1807; 0.2177] | 0.3% | 0.8% |
| Chan DP-2017 | 492 | 1539 | 0.3197 | [0.2964; 0.3436] | 0.3% | 0.8% |
| Chang Y-2009 | 627 | 2819 | 0.2224 | [0.2072; 0.2382] | 0.5% | 0.8% |
| Chen X-2019 | 799 | 4044 | 0.1976 | [0.1854; 0.2102] | 0.7% | 0.8% |
| Chiu DM-2013 | 129 | 450 | 0.2867 | [0.2453; 0.3309] | 0.1% | 0.7% |
| Cong W-2014 | 404 | 1955 | 0.2066 | [0.1889; 0.2253] | 0.3% | 0.8% |
| Cui W-2016 | 228 | 1028 | 0.2218 | [0.1967; 0.2485] | 0.2% | 0.8% |
| Dong C-2012 | 2793 | 14208 | 0.1966 | [0.1901; 0.2032] | 2.4% | 0.8% |
| Feng Y-2018 | 1337 | 1912 | 0.6993 | [0.6782; 0.7198] | 0.3% | 0.8% |
| Fu P-2021 | 249 | 1864 | 0.1336 | [0.1185; 0.1499] | 0.3% | 0.8% |
| Shenyang G-2011 | 37 | 456 | 0.0811 | [0.0578; 0.1101] | 0.1% | 0.7% |
| Geng Y-2019 | 79 | 496 | 0.1593 | [0.1282; 0.1945] | 0.1% | 0.7% |
| Gu G-2015 | 112 | 1491 | 0.0751 | [0.0623; 0.0897] | 0.2% | 0.8% |
| Guo QS-2010 | 14608 | 44816 | 0.3260 | [0.3216; 0.3303] | 7.5% | 0.8% |
| Huang F-2015 | 36 | 388 | 0.0928 | [0.0658; 0.1261] | 0.1% | 0.7% |
| Huang H-2016 | 42 | 391 | 0.1074 | [0.0785; 0.1424] | 0.1% | 0.7% |
| Jia Z-2014 | 3719 | 15852 | 0.2346 | [0.2280; 0.2413] | 2.7% | 0.8% |
| Li H-2021 | 289 | 6269 | 0.0461 | [0.0410; 0.0516] | 1.0% | 0.8% |
| Li M-2020 | 157 | 946 | 0.1660 | [0.1428; 0.1912] | 0.2% | 0.8% |
| Li RC-2006 | 4839 | 10715 | 0.4516 | [0.4422; 0.4611] | 1.8% | 0.8% |
| Liang H-2014 | 129 | 307 | 0.4202 | [0.3644; 0.4776] | 0.1% | 0.7% |
| Liu KSH-2019 | 3408 | 10241 | 0.3328 | [0.3237; 0.3420] | 1.7% | 0.8% |
| Lu J-2009 | 1547 | 8762 | 0.1766 | [0.1686; 0.1847] | 1.5% | 0.8% |
| Ma L-2015 | 87 | 366 | 0.2377 | [0.1950; 0.2847] | 0.1% | 0.7% |
| Ma XX-2021 | 220 | 3568 | 0.0617 | [0.0540; 0.0701] | 0.6% | 0.8% |
| Ma Z-2010 | 407 | 2090 | 0.1947 | [0.1780; 0.2124] | 0.3% | 0.8% |
| Qian Z-2022 | 2275 | 19762 | 0.1151 | [0.1107; 0.1197] | 3.3% | 0.8% |
| Ren F-2013 | 2945 | 10741 | 0.2742 | [0.2658; 0.2827] | 1.8% | 0.8% |
| Rui Z-2018 | 65 | 433 | 0.1501 | [0.1178; 0.1873] | 0.1% | 0.7% |
| Shu Y-2019 | 455 | 1505 | 0.3023 | [0.2792; 0.3262] | 0.3% | 0.8% |
| Tsoi WC-2020 | 315 | 2000 | 0.1575 | [0.1418; 0.1742] | 0.3% | 0.8% |
| Wang M-2017 | 1008 | 4046 | 0.2491 | [0.2359; 0.2628] | 0.7% | 0.8% |
| Wang Y-2018 | 973 | 2592 | 0.3754 | [0.3567; 0.3944] | 0.4% | 0.8% |
| Wen GP-2018 | 1227 | 5345 | 0.2296 | [0.2183; 0.2411] | 0.9% | 0.8% |
| Wong KH-2004 | 176 | 934 | 0.1884 | [0.1638; 0.2150] | 0.2% | 0.8% |
| Xue Y-2013 | 164 | 529 | 0.3100 | [0.2708; 0.3514] | 0.1% | 0.7% |
| Yu Y-2009 | 1299 | 5493 | 0.2365 | [0.2253; 0.2479] | 0.9% | 0.8% |
| Zhang L-2018 | 1002 | 4102 | 0.2443 | [0.2312; 0.2577] | 0.7% | 0.8% |
| Zhang L-2017 | 236 | 600 | 0.3933 | [0.3540; 0.4337] | 0.1% | 0.7% |
| Zhang W-2009 | 117 | 1476 | 0.0793 | [0.0660; 0.0942] | 0.2% | 0.8% |
| Zhuang W-2014 | 113 | 486 | 0.2325 | [0.1956; 0.2727] | 0.1% | 0.7% |
| **Common effect model** | | **597633** | **0.1334** | **[0.1325; 0.1342]** | **100.0%** | **--** |
| **Random effects model** | | | **0.2334** | **[0.2048; 0.2633]** | **--** | **100.0%** |

Heterogeneity: $I^2 = 100\%$, $\tau^2 = 0.0412$, $p = 0$

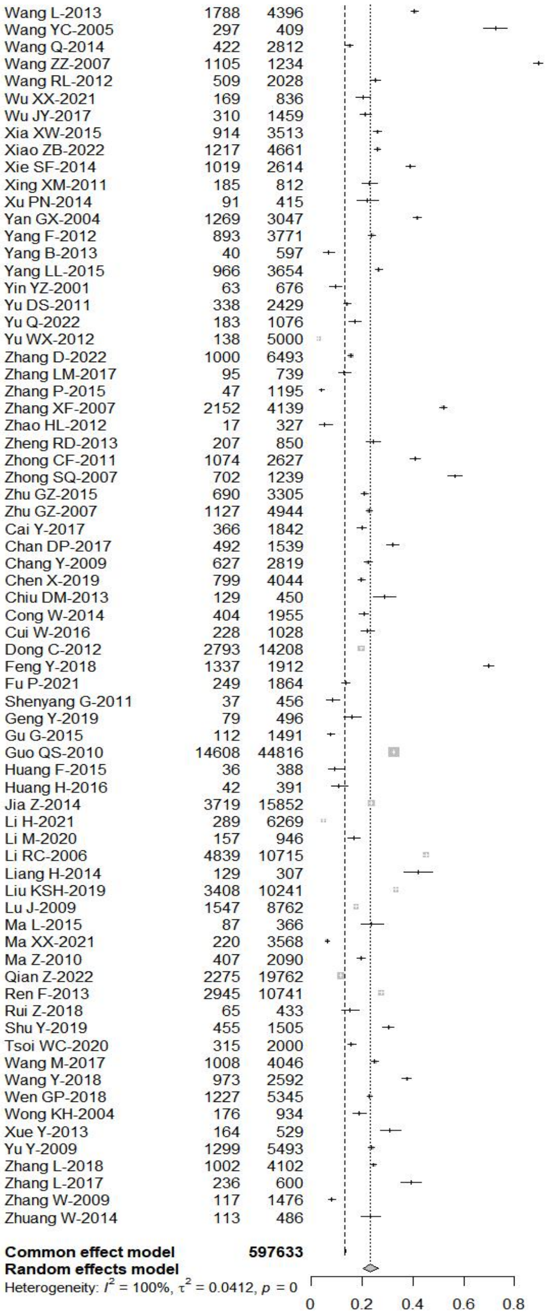


(3)Studies with sample sizes ≤ 300 were excluded

| Study | Events | Total | Proportion | 95%-CI | Weight (common) | Weight (random) |
|---|---|---|---|---|---|---|
| Zhang ZX-2003 | 210 | 574 | 0.3659 | [0.3264; 0.4067] | 0.1% | 0.9% |
| Ai X-2009 | 5588 | 12555 | 0.4451 | [0.4364; 0.4538] | 2.1% | 0.9% |
| Bi L-2008 | 156 | 1204 | 0.1296 | [0.1111; 0.1499] | 0.2% | 0.9% |
| Bo QN-2018 | 127 | 1019 | 0.1246 | [0.1050; 0.1465] | 0.2% | 0.9% |
| Cai YS-2013 | 230 | 510 | 0.4510 | [0.4072; 0.4953] | 0.1% | 0.9% |
| Cao HJ-2004 | 881 | 2290 | 0.3847 | [0.3647; 0.4050] | 0.4% | 0.9% |
| Shen JY-2007 | 749 | 1570 | 0.4771 | [0.4521; 0.5021] | 0.3% | 0.9% |
| Chen XM-2014 | 281 | 868 | 0.3237 | [0.2927; 0.3560] | 0.1% | 0.9% |
| Ning LF-2008 | 1086 | 3561 | 0.3050 | [0.2899; 0.3204] | 0.6% | 0.9% |
| Chen YZ-2006 | 254 | 1084 | 0.2343 | [0.2094; 0.2607] | 0.2% | 0.9% |
| Du JY-2014 | 295 | 952 | 0.3099 | [0.2806; 0.3403] | 0.2% | 0.9% |
| You QZ-2019 | 1113 | 5552 | 0.2005 | [0.1900; 0.2112] | 0.9% | 0.9% |
| Gao DY-2004 | 1804 | 6988 | 0.2582 | [0.2479; 0.2686] | 1.2% | 0.9% |
| Gu HY-2013 | 505 | 6258 | 0.0807 | [0.0741; 0.0877] | 1.1% | 0.9% |
| Yao XF-2013 | 806 | 2012 | 0.4006 | [0.3791; 0.4224] | 0.3% | 0.9% |
| He YW-2018 | 15 | 35857 | 0.0004 | [0.0002; 0.0007] | 6.1% | 0.9% |
| Wu Y-2016 | 482 | 2206 | 0.2185 | [0.2014; 0.2363] | 0.4% | 0.9% |
| Wu JY-2016 | 440 | 1720 | 0.2558 | [0.2353; 0.2771] | 0.3% | 0.9% |
| Wu JY-2016 | 317 | 611 | 0.5188 | [0.4784; 0.5591] | 0.1% | 0.9% |
| Hu AQ-2010 | 377 | 2019 | 0.1867 | [0.1699; 0.2044] | 0.3% | 0.9% |
| Zhou X-2015 | 124 | 912 | 0.1360 | [0.1144; 0.1599] | 0.2% | 0.9% |
| Huang GY-2009 | 1013 | 3044 | 0.3328 | [0.3160; 0.3498] | 0.5% | 0.9% |
| Yao MF-2007 | 635 | 1316 | 0.4825 | [0.4552; 0.5099] | 0.2% | 0.9% |
| Huang SY-2020 | 120 | 648 | 0.1852 | [0.1560; 0.2173] | 0.1% | 0.9% |
| Huang SM-2017 | 273 | 5345 | 0.0511 | [0.0453; 0.0573] | 0.9% | 0.9% |
| Huang XY-2012 | 501 | 2250 | 0.2227 | [0.2056; 0.2404] | 0.4% | 0.9% |
| Meng ZH-2005 | 413 | 980 | 0.4214 | [0.3903; 0.4531] | 0.2% | 0.9% |

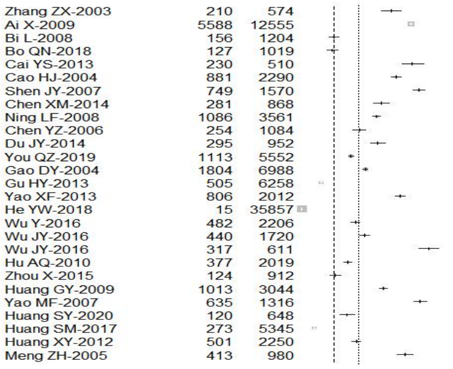

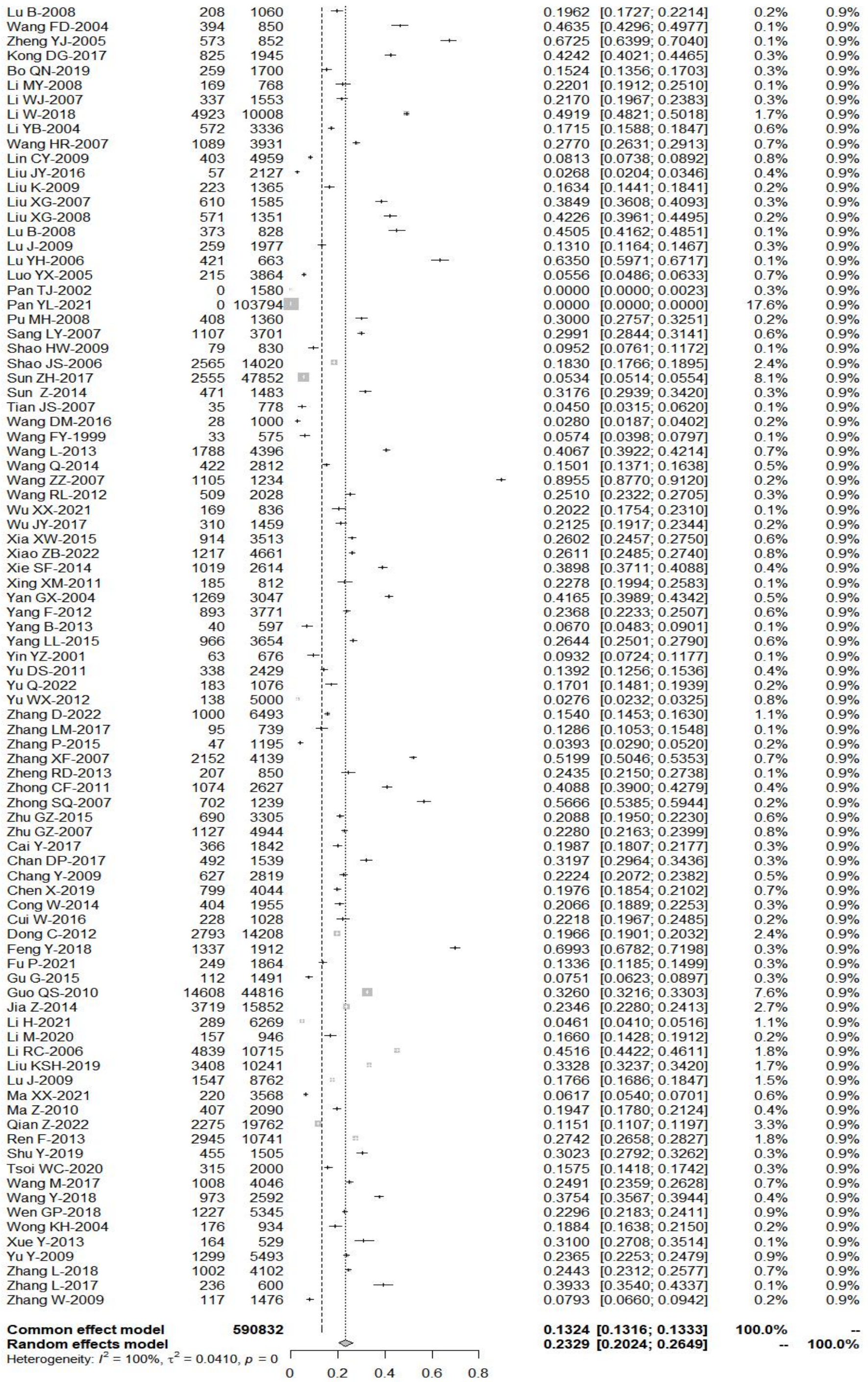


(4)Studies with sample sizes ≤ 500 were excluded

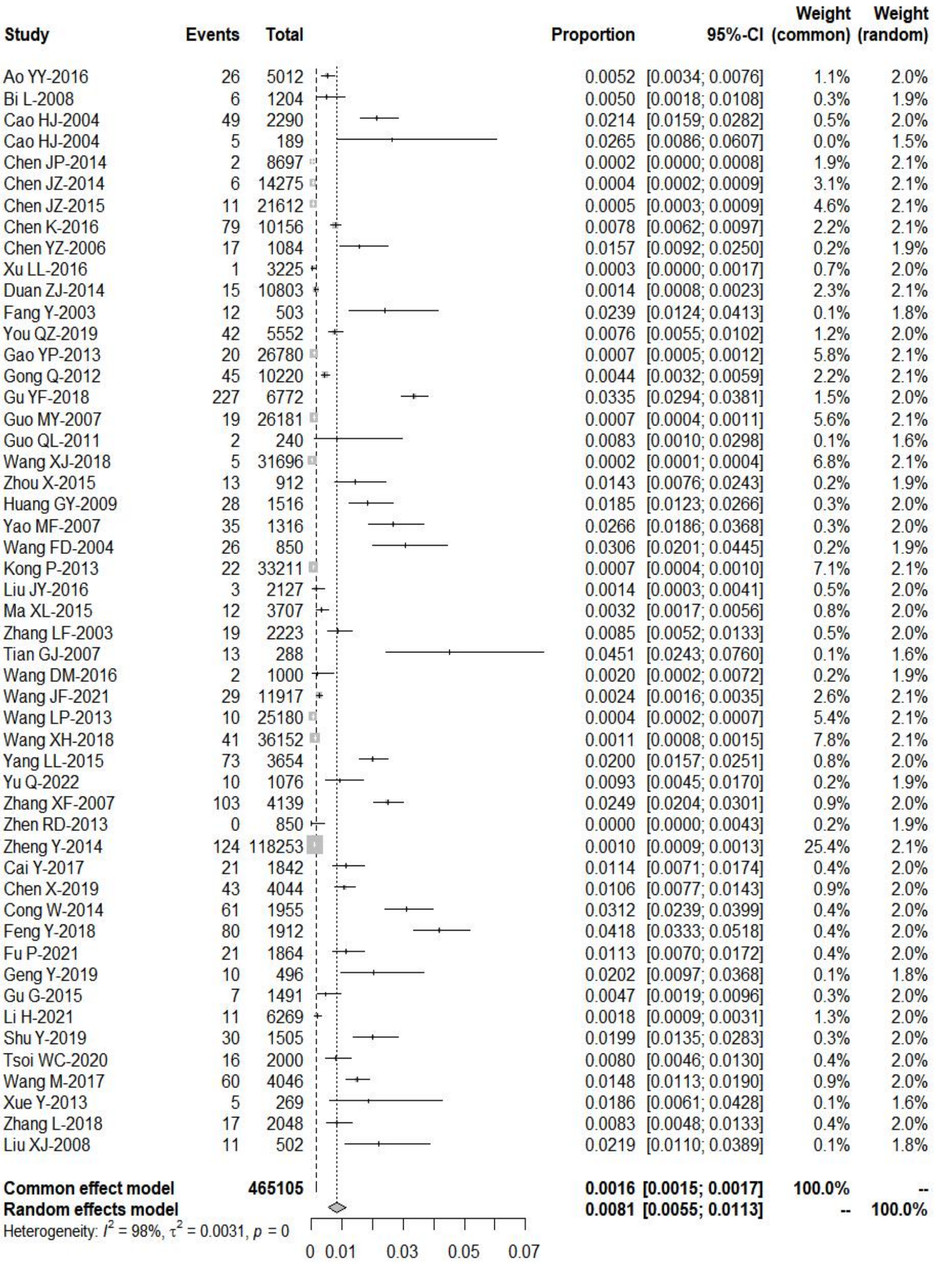


Figure S38. Sensitivity analysis of anti-HEV IgM positivity (1)Studies with a JBI score ≤5 were excluded

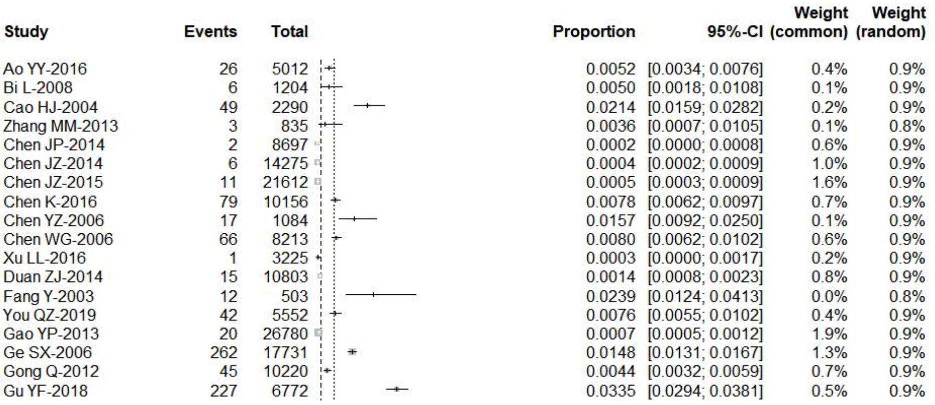

| | | | | | | | |
|---|---|---|---|---|---|---|---|
| Guo MY-2007 | 19 | 26181 | | 0.0007 | [0.0004; 0.0011] | 1.9% | 0.9% |
| Guo QL-2011 | 2 | 240 | | 0.0083 | [0.0010; 0.0298] | 0.0% | 0.7% |
| Wang XJ-2018 | 5 | 31696 | | 0.0002 | [0.0001; 0.0004] | 2.3% | 0.9% |
| Han XX-2010 | 6 | 11362 | | 0.0005 | [0.0002; 0.0011] | 0.8% | 0.9% |
| Hu AQ-2010 | 8 | 2019 | | 0.0040 | [0.0017; 0.0078] | 0.1% | 0.9% |
| Zhou X-2015 | 13 | 912 | | 0.0143 | [0.0076; 0.0243] | 0.1% | 0.8% |
| Huang GY-2009 | 28 | 1516 | | 0.0185 | [0.0123; 0.0266] | 0.1% | 0.9% |
| Yao MF-2007 | 35 | 1316 | | 0.0266 | [0.0186; 0.0368] | 0.1% | 0.9% |
| Huang XY-2012 | 37 | 2250 | | 0.0164 | [0.0116; 0.0226] | 0.2% | 0.9% |
| Wang FD-2004 | 26 | 850 | | 0.0306 | [0.0201; 0.0445] | 0.1% | 0.8% |
| Jiang T-2018 | 26 | 9797 | | 0.0027 | [0.0017; 0.0039] | 0.7% | 0.9% |
| Jin LP-2012 | 6 | 11461 | | 0.0005 | [0.0002; 0.0011] | 0.8% | 0.9% |
| Kong P-2013 | 22 | 33211 | | 0.0007 | [0.0004; 0.0010] | 2.4% | 0.9% |
| Bo QN-2019 | 50 | 1700 | | 0.0294 | [0.0219; 0.0386] | 0.1% | 0.9% |
| Li SB-2010 | 4 | 76654 | | 0.0001 | [0.0000; 0.0001] | 5.5% | 0.9% |
| Li WJ-2007 | 5 | 1553 | | 0.0032 | [0.0010; 0.0075] | 0.1% | 0.9% |
| Li W-2018 | 167 | 10008 | | 0.0167 | [0.0143; 0.0194] | 0.7% | 0.9% |
| Li XJ-2019 | 55 | 16022 | | 0.0034 | [0.0026; 0.0045] | 1.2% | 0.9% |
| Li YH-2021 | 36 | 25098 | | 0.0014 | [0.0010; 0.0020] | 1.8% | 0.9% |
| Li ZR-2005 | 16 | 82670 | | 0.0002 | [0.0001; 0.0003] | 6.0% | 0.9% |
| Li TQ-2016 | 37 | 8257 | | 0.0045 | [0.0032; 0.0062] | 0.6% | 0.9% |
| Lin Q-2012 | 4 | 5681 | | 0.0007 | [0.0002; 0.0018] | 0.4% | 0.9% |
| Liu DX-2019 | 33 | 40182 | | 0.0008 | [0.0006; 0.0012] | 2.9% | 0.9% |
| Liu F-2018 | 6 | 9529 | | 0.0006 | [0.0002; 0.0014] | 0.7% | 0.9% |
| Liu JY-2016 | 3 | 2127 | | 0.0014 | [0.0003; 0.0041] | 0.2% | 0.9% |
| Liu K-2009 | 4 | 1365 | | 0.0029 | [0.0008; 0.0075] | 0.1% | 0.9% |
| Liu XG-2007 | 51 | 1585 | | 0.0322 | [0.0240; 0.0421] | 0.1% | 0.9% |
| Liu Y-2013 | 115 | 25391 | | 0.0045 | [0.0037; 0.0054] | 1.8% | 0.9% |
| Luo Y-2011 | 7 | 31137 | | 0.0002 | [0.0001; 0.0005] | 2.2% | 0.9% |
| Ma SB-2014 | 11 | 5639 | | 0.0020 | [0.0010; 0.0035] | 0.4% | 0.9% |
| Ma XL-2015 | 12 | 3707 | | 0.0032 | [0.0017; 0.0056] | 0.3% | 0.9% |
| Zhang LF-2003 | 19 | 2223 | | 0.0085 | [0.0052; 0.0133] | 0.2% | 0.9% |
| Nong HY-2013 | 7 | 11938 | | 0.0006 | [0.0002; 0.0012] | 0.9% | 0.9% |
| Qiu SH-2013 | 27 | 27977 | | 0.0010 | [0.0006; 0.0014] | 2.0% | 0.9% |
| Sang LY-2007 | 50 | 3701 | | 0.0135 | [0.0100; 0.0178] | 0.3% | 0.9% |
| Shao HW-2009 | 4 | 830 | | 0.0048 | [0.0013; 0.0123] | 0.1% | 0.8% |
| Sun C-2016 | 214 | 128833 | | 0.0017 | [0.0014; 0.0019] | 9.3% | 0.9% |
| Sun JW-2009 | 72 | 5494 | | 0.0131 | [0.0103; 0.0165] | 0.4% | 0.9% |
| Sun LP-2004 | 20 | 1043 | | 0.0192 | [0.0118; 0.0295] | 0.1% | 0.9% |
| Tan PY-1999 | 124 | 1531 | | 0.0810 | [0.0678; 0.0958] | 0.1% | 0.9% |
| Tian GJ-2007 | 13 | 288 | | 0.0451 | [0.0243; 0.0760] | 0.0% | 0.7% |
| Wang DM-2016 | 2 | 1000 | | 0.0020 | [0.0002; 0.0072] | 0.1% | 0.9% |
| Wang JF-2021 | 29 | 11917 | | 0.0024 | [0.0016; 0.0035] | 0.9% | 0.9% |
| Wang LP-2013 | 10 | 25180 | | 0.0004 | [0.0002; 0.0007] | 1.8% | 0.9% |
| Wang XH-2018 | 41 | 36152 | | 0.0011 | [0.0008; 0.0015] | 2.6% | 0.9% |
| Wu ZT-2013 | 14 | 4441 | | 0.0032 | [0.0017; 0.0053] | 0.3% | 0.9% |
| Wu ZH-2017 | 5 | 32120 | | 0.0002 | [0.0001; 0.0004] | 2.3% | 0.9% |
| Wu JY-2017 | 7 | 1459 | | 0.0048 | [0.0019; 0.0099] | 0.1% | 0.9% |
| Xia C-2012 | 11 | 749 | | 0.0147 | [0.0074; 0.0261] | 0.1% | 0.8% |
| Xiao ZY-2016 | 32 | 8952 | | 0.0036 | [0.0024; 0.0050] | 0.6% | 0.9% |
| Xie SF-2014 | 144 | 2614 | | 0.0551 | [0.0467; 0.0645] | 0.2% | 0.9% |
| Xing Y-2016 | 164 | 95217 | | 0.0017 | [0.0015; 0.0020] | 6.9% | 0.9% |
| Xu WL-2018 | 43 | 15897 | | 0.0027 | [0.0020; 0.0036] | 1.1% | 0.9% |
| Yan GX-2004 | 46 | 3047 | | 0.0151 | [0.0111; 0.0201] | 0.2% | 0.9% |
| Yang LL-2015 | 73 | 3654 | | 0.0200 | [0.0157; 0.0251] | 0.3% | 0.9% |
| Yang XY-2016 | 26 | 12271 | | 0.0021 | [0.0014; 0.0031] | 0.9% | 0.9% |
| Yu Q-2022 | 10 | 1076 | | 0.0093 | [0.0045; 0.0170] | 0.1% | 0.9% |
| Yuan ZZ-2022 | 10 | 1604 | | 0.0062 | [0.0030; 0.0114] | 0.1% | 0.9% |
| Zhang LM-2017 | 6 | 739 | | 0.0081 | [0.0030; 0.0176] | 0.1% | 0.8% |
| Zhang P-2015 | 3 | 1195 | | 0.0025 | [0.0005; 0.0073] | 0.1% | 0.9% |
| Zhang WS-2012 | 3 | 6456 | | 0.0005 | [0.0001; 0.0014] | 0.5% | 0.9% |
| Zhang XF-2007 | 103 | 4139 | | 0.0249 | [0.0204; 0.0301] | 0.3% | 0.9% |
| Zhao HL-2012 | 8 | 327 | | 0.0245 | [0.0106; 0.0476] | 0.0% | 0.7% |
| Zhao JN-2017 | 10 | 10000 | | 0.0010 | [0.0005; 0.0018] | 0.7% | 0.9% |
| Zhen RD-2013 | 0 | 850 | | 0.0000 | [0.0000; 0.0043] | 0.1% | 0.8% |
| Zheng Y-2015 | 21 | 26583 | | 0.0008 | [0.0005; 0.0012] | 1.9% | 0.9% |
| Zheng Y-2014 | 124 | 118253 | | 0.0010 | [0.0009; 0.0013] | 8.5% | 0.9% |
| Cai Y-2017 | 21 | 1842 | | 0.0114 | [0.0071; 0.0174] | 0.1% | 0.9% |
| Chen X-2019 | 43 | 4044 | | 0.0106 | [0.0077; 0.0143] | 0.3% | 0.9% |
| Cong W-2014 | 61 | 1955 | | 0.0312 | [0.0239; 0.0399] | 0.1% | 0.9% |
| Dong C-2012 | 4 | 14208 | | 0.0003 | [0.0001; 0.0007] | 1.0% | 0.9% |
| Feng Y-2018 | 80 | 1912 | | 0.0418 | [0.0333; 0.0518] | 0.1% | 0.9% |
| Fu P-2021 | 21 | 1864 | | 0.0113 | [0.0070; 0.0172] | 0.1% | 0.9% |
| Geng Y-2019 | 10 | 496 | | 0.0202 | [0.0097; 0.0368] | 0.0% | 0.8% |
| Gu G-2015 | 7 | 1491 | | 0.0047 | [0.0019; 0.0096] | 0.1% | 0.9% |
| Guo QS-2010 | 420 | 44816 | | 0.0094 | [0.0085; 0.0103] | 3.2% | 0.9% |
| Huang F-2013 | 4 | 293 | | 0.0137 | [0.0037; 0.0346] | 0.0% | 0.7% |
| Huang F-2015 | 15 | 388 | | 0.0387 | [0.0218; 0.0630] | 0.0% | 0.8% |
| Li H-2021 | 11 | 6269 | | 0.0018 | [0.0009; 0.0031] | 0.5% | 0.9% |
| Li M-2020 | 42 | 946 | | 0.0444 | [0.0322; 0.0595] | 0.1% | 0.9% |
| Ma XX-2021 | 21 | 3568 | | 0.0059 | [0.0036; 0.0090] | 0.3% | 0.9% |
| Ma Z-2010 | 9 | 2090 | | 0.0043 | [0.0020; 0.0082] | 0.2% | 0.9% |
| Qian Z-2022 | 50 | 19762 | | 0.0025 | [0.0019; 0.0033] | 1.4% | 0.9% |
| Ren F-2013 | 109 | 10741 | | 0.0101 | [0.0083; 0.0122] | 0.8% | 0.9% |
| Rui Z-2018 | 8 | 433 | | 0.0185 | [0.0080; 0.0361] | 0.0% | 0.8% |
| Shu Y-2019 | 30 | 1505 | | 0.0199 | [0.0135; 0.0283] | 0.1% | 0.9% |
| Tsoi WC-2020 | 16 | 2000 | | 0.0080 | [0.0046; 0.0130] | 0.1% | 0.9% |
| Wang M-2017 | 60 | 4046 | | 0.0148 | [0.0113; 0.0190] | 0.3% | 0.9% |
| Wang Y-2018 | 16 | 2592 | | 0.0062 | [0.0035; 0.0100] | 0.2% | 0.9% |
| Wen GP-2018 | 38 | 5345 | | 0.0071 | [0.0050; 0.0097] | 0.4% | 0.9% |
| Xue Y-2013 | 5 | 269 | | 0.0186 | [0.0061; 0.0428] | 0.0% | 0.7% |
| Yu Y-2009 | 71 | 4979 | | 0.0143 | [0.0112; 0.0180] | 0.4% | 0.9% |
| Zhang L-2018 | 17 | 2048 | | 0.0083 | [0.0048; 0.0133] | 0.1% | 0.9% |
| Zhang W-2009 | 29 | 1476 | | 0.0196 | [0.0132; 0.0281] | 0.1% | 0.9% |
| Liu XJ-2008 | 11 | 502 | | 0.0219 | [0.0110; 0.0389] | 0.0% | 0.8% |
| **Common effect model** | | **1387710** | | **0.0017** | **[0.0016; 0.0018]** | **100.0%** | **--** |
| **Random effects model** | | | | **0.0071** | **[0.0053; 0.0090]** | **--** | **100.0%** |

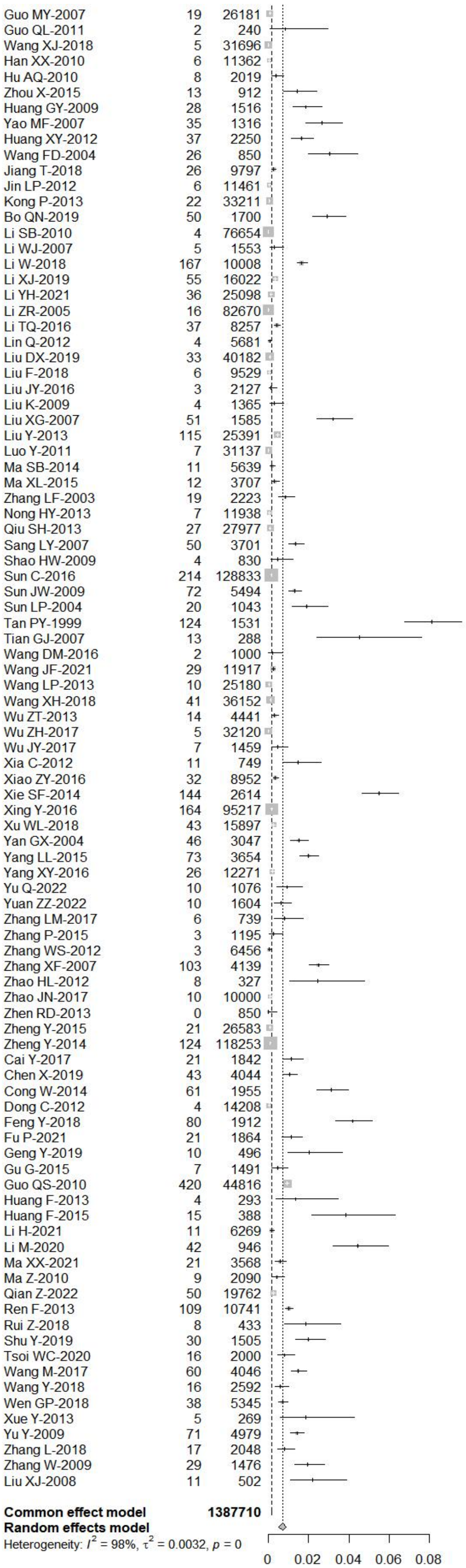


Heterogeneity: $I^2 = 98\%$, $\tau^2 = 0.0032$, $p = 0$

(2) Studies with sample sizes ≤ 200 were excluded

| Study | Events | Total | Proportion | 95%-CI | Weight (common) | Weight (random) |
|---|---|---|---|---|---|---|
| Ao YY-2016 | 26 | 5012 | 0.0052 | [0.0034; 0.0076] | 0.4% | 0.9% |
| Bi L-2008 | 6 | 1204 | 0.0050 | [0.0018; 0.0108] | 0.1% | 0.9% |
| Cao HJ-2004 | 49 | 2290 | 0.0214 | [0.0159; 0.0282] | 0.2% | 0.9% |
| Zhang MM-2013 | 3 | 835 | 0.0036 | [0.0007; 0.0105] | 0.1% | 0.9% |
| Chen JP-2014 | 2 | 8697 | 0.0002 | [0.0000; 0.0008] | 0.6% | 0.9% |
| Chen JZ-2014 | 6 | 14275 | 0.0004 | [0.0002; 0.0009] | 1.0% | 0.9% |
| Chen JZ-2015 | 11 | 21612 | 0.0005 | [0.0003; 0.0009] | 1.6% | 0.9% |
| Chen K-2016 | 79 | 10156 | 0.0078 | [0.0062; 0.0097] | 0.7% | 0.9% |
| Chen YZ-2006 | 17 | 1084 | 0.0157 | [0.0092; 0.0250] | 0.1% | 0.9% |
| Chen WG-2006 | 66 | 8213 | 0.0080 | [0.0062; 0.0102] | 0.6% | 0.9% |
| Xu LL-2016 | 1 | 3225 | 0.0003 | [0.0000; 0.0017] | 0.2% | 0.9% |
| Duan ZJ-2014 | 15 | 10803 | 0.0014 | [0.0008; 0.0023] | 0.8% | 0.9% |
| Fang Y-2003 | 12 | 503 | 0.0239 | [0.0124; 0.0413] | 0.0% | 0.8% |
| You QZ-2019 | 42 | 5552 | 0.0076 | [0.0055; 0.0102] | 0.4% | 0.9% |
| Gao YP-2013 | 20 | 26780 | 0.0007 | [0.0005; 0.0012] | 1.9% | 0.9% |
| Ge SX-2006 | 262 | 17731 | 0.0148 | [0.0131; 0.0167] | 1.3% | 0.9% |
| Gong Q-2012 | 45 | 10220 | 0.0044 | [0.0032; 0.0059] | 0.7% | 0.9% |
| Gu YF-2018 | 227 | 6772 | 0.0335 | [0.0294; 0.0381] | 0.5% | 0.9% |
| Guo MY-2007 | 19 | 26181 | 0.0007 | [0.0004; 0.0011] | 1.9% | 0.9% |
| Wang XJ-2018 | 5 | 31696 | 0.0002 | [0.0001; 0.0004] | 2.3% | 0.9% |
| Han XX-2010 | 6 | 11362 | 0.0005 | [0.0002; 0.0011] | 0.8% | 0.9% |
| Hu AQ-2010 | 8 | 2019 | 0.0040 | [0.0017; 0.0078] | 0.1% | 0.9% |
| Zhou X-2015 | 13 | 912 | 0.0143 | [0.0076; 0.0243] | 0.1% | 0.9% |
| Huang GY-2009 | 28 | 1516 | 0.0185 | [0.0123; 0.0266] | 0.1% | 0.9% |
| Yao MF-2007 | 35 | 1316 | 0.0266 | [0.0186; 0.0368] | 0.1% | 0.9% |
| Huang XY-2012 | 37 | 2250 | 0.0164 | [0.0116; 0.0226] | 0.2% | 0.9% |
| Wang FD-2004 | 26 | 850 | 0.0306 | [0.0201; 0.0445] | 0.1% | 0.9% |
| Jiang T-2018 | 26 | 9797 | 0.0027 | [0.0017; 0.0039] | 0.7% | 0.9% |
| Jin LP-2012 | 6 | 11461 | 0.0005 | [0.0002; 0.0011] | 0.8% | 0.9% |
| Kong P-2013 | 22 | 33211 | 0.0007 | [0.0004; 0.0010] | 2.4% | 0.9% |
| Bo QN-2019 | 50 | 1700 | 0.0294 | [0.0219; 0.0386] | 0.1% | 0.9% |
| Li SB-2010 | 4 | 76654 | 0.0001 | [0.0000; 0.0001] | 5.5% | 0.9% |
| Li WJ-2007 | 5 | 1553 | 0.0032 | [0.0010; 0.0075] | 0.1% | 0.9% |
| Li W-2018 | 167 | 10008 | 0.0167 | [0.0143; 0.0194] | 0.7% | 0.9% |
| Li XJ-2019 | 55 | 16022 | 0.0034 | [0.0026; 0.0045] | 1.2% | 0.9% |
| Li YH-2021 | 36 | 25098 | 0.0014 | [0.0010; 0.0020] | 1.8% | 0.9% |
| Li ZR-2005 | 16 | 82670 | 0.0002 | [0.0001; 0.0003] | 6.0% | 0.9% |
| Li TQ-2016 | 37 | 8257 | 0.0045 | [0.0032; 0.0062] | 0.6% | 0.9% |
| Lin Q-2012 | 4 | 5681 | 0.0007 | [0.0002; 0.0018] | 0.4% | 0.9% |
| Liu DX-2019 | 33 | 40182 | 0.0008 | [0.0006; 0.0012] | 2.9% | 0.9% |
| Liu F-2018 | 6 | 9529 | 0.0006 | [0.0002; 0.0014] | 0.7% | 0.9% |
| Liu JY-2016 | 3 | 2127 | 0.0014 | [0.0003; 0.0041] | 0.2% | 0.9% |
| Liu K-2009 | 4 | 1365 | 0.0029 | [0.0008; 0.0075] | 0.1% | 0.9% |
| Liu XG-2007 | 51 | 1585 | 0.0322 | [0.0240; 0.0421] | 0.1% | 0.9% |
| Liu Y-2013 | 115 | 25391 | 0.0045 | [0.0037; 0.0054] | 1.8% | 0.9% |
| Luo Y-2011 | 7 | 31137 | 0.0002 | [0.0001; 0.0005] | 2.2% | 0.9% |
| Ma SB-2014 | 11 | 5639 | 0.0020 | [0.0010; 0.0035] | 0.4% | 0.9% |
| Ma XL-2015 | 12 | 3707 | 0.0032 | [0.0017; 0.0056] | 0.3% | 0.9% |
| Zhang LF-2003 | 19 | 2223 | 0.0085 | [0.0052; 0.0133] | 0.2% | 0.9% |
| Nong HY-2013 | 7 | 11938 | 0.0006 | [0.0002; 0.0012] | 0.9% | 0.9% |
| Qiu SH-2013 | 27 | 27977 | 0.0010 | [0.0006; 0.0014] | 2.0% | 0.9% |
| Sang LY-2007 | 50 | 3701 | 0.0135 | [0.0100; 0.0178] | 0.3% | 0.9% |
| Shao HW-2009 | 4 | 830 | 0.0048 | [0.0013; 0.0123] | 0.1% | 0.9% |
| Sun C-2016 | 214 | 128833 | 0.0017 | [0.0014; 0.0019] | 9.3% | 0.9% |
| Sun JW-2009 | 72 | 5494 | 0.0131 | [0.0103; 0.0165] | 0.4% | 0.9% |
| Sun LP-2004 | 20 | 1043 | 0.0192 | [0.0118; 0.0295] | 0.1% | 0.9% |
| Tan PY-1999 | 124 | 1531 | 0.0810 | [0.0678; 0.0958] | 0.1% | 0.9% |
| Wang DM-2016 | 2 | 1000 | 0.0020 | [0.0002; 0.0072] | 0.1% | 0.9% |
| Wang JF-2021 | 29 | 11917 | 0.0024 | [0.0016; 0.0035] | 0.9% | 0.9% |
| Wang LP-2013 | 10 | 25180 | 0.0004 | [0.0002; 0.0007] | 1.8% | 0.9% |
| Wang XH-2018 | 41 | 36152 | 0.0011 | [0.0008; 0.0015] | 2.6% | 0.9% |
| Wu ZT-2013 | 14 | 4441 | 0.0032 | [0.0017; 0.0053] | 0.3% | 0.9% |
| Wu ZH-2017 | 5 | 32120 | 0.0002 | [0.0001; 0.0004] | 2.3% | 0.9% |
| Wu JY-2017 | 7 | 1459 | 0.0048 | [0.0019; 0.0099] | 0.1% | 0.9% |
| Xia C-2012 | 11 | 749 | 0.0147 | [0.0074; 0.0261] | 0.1% | 0.9% |
| Xiao ZY-2016 | 32 | 8952 | 0.0036 | [0.0024; 0.0050] | 0.6% | 0.9% |
| Xie SF-2014 | 144 | 2614 | 0.0551 | [0.0467; 0.0645] | 0.2% | 0.9% |
| Xing Y-2016 | 164 | 95217 | 0.0017 | [0.0015; 0.0020] | 6.9% | 0.9% |
| Xu WL-2018 | 43 | 15897 | 0.0027 | [0.0020; 0.0036] | 1.1% | 0.9% |
| Yan GX-2004 | 46 | 3047 | 0.0151 | [0.0111; 0.0201] | 0.2% | 0.9% |
| Yang LL-2015 | 73 | 3654 | 0.0200 | [0.0157; 0.0251] | 0.3% | 0.9% |
| Yang XY-2016 | 26 | 12271 | 0.0021 | [0.0014; 0.0031] | 0.9% | 0.9% |
| Yu Q-2022 | 10 | 1076 | 0.0093 | [0.0045; 0.0170] | 0.1% | 0.9% |
| Yuan ZZ-2022 | 10 | 1604 | 0.0062 | [0.0030; 0.0114] | 0.1% | 0.9% |
| Zhang LM-2017 | 6 | 739 | 0.0081 | [0.0030; 0.0176] | 0.1% | 0.9% |
| Zhang P-2015 | 3 | 1195 | 0.0025 | [0.0005; 0.0073] | 0.1% | 0.9% |
| Zhang WS-2012 | 3 | 6456 | 0.0005 | [0.0001; 0.0014] | 0.5% | 0.9% |
| Zhang XF-2007 | 103 | 4139 | 0.0249 | [0.0204; 0.0301] | 0.3% | 0.9% |
| Zhao HL-2012 | 8 | 327 | 0.0245 | [0.0106; 0.0476] | 0.0% | 0.8% |
| Zhao JN-2017 | 10 | 10000 | 0.0010 | [0.0005; 0.0018] | 0.7% | 0.9% |
| Zhen RD-2013 | 0 | 850 | 0.0000 | [0.0000; 0.0043] | 0.1% | 0.9% |
| Zheng Y-2015 | 21 | 26583 | 0.0008 | [0.0005; 0.0012] | 1.9% | 0.9% |
| Zheng Y-2014 | 124 | 118253 | 0.0010 | [0.0009; 0.0013] | 8.5% | 0.9% |
| Cai Y-2017 | 21 | 1842 | 0.0114 | [0.0071; 0.0174] | 0.1% | 0.9% |
| Chen X-2019 | 43 | 4044 | 0.0106 | [0.0077; 0.0143] | 0.3% | 0.9% |
| Cong W-2014 | 61 | 1955 | 0.0312 | [0.0239; 0.0399] | 0.1% | 0.9% |
| Dong C-2012 | 4 | 14208 | 0.0003 | [0.0001; 0.0007] | 1.0% | 0.9% |
| Feng Y-2018 | 80 | 1912 | 0.0418 | [0.0333; 0.0518] | 0.1% | 0.9% |

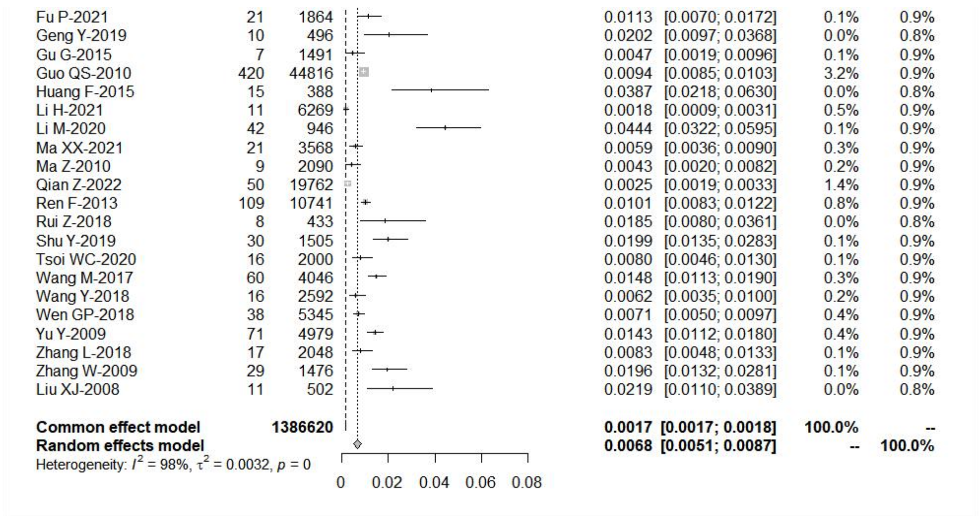


(3) Studies with sample sizes ≤ 300 were excluded

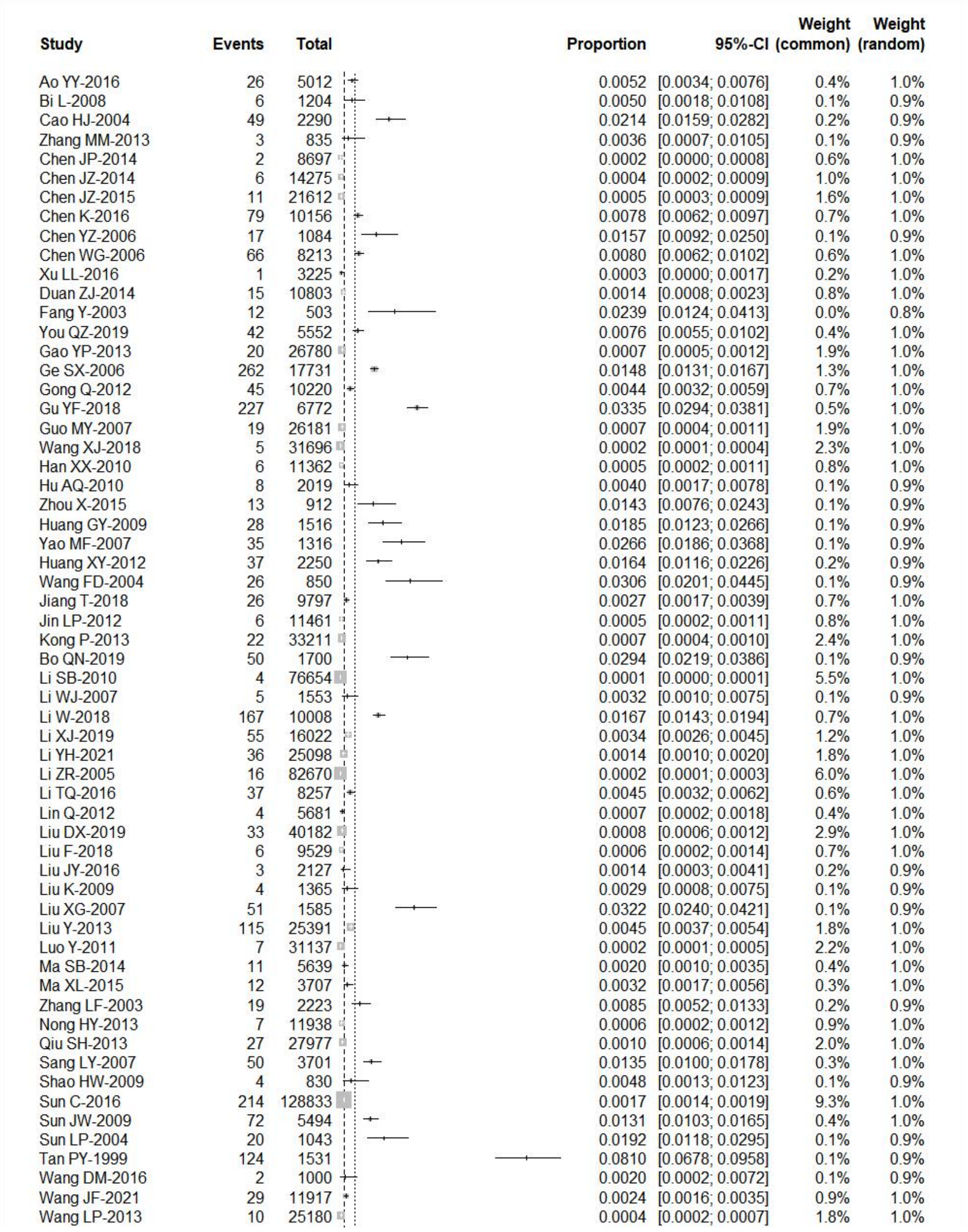

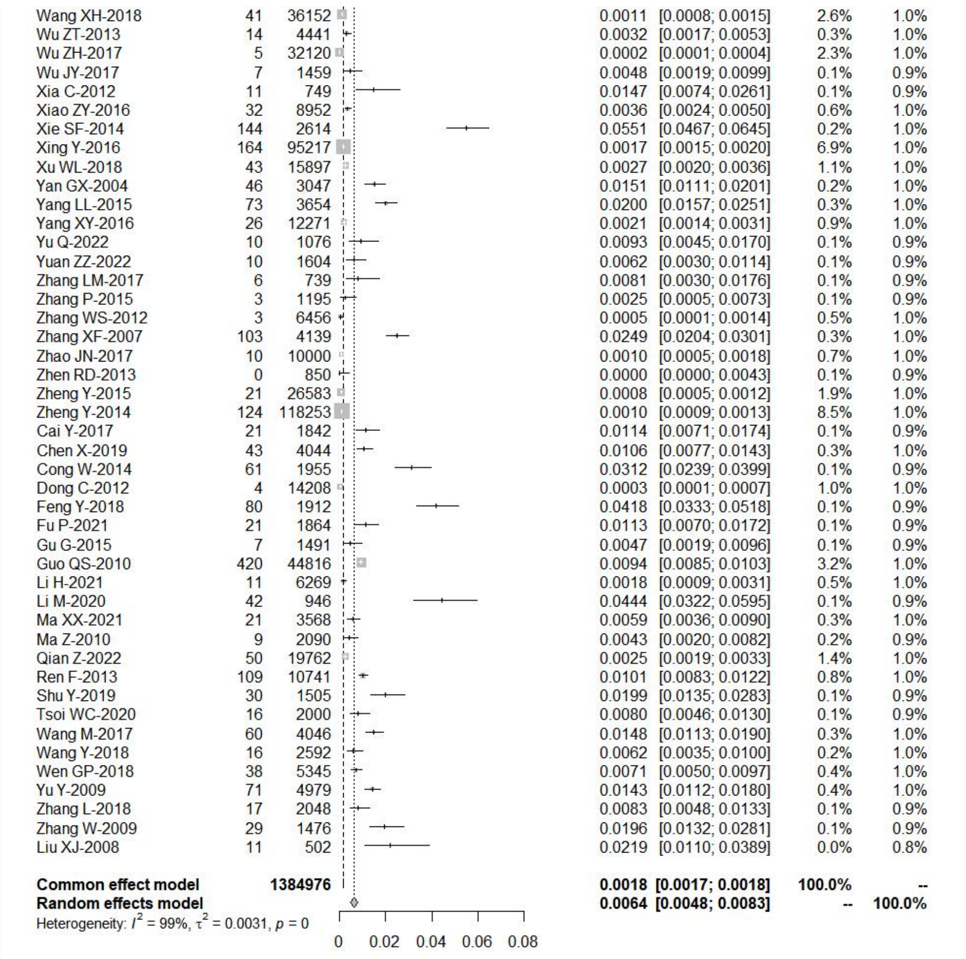


(4) Studies with sample sizes ≤ 500 were excluded

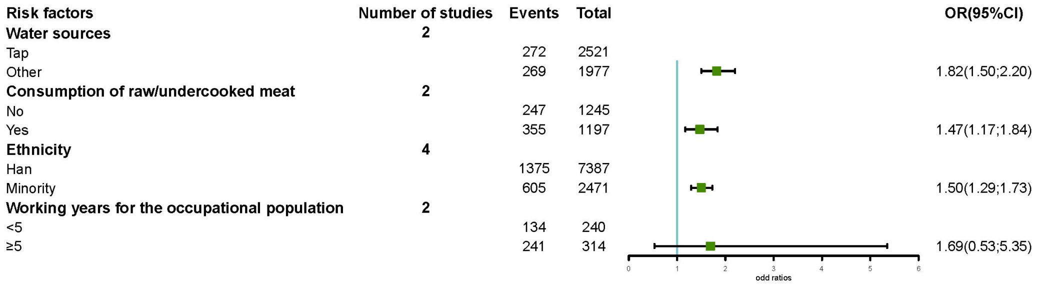


Figure 39. risk factors of anti-HEV IgG positive rate

## 1.2 Supplementary Tables

Table S1. Score of studies evaluated by JBI Critical Appraisal Tools

1. Was the sample frame appropriate to address the target population?
2. Were study participants sampled in an appropriate way?
3. Was the sample size adequate?
4. Were the study subjects and the setting described in detail?
5. Was the data analysis conducted with sufficient coverage of the identified sa mple?
6. Were valid methods used for the identification of the condition?
7. Was the condition measured in a standard, reliable way for all participants?
8. Was there appropriate statistical analysis?
9. Was the response rate adequate, and if not, was the low response rate mana ged appropriately?

Table S2. Univariable and multivariable meta-regression analysis of anti-HEV I gM positive rates

| Variable | Univariable regression | | Multivariable regression | | |
|---|---|---|---|---|---|
| | β (95%CI) | *P* value | β (95%CI) | *P* value | OR (95%CI) |
| **Population** | | | | | |
| The general population (reference) | | | | | |
| Occupational population[a] | 0.0554 (0.0163, 0.0946) | 0.0055 * | | | |
| Pregnant women[a] | 0.0675 (0.0306, 0.1044) | 0.0003 * | | | |
| Hospital attendees | 0.0683 (0.0307, 0.1059) | 0.0004 * | -0.0399 (-0.0805, 0.0006) | 0.0535 | |
| Volunteer blood donors | 0.0402 (0.0134, 0.0670) | 0.0033 * | 0.0365 (-0.0147, 0.0878) | 0.1624 | |
| **Age (yr)** | | | | | |
| 0-9 (reference) | | | | | |
| 10-19 | -0.0112 (-0.0887, -0.0663) | 0.7769 | | | |
| 20-29 | -0.0161 (-0.0871, -0.0549) | 0.6573 | | | |
| 30-39 | -0.0044 (-0.0753, -0.0664) | 0.9026 | | | |
| 40-49 | 0.0218 (-0.0494, -0.0929) | 0.5488 | | | |
| 50-59 | 0.0297 (-0.0457, -0.1050) | 0.4404 | | | |
| 60+ | 0.0424 (-0.0338, -0.1186) | 0.2752 | | | |
| **Gender** | | | | | |
| Female (reference) | | | | | |
| Male | 0.0018 (-0.0197, 0.0234) | 0.8669 | | | |
| **Type of kits** | | | | | |
| Other (reference) | | | | | |
| WanTai | 0.0404 (0.0175, | 0.0005 | -0.0956 (-0.1499, | 0.0006* | 0.91 (0.86, |

| | | | | | |
|---|---|---|---|---|---|
| | 0.0633) | * | -0.0413) | | 0.96) |
| **Region_1** | | | | | |
| The north(reference) | | | | | |
| The south | 0.0298 (0.0081, 0.0514) | 0.0071 * | 0.0583 (0.0282, 0.0884) | 0.0001* | 1.06 (1.03, 1.09) |
| **Region_2** | | | | | |
| Coastal provinces (reference) | | | | | |
| Inland regions | -0.0100 (-0.0321, 0.0122) | 0.3788 | | | |
| **Region_3** | | | | | |
| Other (reference) | | | | | |
| The west | -0.0259 (-0.0502, -0.0016) | 0.0369 | | | |
| **Study period**[a] | | | | | |
| 1997-2000 (reference) | | | | | |
| 2001-2005 | -0.1747 (-0.2832, -0.0663) | 0.0016 * | | | |
| 2006-2011 | -0.2148 (-0.3221, -0.1076) | <0.0001* | | | |
| 2012-2017 | -0.2034 (-0.3104, -0.0964) | 0.0002 * | | | |
| 2018-2022 | -0.2175 (-0.3285, -0.1065) | 0.0001 * | | | |
| **Urban_rural distribution** | | | | | |
| Rural (reference) | | | | | |
| Urban | -0.0142 (-0.0312, 0.0028) | 0.1025 | | | |
